# Supplementary material for: Thermal imaging using sulfur polymer optics
Source: Nat Commun. 2026 Feb 18;17:1561. doi: 10.1038/s41467-026-68889-0 (PMC12916755; doi:10.1038/s41467-026-68889-0)
Supplement: Supplementary file 1 — Supplementary Information [file 41467_2026_68889_MOESM1_ESM.pdf]

Supplementary Information

Thermal Imaging Using Sulfur Polymer Optics

Samuel J. Tonkin,[1] Harshal D. Patel,[1] Jasmine M. M. Pople,[1] Le Nhan Pham,[1] Daniel J. Lewis,[1] Batool A. Aljubran,[1] Jason R. Gascooke,[1,2] Christopher T. Gibson,[3] Tilak Hewagama,[4,5] Donald E. Jennings,[4,6] Frank T. Ferguson,[4,7] Martin R. Johnston,[1] Witold M. Bloch,[1] Alex C. Bissember,[8] Zhongfan Jia,[1] Michelle L. Coote[1] and Justin M. Chalker[1,*]

1) College of Science and Engineering, Flinders University, Bedford Park, Adelaide, South Australia 5042, Australia

2) Australian National Fabrication Facility, South Australia Node, College of Science and Engineering, Flinders University, Bedford Park, Adelaide, South Australia 5042, Australia

3) Adelaide Microscopy, The University of Adelaide, Adelaide, South Australia 5000, Australia

4) NASA Goddard Space Flight Center, Greenbelt, MD 20771, USA

5) Department of Astronomy, University of Maryland, College Park, MD 20742, USA

6) Science Systems and Applications, Inc. (SSAI), Lanham, Maryland 20706 USA

7) Catholic University of America, Washington, DC 20064, USA

8) School of Natural Sciences—Chemistry, University of Tasmania, Hobart, Tasmania 7001, Australia

E-mail: justin.chalker@flinders.edu.au

Table of Contents

Supplementary Figures A-D 4

SUPPLEMENTARY METHODS AND CHARACTERIZATION
General Considerations 8

Synthesis and characterization of polymers prepared directly from norbornadiene 11
Solventless reaction between sulfur and norbornadiene 11
Characterization of material made from solventless reaction 12
Polymer synthesis in solvent 13
Reduction of polymers prepared in solvent 14
FTIR analysis of polymer prepared using solvent 16
Glass transition temperature by DMTA of polymers prepared using solvent 17
Glass transition temperature by DSC of polymers prepared using solvent 19
Infrared transparency of polymers prepared in solvent 21
Refractive index of polymers prepared in solvent 23

Synthesis and isolation of norbornadiene derived cyclic sulfides 24
Extraction of hexane soluble intermediates and analysis with GC-MS 24
Method used to synthesize cyclic sulfide intermediates 30
Purification of intermediates 31
Synthesis and purification of molecules 9 and 10 37
X-ray crystallography of isolated cyclic sulfides 38
Thermal ellipsoid plots 39
ATR-FTIR analysis of cyclic sulfides 41

Ball Milling of Norbornadiene and Sulfur 42
Ball milling method 42
Characterization of ball milled material 43
Reduction and GC-MS analysis of ball milled norbornadiene and sulfur 45

Polymer synthesis and characterization using cyclic sulfides as monomers 49
Polymerization method using cyclic sulfides 49
Optimized polymerization method using 9 and 10 to prepare polymer 1 51
ATR-FTIR of polymers made with purified norbornadiene-derived sulfides 53
Reduction of polymers and analysis with GC-MS 57
Differential scanning calorimetry of polymers made from monomers 2, 6-9 66
Dynamic mechanical thermal analysis of polymer 1 71
Solid state NMR spectra of polymer 1 made from bistrisulfide 9 72
Raman spectroscopy of polymer 1 73
Thermal depolymerization of polymer 1 75
Potassium thioacetate catalyzed depolymerization of polymer 1 76
Infrared transparency of polymer 1 at a range of thicknesses 77
Refractive index testing of polymer 1 83

Theoretical procedures and computational study of polymer 1 85
Computational methods 85
Convergence of IR spectra at DFT 85
Integrated simulated spectra 87
IR spectra of large polymer matrices 88
IR spectra of other monomers 89

Preparation of lenses for FLIR Lepton 3.5 90
Lens designs 90
Preparation of molds and lens casting 92
Overview of lens designs 93
Design of mount for FLIR Lepton 3.5 96
Mask designs 97
Focal length testing 99
Lens resolution testing 103
Thermal sensitivity testing 106
Noise equivalent thermal difference (NETD) testing 109
Relative illumination testing 111
Overview of imaging using polymer lenses on FLIR Lepton 3.5 module 114
Compression molded lenses 115
Imaging with compression molded lenses 117

Long-term stability of polymer 1 and polymer 1 lenses 118
DSC analysis of polymer 1 and aged polymer 1 118
TGA analysis of polymer 1 and aged polymer 1 119
Refractive index testing of polymer 1 and aged polymer 1 120
Imaging with a 1 year old polymer lens 121

Comparative assessment of sulfur-derived polymer optics for LWIR imaging 122

Supplementary references 127

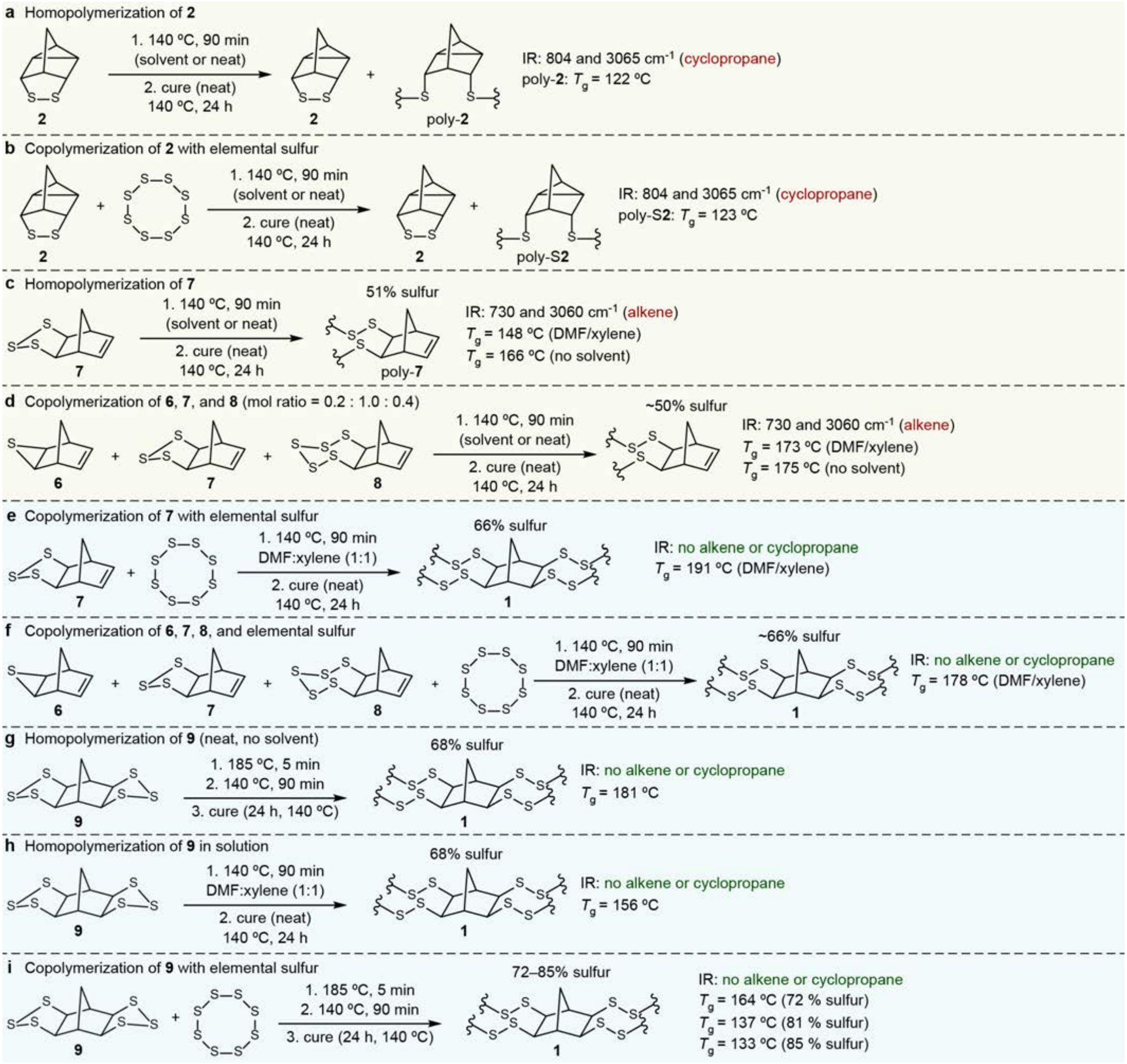

Supplementary Figure A | Polymerization of intermediates. Monomers **2**, **6**, **7**, **8**, and **9** are intermediates formed in the reaction of sulfur with norbornadiene. All of these compounds are capable of forming polymers under these reaction conditions. **a–b,** The cyclopropane unit of **2** remains intact in the polymerization and is strongly absorbing at 804 and 3065 cm^{-1}, which is detrimental to LWIR transparency. **c–f,** Cyclic sulfides **6**, **7**, and **8** can be copolymerized. Elemental sulfur is required to consume the alkene in these reactions which is otherwise strongly absorbing at 730 and 3060 cm^{-1}. **g–i,** Monomer **9** can be hompolymerized or copolymerized with molten sulfur. These reactions provide direct access to target polymer **1** using alkene-free monomers. No cyclopropane rearrangement products are formed.

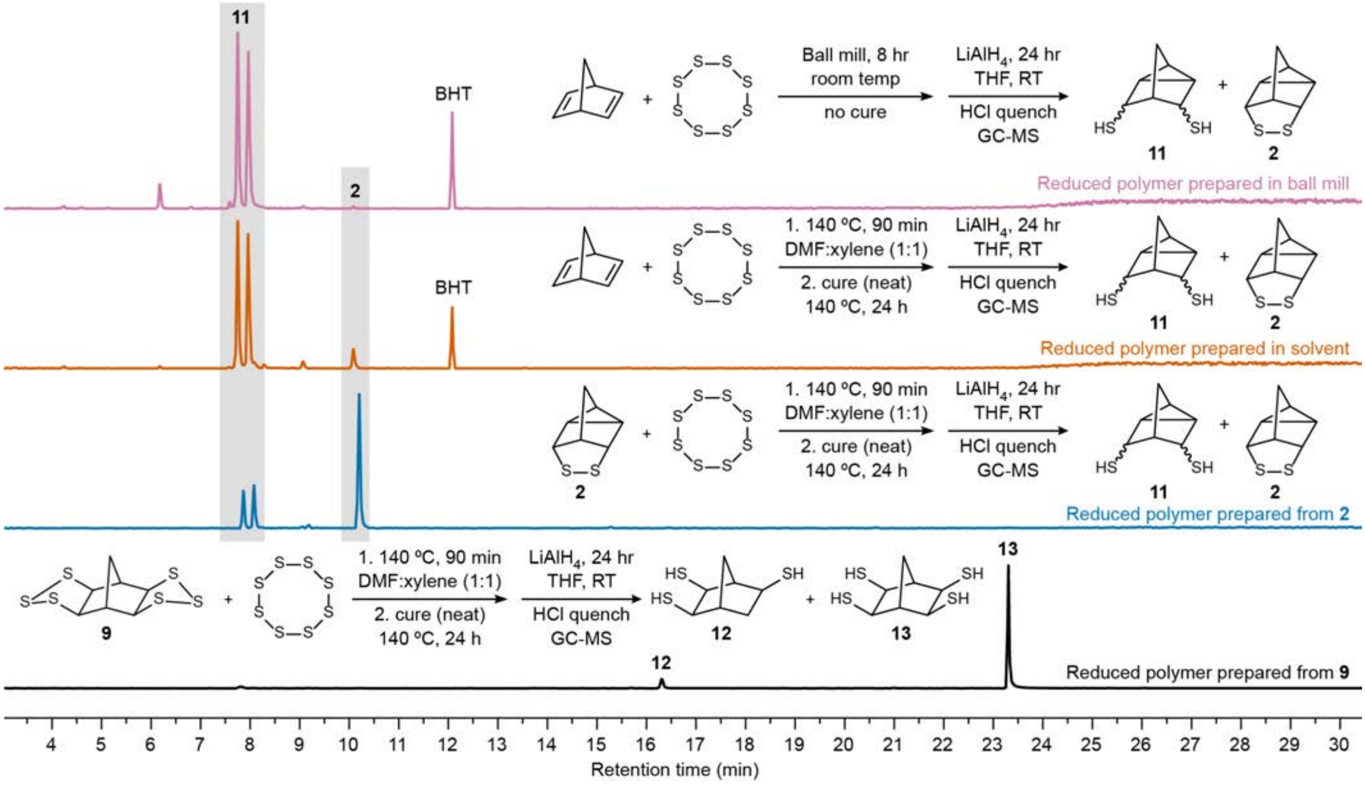

Supplementary Figure B | Reduction of polymers with lithium aluminum hydride and analysis by GC-MS. Polymers were made directly from norbornadiene and sulfur in a ball mill or in a solvent mixture of DMF and xylene (1:1 ratio). Other polymers were prepared from a reaction between **2** or **9** with sulfur in a solvent of DMF and xylene in a 1:1 ratio. All polymers were then reduced with lithium aluminum hydride and analyzed by GC-MS to study the microstructures formed in the polymerization. Polymers prepared from norbornadiene or **2** formed the same cyclopropane products, as evidenced by the detection of **11** and **2** after polymer reduction. After reducing the polymer made from **9**, products **12** and **13** formed; these products are the expected products formed after reducing the target polymer **1**. 2,6-Di-*tert*-butyl-4-methylphenol (BHT) is a stabilizer in the tetrahydrofuran (THF) solvent.

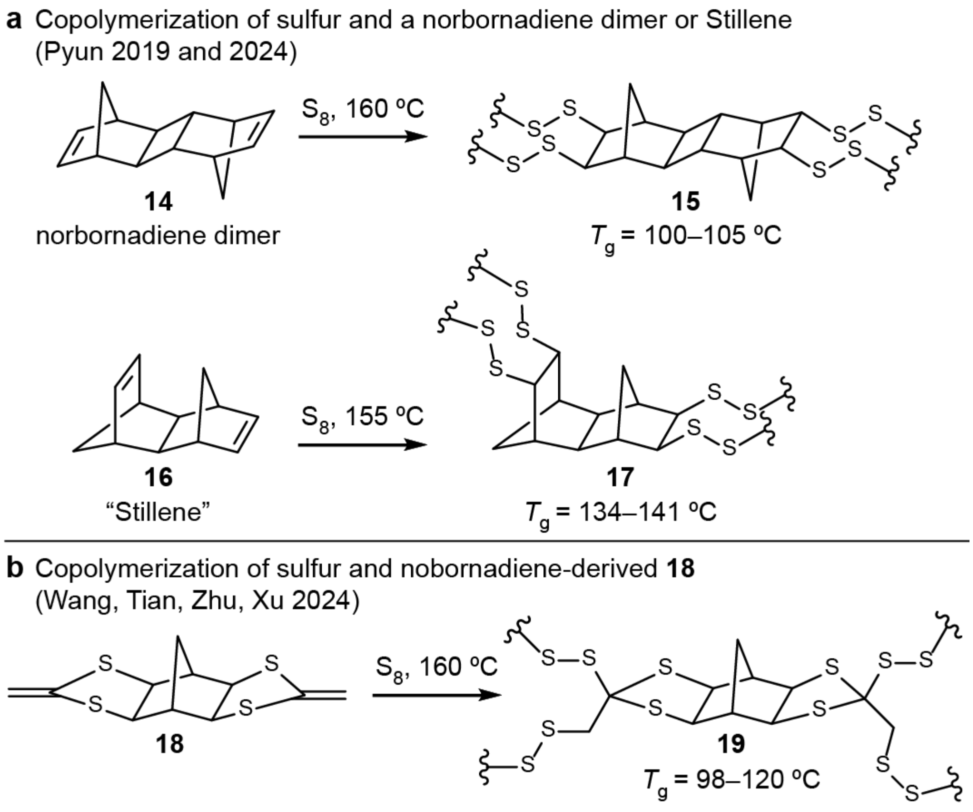

Supplementary Figure C | Norbornadiene-derived polymers from literature. a, Polymers **15** and **17** were developed by Pyun as synthetically accessible alternatives to **1**. These polymers have been validated in LWIR thermal imaging applications. Representative microstructures are shown. **b,** Norbornadiene-derived **18** can be copolymerized with sulfur to provide polymer **19**, which is useful in MWIR imaging. These polymers all have useful glass transition temperatures, but the LWIR transparency of **15**, **17** and **19** is still relatively low and makes LWIR imaging at ambient temperatures challenging (see Supplementary Figure D on the following page).

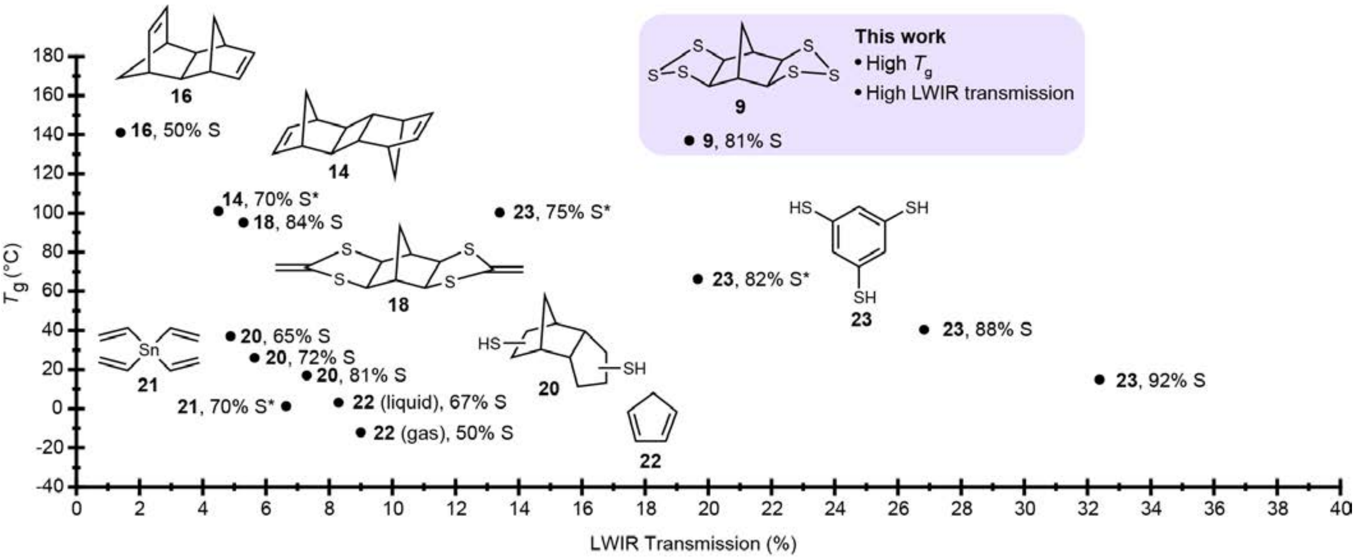

Supplementary Figure D | Comparison of average LWIR transmission (7–14 µm) and T_g for 9 and previously reported sulfur derived polymers. The organic monomer units are shown for simplicity, rather than the polymer formed after their copolymerization with sulfur. The average LWIR transmission is through a ~1 mm thick polymer window, or nearest available thickness reported. Asterisk (*) indicates where average LWIR transmission was estimated through image digitization of published infrared spectra, using the software package Origin 2021. T_g values were obtained by DSC; where unavailable, T_g values were obtained by DMA. Polymers that displayed instability—such as sulfur blooming—were omitted. If more than one polymer composition was reported for a given monomer, those with average LWIR transmission < 5% have been excluded. A detailed comparison of these and other reported polymers (with references) is provided in Supplementary Table S8 on page S122.

General Considerations

Abbreviations
DMF = *N*,*N*-dimethylformamide; NBD = Norbornadiene. RT = room temperature; min = minutes; h = hour or hours; eq. = equivalents; f = focal length.

Neat means the absence of solvent in the reaction.
Degassed means that liquids have been sparged with nitrogen prior to use or that the mixture has been placed under vacuum, as indicated.

Materials: All chemicals were purchased from commercial suppliers and used as received. Solvents were purchased from the commercial suppliers and used as received, unless otherwise stated. Deionized water was used for chemical reactions and work-up procedures. The xylenes were a mix of ortho, meta and para isomers in a 32:100:24 ratio, as analyzed by GC.

Solvent drying
Where dry solvents are used, reactions were conducted under an atmosphere of nitrogen with dry glassware. Solvents were dried for at least 48 hours prior to use if dried over molecular sieves. Reagents and solvents were degassed using nitrogen gas prior to use. Glass vials for chemical reaction were dried in an oven prior to use.

DMF was dried and distilled from CaH_2 and then stored over 4 Å molecular sieves. Anhydrous DMF was also purchased from Sigma Aldrich and used as received. Anhydrous DMF was also obtained from a solvent purification system (LC Technology Solution Inc.) and stored over 4 Å molecular sieves.

Analytical thin-layer chromatography (TLC) and column chromatography
TLC analysis employed commercial aluminum sheets coated with silica gel (Chem-supply, silica gel 60 F254). Compounds were either visualized under UV-light at 254 nm, or by dipping the plates in aqueous potassium permanganate or ceric ammonium molybdate solution followed by heating. Flash column chromatography was performed using either a glass chromatography column or a Biotage Selekt Flash Chromatography Instrument, with either silica gel (6 Å, 40 – 63 µm) or Biotage Sfär Silica (60 µm).

Nuclear magnetic resonance Spectroscopy (NMR)
^{1}H and ^{13}C NMR spectra were recorded on a Bruker Ultrashield Plus 600 MHz spectrometer at 600 MHz and 150 MHz respectively, or a Bruker Ascend 400 MHz spectrometer at 400 MHz and 100 MHz respectively. All spectra were obtained at 298 K unless stated otherwise. Deuterated solvents were used as solvent and internal lock unless stated otherwise. Residual solvent peaks were used as an internal reference for ^{1}H NMR spectra [$CDCl_3$ δ 7.26 ppm] and for ^{13}C NMR spectra [$CDCl_3$ δ 77.16 ppm]. The following abbreviations, or combinations thereof, were used to describe NMR multiplicities: s = singlet, d = doublet, t = triplet, q = quartet, m = multiplet, ap. = apparent, br. = broad).

Solid state nuclear magnetic resonance spectroscopy
The samples were ground into a powder and packed into a 4 mm rotor tube. A Bruker Ascend 400 MHz NMR spectrometer operating at 100 MHz was used with a magic angle spin rate of 5 kHz. The NMR was calibrated using the 29.5 ppm peak of adamantane. All samples were analyzed by ^{13}C cross polarization magic angle spinning solid state NMR at 298 K.

Gas chromatography - mass spectrometry (GC-MS)

GC-MS analysis was performed using an Agilent 5975C series GC-MS system. A 29.4 m x 250 µm x 0.25 µm, (5%-phenyl)-methylpolysiloxane column was used with a helium mobile phase. A 1 µL sample was injected with a split ratio of 60:1 and a gas flow rate of 1 mL/min.

The following GC-MS methods were used for experiments as indicated:
GC-MS method: Initial temperature 100 ºC. Hold at 100 ºC for 3 minutes. Ramp rate at 5 ºC/min to 200 ºC. Ramp rate at 20 ºC/min to 250 ºC. Hold at 250 ºC for 14.5 minutes. Total time of 40 minutes. The hold time at 250 °C was varied for some experiments but the rest of the method remained unchanged.

Fourier-transform infrared spectroscopy (FTIR)

Spectra were recorded between 4000 and 400 cm^{-1} using a Bruker Vertex 80v unless stated otherwise. All FTIR spectra were obtained under vacuum using a silicon carbide globar source, a potassium bromide (KBr) beam splitter and a deuterated triglycine sulfate (DTGS) detector. For ATR, the A225 Platinum ATR attachment with a diamond crystal was used. For reflectance spectroscopy, the A513 attachment was used with all samples referenced against an aluminum mirror. All spectra were integrated over 50 scans with a resolution of 2 cm^{-1}.

Elemental Analysis (CHNS)

Elemental analysis was performed by combustion analysis at the Chemical Analysis Facility at Macquarie University Analytical & Fabrication Facility using an Elementar vario MICRO (Elementar Analyzensysteme GmbH). The instrument hardware was configured for the analysis of 4 elements (C, H, N and S). In a typical procedure, 1-2 mg of sample material was loaded into a tin foil boat and combusted at 1150 °C with oxygen dosing time of 80 s and total O_2 flow rate of 30 mL/min. Ultra-high purity grade helium (BOC, 99.999%) and oxygen (BOC, 99.995%) were employed as working fluids in all cases. Pure sulfanilamide was employed as a standard with quality control samples performed every 15-20 runs. The follow-up data analysis was performed using a custom peak picking and integration algorithm written in Python 3.11.

Melting Point (m.p.)

Melting point of crystalline substances was determined using either a Gallenkamp or a DigiMelt 161 SRS (Stanford Research System) melting point apparatus using open ended capillary tubes and are uncorrected.

Raman spectroscopy

Raman spectra were acquired using a Witec alpha300R Raman microscope at an excitation laser wavelength of 785 nm with a 40X objective (numerical aperture 0.60). Integration times for single spectra were typically 20 seconds and averaged from 1 to 3 accumulations. The grating used was 600 g/mm which gives a spectral resolution of approximately 3 to 4 wavenumbers.

Differential Scanning Calorimetry (DSC)

Four differential scanning calorimeters were used. The method used in each experiment is described in the text. The four models of differential scanning calorimeters used are as follows:
Perkin Elmer DSC 8000
Mettler Toledo DSC
TA 2920 DSC
TA Q20 DSC

Thermogravimetric analysis (TGA)
TGA was carried out on a Perkin Elmer TGA800. The standard method used samples of 5-10 mg with a temperature range of 30-800 °C at a heating rate of 20 °C/min under a flow of nitrogen.

X-Ray diffraction
Single crystals were mounted in paratone-N oil on a nylon loop. X-ray diffraction data was collected at 100(2) K on the MX-1 or MX-2 beamline of the Australian Synchrotron (λ = 0.7107 Å)[1,2]. Structures were solved by direct methods using SHELX[3] and refined with SHELXL[4] and ShelXle[5] as a graphical user interface. All non-hydrogen atoms were refined anisotropically, and hydrogen atoms were included as invariants at geometrically estimated positions. Thermal ellipsoid plots were made using Mercury 2023.2.0 (build 382240).

Dynamic mechanical thermal analysis (DMTA)
DMTA analysis was performed using a TA DMA Q800. Standard temperature sweep method used a temperature range of -10 °C to 200 °C with a ramp rate of 3 °C/min. A preload force of 0.01 N was applied with and amplitude of 1 μm and a frequency of 1 Hz.

3D printing
Both FDM and DLP 3D printing was used. For all FDM printing, a Creality CR-10S Pro was used with standard PLA filament. All DLP printing was performed using a Phrozen Sonic Mini 8K using Aqua-Gray 8K resin.

Compound numbering

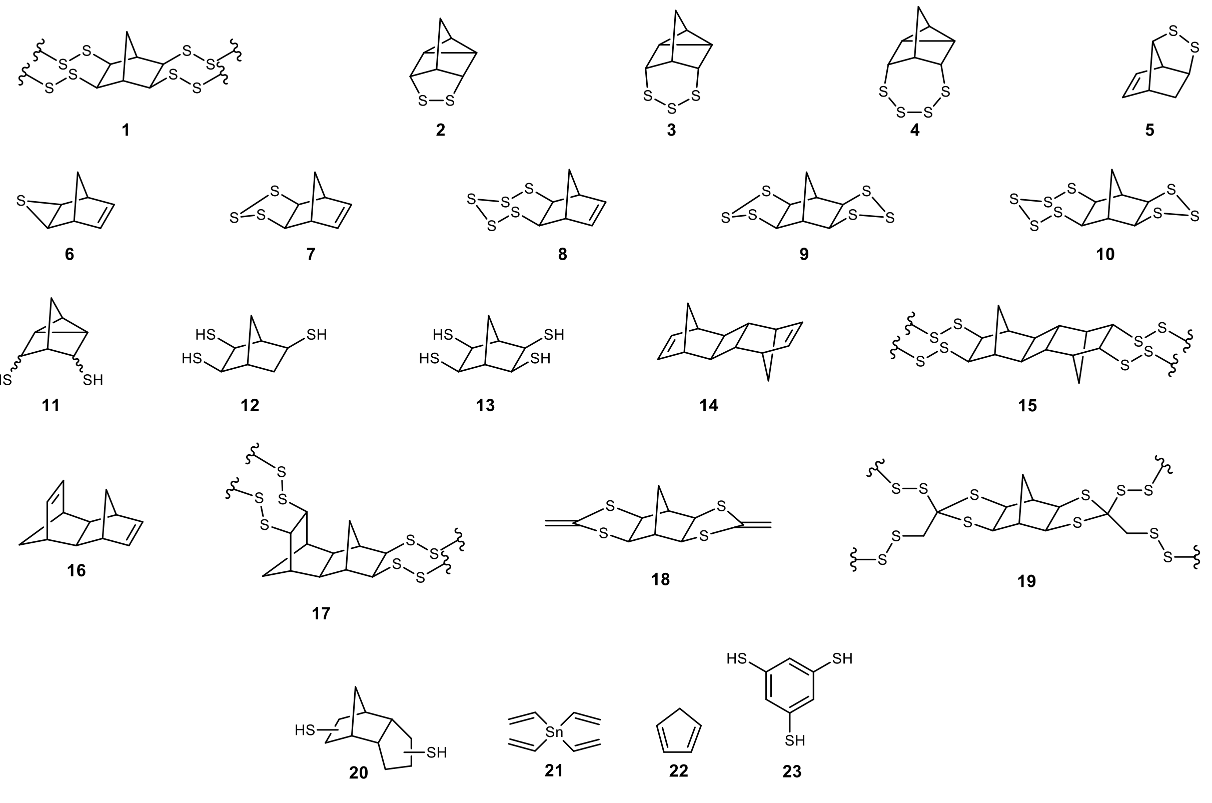

Synthesis and characterization of polymers prepared directly from norbornadiene

Solventless reaction between sulfur and norbornadiene

A direct inverse vulcanization reaction using sulfur as both a solvent and a monomer was first attempted. In this reaction, norbornadiene (NBD) was added directly to molten sulfur. Sulfur (3.00 g, 93.75 mmol S atoms) was added to a 21 mL vial with a magnetic stirrer. The vial was connected to a condenser and lowered into a 140 °C oil bath. The sulfur was heated with constant stirring for three minutes, over which time it melted into a yellow liquid. NBD (2.160 g, 23.44 mmol, 2.38 mL) was slowly added by volumetric pipette through the top of the condenser. The ratio of sulfur to NBD corresponded to a sulfur mass percentage of 58% and a theoretical sulfur rank of 2. Within 4-5 minutes of adding the NBD, the sulfur formed a soft yellow solid. NBD would still visibly reflux on the surface of the sulfur mixture but would no longer mix with the bulk material. Over time, the surface would slowly darken while the material underneath remained a yellow solid. After 90 minutes of heating, the material was removed from the vial and cured in a silicone container at 140 °C for 24 hours. This gave a material that ranged from yellow to black with visible sections of unreacted sulfur. While a reaction between sulfur and NBD did occur, it was not possible to control the reaction and ensure a homogeneous product. Images of the process are shown below.

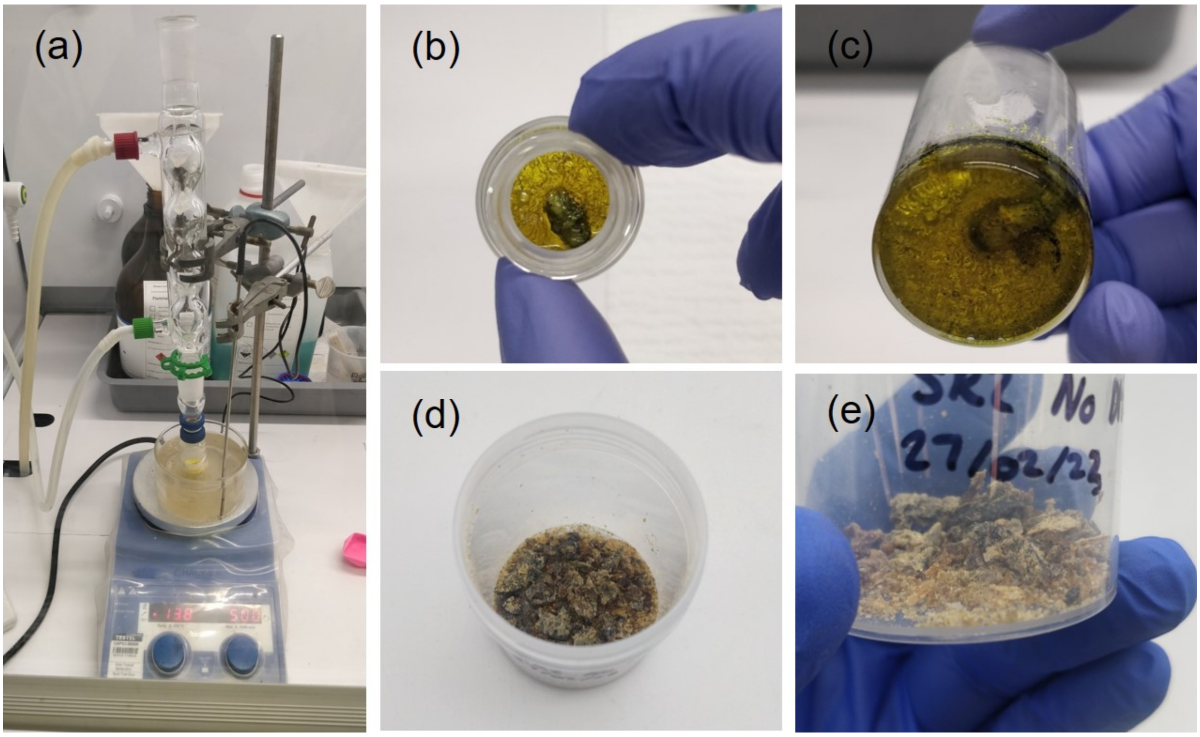

Figure S1: Images showing solventless reaction between sulfur and norbornadiene with 58% sulfur. (a) Reaction set up showing condenser and sulfur in vial. (b-c) images showing vitrified material after addition of norbornadiene. (d-e) Images showing inhomogeneous material from solventless reaction after curing for 24 hours at 140 °C.

Characterization of material made from solventless reaction

The material from the solventless reaction between sulfur and NBD was analyzed by differential scanning calorimetry (DSC), thermal gravimetric analysis (TGA) and elemental analysis. A Perkin Elmer DSC 8000 was used to analyze the material. Starting at room temperature, the material was cooled to -60 °C then heated to 100 °C at a heating rate of 10 °C/min. Following this, the material was cooled to -60 °C again, before being heated to 150 °C. A clear sulfur melting peak is observed around 115 °C, indicating the presence of crystalline sulfur. A TGA Perkin Elmer 8000 was used to analyze the material. Starting at room temperature, the material was heated to 800 °C at a heating rate of 20 °C/min under nitrogen atmosphere. Elemental analysis was performed by the chemical analysis facility at Macquarie University using a Vario Micro cube elemental analyzer. Both the surface where the NBD would reflux on the sulfur and the bottom were analyzed by elemental analysis. The surface had a lower sulfur content than expected with only 42 % while the bottom was over 95 % sulfur. This indicated that the NBD did not mix through the sulfur after it vitrified. The characterization of the reaction without solvents showed that a homogeneous polymer could not be formed. There was clear inhomogeneity caused by the premature vitrification and unreacted sulfur in the polymer network.

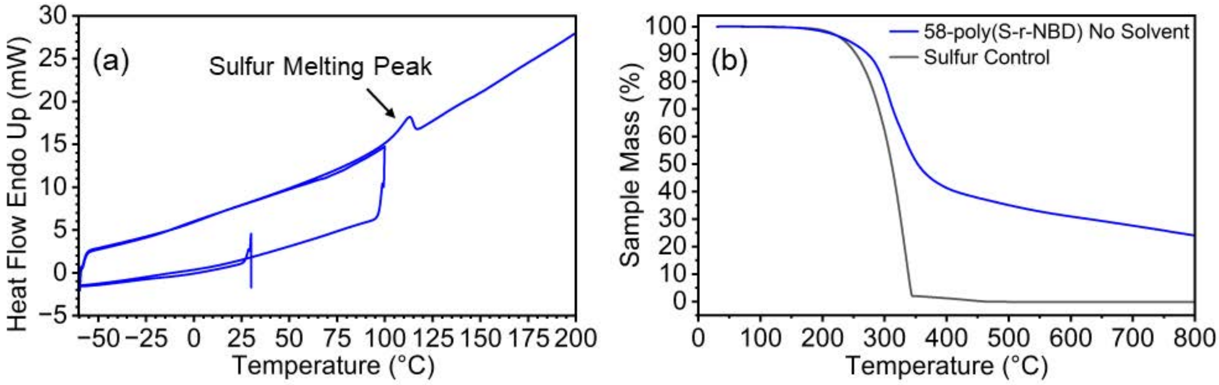

Figure S2: (a) DSC thermogram of the material made from the solventless reaction between sulfur and NBD showing sulfur melting peak. (b) TGA thermogram of the material made from the solventless reaction between sulfur and NBD.

Polymer synthesis in solvent

Polymers were prepared using 50-50 DMF-xylene as a solvent with sulfur contents ranging from 41% to 86%. Nine total polymer compositions were prepared with sulfur contents of 41%, 58%, 68%, 74%, 78%, 81%, 83%, 85% and 86%. These sulfur contents were selected so that the theoretical sulfur rank would range from 1 to 9. The exact sulfur rank was likely higher than this as some rearrangements were observed in this reaction, reducing the crosslinking of the polymer. Sulfur (1.00 g, 31.2 mmol S atoms) was added to a 21 mL vial with 5 mL of xylene and 5 mL of DMF. The vial was attached to a water-cooled reflux condenser and lowered into a 140 °C oil bath. The vial was heated for 10 minutes with constant stirring provided by a magnetic stirring bar. Over this time, the sulfur completely dissolved, giving a yellow solution. After heating for 10 minutes, NBD (volume varied for polymer composition) was added through the top of the reflux condenser using a volumetric pipette. The reaction was heated for an additional 80 minutes. The initially yellow solution slowly darkened over time, but no precipitate formed. The vial was then removed from the oil bath and xylene was evaporated using a rotary evaporator. The product was washed thoroughly in water before being lyophilized overnight. The product was transferred into a silicone mold and added to a 140 °C oven for 24 hours to cure. The polymer material was brittle and dark brown for all compositions apart from those with 85 % and 86 % sulfur. These were yellow in color and had the appearance of sulfur. The 41 % sulfur sample was extremely brittle and difficult to mold. These three samples were not used for future tests.

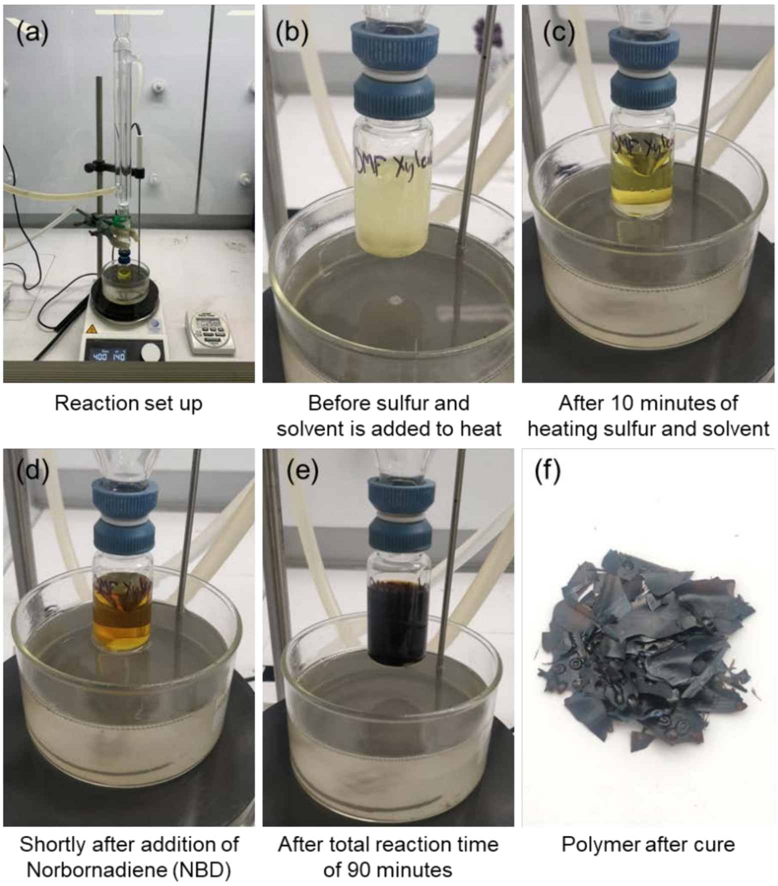

Figure S3: Reaction between sulfur and NBD using 50-50 DMF-xylene as the solvent. (a) Reaction set up. (b) Solution of sulfur in DMF-xylene before reaction. (c) Solution of sulfur in DMF-xylene after 10 minutes of heating at 140 °C. (d) Reaction mixture after addition of norbornadiene. (e) Reaction mixture after a total reaction time of 90 minutes. (f) Polymer material with 58% sulfur after removal of solvents and curing at 140 °C for 24 hours.

Reduction of polymers prepared in solvent

The 58% sulfur polymer made from NBD and sulfur in solution was reduced with lithium aluminum hydride ($LiAlH_4$) and analyzed by gas chromatography-mass spectroscopy (GC-MS) to gain an understanding of the chemical structure of the polymer. Accordingly, 30 mg of the polymer was ground into a powder and added to a flame dried vial and capped. The vial was purged with nitrogen. A 1 M solution of $LiAlH_4$ in anhydrous tetrahydrofuran (THF) was prepared and 5 mL was injected into the vial. The reaction was left for 24 hours with constant stirring. After this time, the reaction was cooled using an ice bath. 5 mL of 1 M hydrochloric acid (HCl) was injected slowly to quench the reaction. 5 mL of hexane was then injected into the mixture and stirred for an additional hour. The organic layer was separated and analyzed by GC-MS using an Agilent single quadrupole GC-MS. When reduced, the major product was a molecule with a cyclopropane formed from rearrangement. Both the dithiol and the disulfide rearranged product were observed in the mass spectrum. The peaks in the chromatogram were identified by synthesizing poly-**2** from independently synthesized monomer **2** and reducing the polymer with $LiAlH_4$. The gas chromatogram for that reduction is also shown below. Mass spectra for major peaks can be found on the following page.

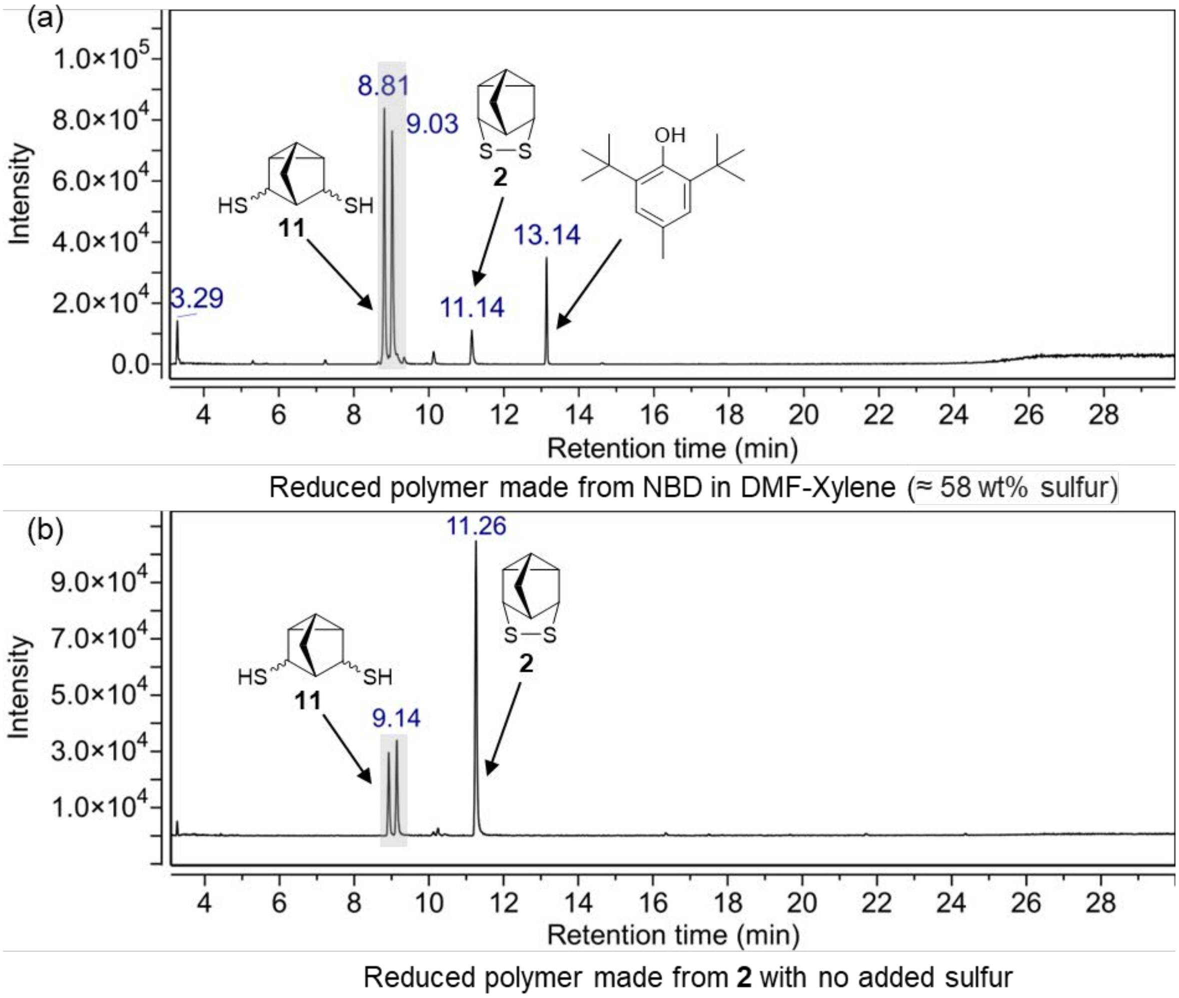

Figure S4: (a) Gas chromatogram of polymer prepared from NBD in a solvent system of DMF-xylene after reduction with $LiAlH_4$. (b) Gas chromatogram of poly-**2** after reduction with $LiAlH_4$.

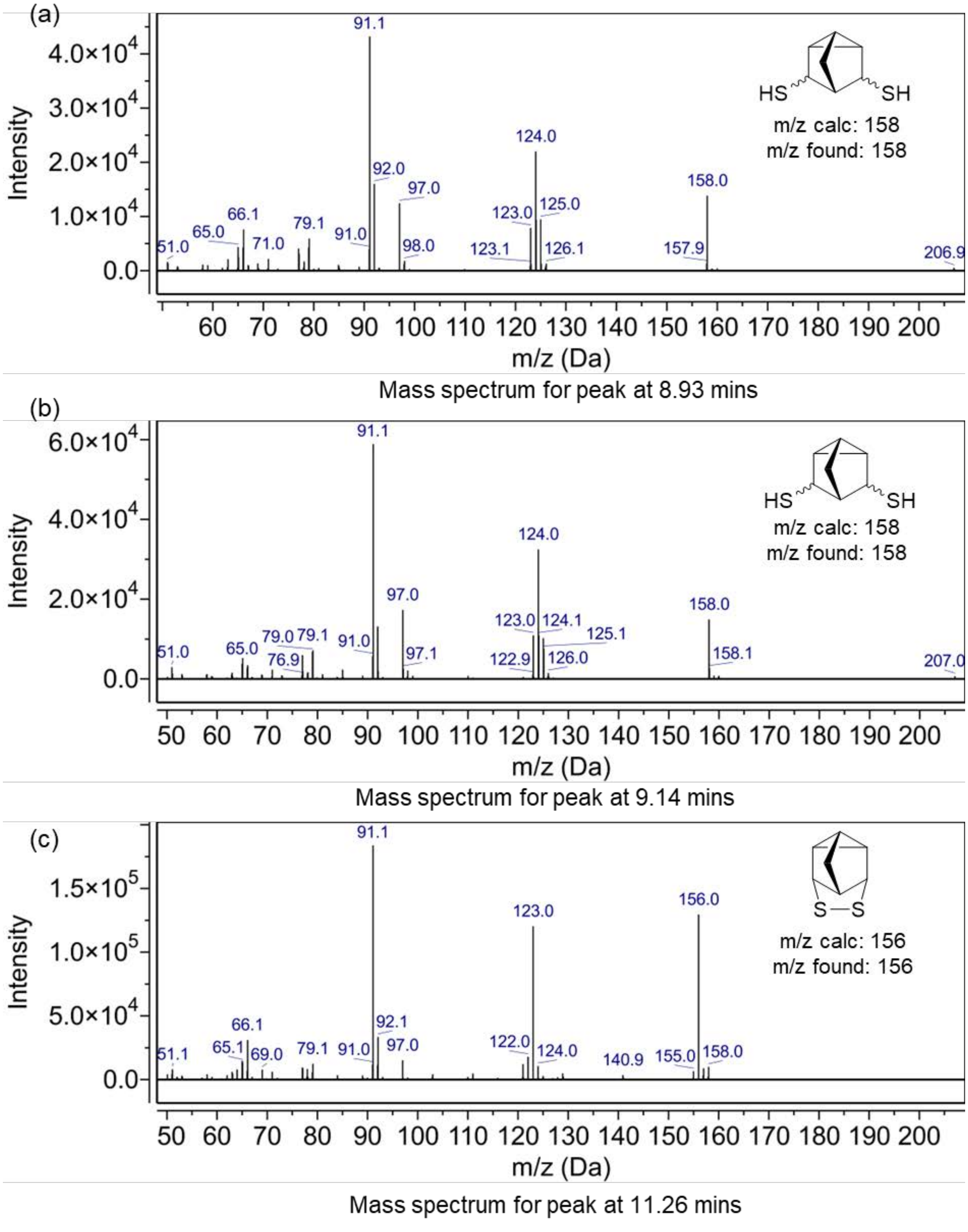

Figure S5: Mass spectra for major peaks in the reduced polymers prepared in solvent at retention times of (a) 8.93 minutes, (b) 9.14 minutes and (c) 11.26 minutes.

FTIR analysis of polymer prepared using solvent

The 58% sulfur polymer prepared using the reaction between sulfur and norbornadiene in a solvent system of DMF-xylene was analyzed by ATR FTIR. Clear peaks for the cyclopropane C-H were observed. This supported the results of the reduction experiments and indicated that rearrangement of the norbornadiene structure likely occurred in the polymerization process.

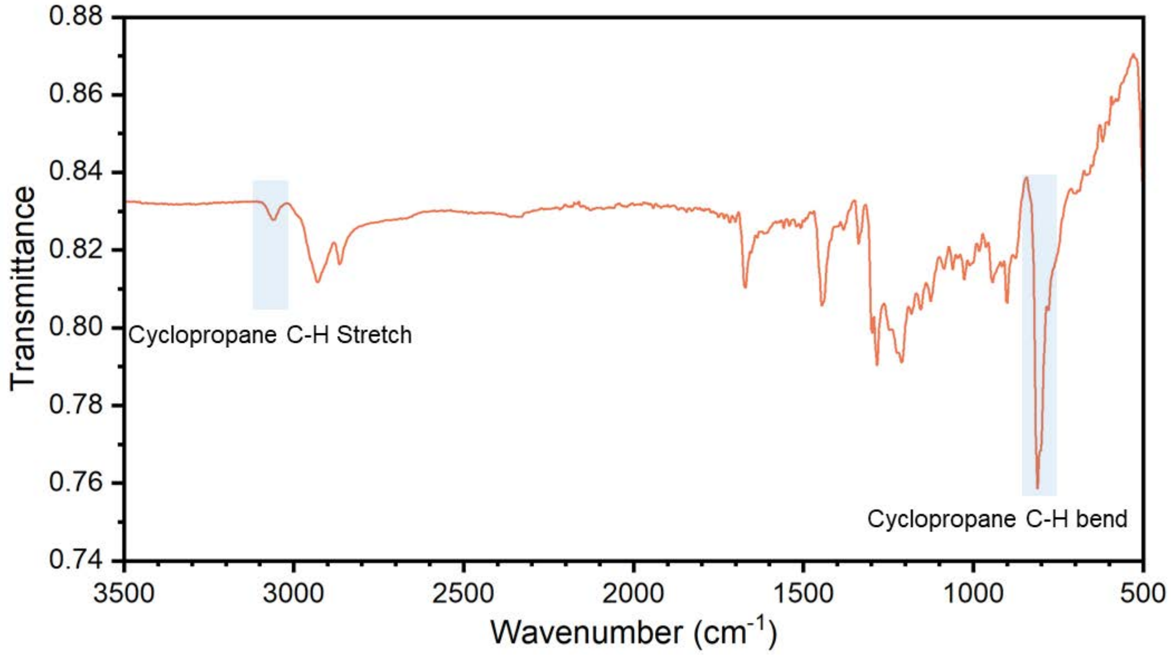

Figure S6: ATR-FTIR spectrum of polymer prepared from a reaction between sulfur and norbornadiene in a solvent system of DMF-xylene with a composition of 58 % sulfur. Peaks from the cyclopropane C-H are highlighted.

Glass transition temperature by DMTA of polymers prepared using solvent

The glass transition temperature of the polymers made in solvent was determined by two different methods: dynamic mechanical thermal analysis (DMTA) and differential scanning calorimetry (DSC). For DMTA, a dual cantilever clamp was used. Polymer samples were molded into rectangular prisms with dimensions of 65 mm by 5 mm by 2.5 mm. To make the mold, a negative was 3D printed with these dimensions. A silicone resin was poured into the 3D printed negative and left to cure for two hours. After this point, the silicone was removed from the negative to be used. The prepolymers would flow when heated above 60 °C if they had not been cured. This property was used to mold the prepolymers into the correct geometry for the dual cantilever clamp in the DMTA. The silicone mold was first heated to 70 °C. The prepolymer was then heated to 70 °C and poured into the mold. To reduce the chance of air bubbles forming in the prepolymer, it was poured into one end of the mold and allowed to flow to the other. Due to the high viscosity of the polymer, it was necessary to push it into the mold with a spatula. The mold and prepolymer were left in the oven at 70 °C for an additional 30 minutes to allow the prepolymer to take the shape of the mold. If needed, more prepolymer would be added to ensure the sample was consistent in height. The mold and prepolymer were then added to an oven at 140 °C for 24 hours to cure. This was done for the samples ranging from 58% sulfur to 83% sulfur. The 41% sulfur prepolymer did not flow when heated and could not be molded. To test the glass transition temperature, a multifrequency temperature sweep was used on the DMTA with a dual cantilever clamp. The polymer sample was added into the clamp and cooled to −10 °C. After equilibrating, it was heated at 3 °C/min until 150 °C. The glass transition was determined from the temperature of the center of the tan delta peak. The glass transition temperature decreased with increasing sulfur content.

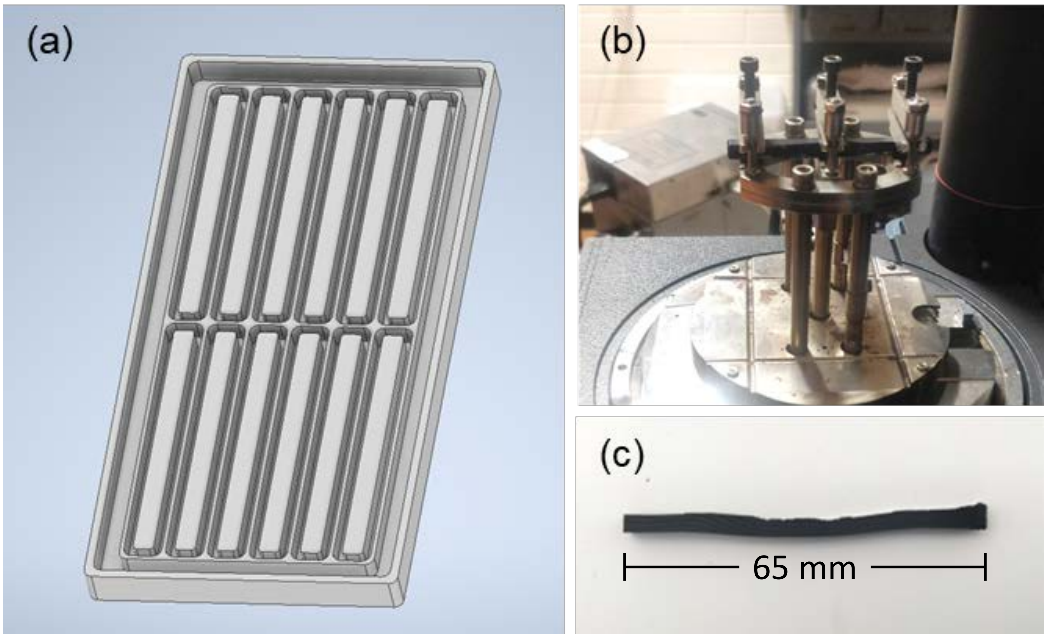

Figure S7: Images showing the testing of sulfur polymers by dynamic mechanical thermal analysis. (a) Image of negative that was 3D printed to make silicone mold. (b) Polymer sample in dual cantilever clamp of TA Q800 dynamic mechanical analyzer. (c) sample after multifrequency temperature sweep

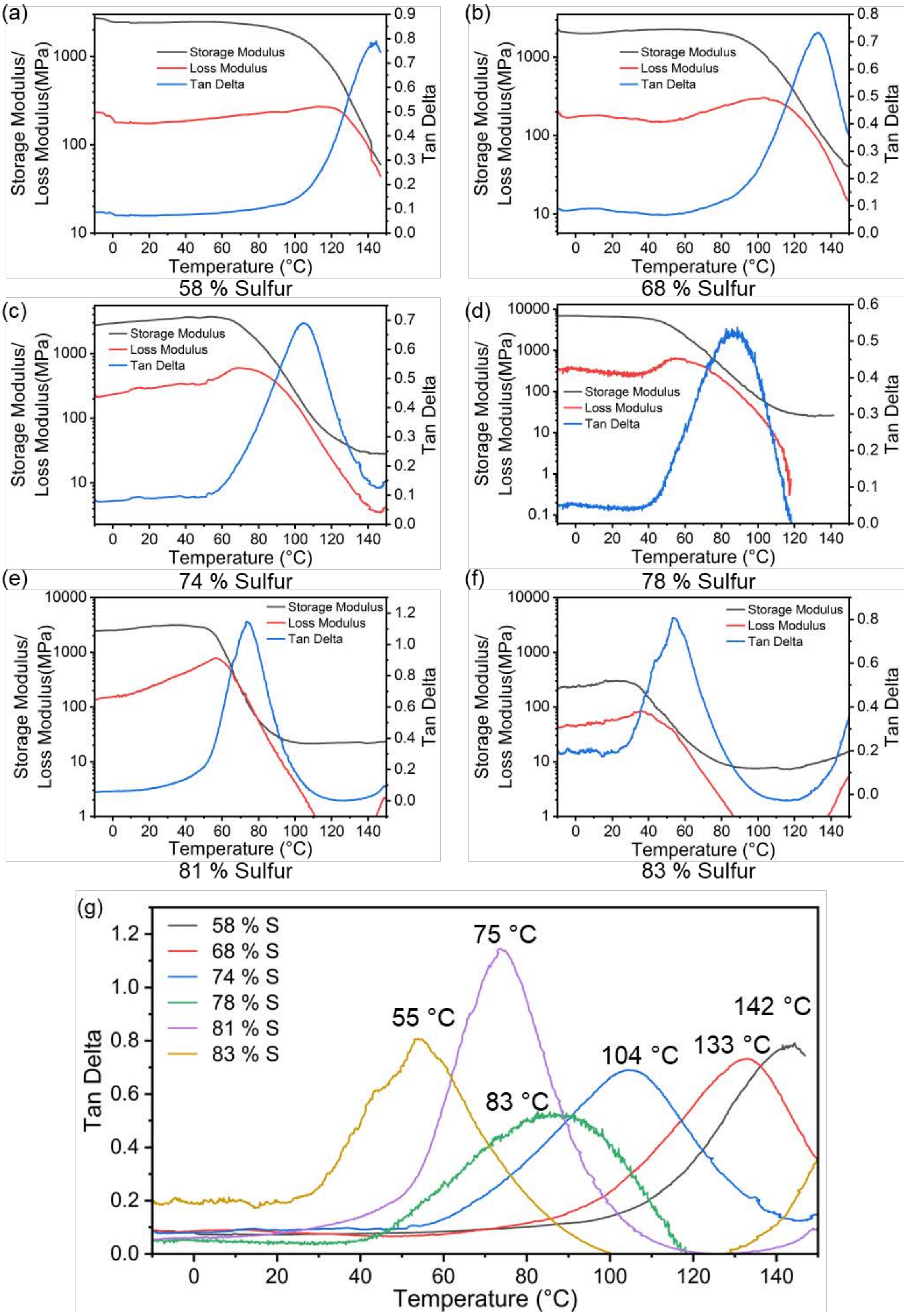

Figure S8: (a-f) DMTA thermograms for polymers of sulfur and NBD that were made in a solvent system of 50-50 DMF-xylene. A cantilever bending clamp was used with a multifrequency temperature sweep from −10 °C to 150 °C. The sulfur content of the polymers ranged from 58 % to 83 %. (g) Tan delta for DMTA thermogram of each polymer sample.

Glass transition temperature by DSC of polymers prepared using solvent

To confirm the results of the DMTA experiments, a method was developed to cure the prepolymer in the DSC. In this method, the uncured prepolymer was placed into a Perkin Elmer DSC 8000. The samples were first heated to 100 °C for 1 minute to remove any thermal history. Following this, the temperature was decreased to -60 °C at 25 °C/min and held for 1 minute before increasing to 140 °C. This step allowed for the initial glass transition temperature to be determined. After this, the temperature was held at 140 °C for an hour to cure the prepolymer before another temperature sweep to -60 °C. The process of curing for an hour at 140 °C before a temperature sweep was repeated for 24 hours. This allowed for the measurement of the glass transition temperature over the full cure of the polymer. As the sulfur in the sample increased, the time to reach full cure and the final glass transition temperature decreased. The only sample that showed a crystalline sulfur melting peak was the 83 % sulfur sample. This indicated that there is unreacted sulfur in the polymer, and this is therefore an upper limit to the sulfur content of the polymer. The glass transition temperature was lower than the values obtained by DMTA but followed the same trends.

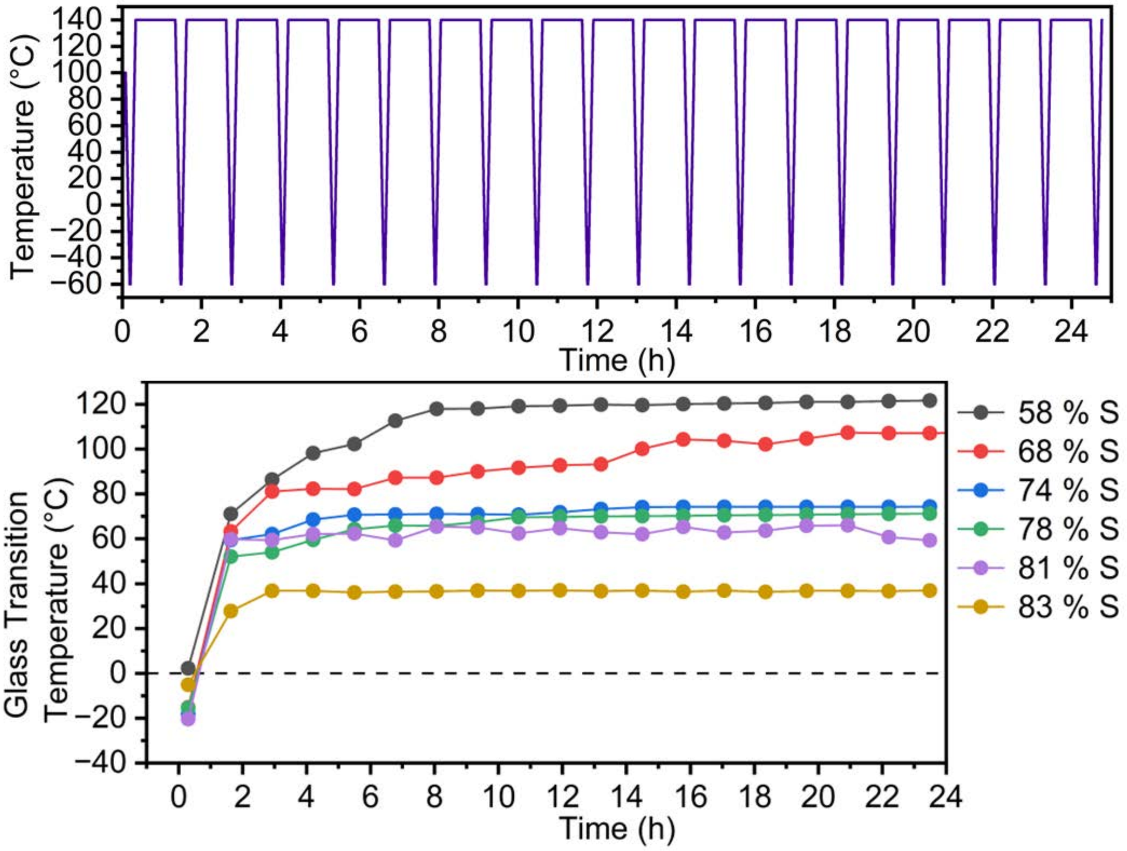

Figure S9: Temperature profile of DSC method and glass transition temperature against cure time in DSC for polymers made from sulfur and norbornadiene in different ratios. All polymers were made in a solvent system of 50-50 DMF-xylene.

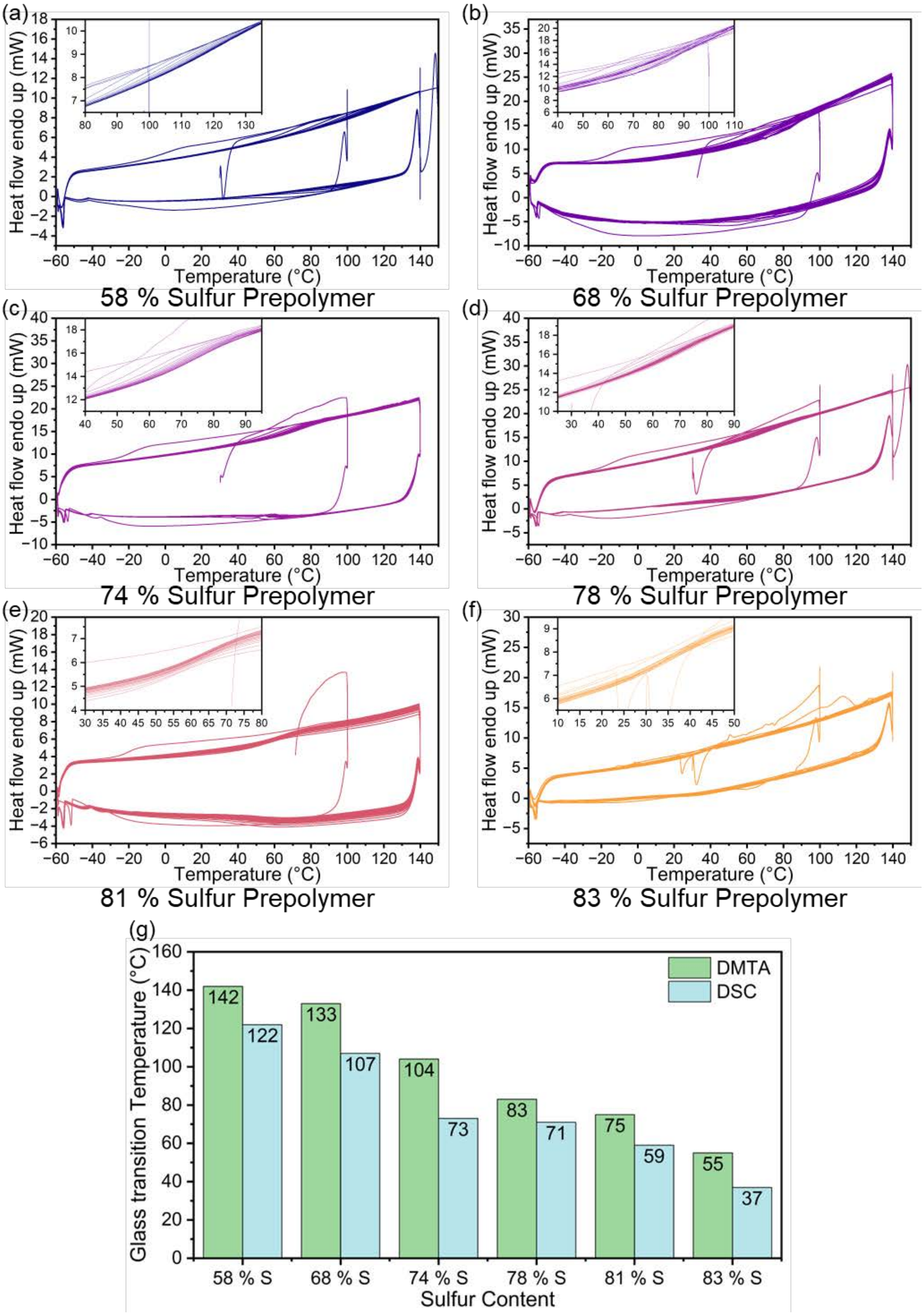

Figure S10: (a-f) DSC thermograms for prepolymers of sulfur and NBD that were made in a solvent system of 50-50 DMF-xylene. The sulfur content of the prepolymers ranged from 58 % to 83 %. (g) Plot showing the glass transition temperature obtained by DMTA and DSC.

Infrared transparency of polymers prepared in solvent

To prepare the polymer windows, a custom compression die was machined by the engineering services at Flinders University. This die was necessary to control the thickness and geometry of the polymer windows prepared by compression molding so they could be tested for infrared transparency. The die was designed on Autodesk Inventor and machined from aluminum. There were several parts to the die, including a push rod, an outer sleeve, two polished disks and several thickness determining rings. When assembled, the sample could be put between two discs which fit into the outer sleeve. A ring could be selected to vary how far the push rod could extend into the sleeve, altering the thickness of the polymer window. Each of the polymers made from norbornadiene and sulfur in DMF-xylene were compressed into windows of three thicknesses using the following method: The die was preheated to 140 °C and the cured polymer was crushed into a powder. Approximately 500 mg of polymer was loaded into the aluminum die and added to a heated press. 20 MPa of pressure was applied for 10 minutes. The aluminum die was then removed from the press and left to cool to approximately 40 °C before the window was extracted. Each polymer composition was compressed into windows with three thickness: 0.75 mm, 1.0 mm, and 1.1 mm. Each polymer window was tested for transmission using a Perkin Elmer Frontier FTIR spectrometer. The transmittance was integrated over the mid- (3 µm – 5 µm) and the long- (7 µm – 14 µm) wave infrared regions then divided by the wavelength range to obtain an average transmittance.

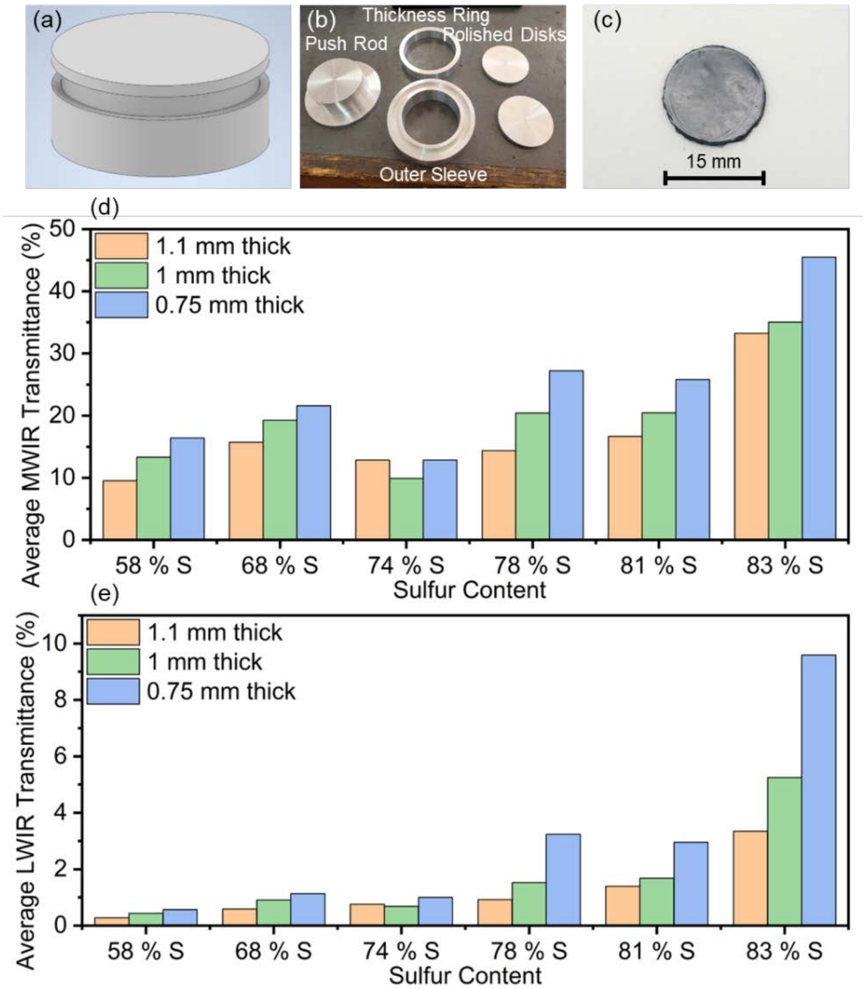

Figure S11: (a-b) Autodesk Inventor design and photo of compression mold die. (c) Photo of polymer window after compression. (d) Average MWIR (3 µm – 5 µm) transmission of polymer windows with varying thickness and sulfur content. (e) Average LWIR (7 µm – 14 µm) transmission of polymer windows with varying thickness and sulfur content.

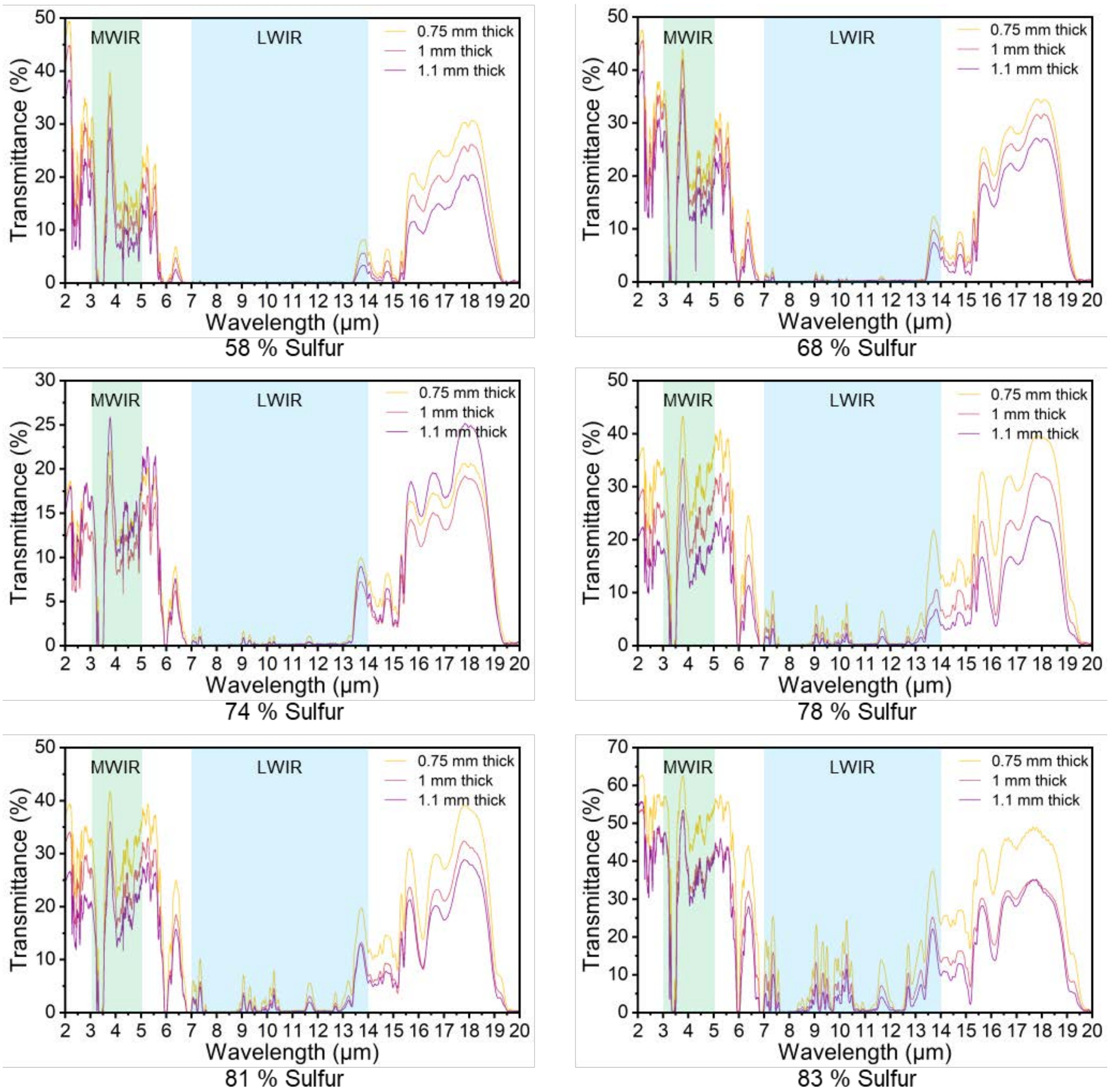

Figure S12: FTIR spectra measured on a Perkin Elmer Frontier FTIR spectrometer for polymers made from NBD and sulfur in a solvent system of 50-50 DMF-xylene. Several compositions are shown with sulfur contents ranging from 58 % to 83 %. For each composition, three window thicknesses were tested: 0.75 mm, 1 mm, and 1.1 mm.

Refractive index of polymers prepared in solvent

Each of the polymers were tested for refractive index using their specular reflectance using a Bruker Vertex v80 instrument with the 1513/QA attachment. The 1 mm thick window of each polymer composition was tested with an angle from the surface normal of 15 ° under vacuum. All samples were referenced using the reflectance spectrum of an aluminum mirror. A Kramers-Kronig transform was applied using the OPUS V7.2 software to find the wavenumber dependent phase shift spectrum. The refractive index was then calculated using the real portion of the complex refractive index, $\eta = n + ik$. As the sulfur content of the samples increased, the reflectance and refractive index increased. This would be expected as the sulfur atom is large and polarizable when compared to carbon[6], leading to an increase in refractive index.

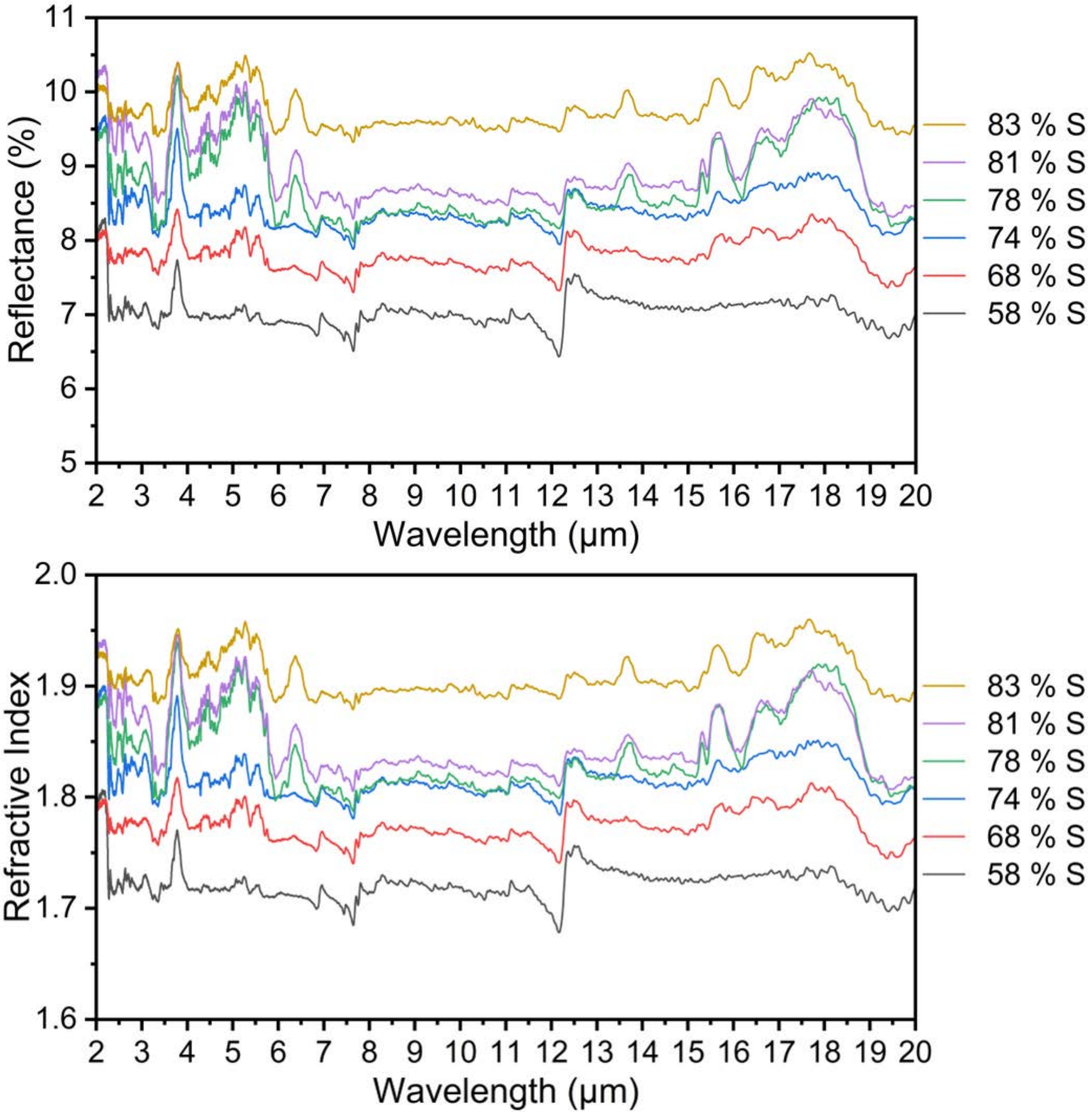

Figure S13: Reflectance and refractive index spectra of polymers made from sulfur and norbornadiene in a solvent system of 50-50 DMF-xylene. All spectra were obtained on a Bruker Vertex v80 using the 1513/QA attachment. An angle from the surface normal of 15 ° was used with all spectra reference against an aluminum mirror. The refractive index was determined by first taking the Kramers-Kronig transform of the reflectance spectra of each sample then taking the real portion of the complex refractive index, $\eta = n + ik$.

Synthesis and isolation of norbornadiene derived cyclic sulfides

Extraction of hexane soluble intermediates and analysis with GC-MS

To gain a greater understanding of the reaction between norbornadiene and sulfur, a procedure was developed to isolate and characterize the intermediates of the reaction. Accordingly, elemental sulfur (5.00 g, 156 mmol S atoms) was added to a 250 mL two necked round bottom flask along with a magnetic stirrer, DMF (50 mL) and xylene (50 mL). A condenser was added to the main neck of the flask and the other was sealed with a rubber septum. The reaction was then heated at 140 °C for 30 minutes with constant stirring. Over this time, the sulfur completely dissolved in the solvents to form a deep orange liquid. Norbornadiene (3.98 mL, 39.13 mmol) was added using a syringe through the rubber septum. Immediately after the addition of NBD, a 1 mL aliquot of the reaction mixture was removed using a needle through the rubber septum and added to a glass vial. This was the 30-minute sample, indicating the reaction time for sulfur. Aliquots were then taken every 15 minutes over 120 minutes and then again at 24 hours. The aliquots were left to cool before deionized water (2 mL) and hexane (2 mL) were added. In this mixture, polymeric material precipitated in the water/DMF (bottom) layer. A 100 µL aliquot of the hexane/xylene layer (top) was filtered through cotton wool and added to a GC-MS vial. The aliquot was diluted to 1 mL with hexane before being filtered and analyzed on a Perkin Elmer Agilent GC-MS. It should be noted that molecules **8, 9** and **10** would decompose under the GC-MS conditions and could not be identified. These molecules were later confirmed by NMR spectroscopic analysis.

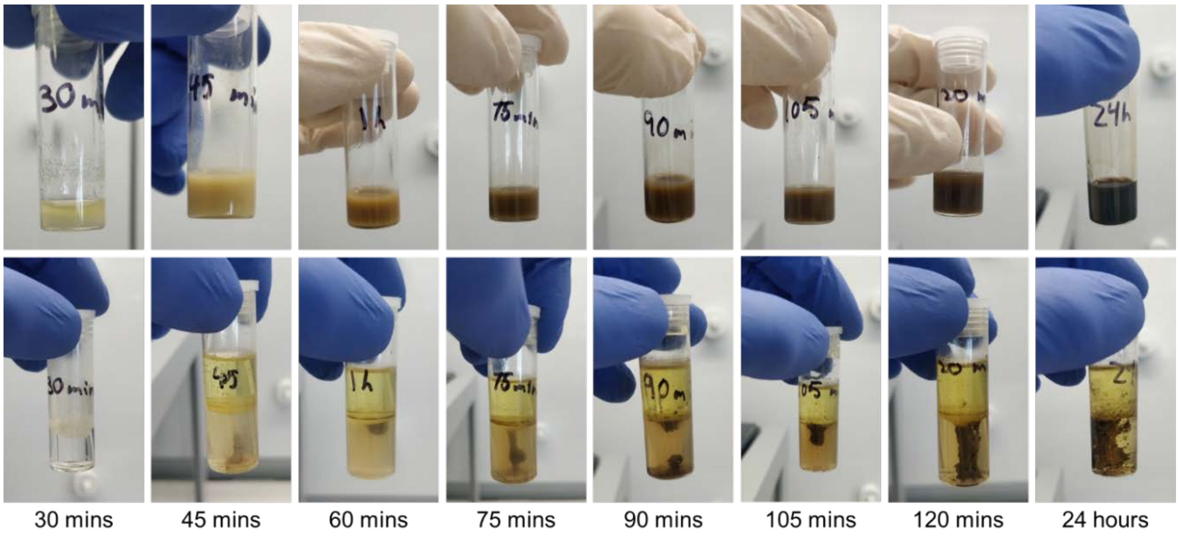

Figure S14: Aliquots taken from reaction between sulfur and NBD in DMF-xylene. The reaction was left for a total of 24 hours with aliquots removed every 15 minutes for the first 120 minutes then another at 24 hours. The top row shows the aliquots directly after being removed from the reaction. The bottom row is after 2 mL of deionized water and 2 mL of hexane was added. Polymeric material precipitated in the bottom layer and hexane-soluble intermediates were extracted into the top layer for further analysis by GC-MS.

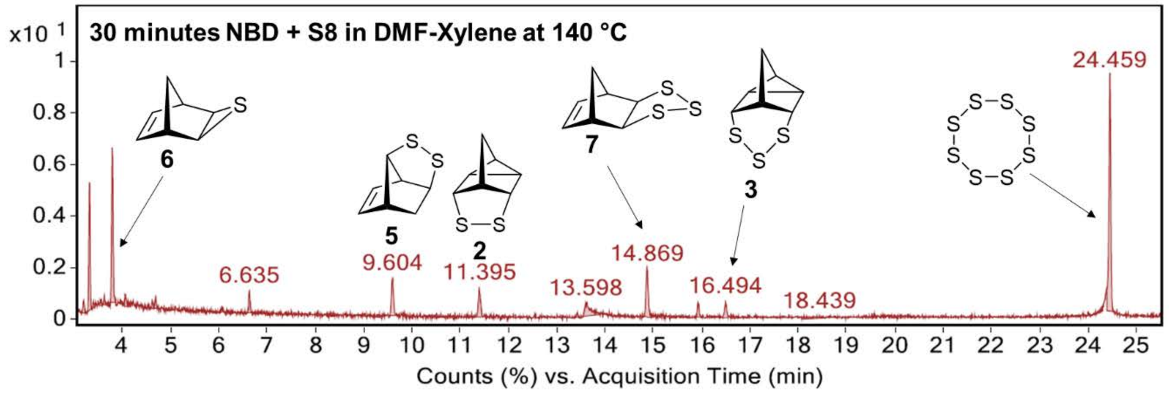
30 minutes NBD + S8 in DMF-Xylene at 140 °C
x10 1
6
5
2
7
3
6.635
9.604
11.395
13.598
14.869
16.494
18.439
24.459
Counts (%) vs. Acquisition Time (min)

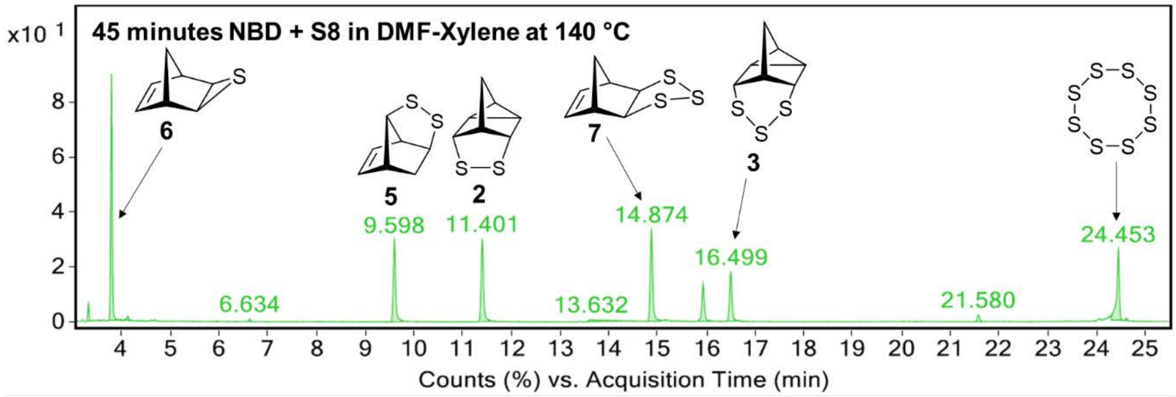
45 minutes NBD + S8 in DMF-Xylene at 140 °C
x10 1
6
5
2
7
3
6.634
9.598
11.401
13.632
14.874
16.499
21.580
24.453
Counts (%) vs. Acquisition Time (min)

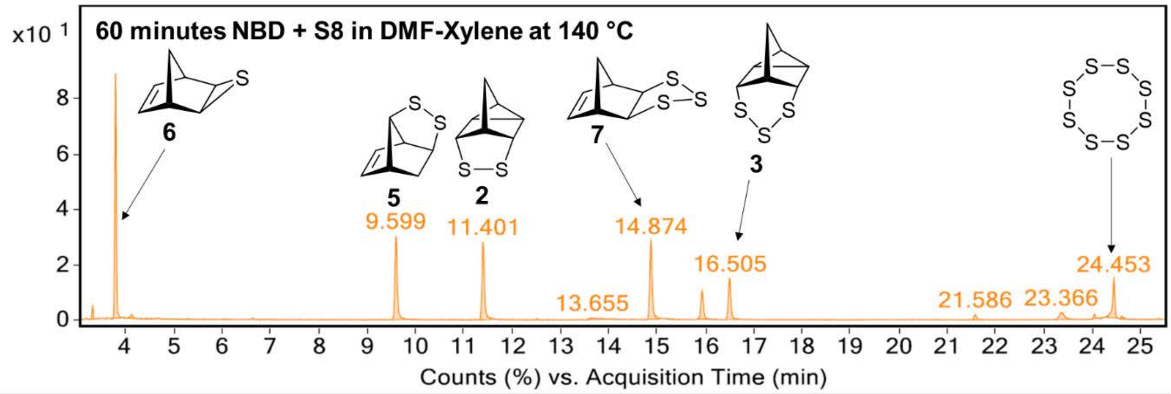
60 minutes NBD + S8 in DMF-Xylene at 140 °C
x10 1
6
5
2
7
3
9.599
11.401
13.655
14.874
16.505
21.586
23.366
24.453
Counts (%) vs. Acquisition Time (min)

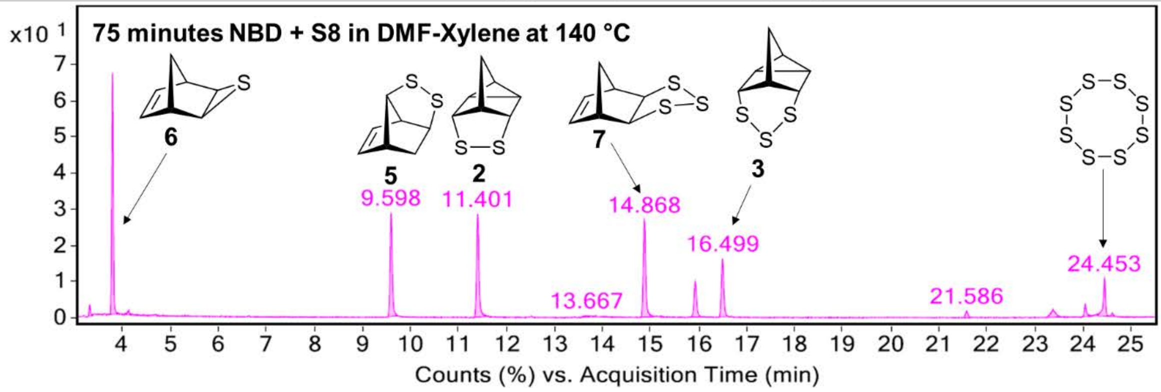
75 minutes NBD + S8 in DMF-Xylene at 140 °C
x10 1
6
5
2
7
3
9.598
11.401
13.667
14.868
16.499
21.586
24.453
Counts (%) vs. Acquisition Time (min)

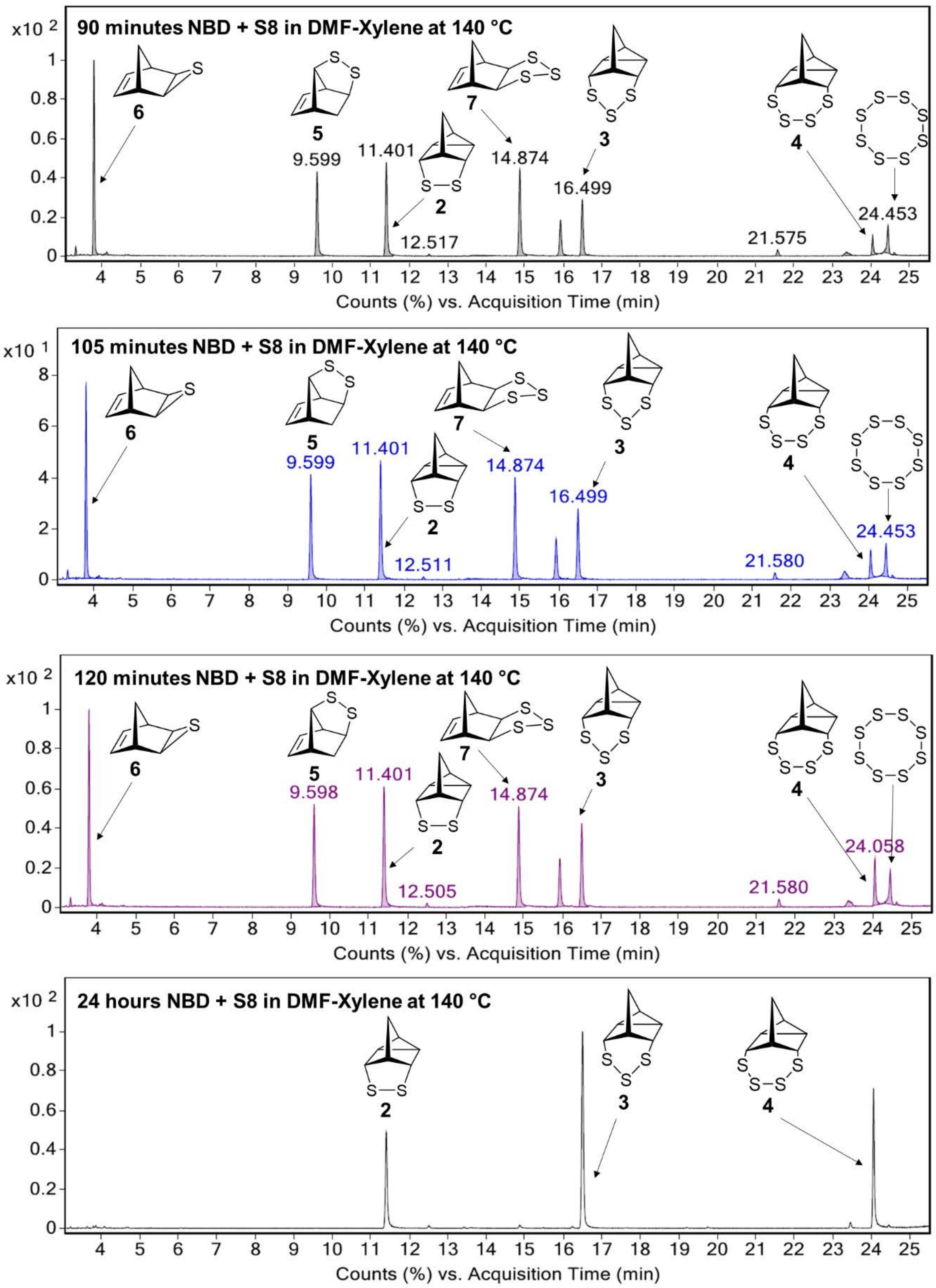

Figure S15: Gas chromatograms for extracted products of the reaction between sulfur and NBD in a solvent system of DMF-xylene.

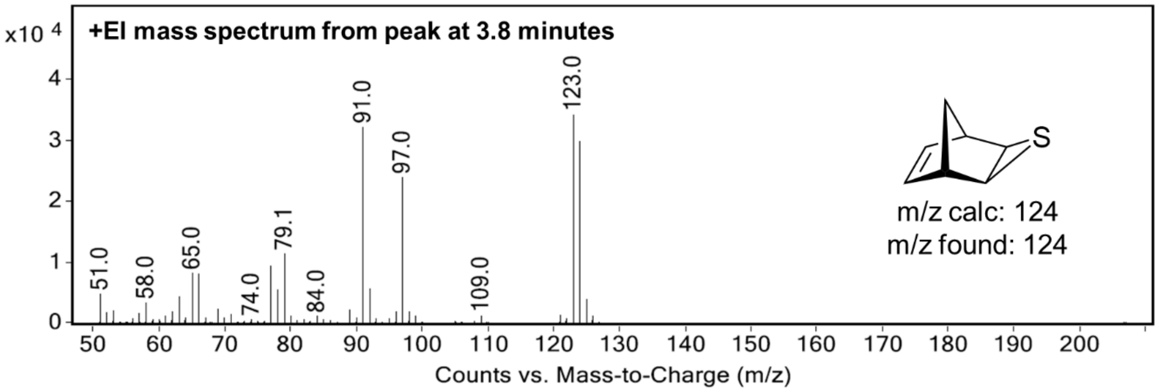
x10 4
+EI mass spectrum from peak at 3.8 minutes
51.0
58.0
65.0
74.0
79.1
84.0
91.0
97.0
109.0
123.0
S
m/z calc: 124
m/z found: 124
Counts vs. Mass-to-Charge (m/z)

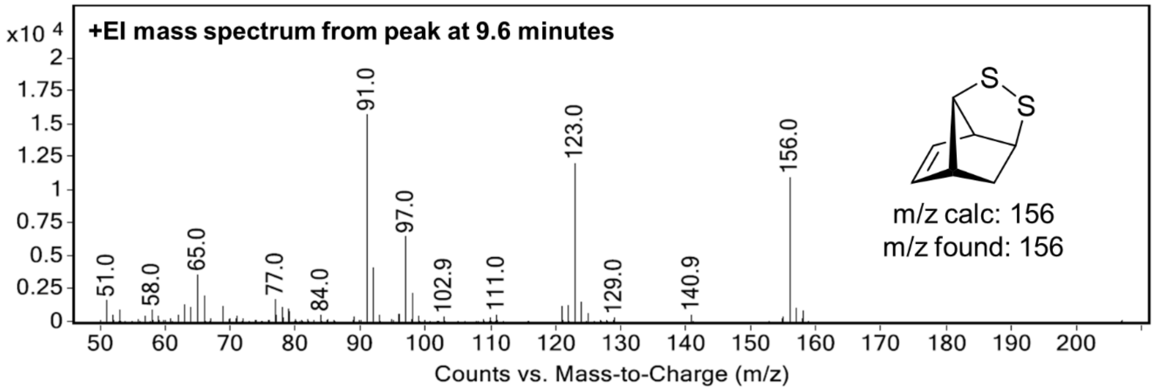
x10 4
+EI mass spectrum from peak at 9.6 minutes
51.0
58.0
65.0
77.0
84.0
91.0
97.0
102.9
111.0
123.0
129.0
140.9
156.0
S
S
m/z calc: 156
m/z found: 156
Counts vs. Mass-to-Charge (m/z)

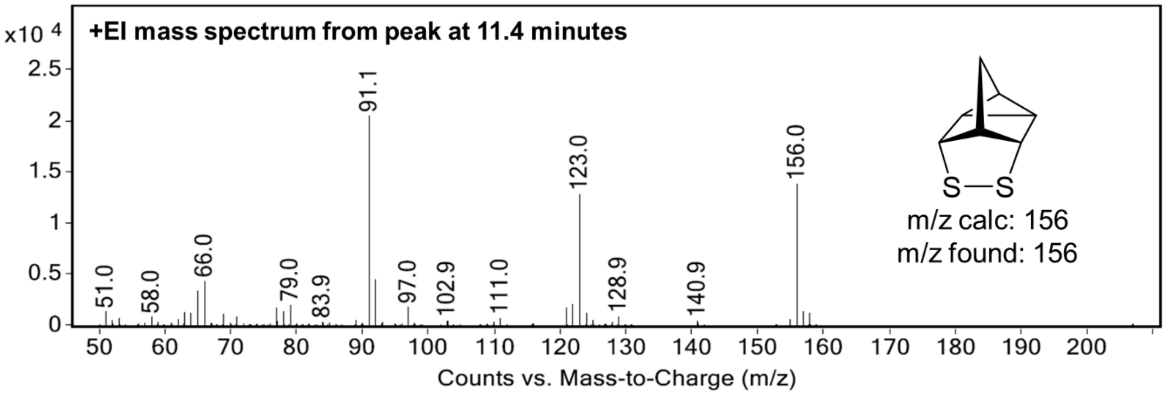
x10 4
+EI mass spectrum from peak at 11.4 minutes
51.0
58.0
66.0
79.0
83.9
91.1
97.0
102.9
111.0
123.0
128.9
140.9
156.0
S—S
m/z calc: 156
m/z found: 156
Counts vs. Mass-to-Charge (m/z)

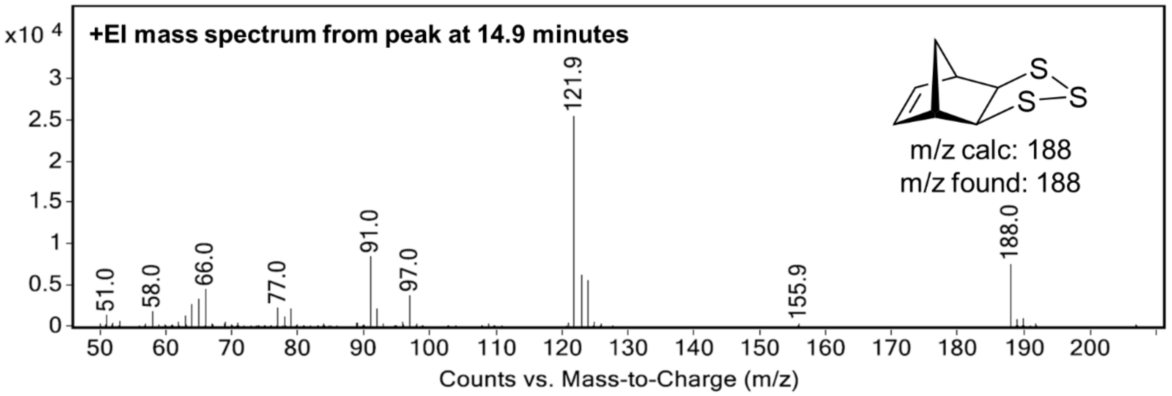
x10 4
+EI mass spectrum from peak at 14.9 minutes
51.0
58.0
66.0
77.0
91.0
97.0
121.9
155.9
188.0
S
S
S
m/z calc: 188
m/z found: 188
Counts vs. Mass-to-Charge (m/z)

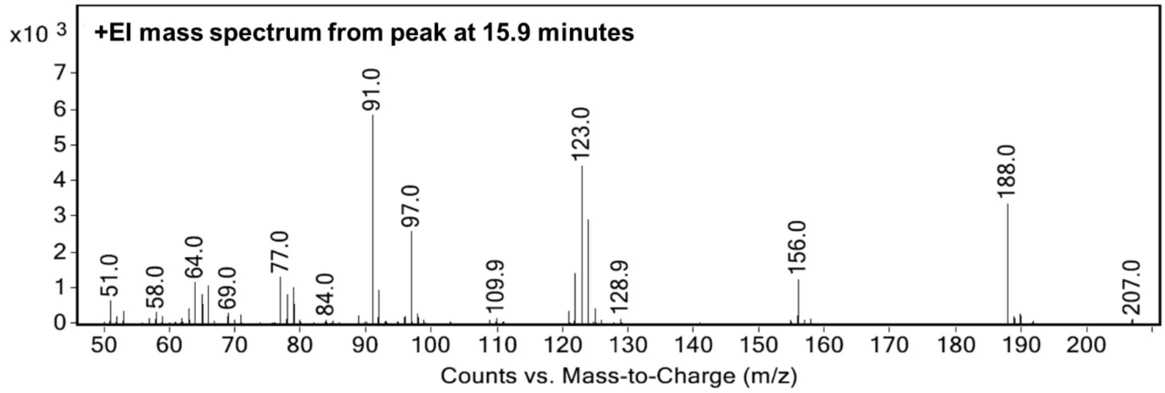
x10 3
+EI mass spectrum from peak at 15.9 minutes
51.0
58.0
64.0
69.0
77.0
84.0
91.0
97.0
109.9
123.0
128.9
156.0
188.0
207.0
Counts vs. Mass-to-Charge (m/z)

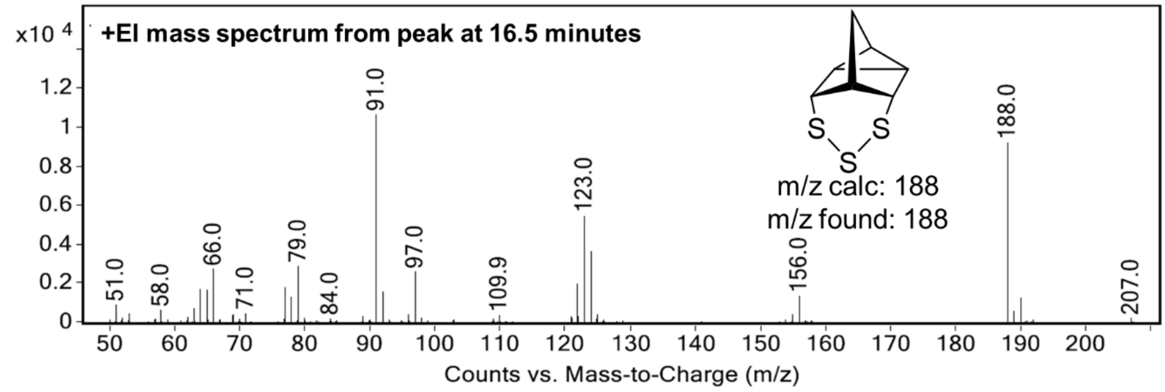
x10 4
+EI mass spectrum from peak at 16.5 minutes
S
S
S
m/z calc: 188
m/z found: 188
51.0
58.0
66.0
71.0
79.0
84.0
91.0
97.0
109.9
123.0
156.0
188.0
207.0
Counts vs. Mass-to-Charge (m/z)

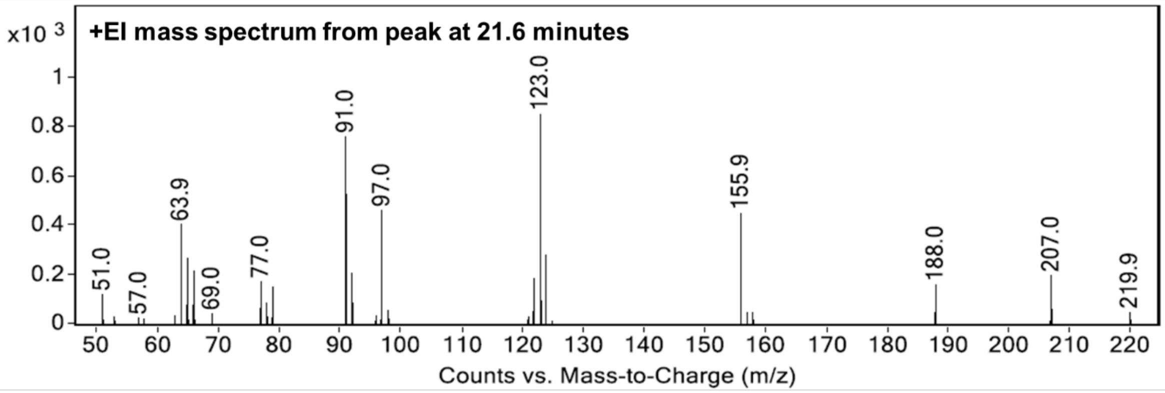
x10 3
+EI mass spectrum from peak at 21.6 minutes
51.0
57.0
63.9
69.0
77.0
91.0
97.0
123.0
155.9
188.0
207.0
219.9
Counts vs. Mass-to-Charge (m/z)

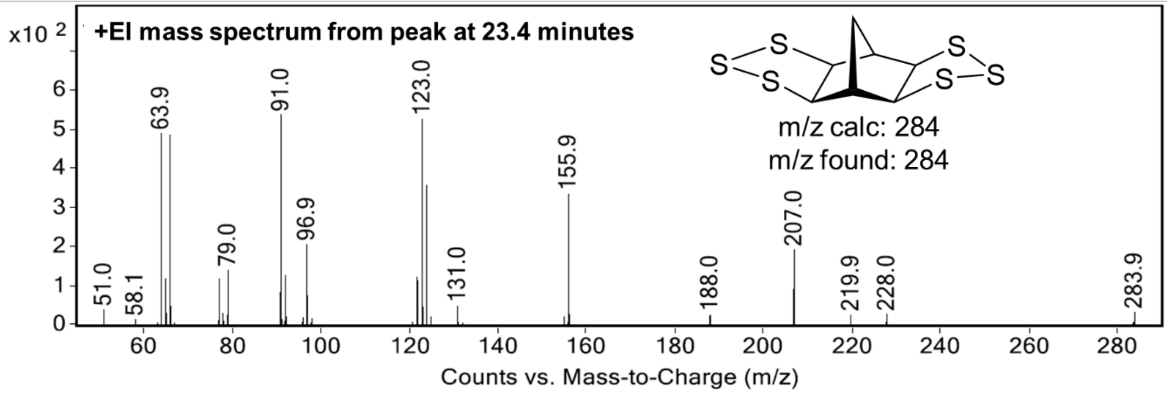
x10 2
+EI mass spectrum from peak at 23.4 minutes
S
S
S
S
S
S
m/z calc: 284
m/z found: 284
51.0
58.1
63.9
79.0
91.0
96.9
123.0
131.0
155.9
188.0
207.0
219.9
228.0
283.9
Counts vs. Mass-to-Charge (m/z)

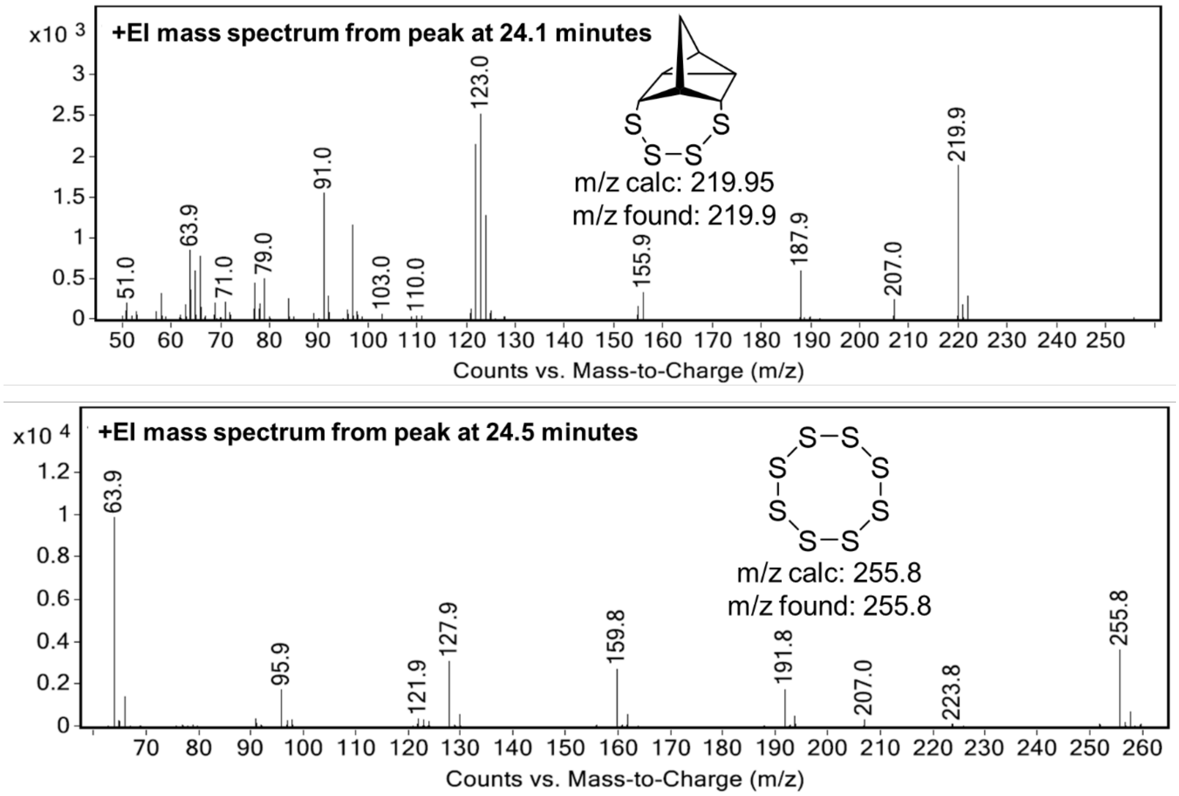

Figure S16: Mass spectra for the peaks identified in the GC-MS analysis of the reaction between solution-phase reaction between norbornadiene and sulfur.

Method used to synthesize cyclic sulfide intermediates

A method was prepared to optimize the formation of the cyclic sulfide intermediates so they could be isolated and characterized fully. The optimized method was slightly different to the method used in the polymerization as it used a lower temperature (120 °C instead of 140 °C), a $[Ni(NH_3)_6]Cl_2$ catalyst, and 50-50 toluene-DMF instead of 50-50 xylene-DMF. The lower temperature resulted in greatly reduced polymer and more soluble intermediates. Toluene was used instead of xylene as it has a lower boiling point which aided solvent removal during workup. The reaction was also run for 90 minutes so that the intermediates did not react completely or form polymers. After this point, a workup was used to separate the cyclic intermediates from any polymeric material. The specific method used to synthesize the cyclic sulfide intermediates is as follows: A 250 mL round bottomed flask was charged with sulfur (4.725 g, 147.4 mmol S atoms), norbornadiene (5.00 mL, 4.53 g, 49.15 mmol) and $[Ni(NH_3)_6]Cl_2$ (228 mg, 0.984 mmol, 2 mol%). 50 mL of DMF and 50 mL of toluene were added along with a magnetic stirrer. The reaction was heated to 120 °C and left for 90 minutes. After this time, the solution was poured into a beaker containing 250 mL of hexane and 250 mL of distilled water. This caused any polymeric material to precipitate and combine into the water layer. The organic layer was then filtered through celite, and the filtrate was collected. The orange-colored filtrate was washed three times with 250 mL of distilled water to remove any DMF. The solution was then dried with magnesium sulfate and filtered into a round bottomed flask. The hexane and toluene were removed by rotary vacuum to provide a crude mixture of products with approximately 60 % of the original mass. NMR and GC-MS of the crude products showed a very similar distribution of products as the previous experiments involving the reaction of sulfur and norbornadiene.

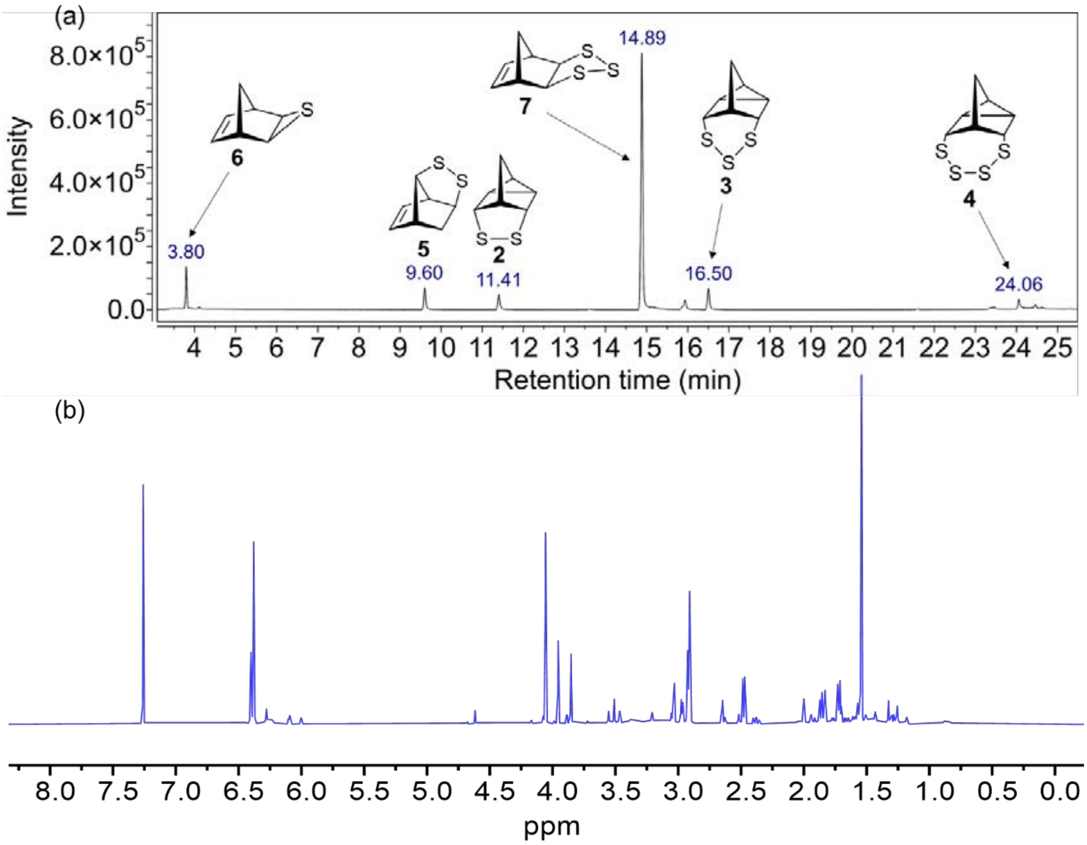

Figure S17: Analysis of crude products obtained from reaction between norbornadiene and sulfur at 120 °C in DMF-toluene using 2 mol% $[Ni(NH_3)_6]Cl_2$. (a) GC-MS chromatogram of hexane soluble crude products. (b) Proton NMR spectrum in deuterated chloroform of hexane soluble crude products.

Purification of intermediates

The cyclic sulfide intermediates were isolated using flash chromatography over silica gel using hexane as a mobile phase. This was done so they could be characterized fully and then be investigated as monomers. The first fraction to elute was a mixture of mostly norbornene-derived epi, tri, and penta-sulfides (molecule **6**, **7** and **8**). These would usually be produced in a ratio of 0.24:1:0.4 of **6**:**7**:**8** respectively. Compounds **7** and **8** could be separated by growing crystals overnight at 4 °C from the neat mixture. Compound **6** remained as an oil which was decanted to remove it from the crystals. The crystals of **7** and **8** could then be washed with cold hexane to remove any residual **6**. In some samples, a small amount of **8** could be isolated by washing the crystals in chloroform and filtering the solution. The crystals of **8** were only soluble in chloroform with extended sonication while the crystals of **7** dissolved readily. Compound **8** could not be detected by GC-MS as it would degrade to form **7** and sulfur in the column but could be detected by NMR spectroscopy. Compound **6** could not be isolated as it would always have some impurity of **7** and **8**. The GC-MS and NMR spectroscopic characterization of each fraction and isolated products are shown below.

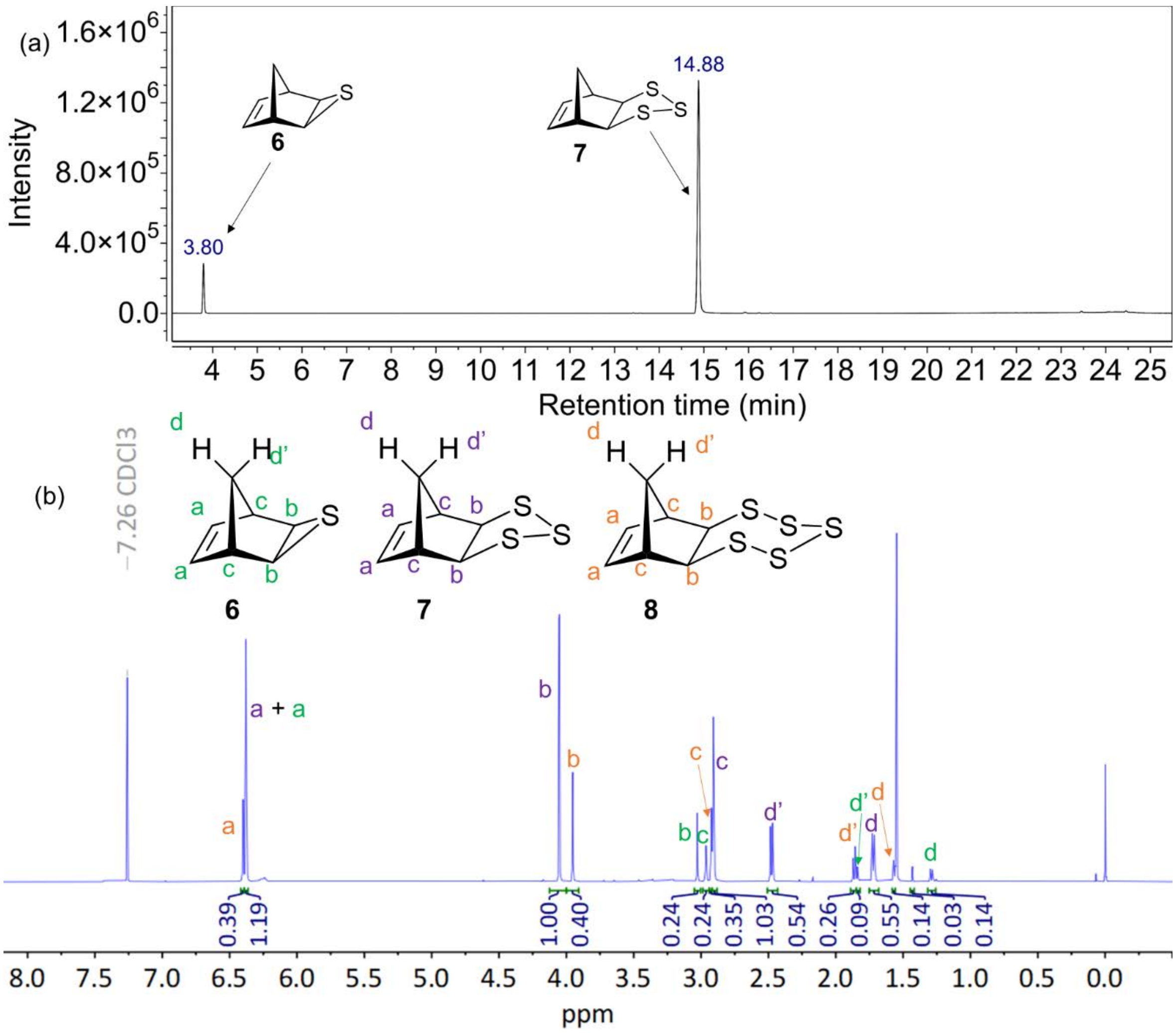

Figure S18: Characterization of fraction one, obtained by column chromatography. (a) GC-MS chromatogram from fraction one. (b) ^{1}H NMR spectrum in deuterated chloroform of fraction one showing peaks for compounds **6**, **7** and **8**.

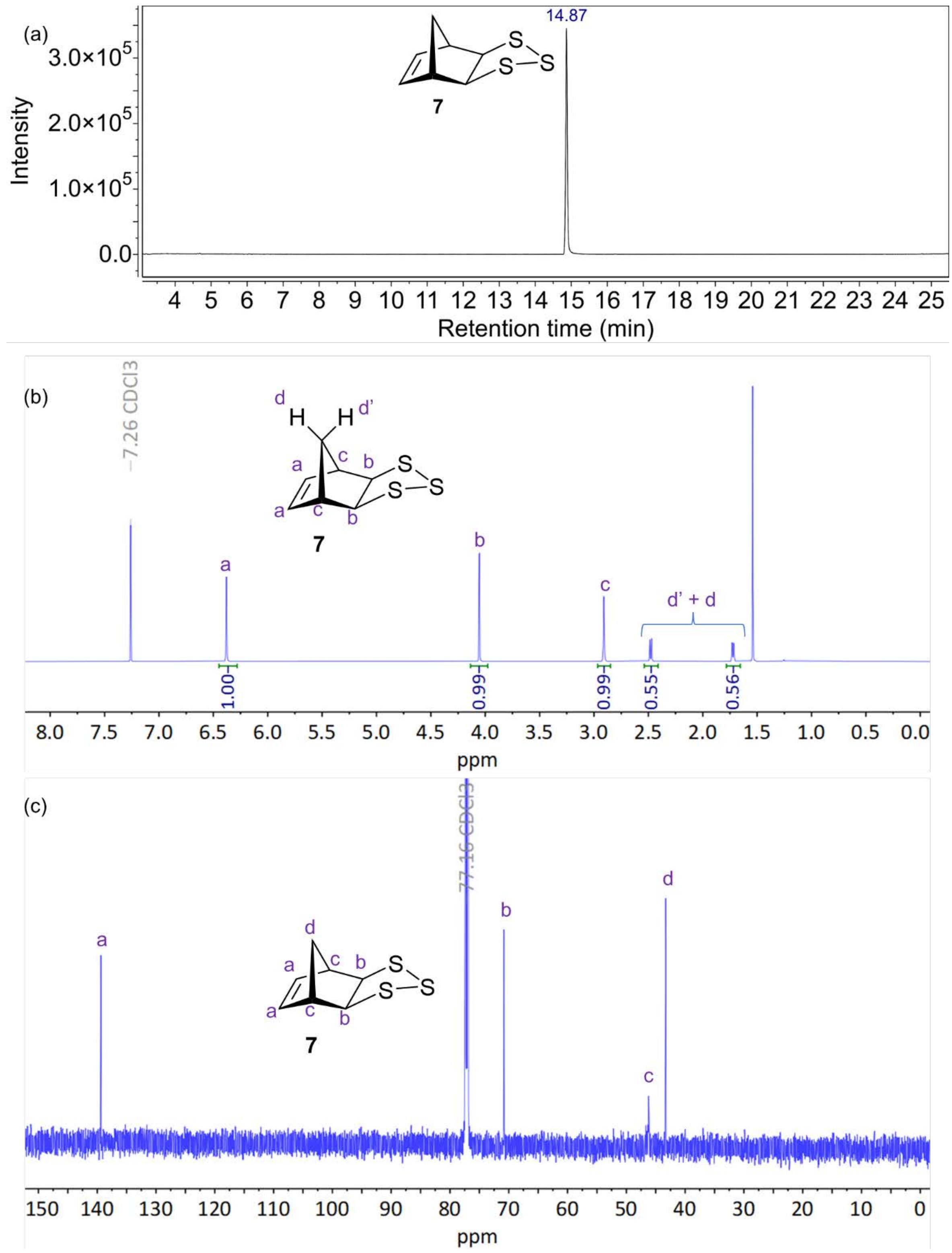

^{1}H NMR (600 MHz, $CDCl_3$) δ 6.38 (m, 2H), 4.05 (d, J = 1.9 Hz, 2H), 2.91 (m, 2H), 2.48 (ap. d, J = 9.3, 1H), 1.72 (m, 1H). **^{13}C NMR** (151 MHz, $CDCl_3$) δ 139.38, 70.81, 46.21, 43.32

Figure S19: Characterization of trisulfide **7**. (a) GC-MS chromatogram of **7** (b) ^{1}H NMR spectrum in deuterated chloroform of **7**. (c) ^{13}C NMR spectrum of **7** in deuterated chloroform.

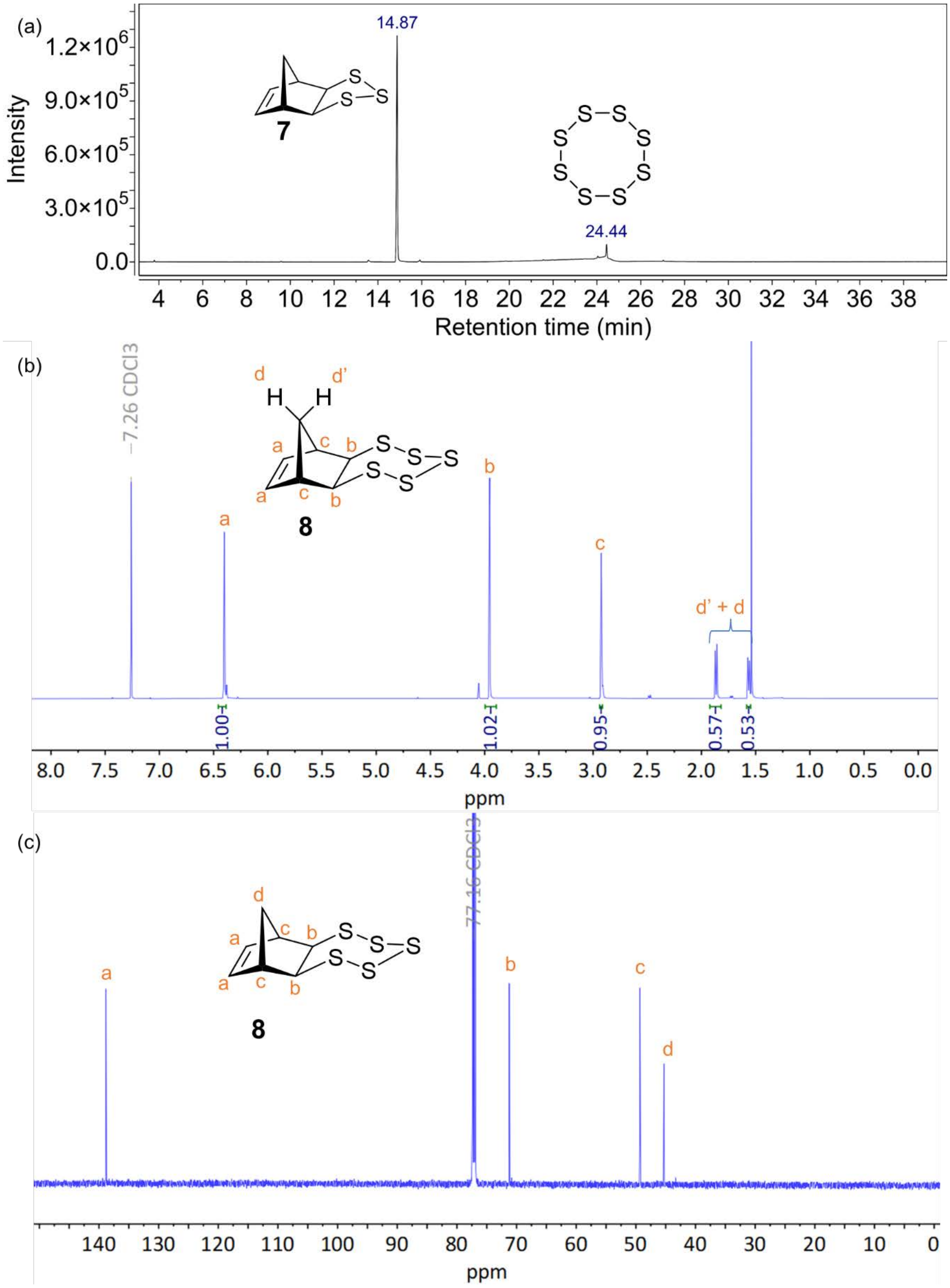

^{1}H NMR (600 MHz, $CDCl_3$) δ 6.40 (m, 2H), 3.95 (d, J = 2.0 Hz, 2H), 2.92 (m, 2H), 1.86 (ap. d, J = 9.4, 1H), 1.56 (m, 1H). **^{13}C NMR** (151 MHz, $CDCl_3$) δ 138.83, 71.22, 49.33, 45.29.

Figure S20: Characterization of pentasulfide **8**. (a) GC-MS chromatogram of **8**, indicating conversion to **7** and sulfur (b) ^{1}H NMR spectrum of **8** in deuterated chloroform. (c) ^{13}C NMR spectrum of **8** in deuterated chloroform.

The second fraction from column chromatography contained compounds **5** and **3**, which are products of rearrangement in the reaction of norbornadiene and sulfur. This fraction also contained bistrisulfide **9**, but **9** could be removed by growing crystals in a dilute solution in hexane. Further purification of this fraction could not be done as it formed a yellow gel over a period of 24 hours which was no longer soluble in chloroform or hexane. The NMR spectra are consistent with those reported by Bartlett and Ghosh[7].

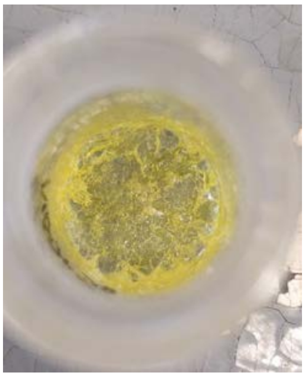

Figure S21: Yellow gel formed over several days from fraction two, indicating that the mixture of compounds **5** and **3** have limited stability when concentrated.

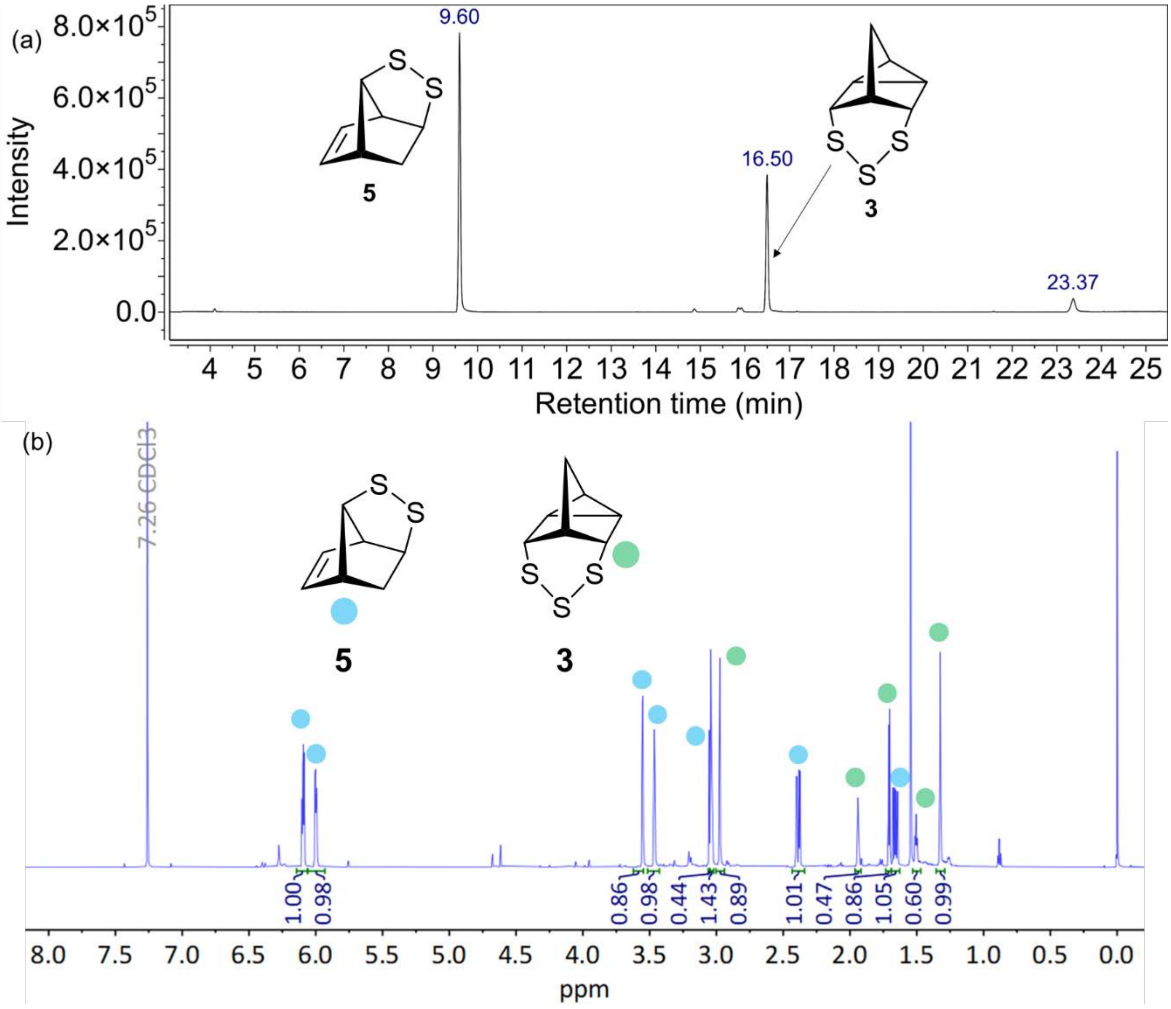

Figure S22: Characterization of fraction two, which contained compounds **5** and **3**. (a) GC-MS chromatogram showing molecules **5** and **3**. (b) ^{1}H NMR spectrum in deuterated chloroform showing peak assignments for molecules **5** and **3**.

The third fraction contained mostly bistrisulfide **9**. Crystals of **9** would form overnight at room temperature from a dilute hexane solution. **9** was not stable in the GC-MS column and gave a broad peak at around 26 minutes and a range of sharper peaks. When using this method, the relative amount of the **9** formed was low and yields were typically less than 5%. A more efficient method was subsequently developed to improve this yield in the "synthesis and purification of molecule **9** and **10**" section on page S33.

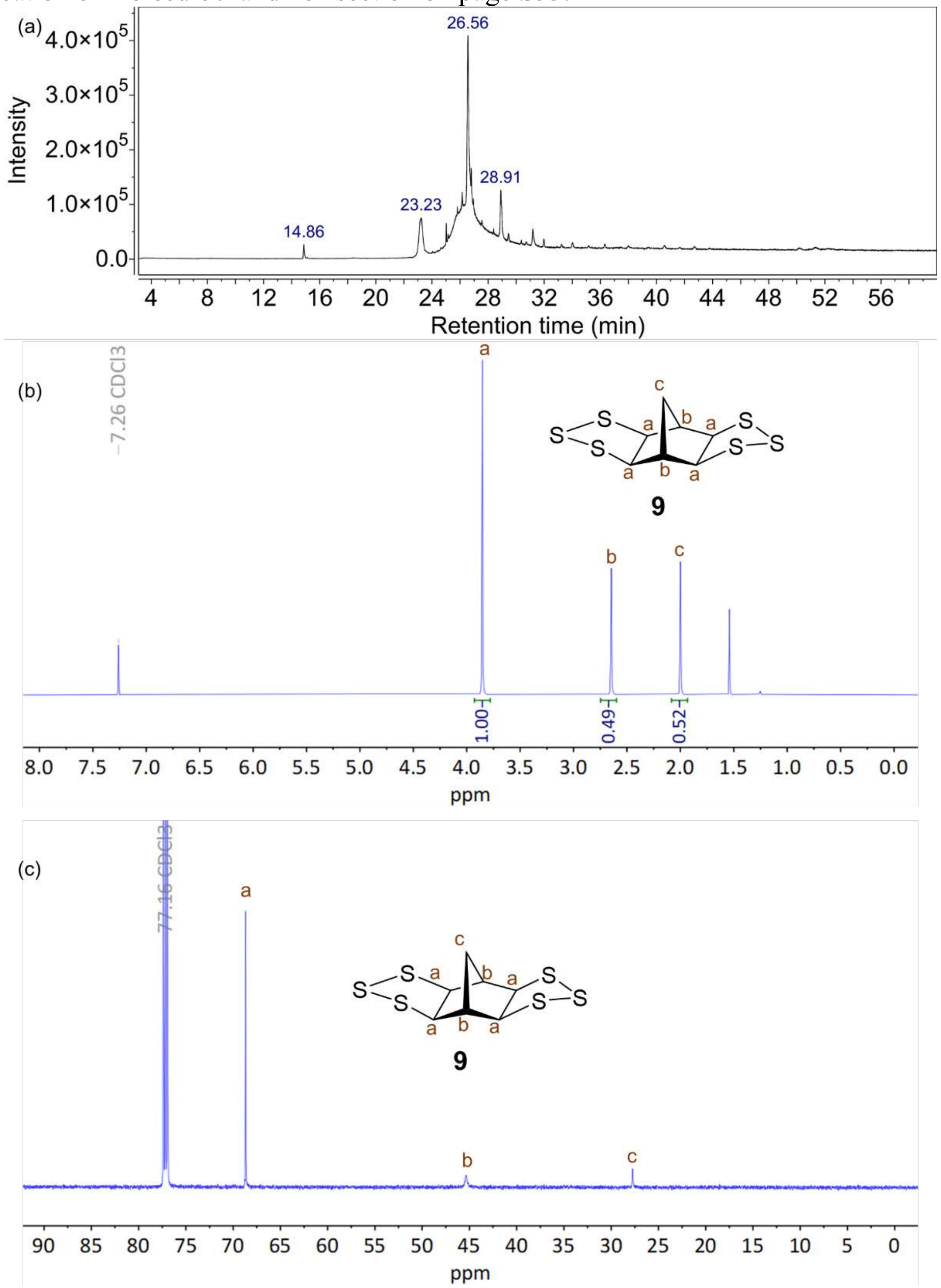

^{1}H NMR (600 MHz, $CDCl_3$) δ 3.85 (ap. s, 4H), 2.65 (ap. s, J = 2.0 Hz, 2H), 2.00 (ap.s, 2H). **^{13}C NMR** (151 MHz, $CDCl_3$) δ 68.68, 45.35, 27.74.

Figure S23: Characterization of bistrisulfide **9**. (a) GC-MS chromatogram of **9** (b) ^{1}H NMR spectrum in deuterated chloroform of **9**. (c) ^{13}C NMR spectrum of **9** in deuterated chloroform.

The fourth fraction contained mostly a rearranged product with a cyclic disulfide (molecule **2**). There was a very small impurity which mostly consisted of the rearranged product with a tetrasulfide ring (molecule **4**). Over several days, **2** would crystallize from the neat mixture.

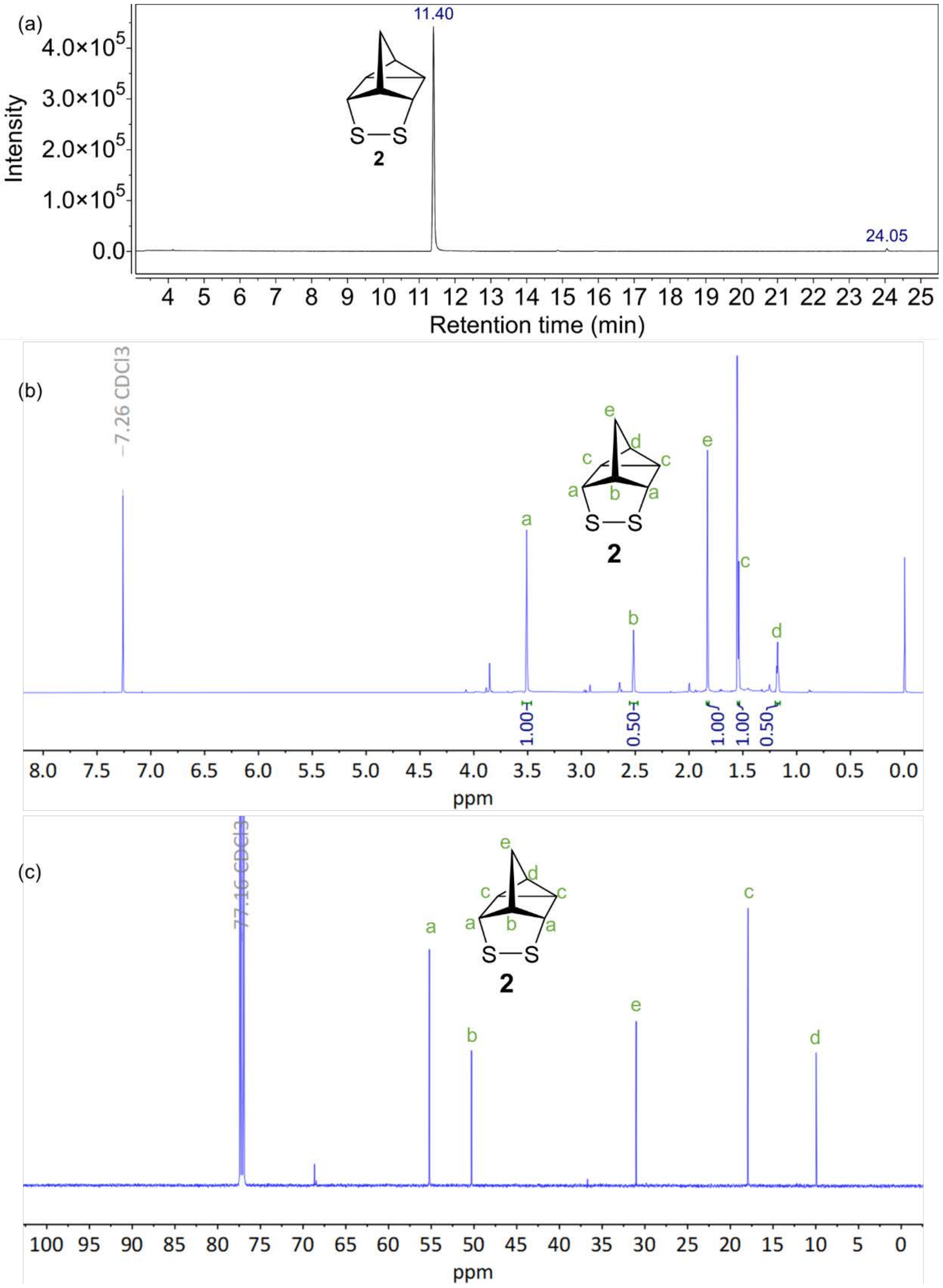

^{1}H NMR (600 MHz, $CDCl_3$) δ 3.51 (m, 2H), 2.52 (m, 1H), 1.83 (t, J = 1.5 Hz, 2H), 1.54 (ap. d, J = 5.0, 2H), 1.18 (td, J = 5.0, 1.2 Hz, 1H). **^{13}C NMR** (151 MHz, $CDCl_3$) δ 55.22, 50.28, 31.01, 17.93.

Figure S24: Characterization of rearranged product from fraction four (molecule **2**). (a) GC-MS chromatogram of **2** (b) ^{1}H NMR spectrum in deuterated chloroform of **2**. (c) ^{13}C NMR spectrum of **2** in deuterated chloroform.

Synthesis and purification of molecules 9 and 10

While the method above was effective in isolating most of the other intermediates, norbornane bistrisulfide (molecule **9**) could only be prepared in a small quantity and low yield. To modify the method to favor the formation of molecule **9**, a reaction time of 24 hours and molar ratio of 6 sulfur atoms for every norbornadiene molecule was used. These modifications were found to improve the yield of **9**. Accordingly, a 250 mL round bottomed flask was charged with sulfur (9.450 g, 295 mmol S atoms), norbornadiene (5.00 mL, 4.53 g, 49.15 mmol) and $[Ni(NH_3)_6]Cl_2$ (228 mg, 0.984 mmol, 2 mol%). 50 mL of DMF and 50 mL of toluene were added along with a magnetic stirrer. The flask was equipped with a condenser and heated at 120 °C for 24 hours. After this time, the solution was poured into a beaker containing 250 mL of hexane and 250 mL of distilled water. Polymeric material precipitated and settled in the bottom layer. The organic layer was filtered through celite, and the filtrate was collected. The orange-colored filtrate was washed three times with 250 mL of distilled water to remove any DMF. The solution was then dried with magnesium sulfate and filtered into a round bottomed flask. The washing and filtering steps were done quickly because **9** would start to precipitate during the procedure. The round bottomed flask was then sealed and left overnight. Bistrisulfide **9** would precipitate as a yellow powder over 24 hours and would usually stick to the side of the glass. The hexane solution was carefully decanted to avoid any loss of molecule **9**. Toluene was then added to the round bottomed flask to aid in the removal of **9**. Sonication was required to remove it from the glassware. Toluene was then removed by rotary evaporation. ^{1}H NMR analysis indicated that **9** co-precipitated with molecule **10**. If a pure crystalline sample was required, a hot recrystallization in hexane gave pure **9** with a melting point of 179 °C. A maximum yield of ~10% of **9** and **10** was obtained using this protocol.

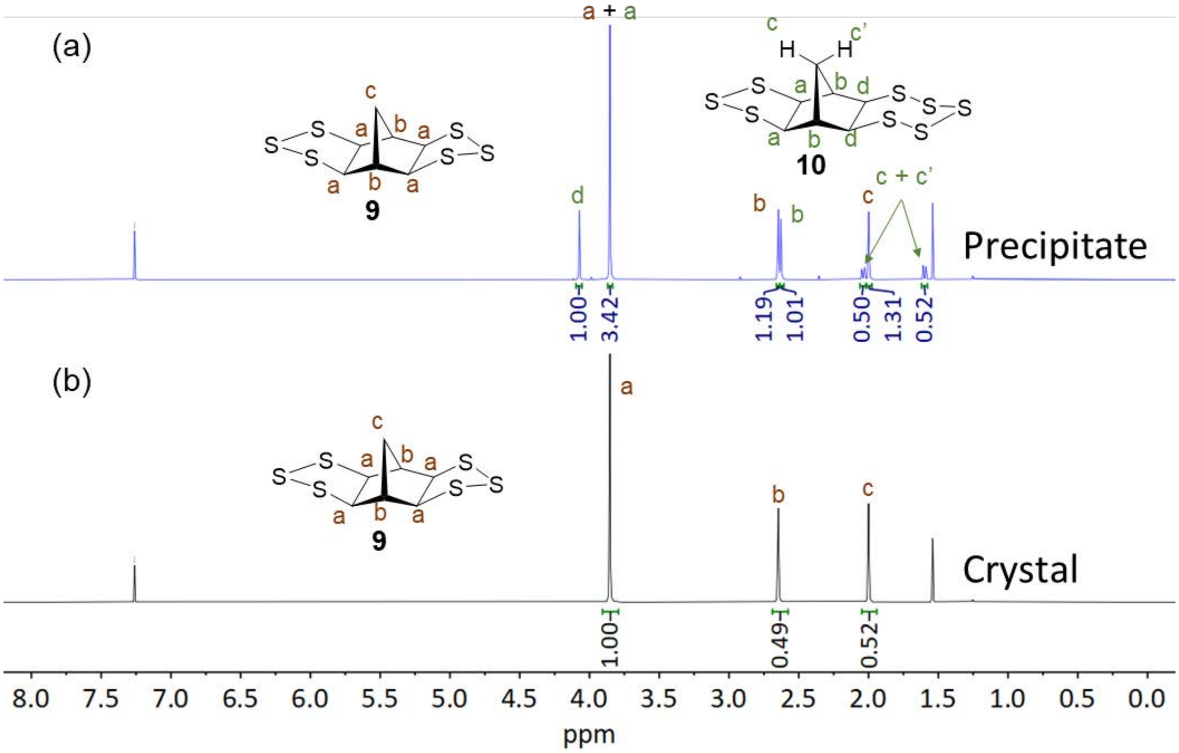

Figure S25: (a) ^{1}H NMR spectrum of precipitate showing both molecule **9** and **10** in deuterated chloroform. (b) ^{1}H NMR spectrum of **9** in deuterated chloroform, after purification by recrystallization from hexane.

X-ray crystallography of isolated cyclic sulfides

Crystals of norbornene trisulfide (**7**), norbornene pentasulfide (**8**), bistrisulfide (**9**), and the rearranged disulfide (**2**) were prepared and analyzed at the Australian Synchrotron. The data showed that the sulfur chain was in the exo configuration for **7**, **8** and **9**. In contrast, the C-S bonds in compound **2** were axial in configuration. Single crystals were mounted in paratone-N oil on a nylon loop. X-ray diffraction data was collected at 100(2) K on the MX-1 or MX-2 beamline of the Australian Synchrotron (λ = 0.7107 Å).[1,2] Structures were solved by direct methods using SHELX[3] and refined with SHELXL[4] and ShelXle[5] as a graphical user interface. All non-hydrogen atoms were refined anisotropically, and hydrogen atoms were included as invariants at geometrically estimated positions.

Table S1: X-ray experimental data for molecules **7**, **8**, **9**, and **2.**

**Compound**	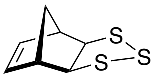 **7**	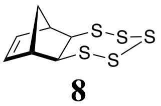 **8**	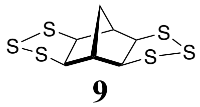 **9**	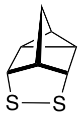 **2**
**CCDC number**	2289708	2305218	2289709	2305219
**Empirical formula**	$C_7H_8S_3$	$C_7H_8S_5$	$C_7H_8S_6$	$C_7H_8S_2$
**Formula weight**	188.31	252.43	284.49	156.25
**Crystal system**	Orthorhombic	triclinic	Monoclinic	monoclinic
**Space group**	Pbcn	P-1	$P2_1/c$	P 21/m
a **(Å)**	42.045(8)	9.0240(18)	6.7640(14)	5.8750(12)
b **(Å)**	7.7730(16)	9.921(2)	13.008(3)	9.2290(18)
c **(Å)**	9.6930(19)	11.724(2)	12.204(2)	6.527(2)
**α (º)**	90	87.16(3)	90	90
**β (º)**	90	71.21(3)	100.82(3)	108.75(3)
**γ (º)**	90	86.85(3)	90	90
**Volume ($Å^3$)**	3167.8(11)	991.6(4)	1054.7(4)	335.12(15)
Z	16	4	4	2
**Density (calc.) (mg/m^3)**	1.579	1.691	1.792	1.548
**Absorption coefficient (mm^{-1})**	0.849	1.107	1.243	0.686
**F(000)**	1568	520.0	584	164.0
**Crystal size (mm^3)**	0.17 x 0.15 x 0.12	0.23 x 0.12 x 0.11	0.25 x 0.21 x 0.18	0.22 x 0.08 x 0.07
**θ range for data collection (º)**	0.969 to 27.882	1.836 to 28.597	2.310 to 27.882	3.296 to 28.506
**Reflections collected**	46785	12602	15666	4165
**Observed reflections [R(int)]**	3673 [0.0385]	3247 [0.0361]	2510 [0.0538]	720
**Goodness-of-fit on F^2**	1.105	1.081	1.080	1.083
**R_1 [I>2σ(I)]**	0.0300	0.0373	0.0301	0.0306
**wR_2 (all data)**	0.0806	0.0990	0.0768	0.0902
**Largest diff. peak and hole (e.Å-3)**	0.388 and -0.389	1.213 and -0.465	0.614 and -0.530	0.293 and -0.510
**Data / restraints / parameters**	3673 / 0 / 181	3433/0/217	2510 / 0 / 119	720/0/46

Thermal ellipsoid plots

Trisulfide **7**

Yellow crystals of **7** were grown overnight from a neat solution of fraction one at 4 °C. The crystals were washed with cold hexane to remove episulfide **6**. Crystals of both trisulfide **7** and pentasulfide **8** were obtained by this method. The trisulfide **7** crystals could be easily recognized as they were yellow while the crystals of the pentasulfide **8** were clear. The structure was solved in the orthorhombic space group Pcbn and refined to an R1 value of 3%. The asymmetric unit contains two norbornene trisulfide molecules. The melting point of the crystal was 138 °C.

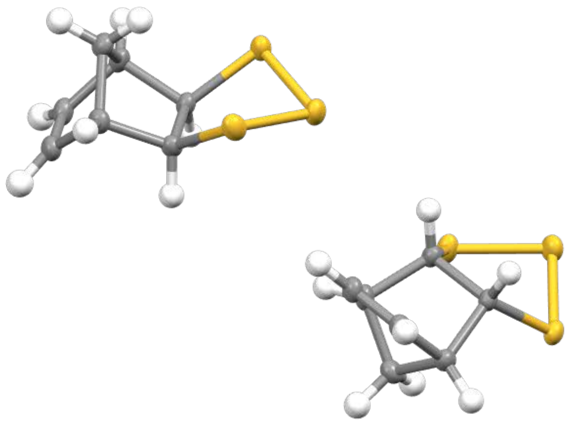

Figure S26: Image of trisulfide **7** asymmetric unit with all non-hydrogen atoms shown as ellipsoids at the 50 % probability level.

Pentasulfide **8**

White crystals of pentasulfide **8** were grown overnight at 4 °C from a neat solution of fraction one. Crystals of both the trisulfide **7** and pentasulfide **8** were formed in this protocol. To purify pentasulfide **8**, the crystals were washed with chloroform. Trisulfide **7** dissolved in chloroform and the pentasulfide **8** remained as a crystal that did not readily dissolve in chloroform. The structure was solved in the triclinic space group P1 and refined to an R1 value of 3.63 %. The asymmetric unit contains two molecules of pentasulfide **8**. The melting point of the crystal was 93 °C.

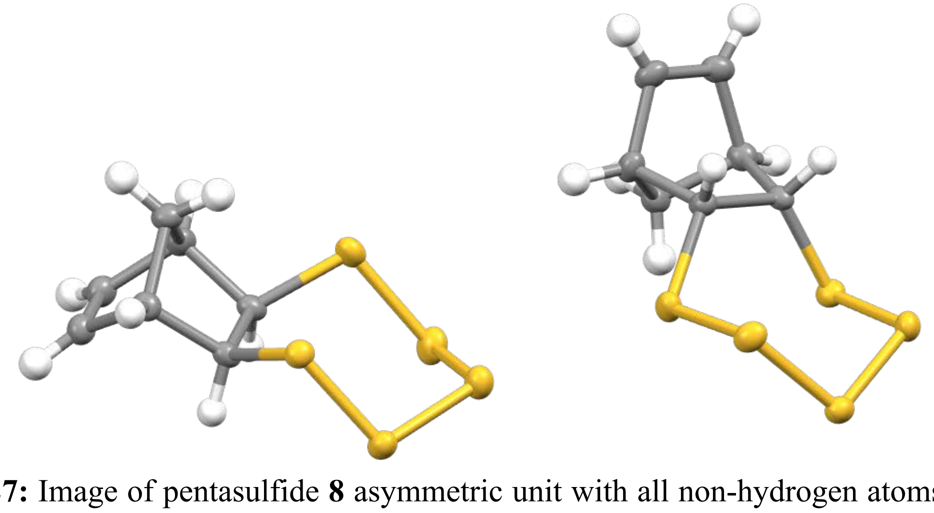

Figure S27: Image of pentasulfide **8** asymmetric unit with all non-hydrogen atoms shown as ellipsoids at the 50 % probability level.

Bistrisulfide **9**

Yellow crystals of bistrisulfide **8** were grown directly from hexane after separation by flash chromatography. The structure was solved in the monoclinic space group $P2_1c$ and refined to an R1 value of 3.01%. The asymmetric unit contains a single molecule of **8**. The melting point of the crystal was 179 °C.

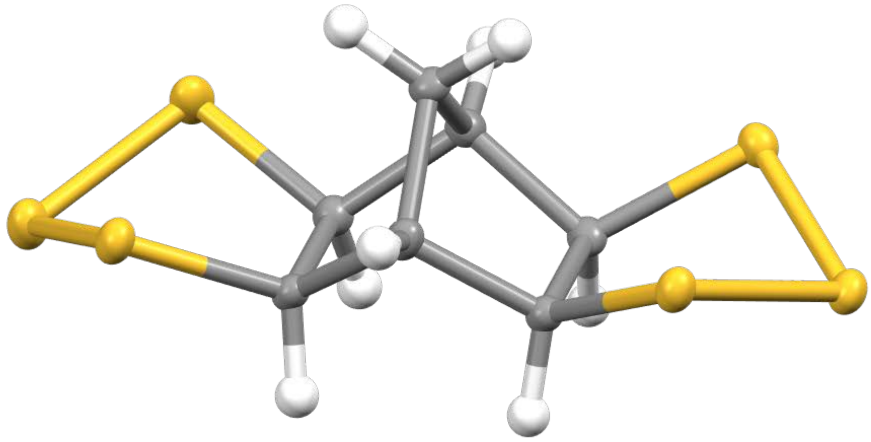

Figure S28: Image of bistrisulfide **9** asymmetric unit with all non-hydrogen atoms shown as ellipsoids at the 50 % probability level.

Rearranged disulfide **2**

Crystals of disulfide **2** formed from the neat mixture from fraction four over several days at room temperature. The structure was solved in the monoclinic space group $P2_1/n$ and refined to an R1 value of 3.1 %. The asymmetric unit contains a single molecule of **2**. The melting point of the crystal was 63 °C.

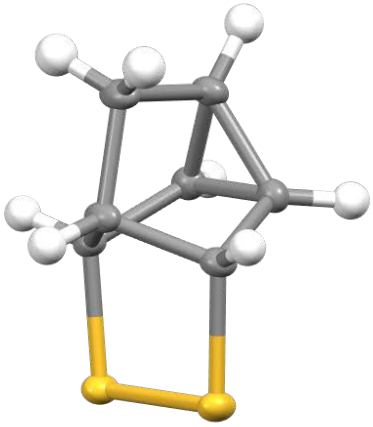

Figure S29: Image of a disulfide **2** asymmetric unit with all non-hydrogen atoms shown as ellipsoids at the 50 % probability level. The cyclopropane product of rearrangement is clearly shown in the crystal structure.

ATR-FTIR analysis of cyclic sulfides

The IR spectrum of all norbornadiene derived cyclic sulfides were tested using a Bruker Vertex 80 V Spectrometer using the ATR attachment. The polymers were first ground into a fine powder using a mortar and pestle before being analyzed. Alkene peaks could be observed at 3140 cm^{-1}, 3060 cm^{-1} and 730 cm^{-1} in spectra containing **6**, **7** or **8**. The rearranged disulfide **2** had several characteristic peaks from the cyclopropane unit at 3065 cm^{-1} and 804 cm^{-1}. The peak at 3065 cm^{-1} would overlap with the alkene peak from alkenes if present but the bending peak at 804 cm^{-1} is very useful for identifying the presence of the cyclopropane unit.

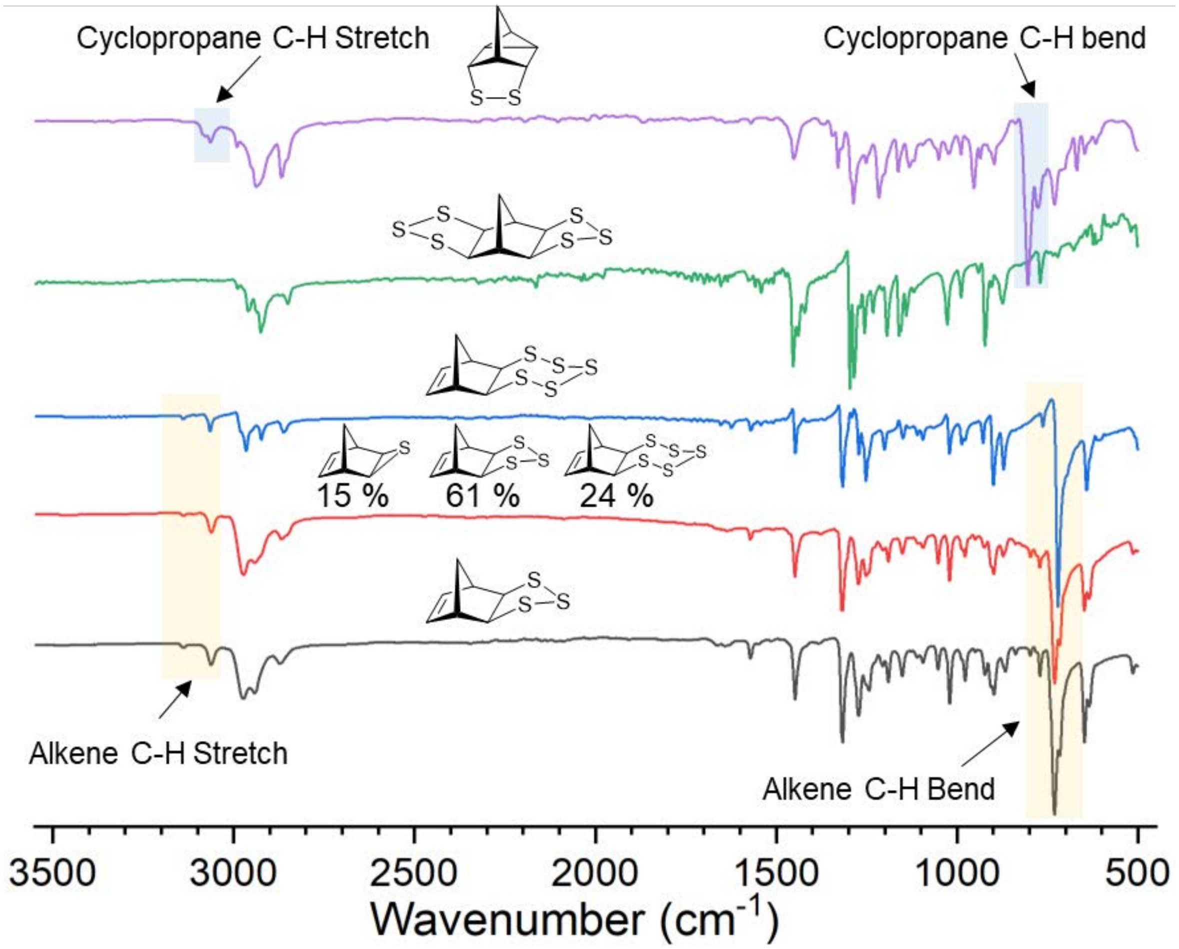

Figure S30: FTIR spectra from cyclic sulfides isolated from a reaction between sulfur and norbornadiene. Peaks for the cyclopropane C-H functional group in molecule **2** are labelled blue. Peaks for the alkene C-H functional group in molecules **6**, **7** and **8** are labelled yellow.

Ball Milling of Norbornadiene and Sulfur

Ball milling method

Ball milling was performed using a Planetary Ball Mill PM 100 (Retsch). Norbornadiene (6.00 g, 65.1 mmol) and sulfur (14.0 g, 436.8 mmol S atoms) were added to a 250 mL agate grinding jar with 45 agate grinding balls (ø 10 mm). The mixture was ball milled for 3 hours at 500 RPM with rotation inversion cycles of 15 minutes and 5 second pauses between inversion cycles. The jar was opened, and solid material was dislodged from the walls of the reactor vessel using a spatula. Milling was resumed for 3 hours at 500 RPM with rotation inversion cycles of 15 minutes and 5 second pauses between inversion cycles. The jar was opened, and the grinding balls were observed to be clumped together with solid material; they were dislodged from each other by hand. Milling was resumed for 1 hour at 600 RPM with rotation inversion cycles of 15 minutes and 5 second pauses between inversion cycles. A final milling for 1 hour at 100 RPM with rotation inversion cycles of 10 minutes and 10 minute pauses between inversion cycles was performed. The solid material was collected for further analysis; material stuck to the agate balls was dislodged using a hammer. A total of 12.542 g of a beige-brown solid were recovered and further analyzed.

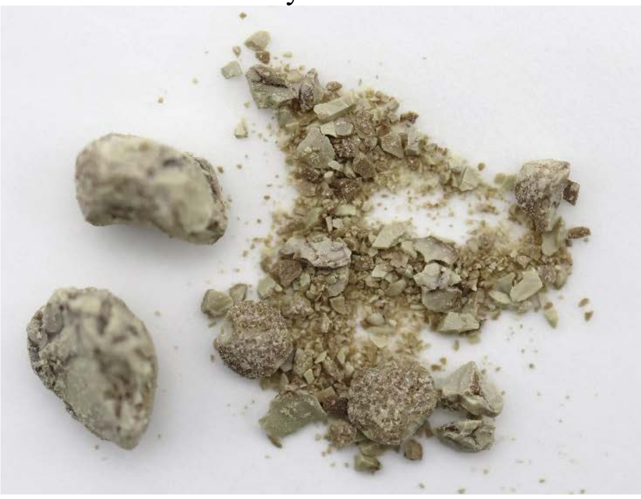

Figure S31: Image of solids obtained from ball milling sulfur and norbornadiene.

Characterization of ball milled material

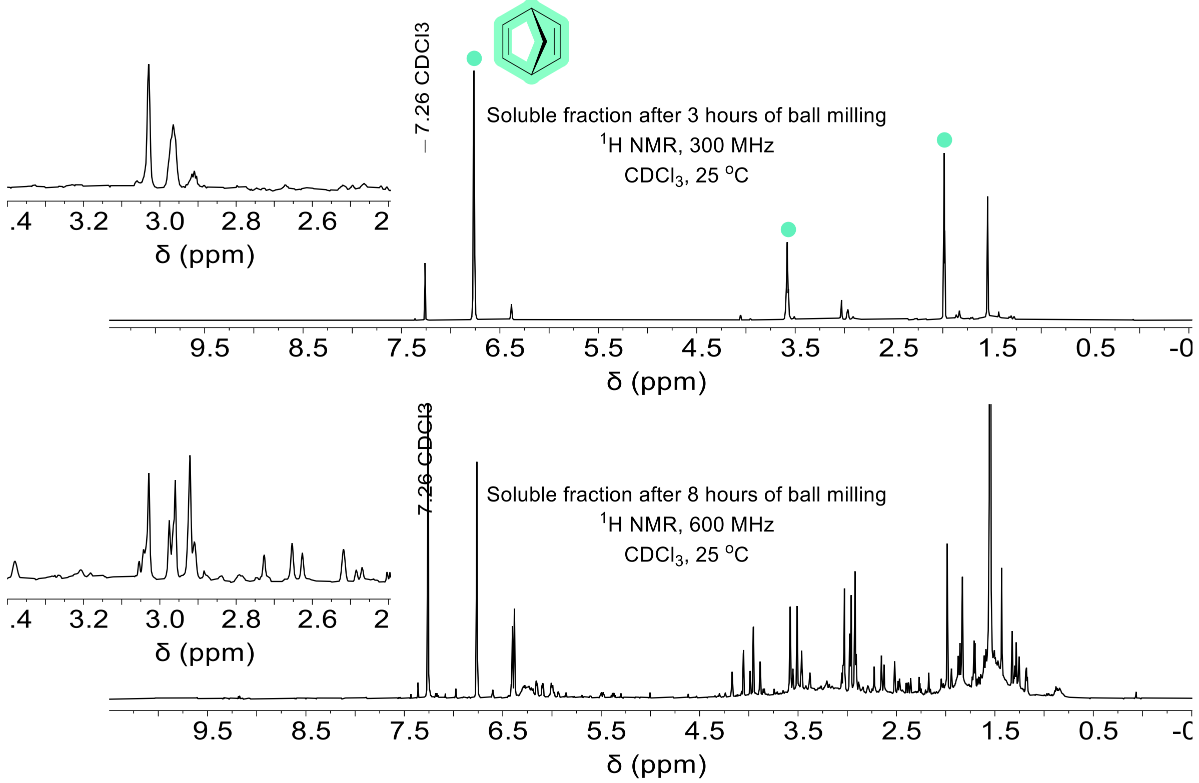

Figure S32: ^{1}H NMR spectra comparison of the $CDCl_3$ soluble fraction following ball milling of sulfur and norbornadiene after 3 hours (top) and 8 hours (bottom). Proton signals for norbornadiene have been highlighted in the top spectrum.

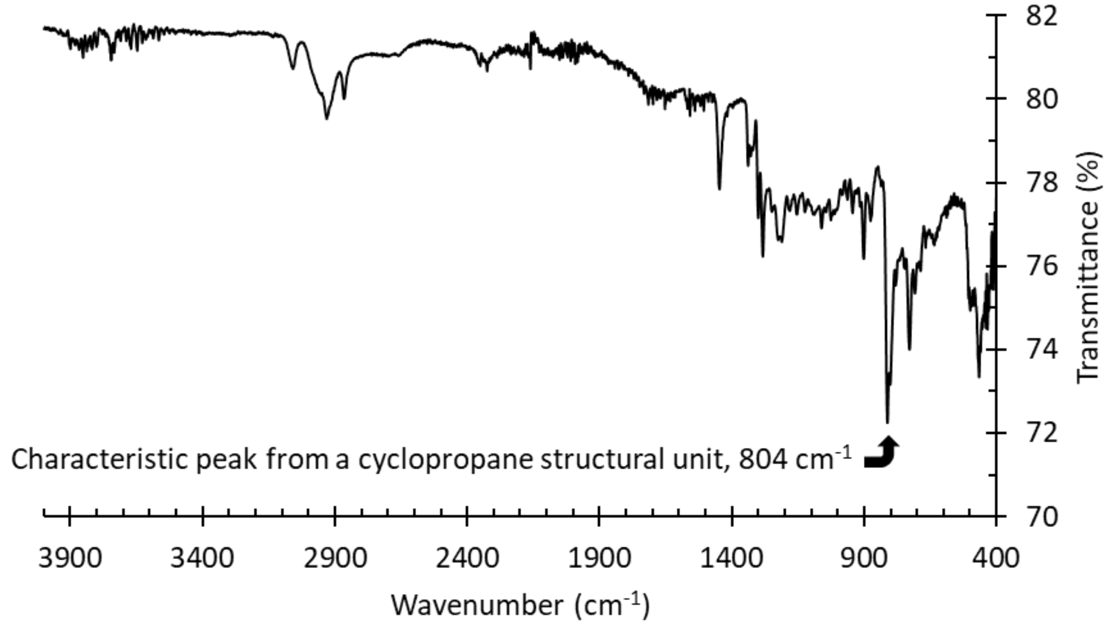

Figure S33: ATR-FTIR spectra of ball milled material from the reaction between norbornadiene and sulfur. The absorption peak at 804 cm^{-1} is characteristic of a cyclopropane structural motif. See figure S30.

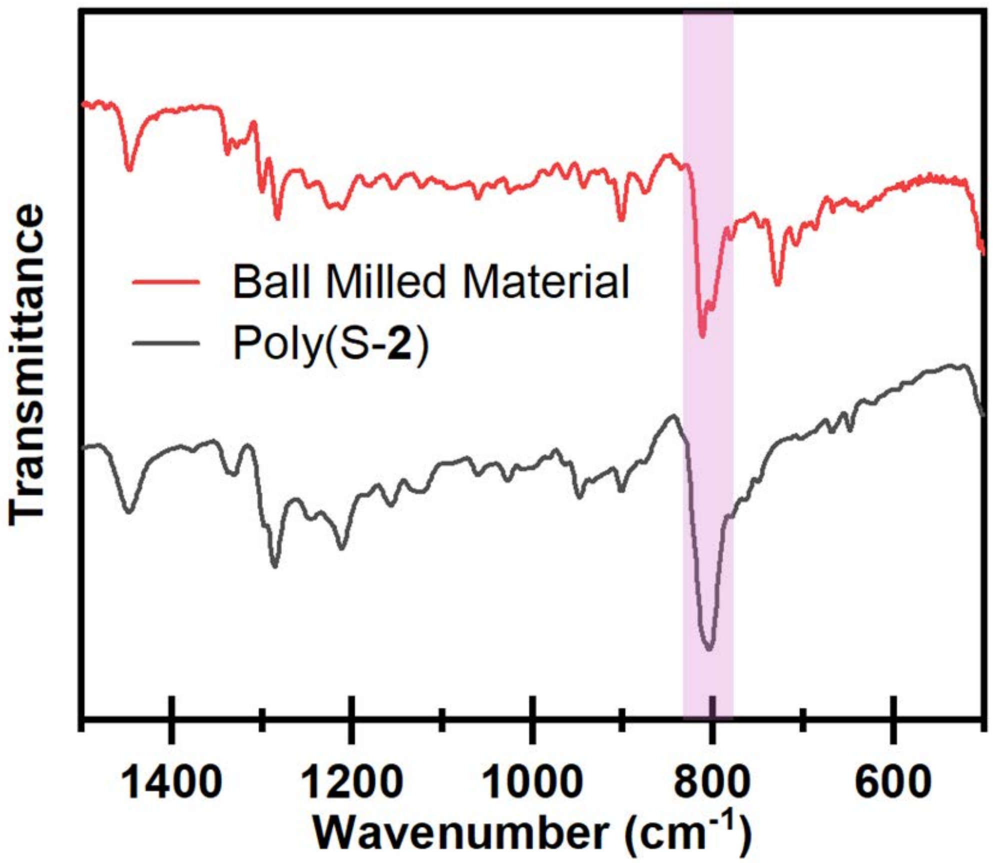

Figure S34: ATR-FTIR spectra of ball milled material from the reaction between norbornadiene and sulfur compared with poly-S**2**. The highlighted absorption peak at 804 cm^{-1} is characteristic of a cyclopropane structural motif and is observed in both samples. See figure S30.

Reduction and GC-MS analysis of ball milled norbornadiene and sulfur

30 mg of the polymer was ground into a powder and added to a flame dried vial and capped. The vial was purged with nitrogen. A 1 M solution of $LiAlH_4$ in anhydrous tetrahydrofuran (THF) was prepared and 5 mL was injected into the vial. The reaction was left for 24 hours with constant stirring. After this time, the reaction was cooled using an ice bath. 5 mL of 1 M hydrochloric acid (HCl) was injected slowly to quench the reaction. 5 mL of hexane was then injected into the mixture and stirred for an additional hour. The organic layer was separated and analyzed by GC-MS using an Agilent single quadrupole GC-MS.

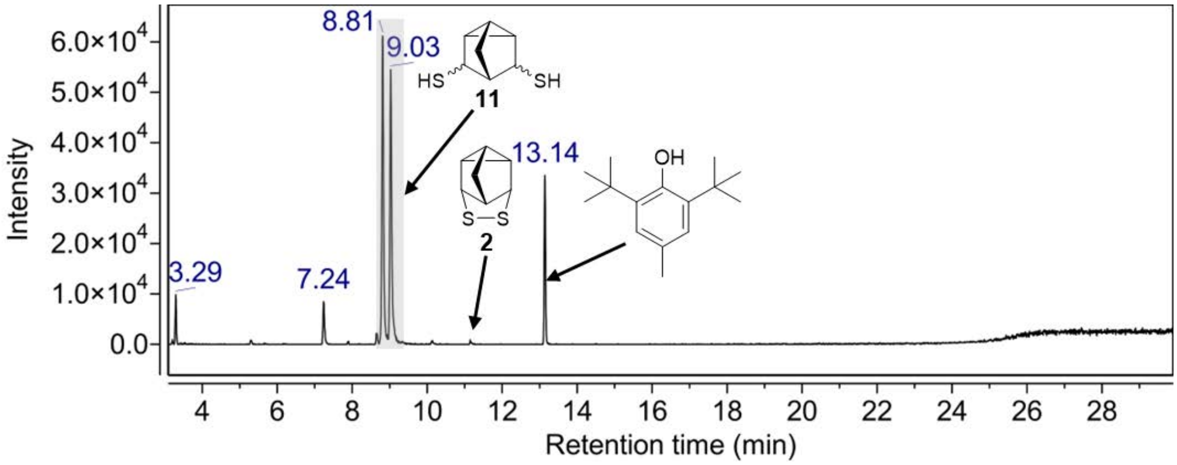

Figure S35: GC chromatogram of material from the reduction of ball milled norbornadiene and sulfur by $LiAlH_4$, indicating the major products contain a cyclopropane unit resulting from rearrangement.

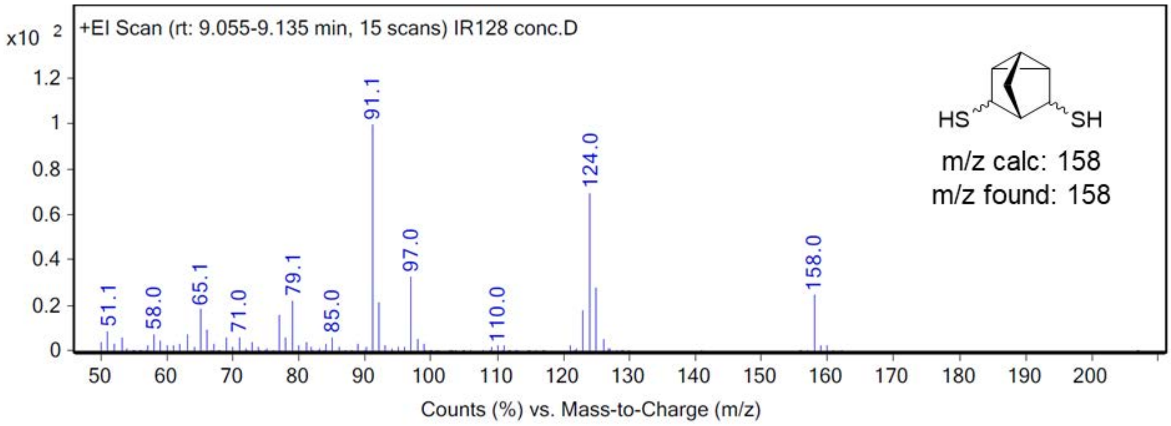

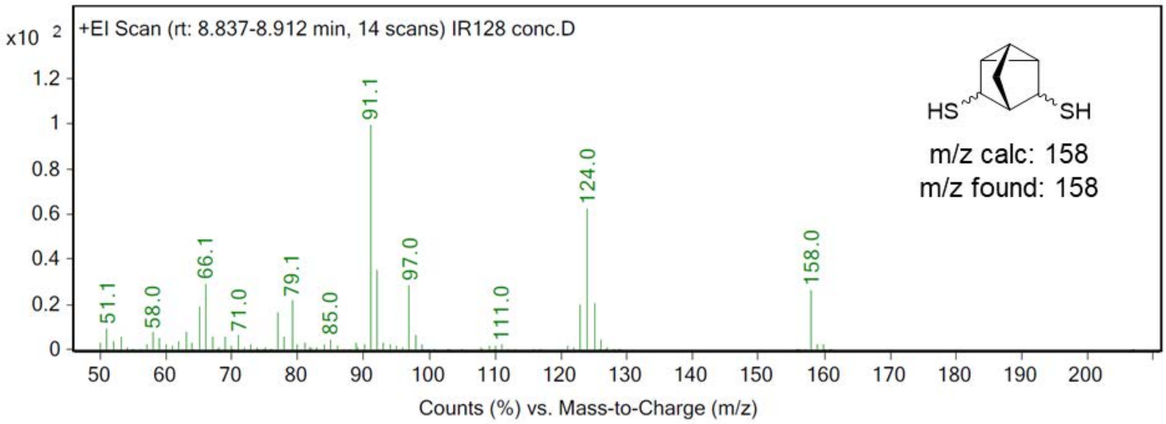

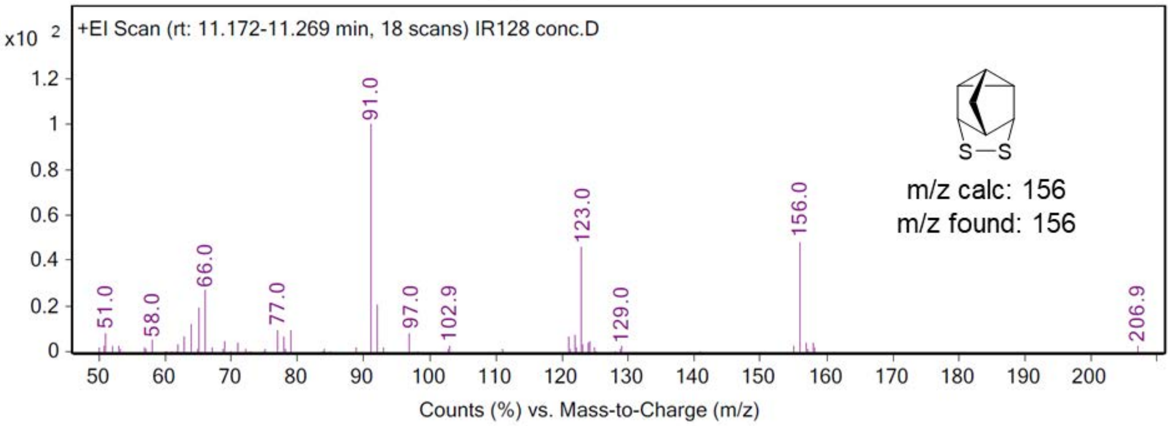

Figure S36: Mass spectra of material from the reduction of ball milled norbornadiene and sulfur by $LiAlH_4$.

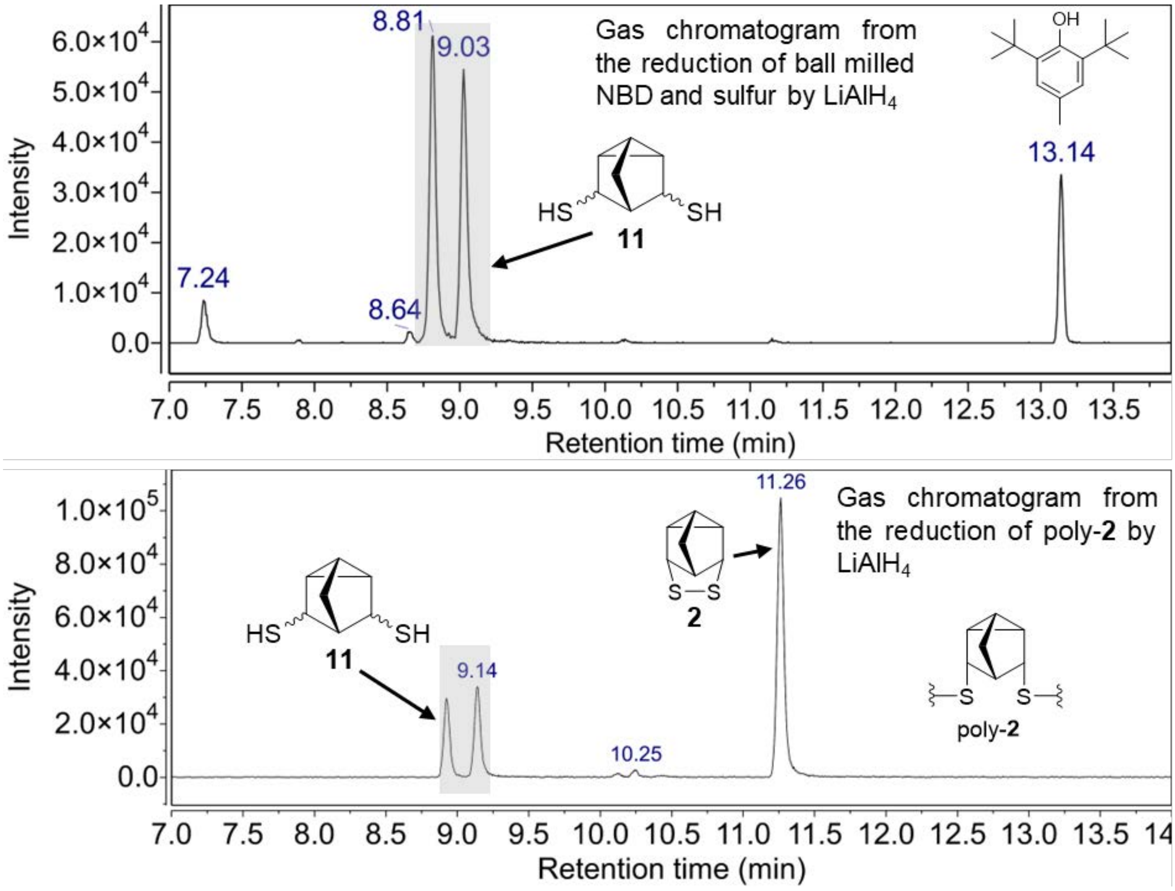

Figure S37: Comparison of the GC chromatogram of material from the reduction of ball milled norbornadiene and sulfur by $LiAlH_4$ (top), with synthesized poly-**2** that was reduced with $LiAlH_4$ (bottom). Signals related to cyclopropane containing products are observed in both chromatograms and the mass spectra corresponding to these GC peaks were the same for both samples.

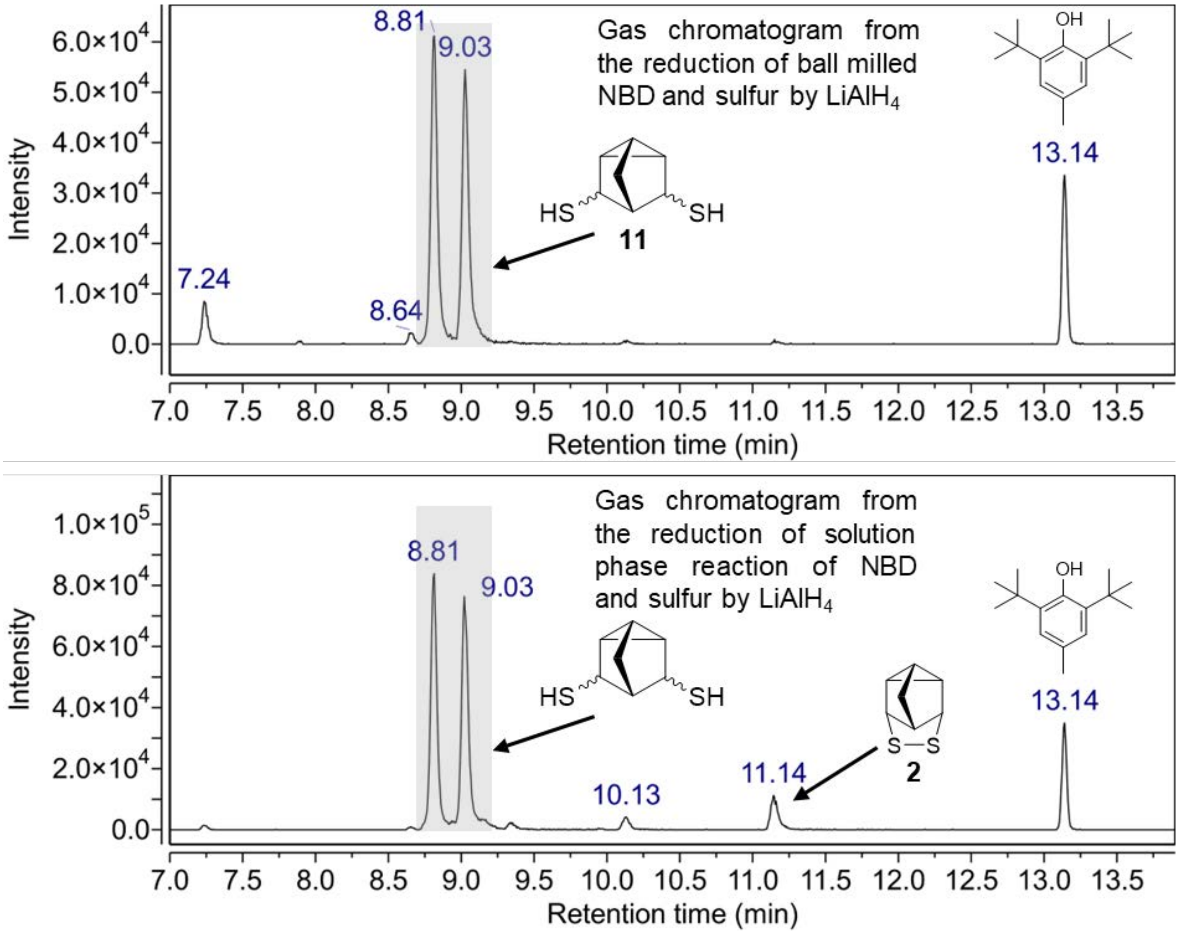

Figure S38: Comparison of the GC chromatogram of material from the reduction of ball milled norbornadiene and sulfur by $LiAlH_4$ (top), with material from the reaction of sulfur and norbornadiene in solvent, after reduction with $LiAlH_4$ (bottom). The mass spectra for the product peaks were the same for both samples.

Polymer synthesis and characterization using cyclic sulfides as monomers

Polymerization method using cyclic sulfides

Purified trisulfide **7**, bistrisulfide **9**, and disulfide **2** were each investigated as monomers. Additionally, another set of reactions were performed using molecules **6**, **7**, and **8** in the same ratio as observed in the reaction of norbornadiene and sulfur. This ratio was determined by ^{1}H NMR spectroscopy as 0.24:1:0.4 for **6**:**7**:**8**. The purified intermediates were reacted under similar conditions used previously in the reaction of norbornadiene and sulfur. This experiment was to determine if these intermediates can be incorporated into polymer products. For each molecule, four types of reaction were carried out: One where the sulfides were heated neat with no added sulfur. Another where the sulfides were heated in a solvent system of 50-50 DMF-xylene with no added sulfur. These reactions were done to see if the sulfides could undergo a homopolymerization, potentially by ring opening of the cyclic sulfide ring. Two more reactions were done where sulfur was added to determine if copolymerization with sulfur was possible and if the alkene reacts. The samples made with **7** or the combination of **6**, **7** and **8** had an amount of sulfur added so that approximately 66% of sulfur was present by mass. Such sulfur loadings correspond to the amount of sulfur in the samples using only bistrisulfide **9**. The polymers made with **2** had enough sulfur added to provide an average sulfur rank of 4 which corresponded to approximately 58% sulfur. All reactions were done with 100 mg of reactants. The sulfides were added to a vial with a stir bar. 1 mL of DMF and Xylene was added to the reactions in the samples with solvent and sulfur was added to the appropriate samples. The mass of the sulfides and sulfur to make 100 mg is summarized in the table below. The vials were then added to a preheated oil bath at 140 °C and heated with stirring for 90 minutes. After this time, they were removed from the oil bath and the magnetic stir bar was removed. Any solvent was evaporated using rotary evaporation and the samples were added to a preheated oven at 140 °C for 24 hours to cure. These reaction conditions had to be modified when **9** was used due to its high melting point. For the reactions using **9** without solvent, they were first heated to 185 °C for 5 minutes before following the same method as the other samples. When solvent was used, **9** would dissolve and the standard method was used, as described above.

Table S2: Table showing mass of sulfides and sulfur used in polymerization reactions

**Sample**	**Sulfide mass**	**Sulfur mass**	**Sulfur Content (%)**
**7, no S, neat**	100 mg	0	51 %
**7, with S, neat**	69 mg	31 mg	66 %
**7, no S, with solvent**	100 mg	0	51 %
**7, with S, with solvent**	69 mg	31 mg	66 %
**6, 7 and 8; no S, neat**	100 mg	0	53 %
**6, 7 and 8; with S, neat**	68.5 mg	31.5 mg	66 %
**6, 7 and 8; no S, with solvent**	100 mg	0	53 %
**6, 7 and 8; with S, with solvent**	68.5 mg	31.5 mg	66 %
**9, no S, neat**	100 mg	0	67 %
**9, with S, neat**	87 mg	13 mg	72 %
**9, no S, with solvent**	100 mg	0	67 %
**9, with S, with solvent**	87 mg	13 mg	72 %
**2, no S, neat**	100 mg	0	41 %

**2, with S, neat**	71 mg	29 mg	58 %
**2, no S, with solvent**	100 mg	0	41 %
**2, with S, with solvent**	71 mg	29 mg	58 %

All samples showed a significant change after the reaction forming a brittle material after the curing process. The reactions using **7**, **9** or the combination of sulfides (**6**, **7**, and **8**) made a yellow-orange glassy material when reacted neat. When DMF-Xylene was used as a solvent, the samples were much darker orange but were still brittle and glassy. The samples made with **2** formed a dark brown material. There was no obvious difference in color or physical properties between the neat and solution-processed samples for molecule **2**. There was also no obvious difference when additional sulfur was added, and all sulfur reacted and was incorporated into the product.

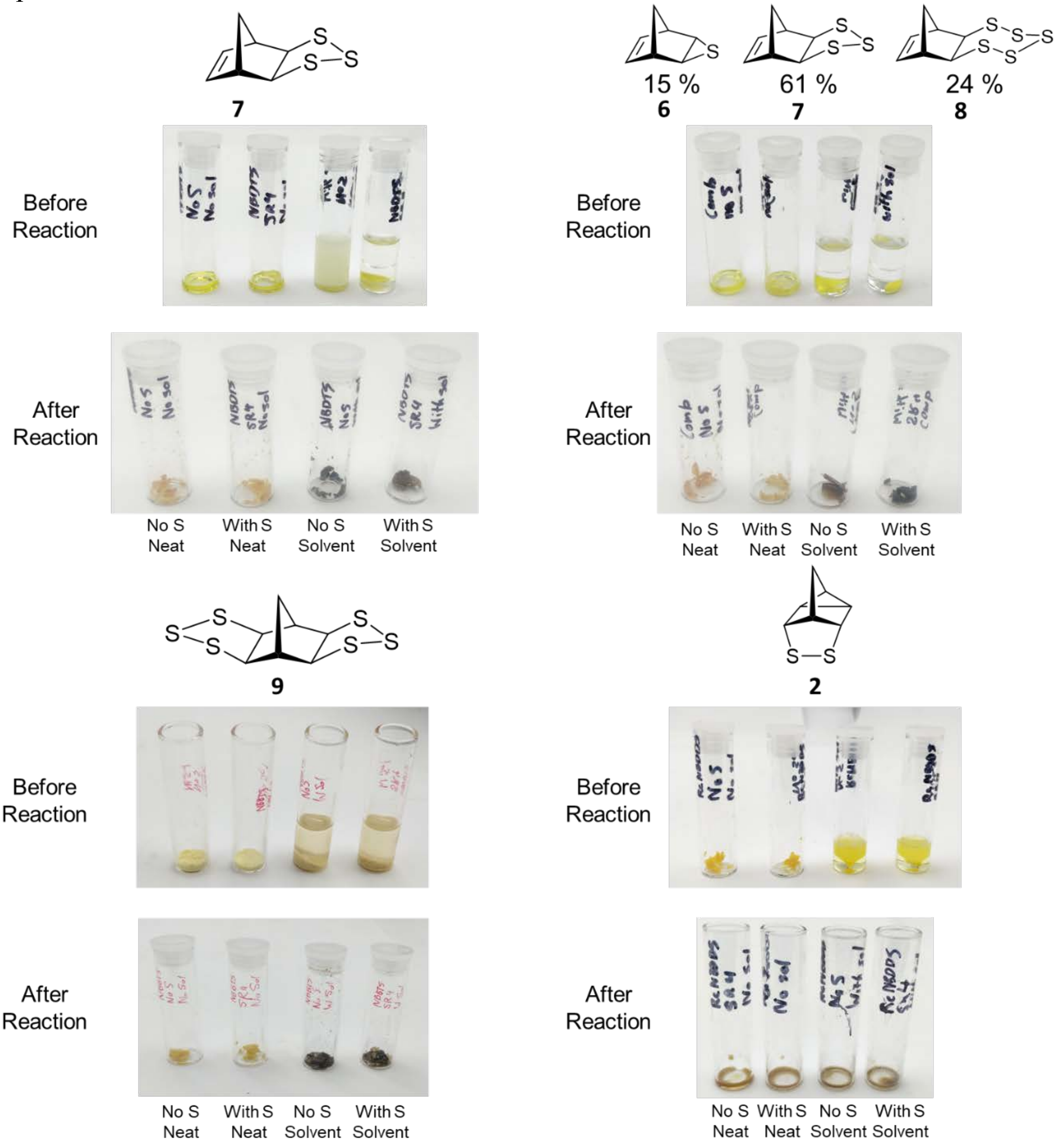

Figure S39: Images showing the reactions using independently synthesized and isolated cyclic sulfides. The solvent system used in this reaction was 50-50 DMF-xylene.

Optimized polymerization method using 9 and 10 to prepare polymer 1

The melting point of bistrisulfide **9** (179 ºC) was higher than the other cyclic sulfide monomers investigated. Because this melting point is higher than the reaction temperatures described in the previous section (140 ºC), some modifications to the polymerization method were required to ensure complete consumption of **9** in its homopolymerization or copolymerzation with sulfur.

For reactions where the polymer *did not need to be cast*, the reaction was first heated to 185 °C for 5 minutes to melt monomer **9** and initiate its homopolymerization or copolymerization with sulfur. Within 15 seconds at 185 ºC, the reaction vitrifies so there is insufficient time for casting into a mold. After 5 minutes at 185 ºC, the reaction mixture was then moved to a second oil bath preheated to 140 ºC and heated for 90 minutes, followed by further curing at 140 ºC for 24 hours in an oven. The reaction is likely complete during the short 185 ºC heating period, but the additional heating was still carried out to be consistent with the previous section.

To make a *castable polymerization reaction*, molten sulfur was used as a solvent and comonomer. The organic monomer could be purified **9** or a mixture of monomers **9** and **10**. In these cases, the working time of the reaction could be increased to approximately 3 minutes before vitrification. While this working time is short, it was sufficient for casting the polymerization mixture into a mold for making polymer windows or lenses. This method was best suited for reactions in which the total sulfur content is 80% or greater. In such cases the monomers could be fully dissolved in the molten sulfur before vitrification—an important consideration for homogeneity and preventing unreacted **9** or **10** from contaminating the vitrified polymer product.

The standard method for making and casting polymer **1** is described here for a 750 mg reaction, but reactions as large as 10 g have been successfully completed using this protocol. First, monomer **9** or mixtures of monomers **9** and **10** were processed in a Soxhlet extractor with hexane to provide them in an amorphous, powdered form that readily dissolved in molten sulfur. This powdered form of **9** or the mixture of **9** and **10** was dried under vacuum for several hours to remove solvent before subjecting the monomers to polymerization. If crystalline samples of **9** were used, the dissolution in molten sulfur was slower, which led to a risk of unreacted and crystalline **9** contaminating the vitrified polymer product. The Soxhlet extraction and drying process was also effective at preventing any solvent from contaminating the monomer. Second, molten sulfur was degassed under reduced pressure immediately before the polymerization to remove water or other volatile species that could form bubbles in the cast polymer. The specific details are described below:

*Protocol for making and casting polymer **1**:* Sulfur (300 mg, 9.36 mmol S atoms) was added to a 5 mL vial. The vial was added to a preheated oil bath at 140 °C. The sulfur was heated for 3 minutes at 140 ºC, causing it to melt. The vial of molten sulfur was then transferred to vacuum oven preheated to 140 °C. The pressure in the oven was decreased to approximately 2 mbar and held for approximately 30 minutes with continued heating at 140 ºC. After degassing the sulfur, the sample was returned to atmospheric pressure and immediately returned to the 140 °C oil bath. Monomer **9** (445 mg, 1.56 mmol) was then added to the molten sulfur in one portion. This quantity of sulfur and **9** provided a final polymer product containing 81% sulfur by mass. The reaction mixture was stirred gently with a heated spatula to ensure the that **9** was fully mixed with the molten sulfur. This manual stirring was continued for a total of 45 seconds from the time monomer **9** was added. After this time, the reaction was homogenous and not mixed further. After 60 seconds of total reaction time, the reaction mixture was poured into a

silicone mold that was preheated to 140 ºC. The mold was then placed in a 100 °C oven where it was left for 1 hour to cure.

*Note on reaction time for making and casting polymer **1**:* Only 60 seconds of reaction is recommended before casting. This amount of time ensures that the mixture is homogenous but still has a sufficiently low viscosity to be cast into a mold. With longer reaction times, the viscosity rapidly increases, and the mixture vitrifies, which prevents casting. This recommendation applies to copolymerizations of sulfur and **9**, as well as copolymerizations of sulfur and mixtures of **9** and **10**.

If mixtures of monomer **9** and **10** are used in the copolymerization with sulfur, ^{1}H NMR spectroscopy was first used to quantify the ratio of **9** and **10**, and the amount of elemental sulfur was adjusted accordingly to provide the desired composition in the final polymer.

Below are some images of polymer windows prepared using this method. The polymer was yellow and transparent.

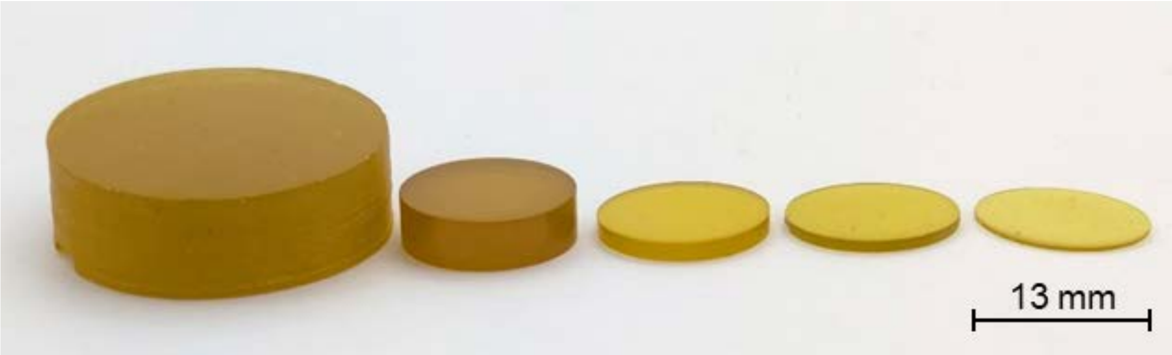

Figure S40: Images of polymer **1** windows with a sulfur content of 81% by mass.

ATR-FTIR of polymers made with purified norbornadiene-derived sulfides

The FTIR spectrum of all polymers made from the purified sulfide monomers were tested using a Bruker Vertex 80 V Spectrometer using the ATR attachment. The polymers were first ground into a fine powder using a mortar and pestle before analysis. All samples were tested under vacuum from 5000 cm^{-1} to 500 cm^{-1} over a total of 30 scans. The polymers made from **7** had very similar infrared spectra to the polymerization with **6**, **7** and **8** in a ratio of 0.21:1:0.4. They both showed very little change in the alkene peaks when no sulfur was added. When sulfur was added to the reaction, the alkene peaks decreased in intensity. This was more noticeable in the polymer prepared in solvent (DMF-xylene) as the alkene peaks were almost completely consumed. These results show that when no additional sulfur was added, the reaction likely proceeds through a ring opening polymerization. However, when additional sulfur is added into the reaction, the alkene can also react.

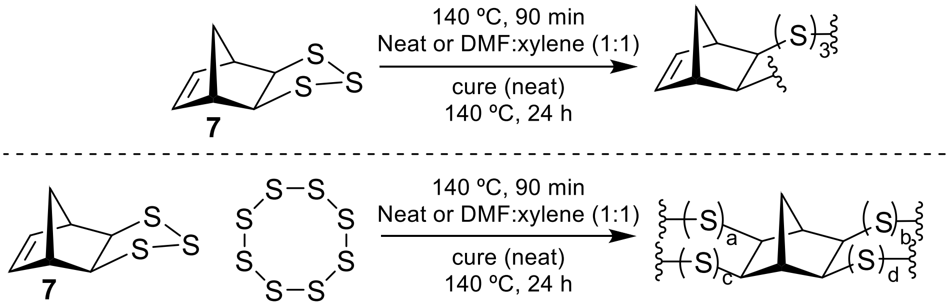

Figure S41: Polymerization reactions using trisulfide monomer **7**

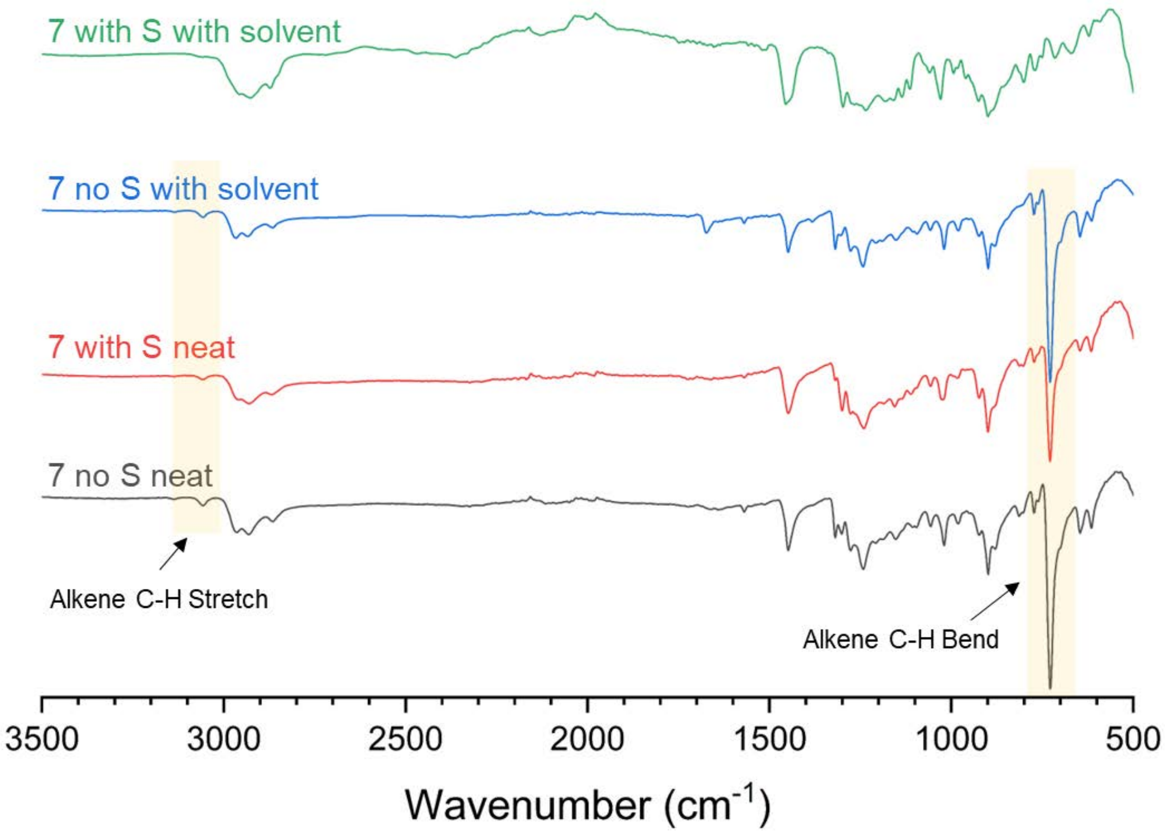

Figure S42: ATR-FTIR spectra of polymers made from **7**. Samples labelled with S have approximately 66% sulfur. The samples prepared in solvent were made in a DMF-xylene mixture.

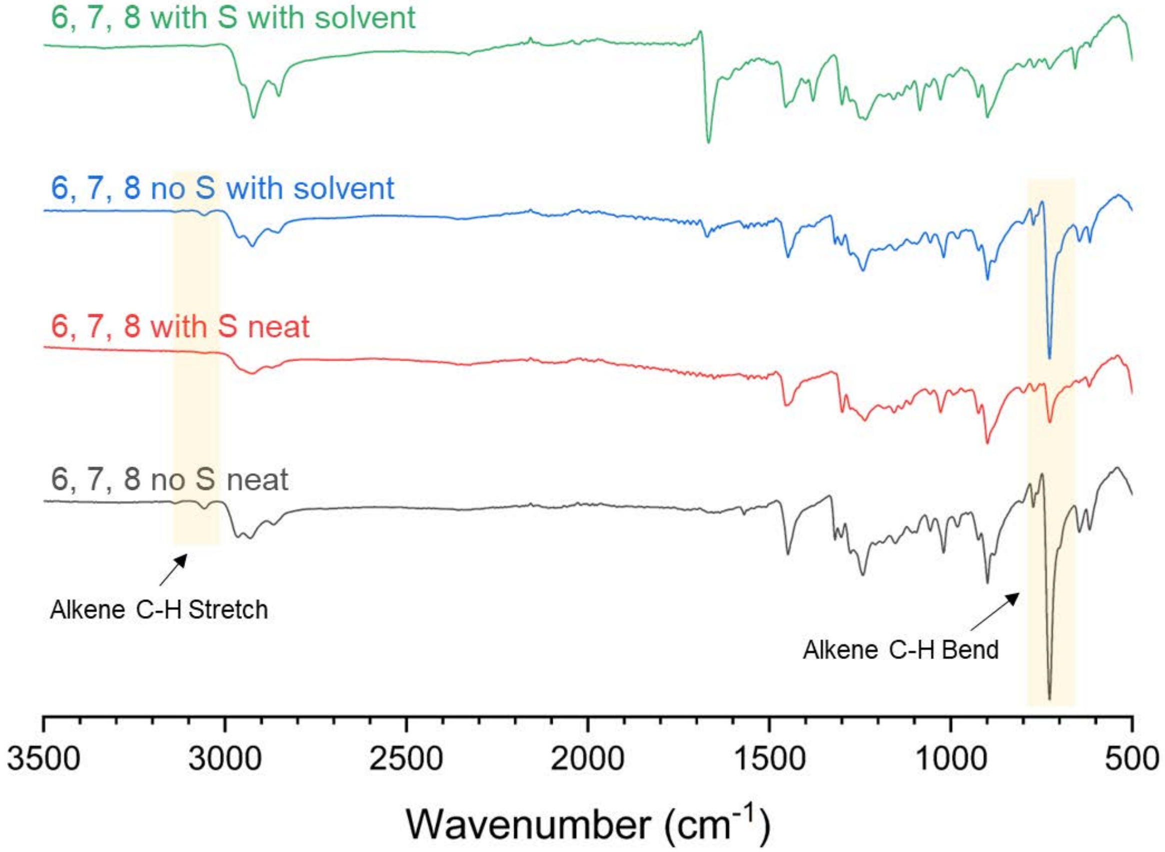

Figure S43: ATR-FTIR spectra of polymers made from a mixture of **6**, **7**, and **8** in a 24:100:40 ratio. Samples labelled with S have approximately 66% sulfur. The samples prepared in solvent were made in a DMF-xylene mixture.

When **9** was polymerized, the sharp peaks broadened slightly but there was no significant change in the infrared spectrum of the corresponding polymer. This is consistent with ring-opening polymerization and no reaction of the organic components of the polymer.

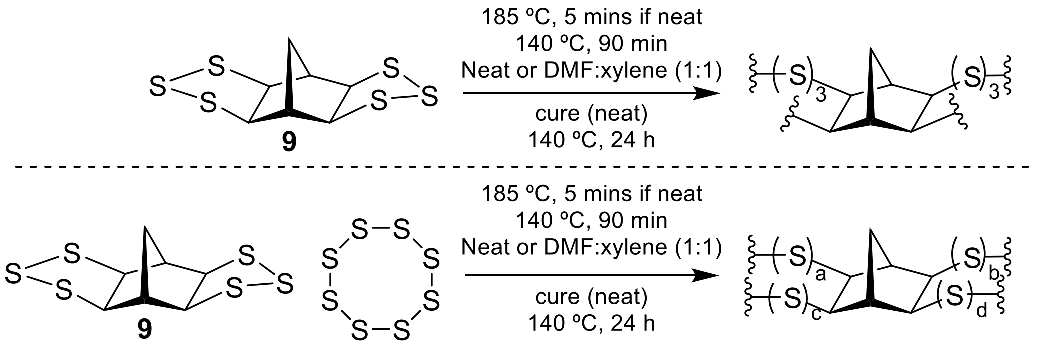

Figure S44: Polymerization reactions using bistrisulfide **9**.

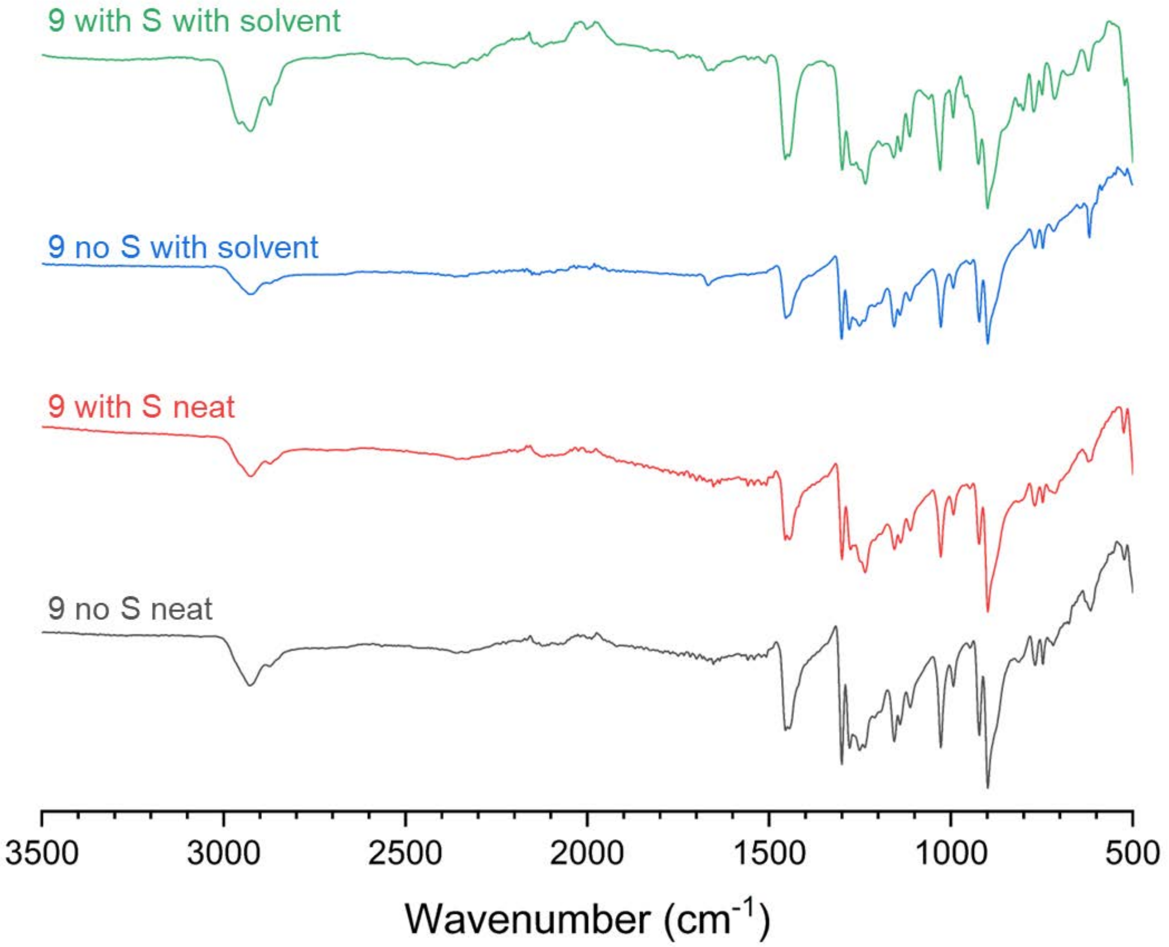

Figure S45: ATR-FTIR spectra of polymers made from **9**. Samples labelled with S have approximately 72% sulfur. The samples prepared in solvent were made in a DMF-xylene mixture.

The polymers made from **2** all had a similar FTIR spectra. This result is consistent with ring-opening polymerization of the cyclic disulfide and no reaction in the organic domain of the polymer. The large peak at 804 cm^{-1} was visible in all samples, indicating the cyclopropane unit remains intact in the polymerization.

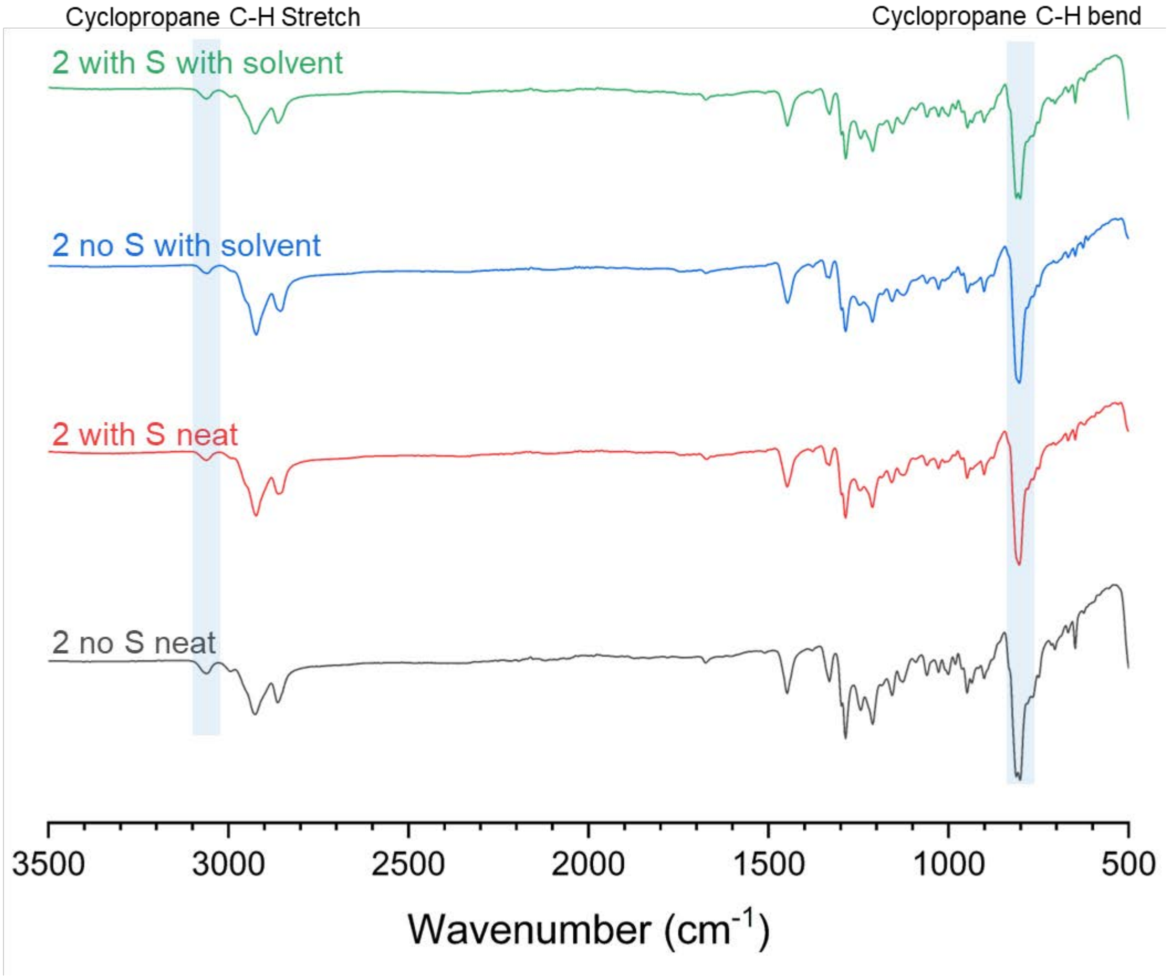

Figure S46: ATR-FTIR spectra of polymers made from **2**. Samples labelled with S have approximately 58% sulfur. The samples prepared in solvent were made in a DMF-xylene mixture.

Reduction of polymers and analysis with GC-MS

While the FTIR data provided some insight into the structure of the polymers, additional analysis was required to understand the microstructures formed in the polymers. The polymers were not soluble, so they could not be analyzed by solution-based NMR spectroscopy. Instead, the polymers were reduced with lithium aluminum hydride ($LiAlH_4$) to break the S-S bonds and provide components soluble in THF. Analyzing these products by GC-MS provides insight into the microstructures of the polymer. In the reductions, 30 mg of the polymer was ground into a powder and added to a flame dried vial and capped. The vial was purged with nitrogen. A 1 M solution of $LiAlH_4$ in anhydrous tetrahydrofuran (THF) was prepared and 5 mL was injected into the vial. The reaction was left for 24 hours with constant stirring. After this time, the round bottomed flask was added to an ice bath to cool. To quench the reaction, 5 mL of 1 M hydrochloric acid (HCl) was injected slowly to quench the reaction. To extract the organic products, 5 mL of hexane was added and the mixture was stirred for 1 hour. The organic layer was separated and analyzed by GC-MS using an Agilent single quadrupole GC-MS.

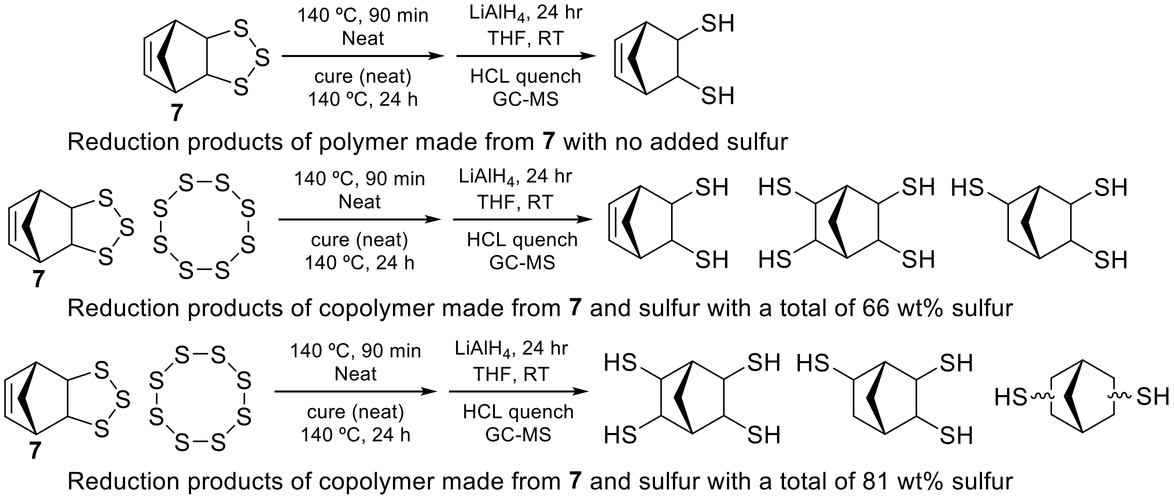

Figure S47: Overview of the reduction of polymers prepared from 7 and copolymerizations with sulfur

When the polymers made using trisulfide **7** were reduced, the GC-MS chromatograms were consistent with the FTIR data. When no sulfur was added, the major peak was a norbornene dithiol (top right in Figure S48). This peak indicated that the alkene in **7** remained largely intact and did not react. In contrast, the polymers made from **7** with added sulfur indicated the alkene reacted. Two samples of this type were reduced. One had a total of approximately 66 wt% sulfur while the other had approximately 81 wt% sulfur. In the sample with 66 wt% sulfur, the largest peak shifted to the expected tetrathiol (middle right in Figure S48). This indicated that a large portion of the alkenes had reacted, which is consistent with the IR spectrum of the polymer. This sample also showed a peak for a trithiol which could be produced when the tetrathiol is further reduced by lithium aluminum hydride (bottom, Figure S48). The peak for the dithiol with an alkene was still present, indicating that not all the alkenes had reacted. After reducing the sample with 81% sulfur, no alkenes were observed in the products. The results of this series of experiments suggest that **7** can undergo homopolymerization without significant reaction at the alkene. However, the copolymerization of **7** with sulfur causes reaction with the alkene. At 81 wt% total sulfur, all of the alkene was consumed.

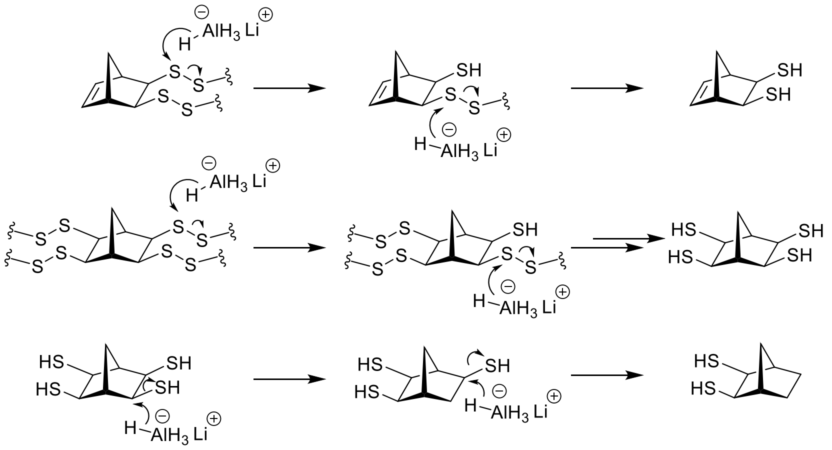

Figure S48: Proposed mechanism for the reduction of several potential polymers to give molecules observed in GC-MS.

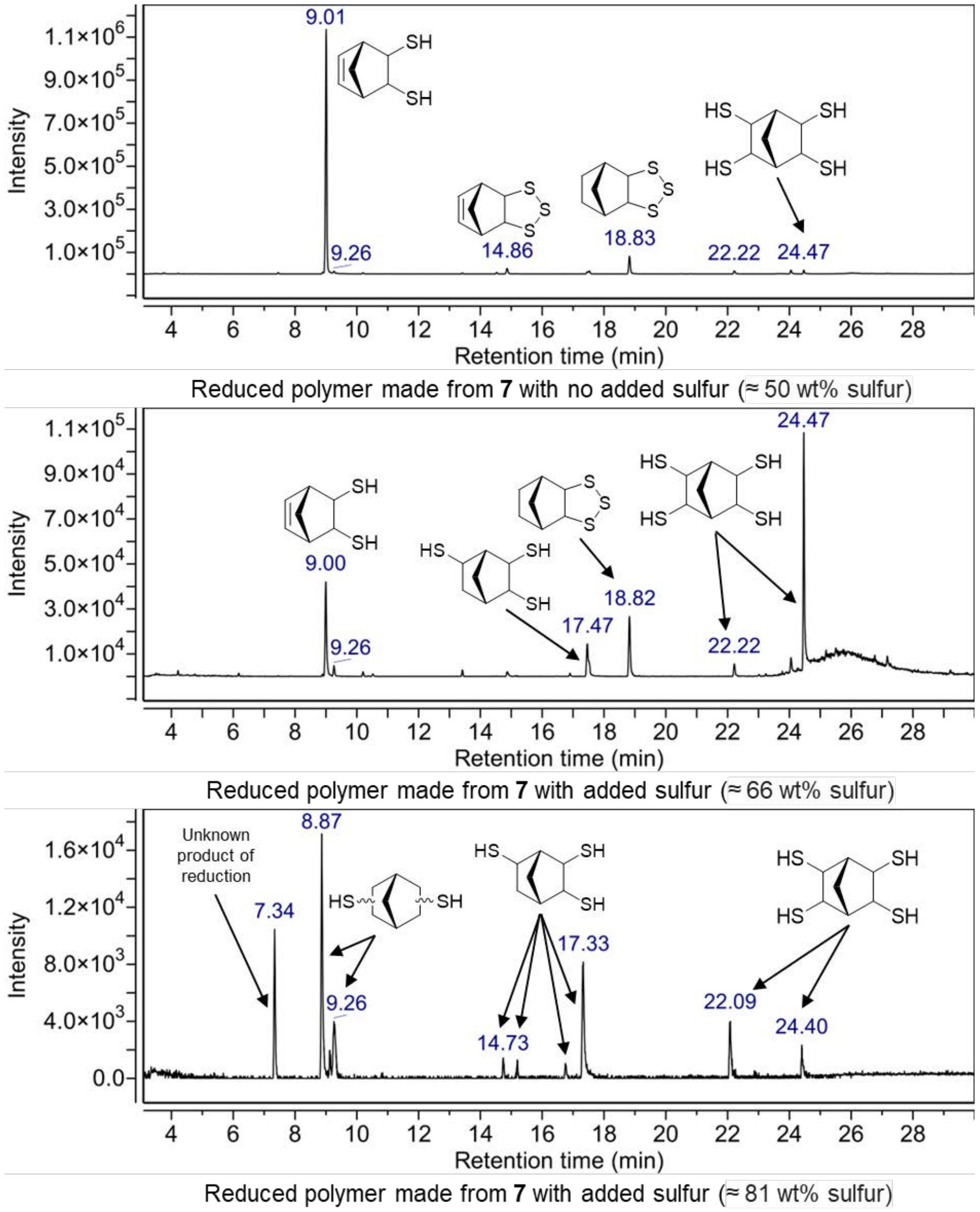

Figure S49: GC-MS chromatograms of the products formed after reducing polymers made from **7**. All polymers were prepared from a neat reaction.

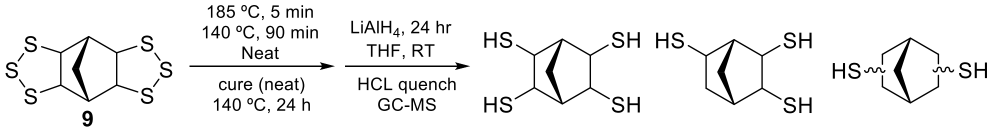

Reduction products of polymer made from **9** with no added sulfur

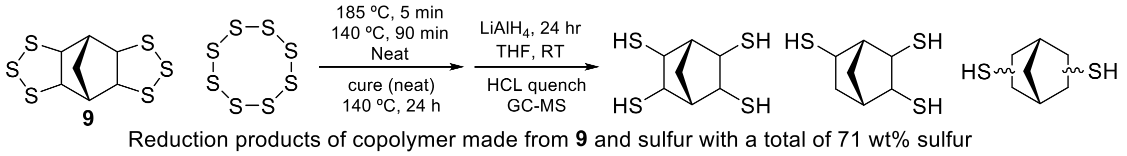

Reduction products of copolymer made from **9** and sulfur with a total of 71 wt% sulfur

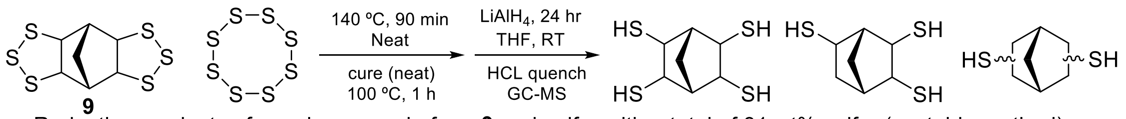

Reduction products of copolymer made from **9** and sulfur with a total of 81 wt% sulfur (castable method)

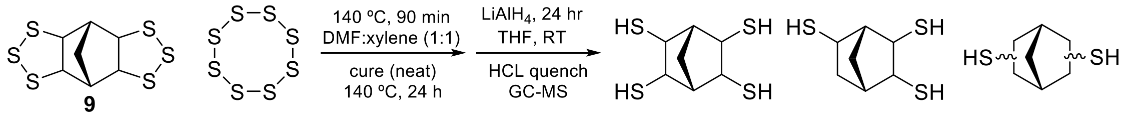

Reduction products of copolymer made from **9** and sulfur with a total of 71 wt% sulfur in a solvent of DMF:xylene (1:1)

Figure S50: Overview of the reduction of polymers prepared from **9** and copolymerizations with sulfur

Four samples made from bistrisulfide **9** were reduced with lithium aluminum hydride and the products were analyzed by GC-MS. Three of the samples were from neat reactions with differing amounts of sulfur and one was polymerized in a solvent system of 50-50 DMF-xylene. All samples showed the expected tetrathiol product. They also showed other products of over reduction. None of the chromatograms showed peaks for products containing alkenes, cyclopropane, or other products of rearrangement. The chromatograms are shown on the following page.

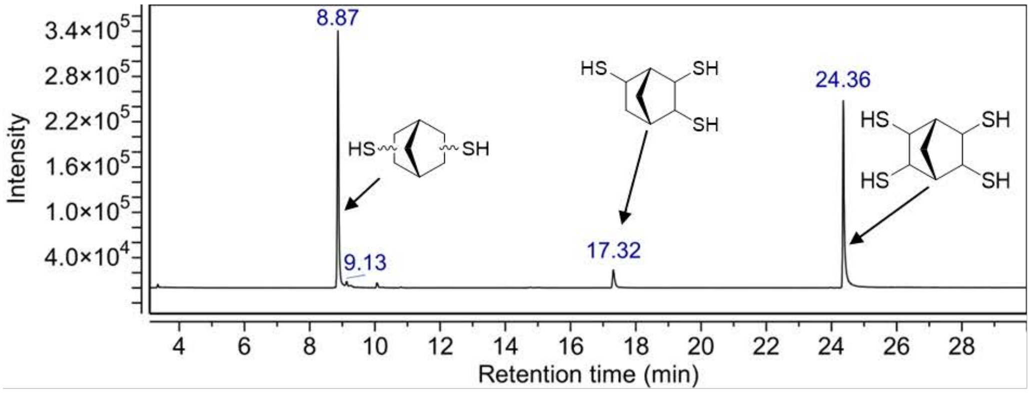

Reduced polymer made from **9** with no added sulfur (≈ 67 wt% sulfur)

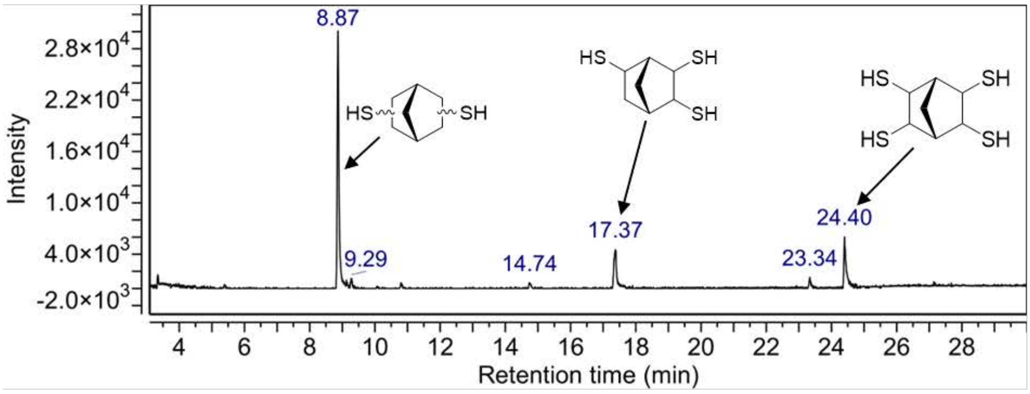

Reduced polymer made from **9** with added sulfur (≈ 71 wt% sulfur)

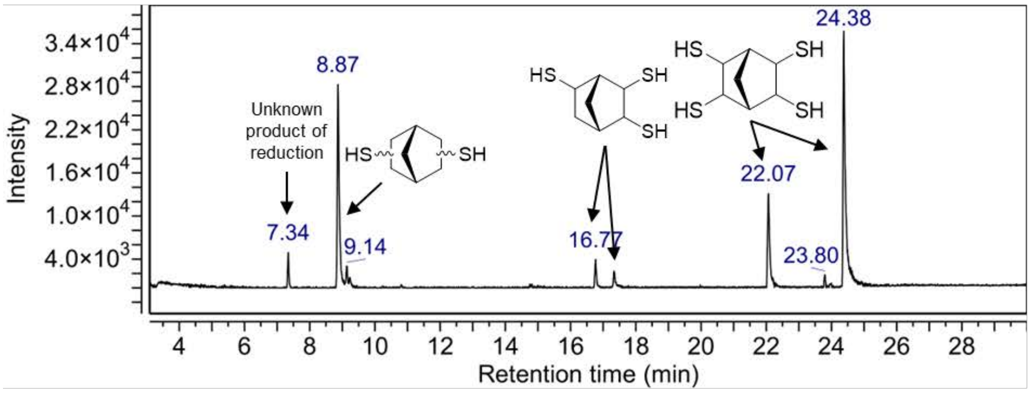

Reduced polymer made from **9** with added sulfur (≈ 81 % S polymer **1**)

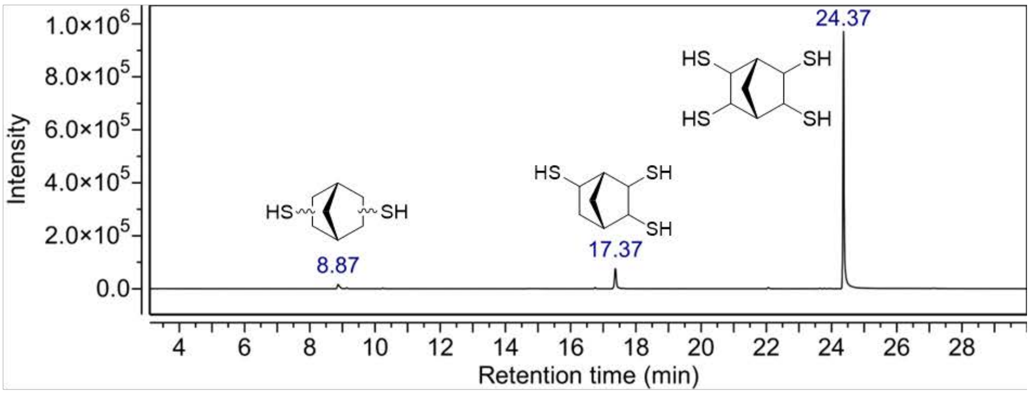

Reduced polymer made from **9** with added sulfur in solvent (≈ 71 wt% sulfur)

Figure S51: GC-MS chromatograms for the products formed after $LiAlH_4$ reduction of polymers made from bistrisulfide **9**. The last spectrum was for a polymer prepared in solution (50-50 DMF-xylene); all others were prepared neat.

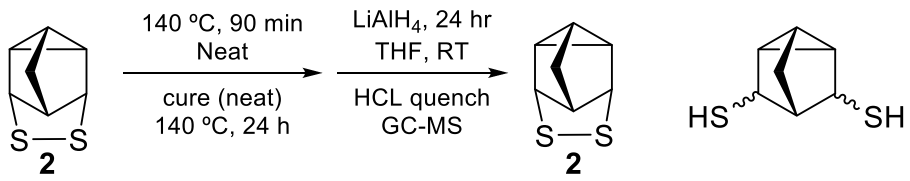

Reduction products of polymer made from 2 with no added sulfur

Figure S52: Overview of the reduction of polymers prepared from **2** and copolymerizations with sulfur

A polymer prepared from the rearranged disulfide **2** was reduced with lithium aluminum hydride. The polymer was prepared neat with no added sulfur. After reduction and extraction, there were three major peaks in the GC. Two peaks were isomers of a dithiol and the other peak corresponded to monomer **2**. These results indicate that the C-S stereochemistry of **2** can be inverted in the polymerization or reduction. These results also indicate that monomer **2** can be reformed and not be completely reduced by $LiAlH_4$ under these conditions (Figure S53, top). For reference, the overlayed chromatogram in Figure S53 (bottom) indicates that those di-thiol products of reduction can be resolved. This is important because it means that after reducing polymer made from **7** or **9**, it is clear that no cyclopropane-containing products are formed.

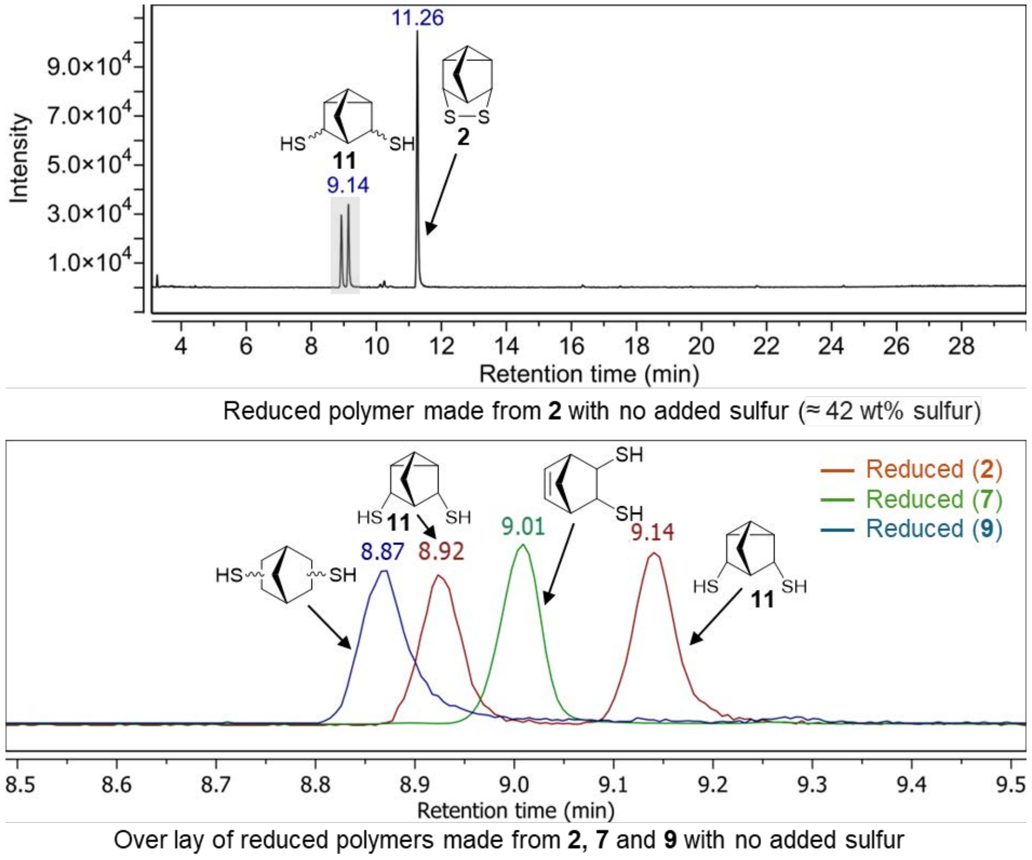

Figure S53: Top: GC-MS chromatogram of the products formed after reducing poly-**2** with $LiAlH_4$. Bottom: overlayed chromatograms of the products formed after reducing polymers **2**, **7** and **9** between a retention time of 8.5 minutes to 9.5 minutes.

Representative mass spectra for the reduction of polymers with $LiAlH_4$

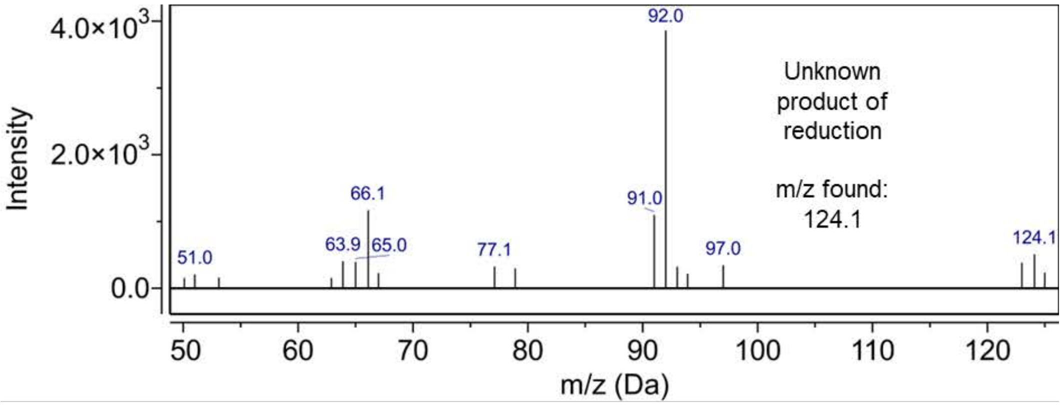

Mass spectrum for peak at 7.34 mins

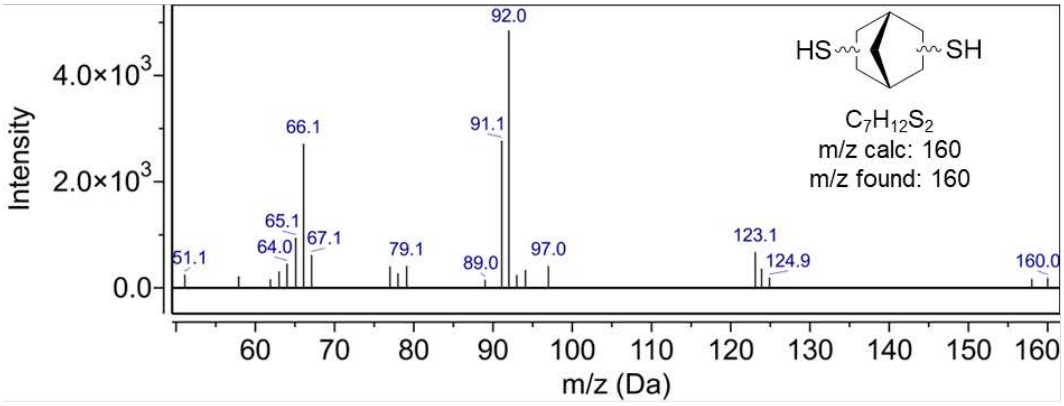

Mass spectrum for peak at 8.87 mins

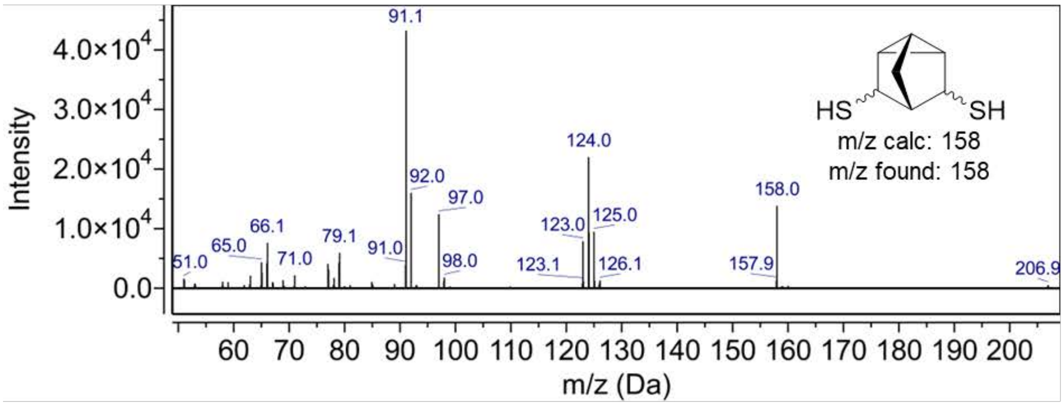

Mass spectrum for peak at 8.92 mins

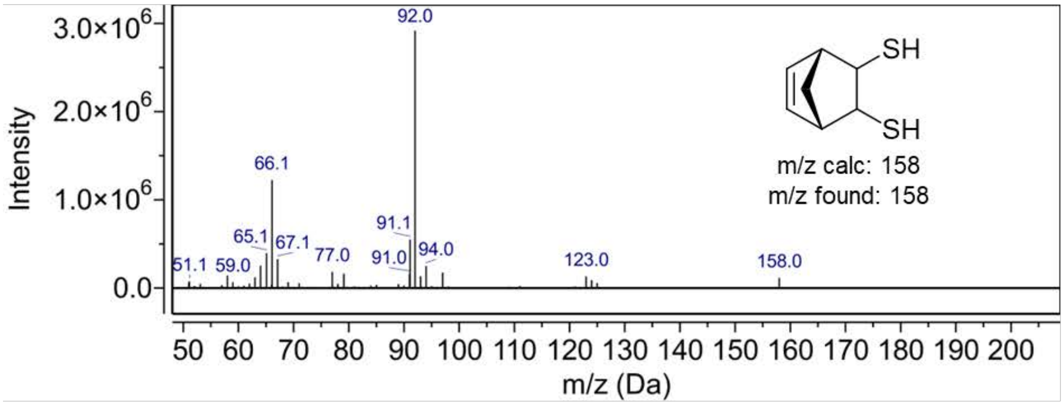

Mass spectrum for peak at 9.01 mins

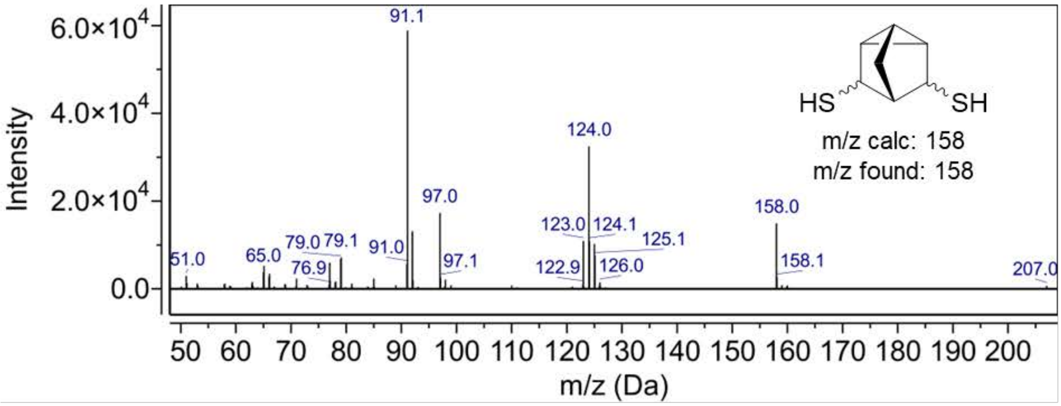

Mass spectrum for peak at 9.14 mins

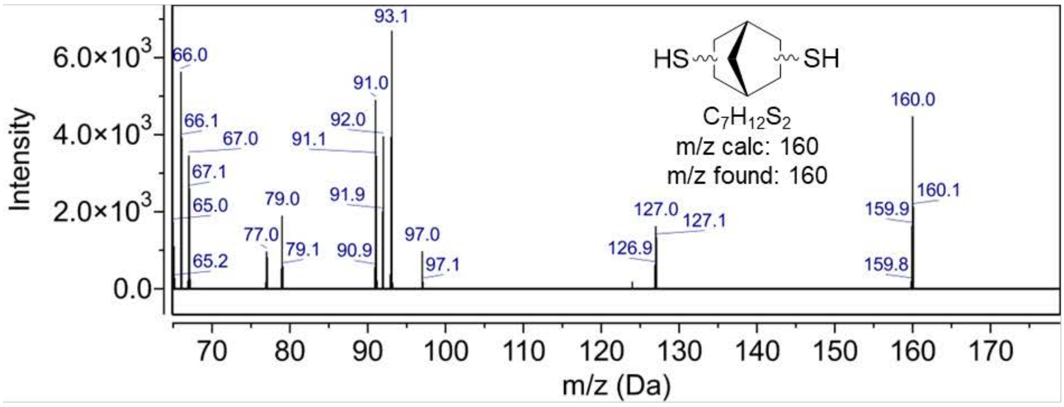

Mass spectrum for peak at 9.26 mins

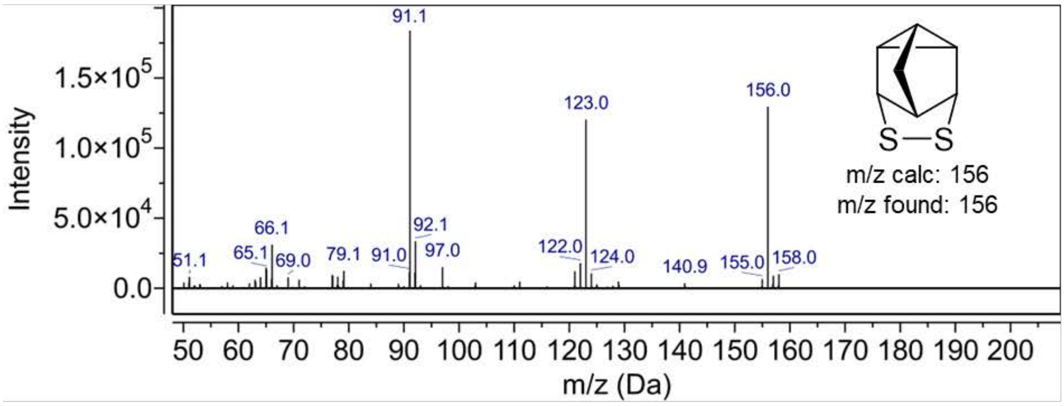

Mass spectrum for peak at 11.26 mins

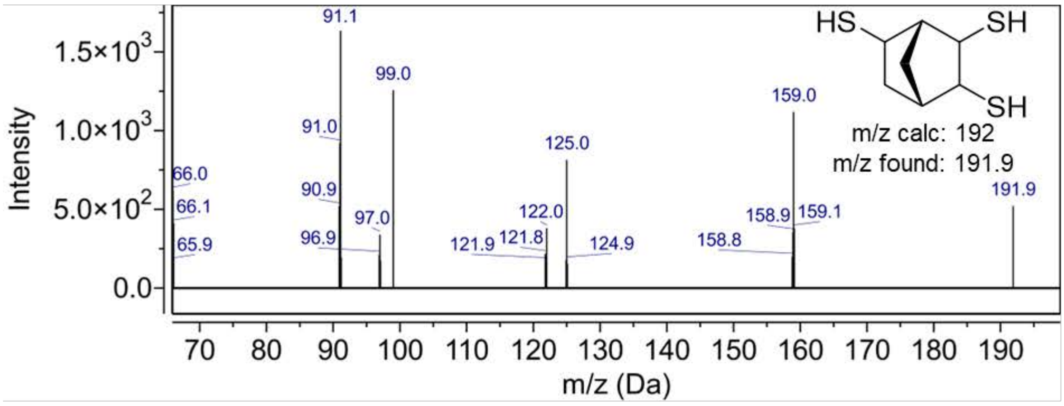

Mass spectrum for peak at 14.73 mins

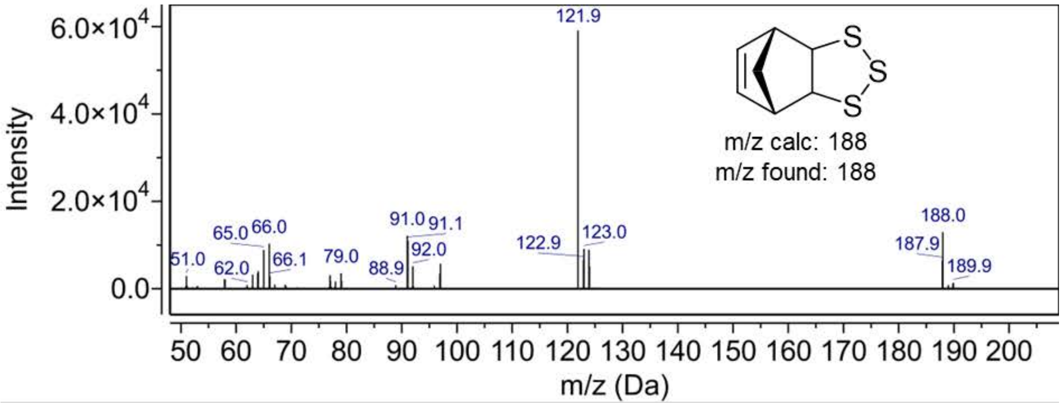

Mass spectrum for peak at 14.86 mins

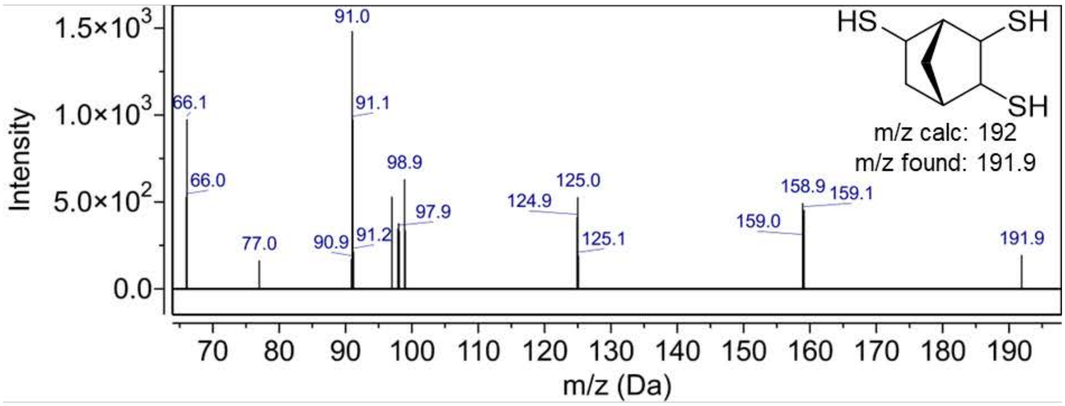

Mass spectrum for peak at 15.20 mins

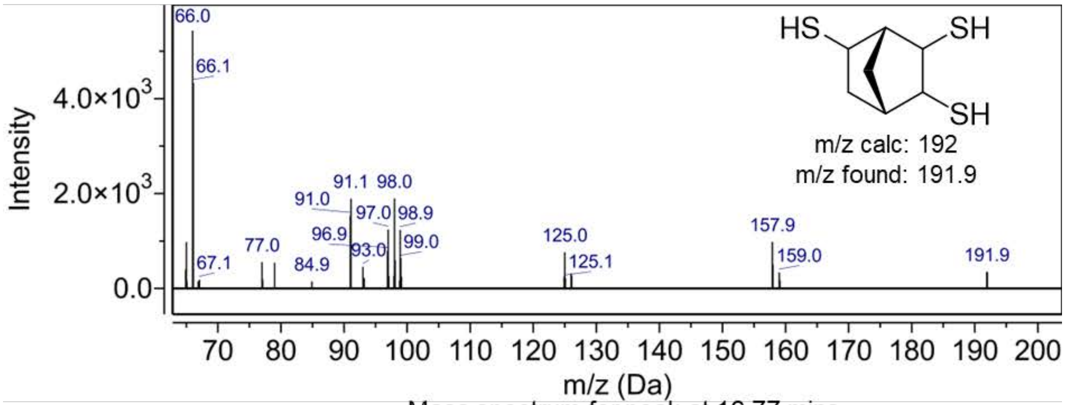

Mass spectrum for peak at 16.77 mins

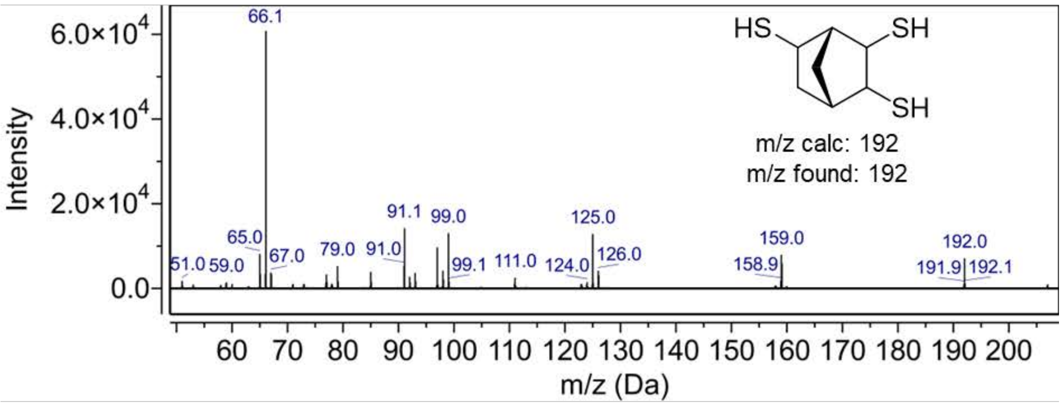

Mass spectrum for peak at 17.33 mins

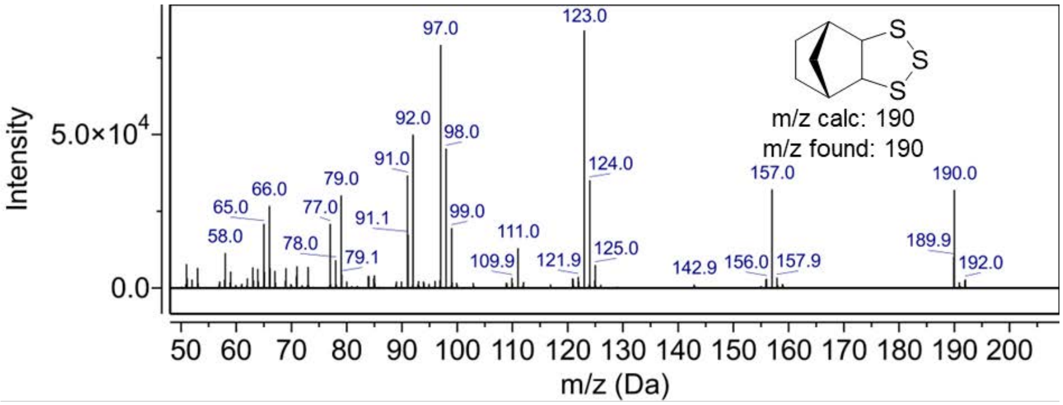

Mass spectrum for peak at 18.83 mins

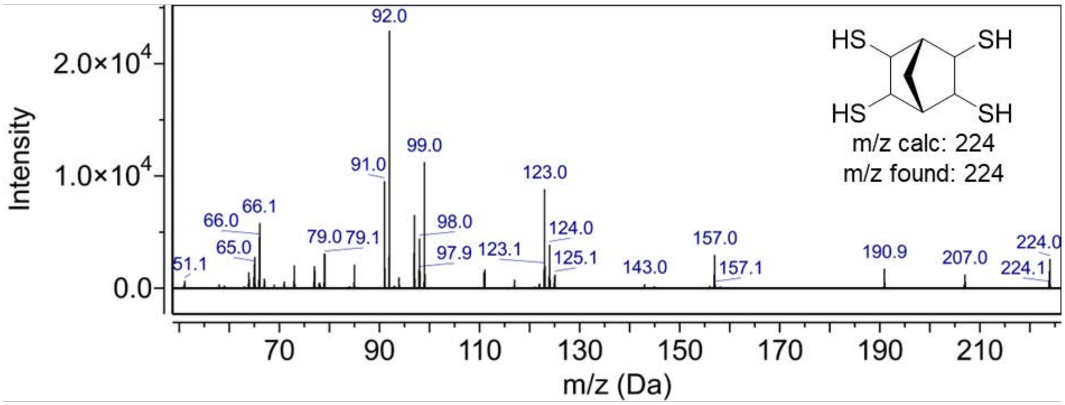

Mass spectrum for peak at 22.09 mins

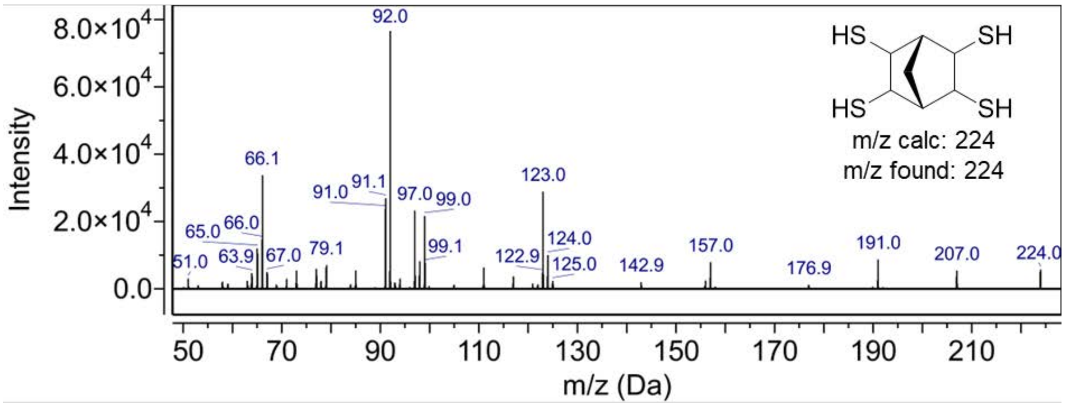

Mass spectrum for peak at 24.4 mins

Figure S54: Mass spectra for the range of products formed after reducing the polymers with $LiAlH_4$. The retention times for the GC-trace are also provided.

Differential scanning calorimetry of polymers made from monomers 2, 6-9

Each of the polymers made with cyclic sulfides were tested by differential scanning calorimetry (DSC) to measure the glass transition temperatures or any other thermal properties of relevance (e.g. melting transitions of unreacted monomers or sulfur). The samples were analyzed at the ANFF Queensland node using a Mettler Toledo DSC. Several of the samples were analyzed using a TA 2920 DSC. All samples were run using the same method. The samples were first heated from room temperature to 100 °C at 25 °C/min and held for 1 minute. This step was to remove any thermal history in the samples. The temperature was then decreased to 0 °C at 25 °C/min where it was held again for 1 minute. The temperature was then increased to 200 °C at 25 °C/min. All samples were under a constant stream of nitrogen. The samples that were run on the TA 2920 DSC followed the same method, but the samples were only cooled to 40 degrees. The glass transition temperatures of the polymer made from trisulfide **7** were very high, ranging from 148 °C to 196 °C. Interestingly, the glass transition temperature increased when **7** was polymerized with additional sulfur. Usually, the glass transition temperature of sulfur polymers decreases with increasing sulfur content.[8-10] It is possible that this was due to the alkene reacting with additional sulfur, leading to more crosslinking and a higher glass transition temperature. No samples had any crystalline sulfur melting peaks.

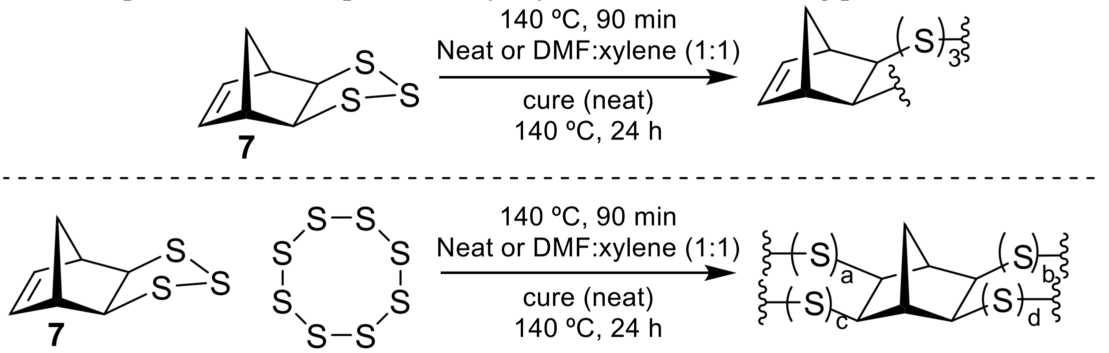

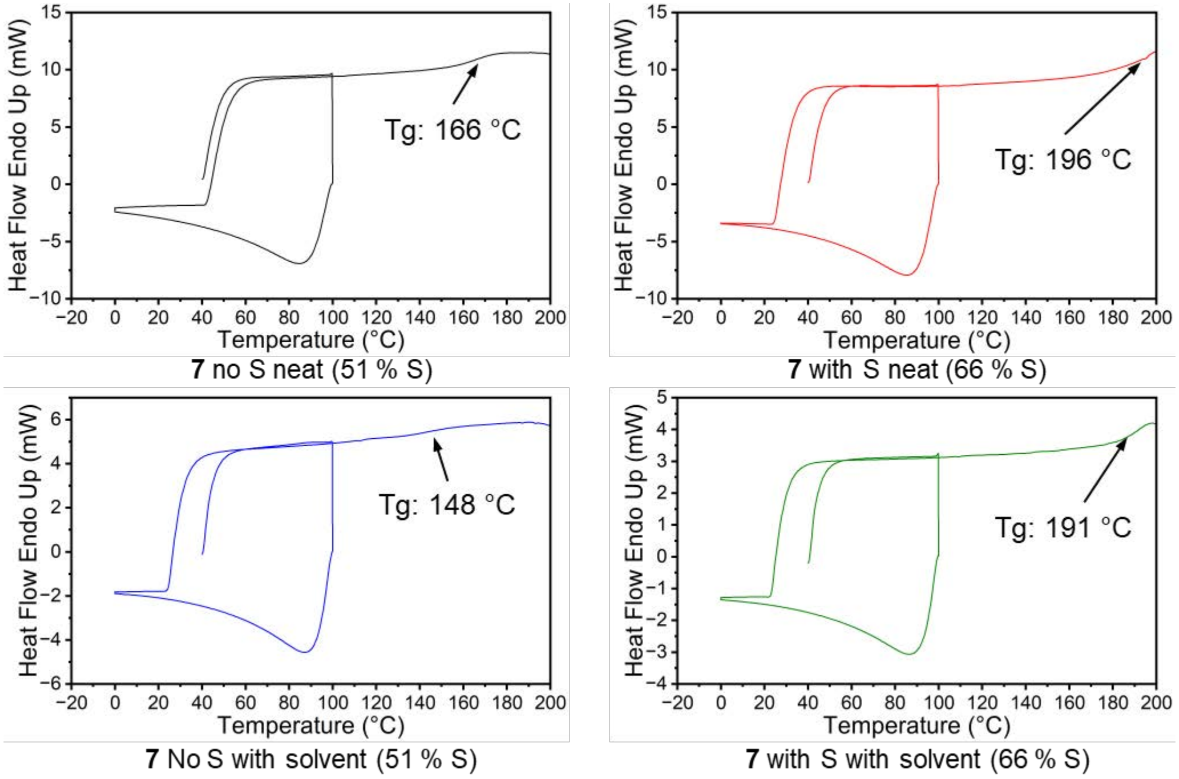

Figure S55: Differential scanning calorimetry thermogram of polymers made from trisulfide **7**.

The polymers made from the mixture of monomers **6**, **7**, and **8** had very similar thermograms to the polymer prepared from **7**. Just like the samples made with only **7**, the polymers made by reaction of **6**, **7**, and **8** with added sulfur had a greater glass transition temperature. The samples made in solvent had a slightly decreased glass transition temperature than those made neat.

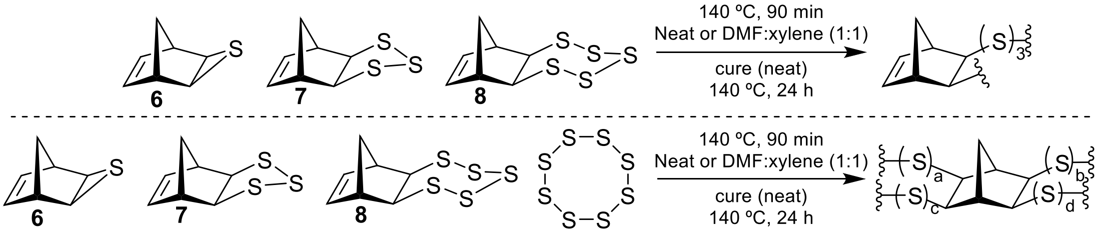

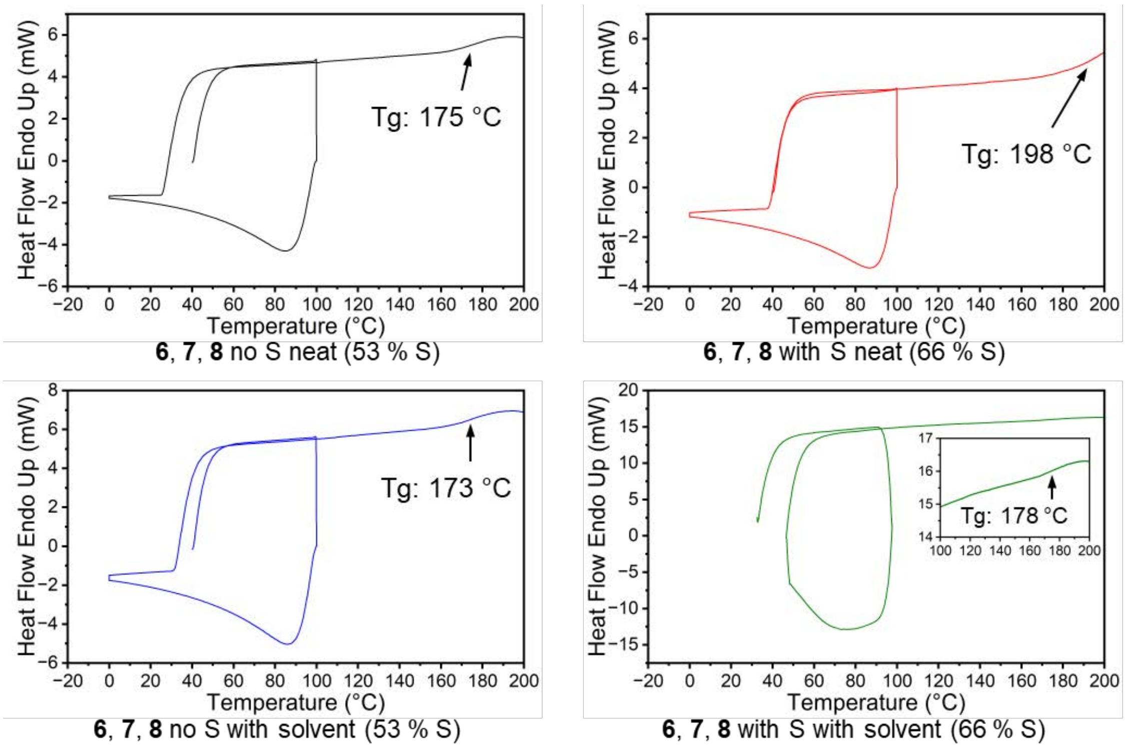

Figure S56: Differential scanning calorimetry thermogram of polymers made from a combination of (**6**, **7**, **8**) in a 24:100:40 ratio. The samples labelled with sulfur had a total sulfur content of 66%.

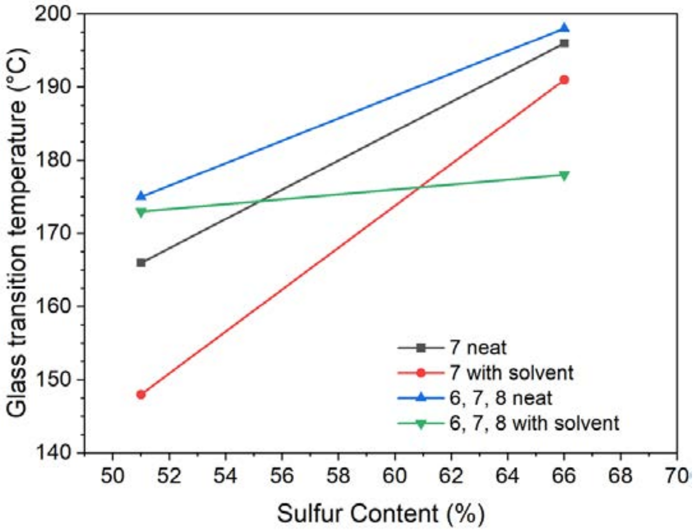

Figure S57: Glass transition temperature of polymers made from **7** or a combination of **6**, **7**, **8** in a ratio of 24:100:40.

The polymers made from bistrisulfide **9** had very high glass transition temperatures. For these polymers, a decrease in glass transition temperature was observed with increasing sulfur content. The samples made in a solvent of 50-50 DMF-Xylene had a slightly decreased glass transition temperature than those made neat.

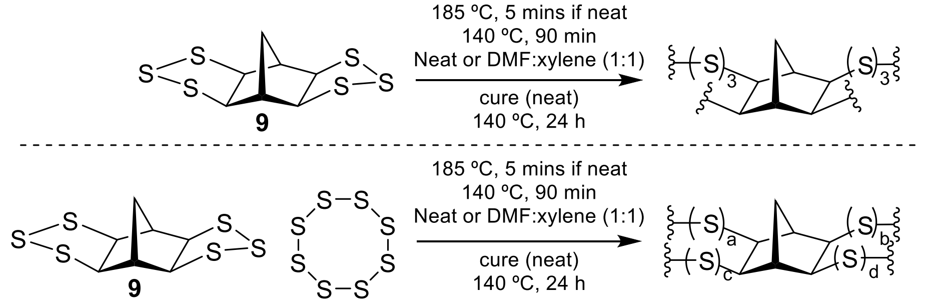

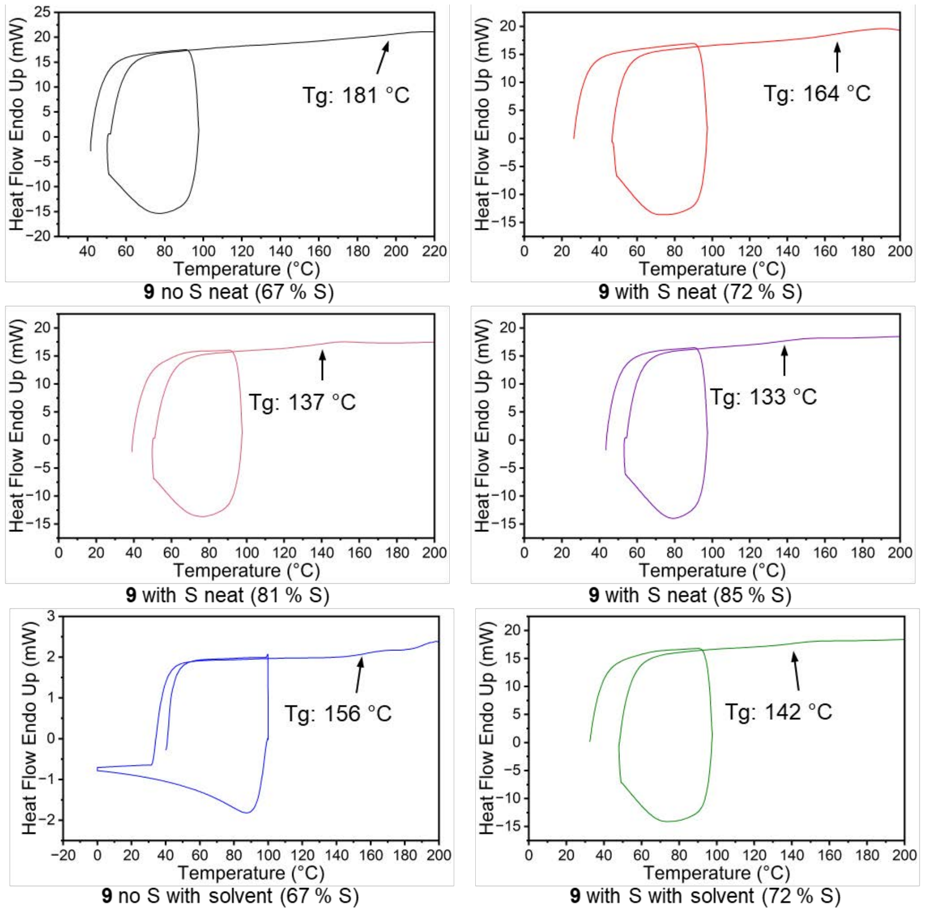

Figure S58: Differential scanning calorimetry thermogram of polymers made from bistrisulfide **9**.

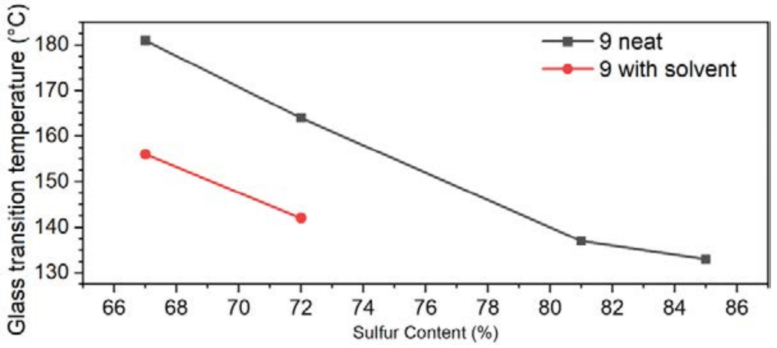

Figure S59: Glass transition temperature of polymers made from **9**.

The above DSC thermograms were obtained using a TA 2920 DSC with a heating rate of 25 °C/min. This heating rate is likely too high and could lead to an overestimation of the glass transition temperature due to thermal lag. To confirm the glass transition temperature of the polymer **1** at a composition of 81 % sulfur, it was repeated with a newer TA Q20 DSC at a more moderate heating rate of 10 °C/minute. Two full heating ramps were obtained between -60 °C and 200 °C. A third ramp was also obtained at 20 °C/minute to show the apparent change in glass transition temperature at a higher heating rate. The measured glass transition temperature increased by 7 °C with the higher heating rate. Thermal lag is a known issue with DSC so the glass transition temperature was also confirmed using DMTA which can be found in the next section. Along with the glass transition temperature, another transition was observed in the heating cycle of the DSC at 163 °C (with a heating rate of 10 °C/min). This transition was not observed in cooling.

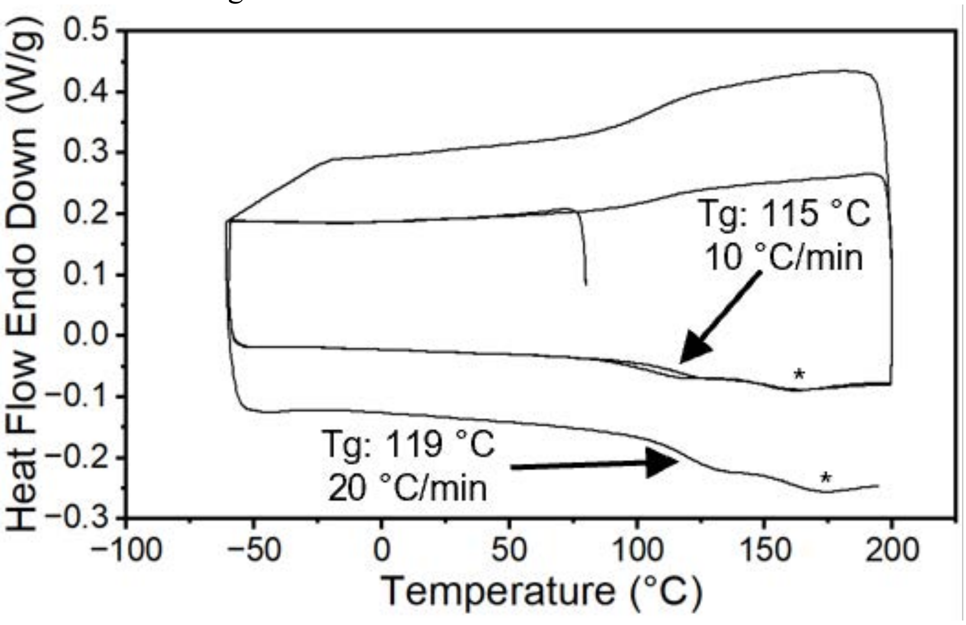

Figure S60: Differential scanning calorimetry thermogram for polymer **1** with a sulfur composition of 81 % using a TA Q20 DSC. Two ramps at 10 °C/min and one at 20 °C/min are displayed. The thermal transition labelled with a star is only observed during heating and occurs at 163 °C and 174 °C with a heating rate of 10 °C/min and 20 °C/min respectively.

The polymers prepared from the **2** had a very different DSC thermogram to all the other polymers. All the samples showed a melting peak in the first heating run. This potentially indicated that there was some unreacted monomer in these samples as **2** melts around this temperature. The samples also did not show a clear glass transition temperature in the with S neat or No S with solvent samples. The other two samples had a glass transition temperature around 122 °C.

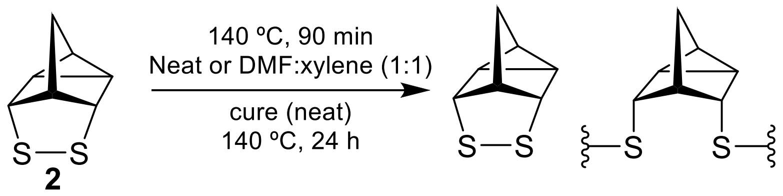

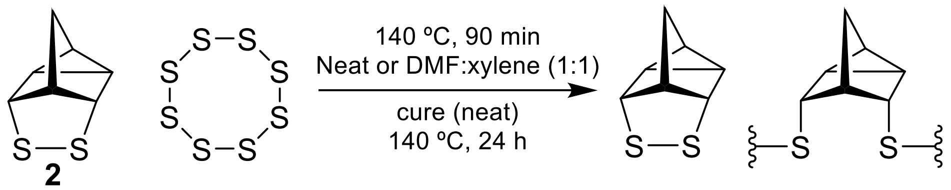

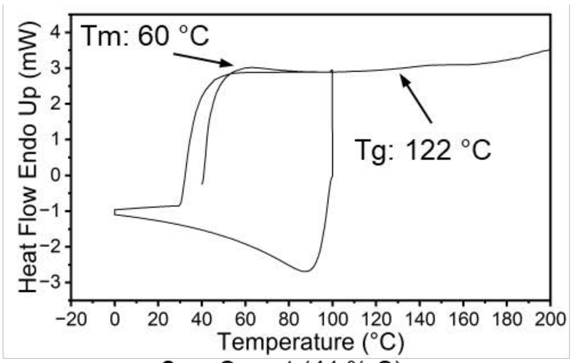

2 no S neat (41 % S)

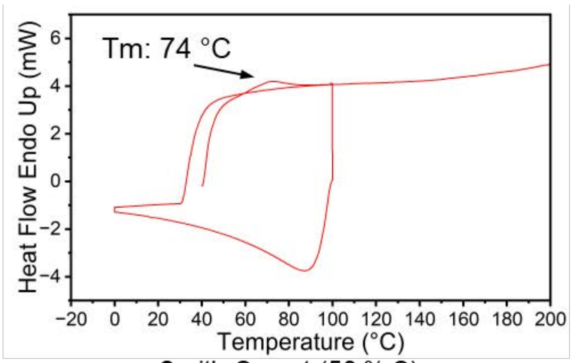

2 with S neat (58 % S)

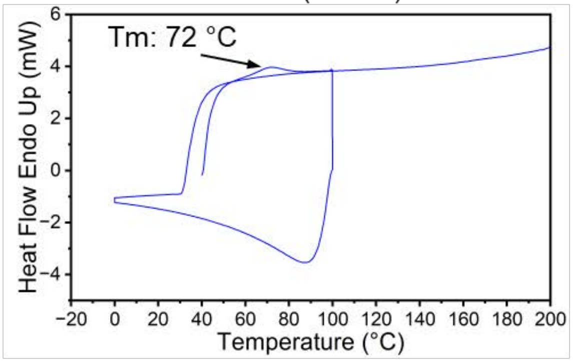

2 no S with solvent (41 % S)

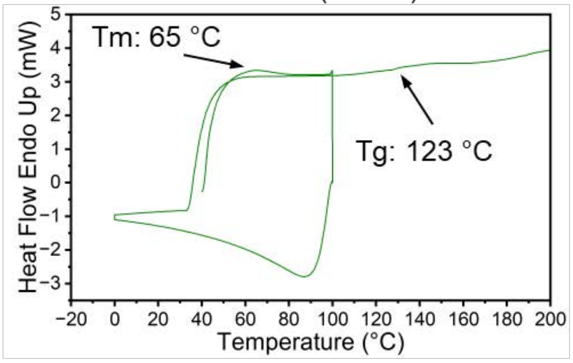

2 with S with solvent (58 % S)

Figure S61: Differential scanning calorimetry thermogram of polymers made from disulfide **2**.

Dynamic mechanical thermal analysis of polymer 1

The glass transition temperature of polymer **1** prepared from **9** (81% sulfur by mass) was also assessed using dynamic mechanical thermal analysis (DMTA). DMTA analysis was performed using a TA DMA Q800. Standard temperature sweep method used a temperature range of room temperature (19.5 °C) to 200 °C with a ramp rate of 3 °C/min. A preload force of 0.01 N was applied with and amplitude of 1 μm and a frequency of 1 Hz. The tension clamp was used with a rectangular sample with dimensions of approximately 15 mm x 10 mm x 1 mm. Polymer **1** had a broad glass transition which was very clear in the DMTA thermogram. The onset of the storage modulus was 133.09 °C at 476.4 MPa. The loss modulus had a peak at 136.11 °C and the tan delta had a peak at 153.41 °C. These results are complementary to the DSC measurements and provide a similar glass transition temperature.

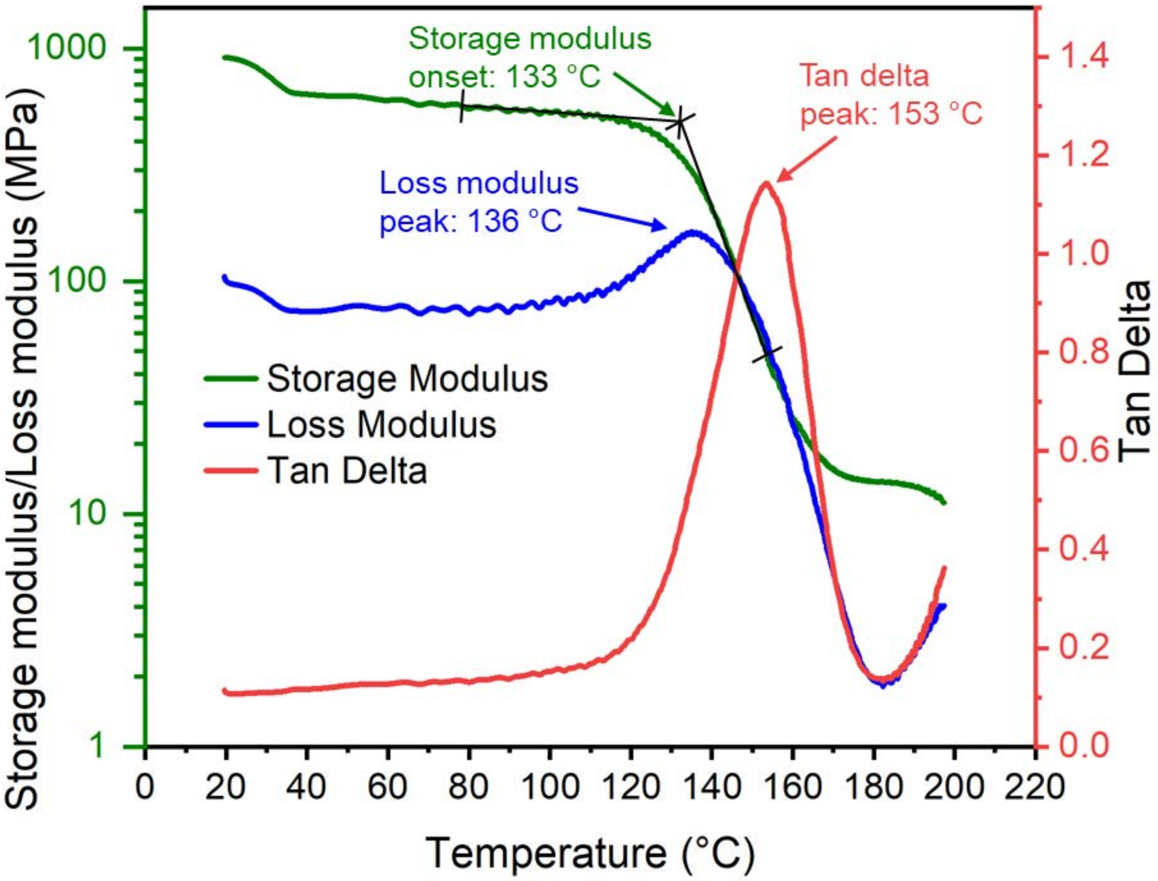

Figure S62: DMTA thermogram of a sample of polymer **1** (81% sulfur) tested using a TA DMA Q800 with the tension clamp.

Solid state NMR spectra of polymer 1 made from bistrisulfide 9

To understand the polymer structure further, molecule **9** and two polymers with different sulfur contents were analyzed using carbon-13 cross polarization solid state NMR spectroscopy. Like in the solution ^{13}C NMR spectrum, **9** had three peaks at around 70 ppm, 47 ppm and 31 ppm. In the polymer samples, these peaks were still observed but they were significantly broader and shifted to a downfield chemical shift. No peaks were observed below 25 ppm apart from a spinning side band. The peaks from cyclopropane are usually observed in this region, providing evidence that no rearrangement occurred. For the acquisition of these spectra, the samples were ground into a powder and packed into a 4 mm sample tube. A 400 MHz NMR spectrometer operating at 100 MHz was used with a magic angle spin rate of 5 kHz. The spectrometer was calibrated using the 29.5 ppm peak of adamantane. All samples were analyzed by cross polarization magic angle spinning solid state NMR spectroscopy.

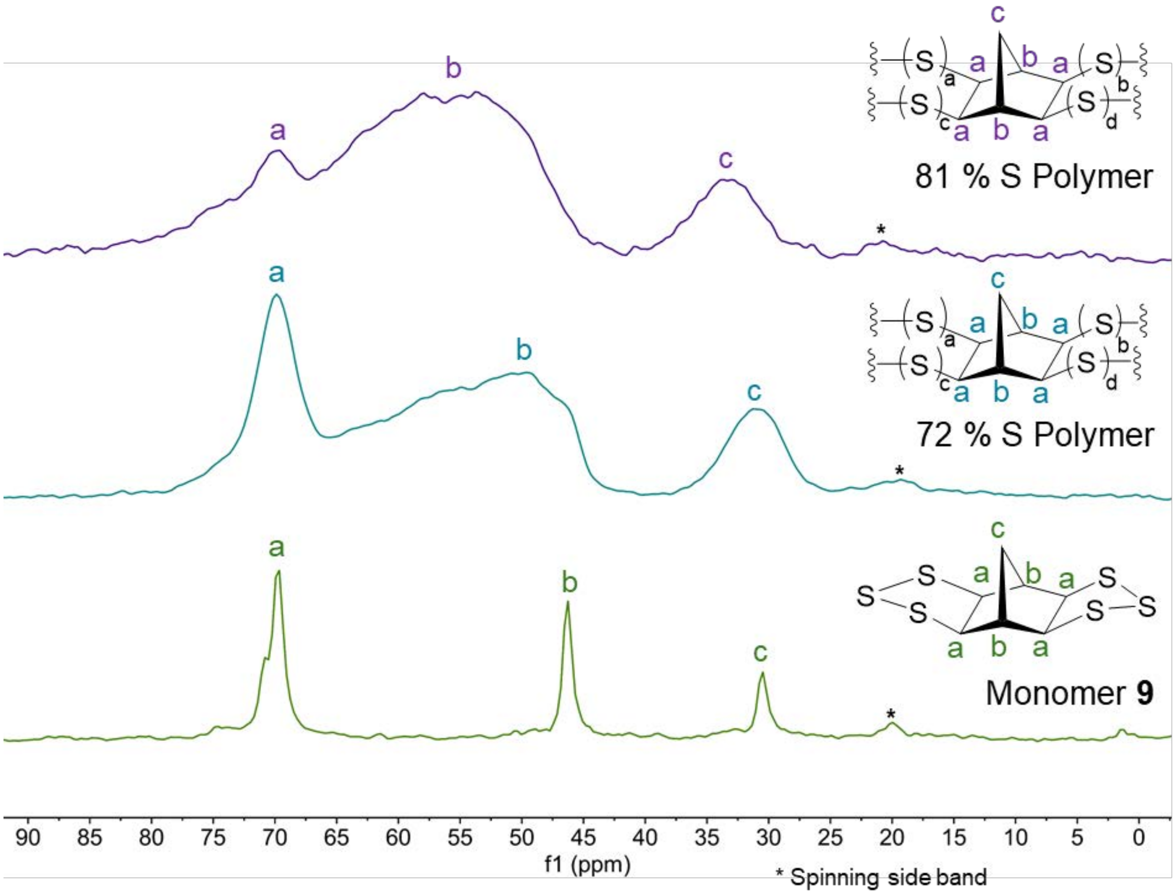

Figure S63: Cross polarization, magic angle spinning, solid state NMR spectra of monomer **9** and two samples of polymer **1** with different sulfur content.

Raman spectroscopy of polymer 1

Raman spectroscopy can be used to estimate the number of sulfur atoms that link each monomer[11]. It can also be used to detect crystalline sulfur in the sample[12]. To analyze the sulfur region of the Raman spectrum of polymer **1** (made from **9**), the spectrum was compared to reference samples of elemental sulfur and a disulfide, trisulfide and tetrasulfide. Raman spectra were collected using a Witec alpha 300R Raman microscope at an excitation laser wavelength of 785 nm with a 40X objective. A 600 groves/mm grating was used which gives a spectral resolution of approximately 3 to 4 cm^{-1}. The x-axis was calibrated to the 520.6 cm^{-1} peak for silicon. Approximately 10 samples were taken for each sample with an integration time per spectra between 10 and 20 seconds with 2 to 3 accumulations. When compared with monomer **9**, polymer **1** had broader peaks. There was significant fluorescence, which has been reported for sulfur polymers in literature.[11] The fluorescence was much more pronounced in the samples which were synthesized in solvent. Nonetheless, all of the polymer samples of **1** had the same peaks. None of the polymer samples had the characteristic peaks for crystalline sulfur, indicating that there was no unreacted sulfur in the polymer matrix. The broad peaks in the polymer between 400-500 cm^{-1} suggest a mixture of sulfur ranks, with the major peaks corresponding to trisulfide and tetrasulfide units. The shift to lower wavenumber also suggests the cyclic trisulfide in monomer **9** was opened in the polymerization, but some cyclic trisulfide end groups may still be present in the polymer. A mixture of sulfur ranks is expected for the series of S-S metathesis events that occur in the polymerization process.

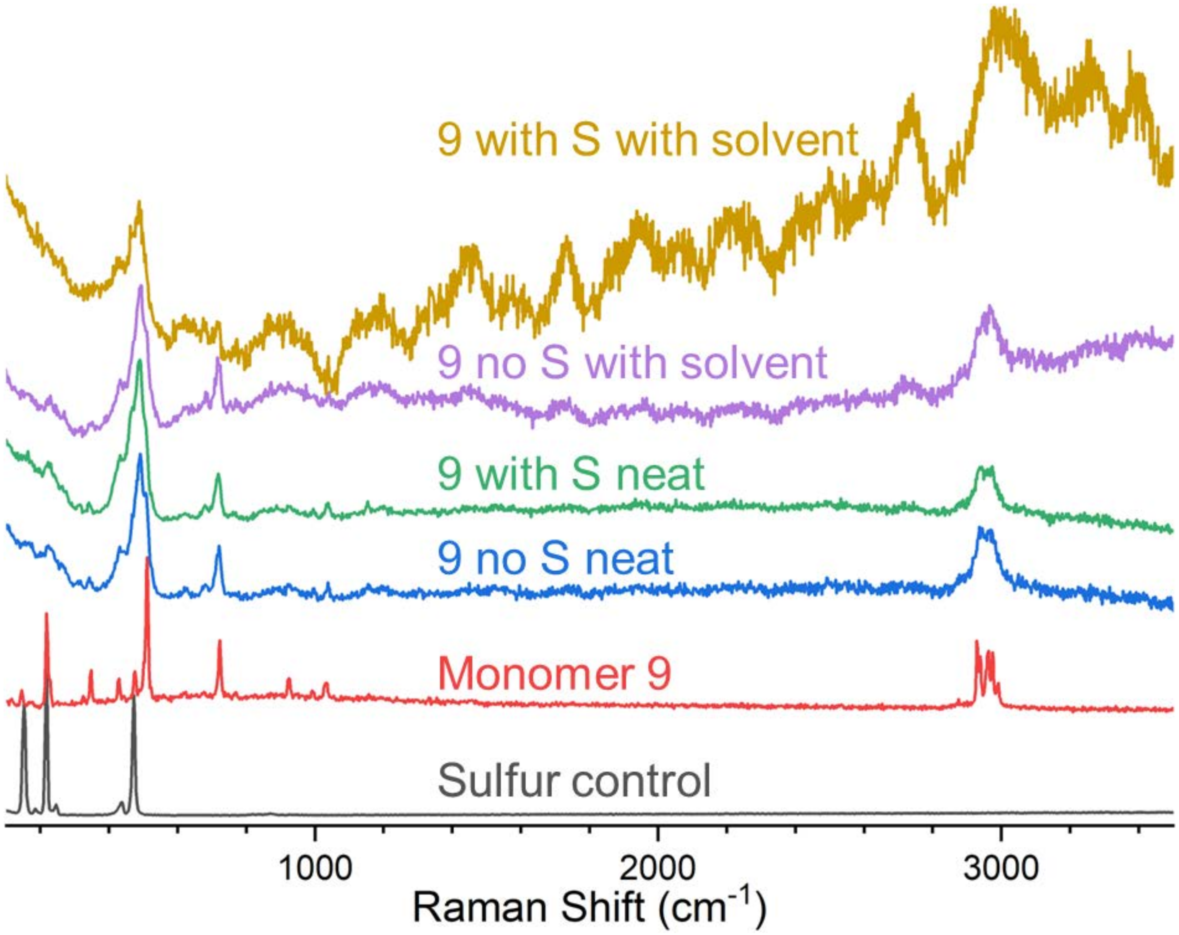

Figure S64: Raman spectra for a sulfur control, the monomer bistrisulfide **9** and polymers made using **9**.

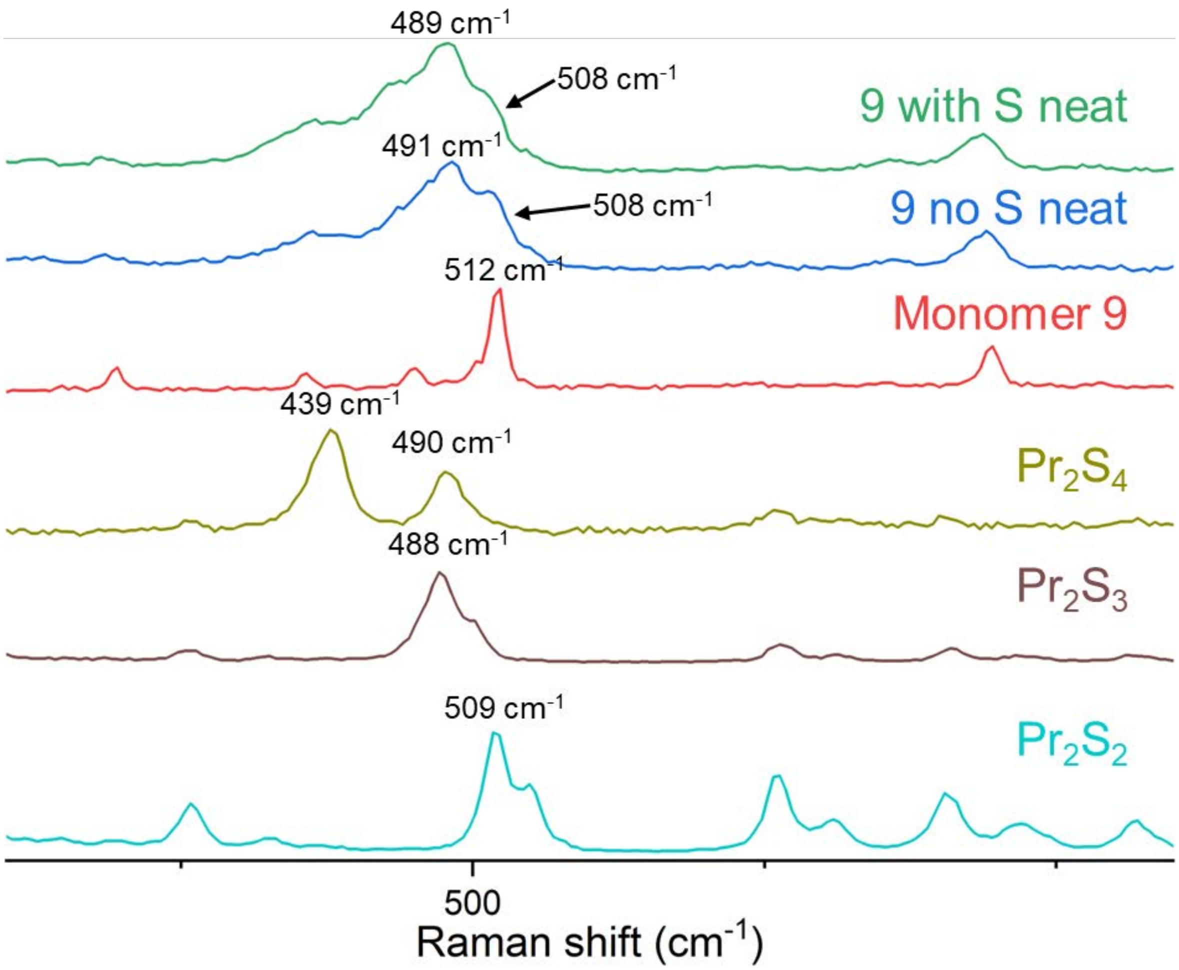

Figure S65: Raman spectra of disulfide, trisulfide and tetrasulfide reference compounds, bistrisulfide monomer **9** and polymer **1** made from **9**.

Thermal depolymerization of polymer 1

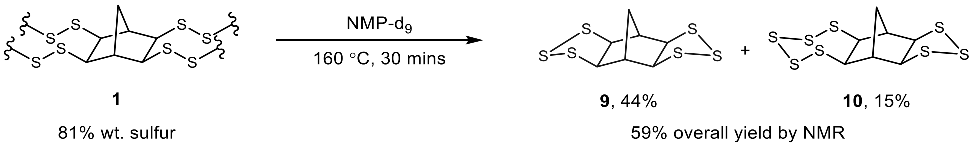

Crushed polymer **1** (15.9 mg, ~1 mm diameter) and deuterated NMP (1 mL) were stirred together at 160 °C for 30 mins. After cooling down to room temperature, trichloroethylene (11.9 mg) was added to the homogenous solution as an internal standard. An aliquot was removed and analyzed by NMR in $CDCl_3$. The overall yield was determined to be 59% by NMR (monomer **9**, 44%; monomer **10**, 15%), based on relative integrations and comparison to the internal standard.

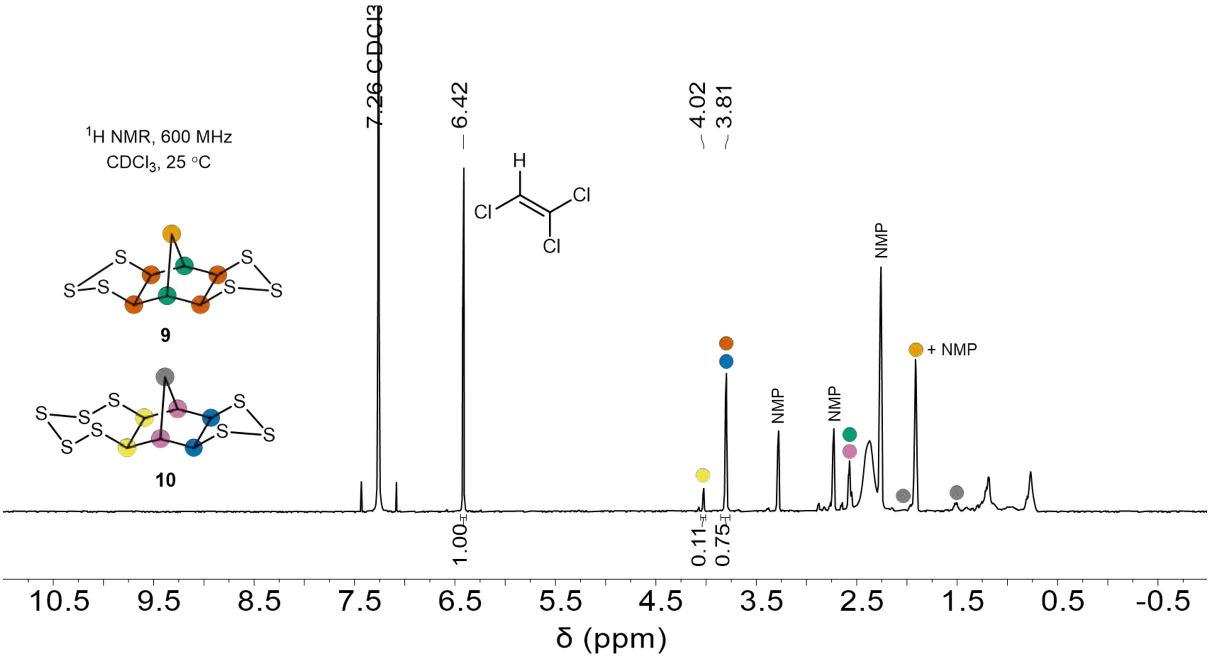

Figure S66: ^{1}H NMR in $CDCl_3$ of the thermal depolymerization of polymer **1**. The internal standard peak and the C-H signals of monomers **9** and **10** that were used to calculate the yield have been integrated.

Potassium thioacetate catalyzed depolymerization of polymer 1

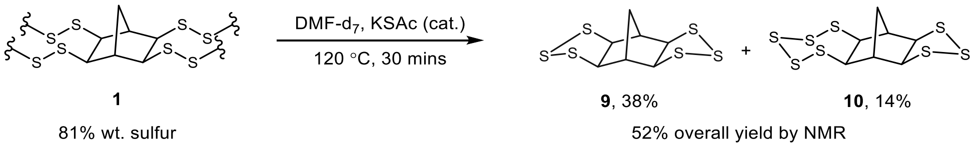

Crushed polymer **1** (14.2 mg, ~1 mm diameter), deuterated DMF (1 mL), and KSAc (1 mg) were stirred together at 120 °C for 30 mins forming a homogenous solution. After cooling down to room temperature, which results in precipitate formation, trichloroethylene (11.9 mg) was added as an internal standard. An aliquot was immediately removed and analyzed by NMR in $CDCl_3$. The overall yield was determined to be 52% by NMR (monomer **9**, 38%; monomer **10**, 14%).

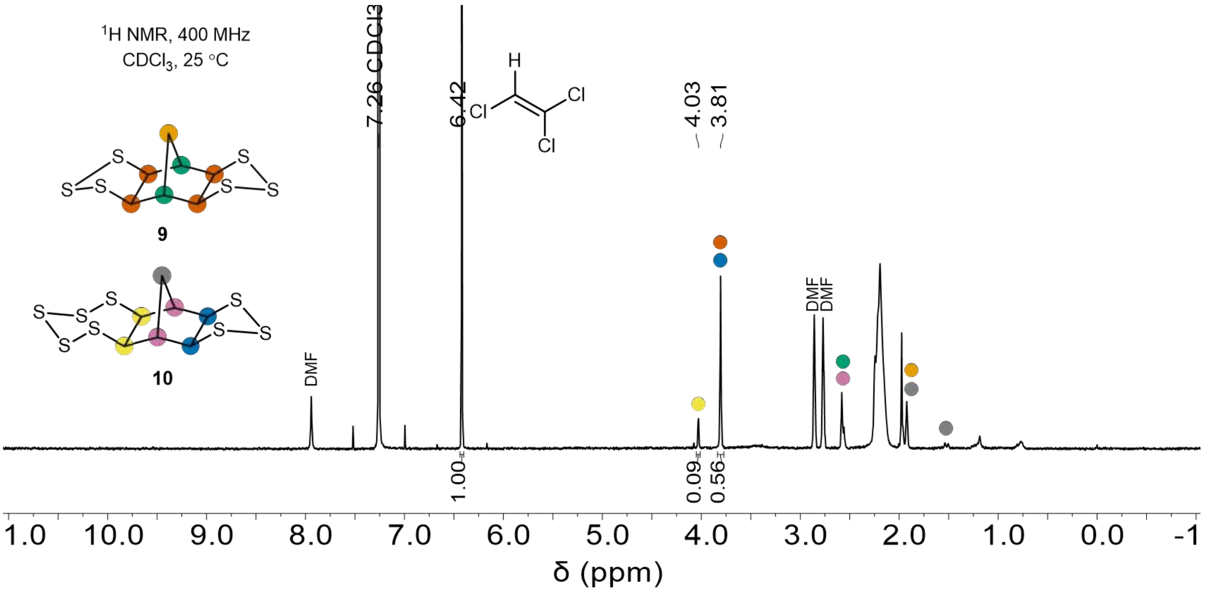

Figure S67: ^{1}H NMR in $CDCl_3$ of the KSAc catalyzed depolymerization of polymer **1**. The internal standard peak and the C-H signals of monomers **9** and **10** that were used to calculate the yield have been integrated.

Infrared transparency of polymer 1 at a range of thicknesses

The infrared transparency over the mid (3 µm – 5 µm) and long-wave 7 µm – 14 µm) infrared regions of polymer **1** was investigated to assess its potential in thermal imaging applications. A 1.62 mm thick, 13 mm diameter window of polymer **1** (81% sulfur) was cast into a silicone mold using the standard method described earlier. Its surfaces were polished using micromesh sandpaper ranging from 1500 to 12000 grit. After polishing, the infrared spectrum was analyzed using a Bruker Vertex 80 V. Images through the window were also taken with a standard camera and with a FLIR E6 thermal camera. The FLIR E6 used wavelengths ranging from 7.5 µm to 13 µm (LWIR). To reduce the thickness of the window, one side was lightly sanded using the 1500 grit micromesh sandpaper and then polished again. This process reduced the thickness of the window by approximately 50-150 µm with exact thickness measured by calipers. After every reduction in thickness, the polymer window was analyzed using the same method as the initial window. A total of 16 thickness were tested, ranging from 1.62 mm to 0.15 mm. The results are shown below.

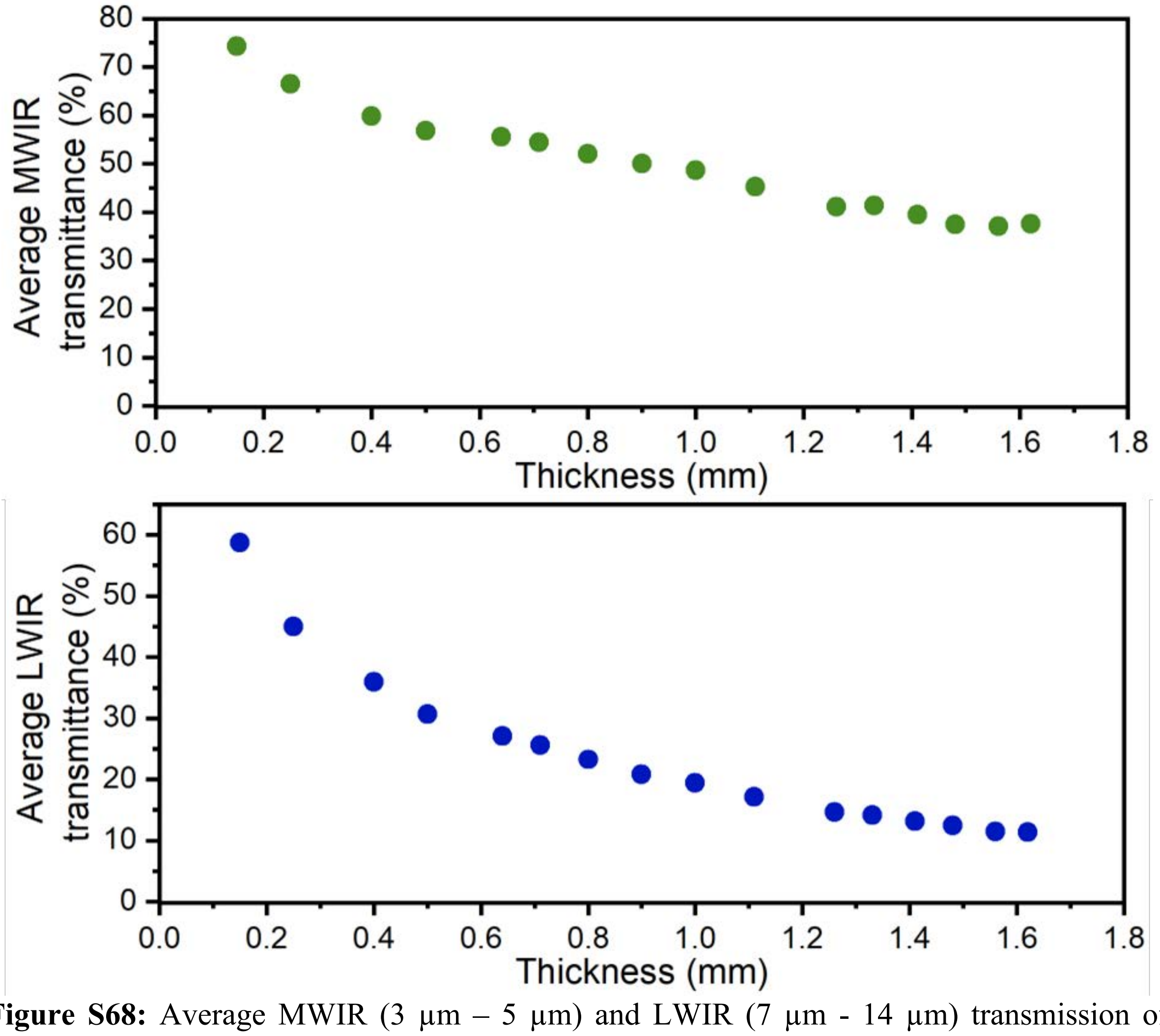

Figure S68: Average MWIR (3 µm – 5 µm) and LWIR (7 µm - 14 µm) transmission of polymer **1** (81% sulfur) obtained by integrating the FTIR transmission spectrum for windows of each thickness and dividing by the wavelength range.

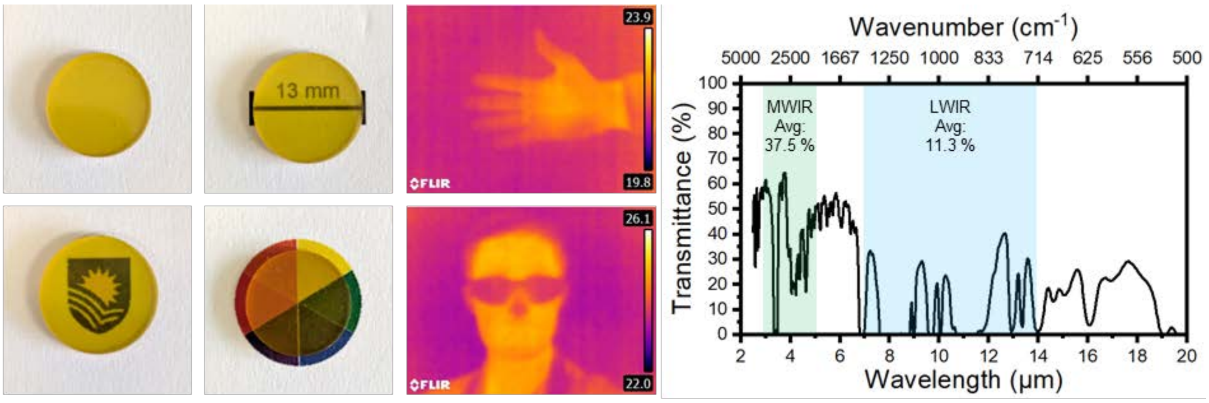

1.62 mm thick

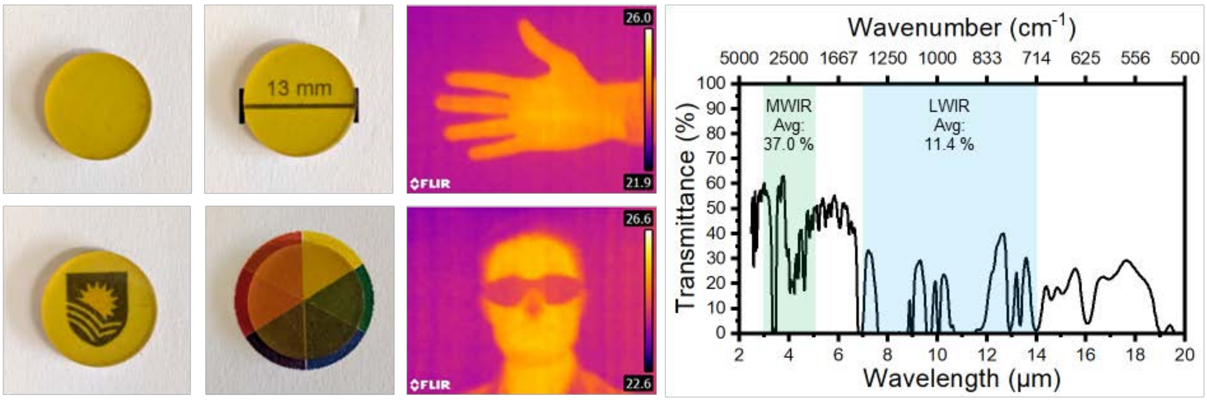

1.56 mm thick

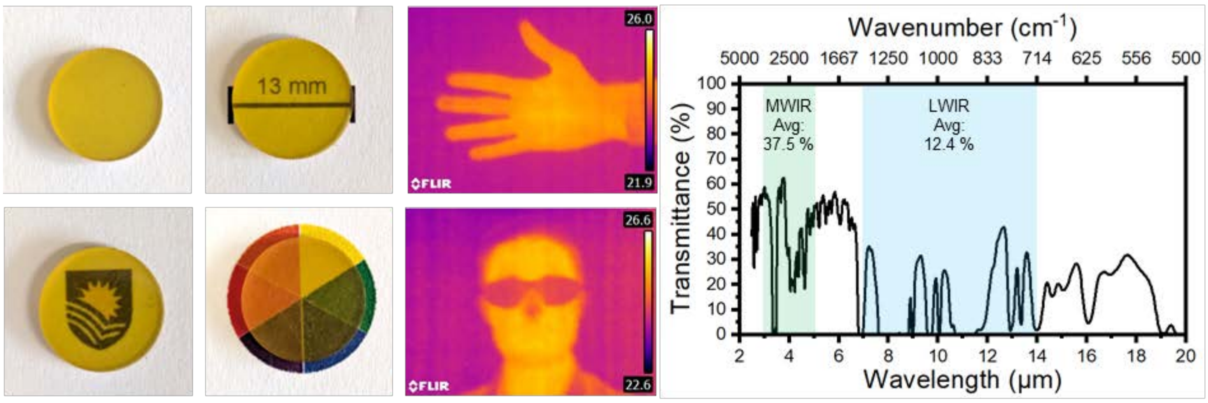

1.48 mm thick

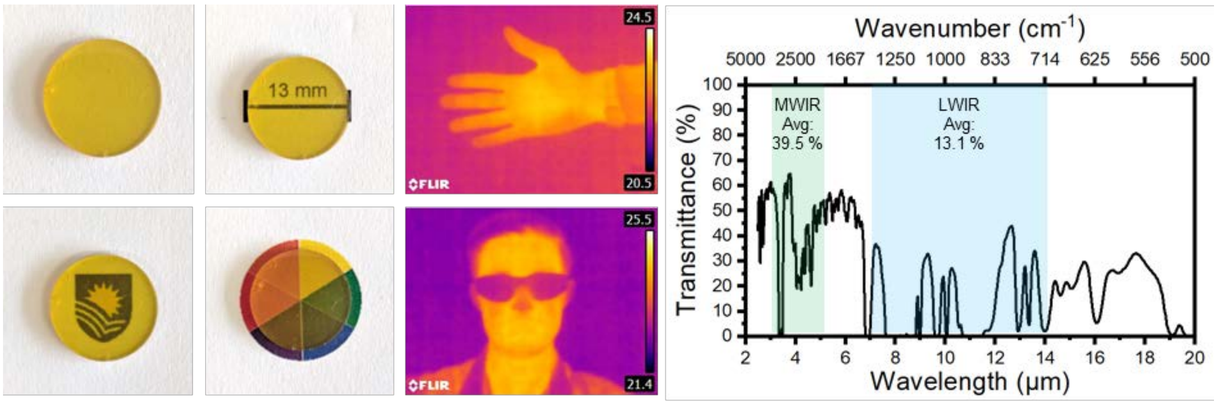

1.41 mm thick

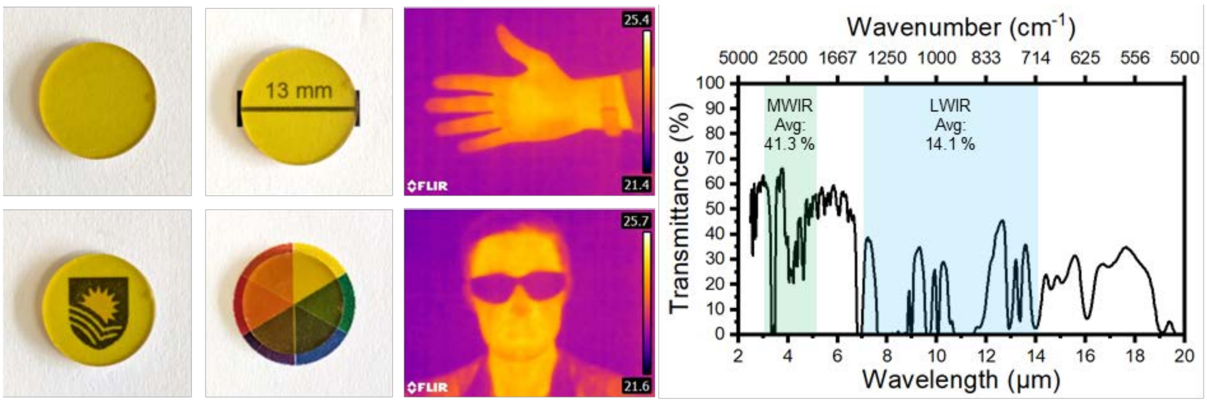

1.33 mm thick

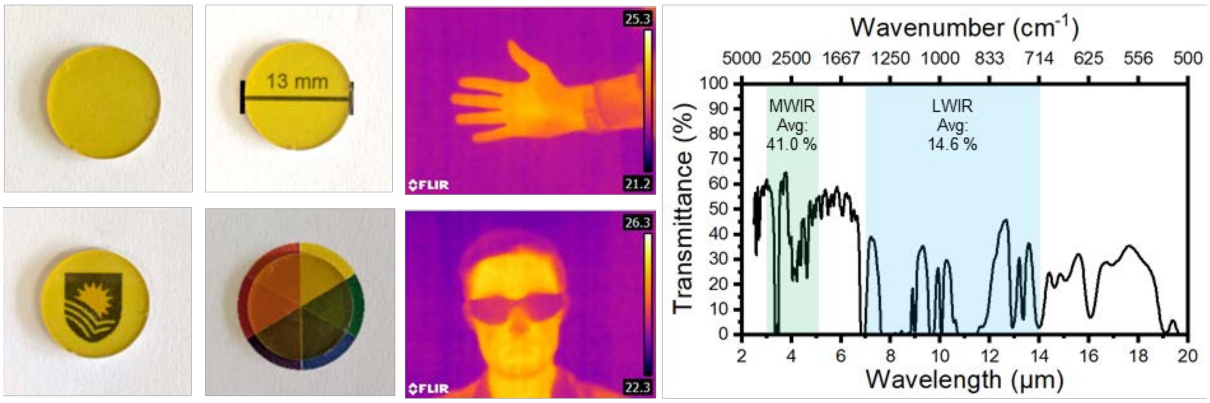

1.26 mm thick

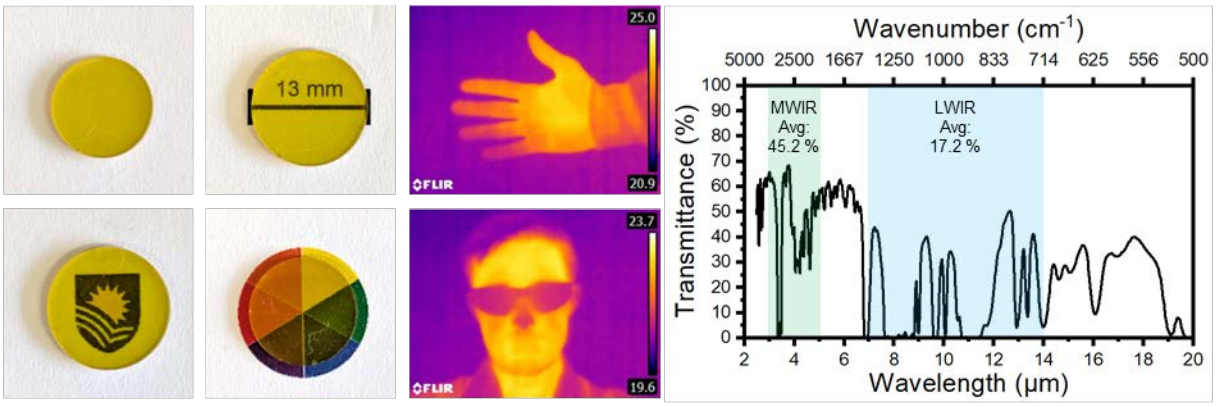

1.11 mm thick

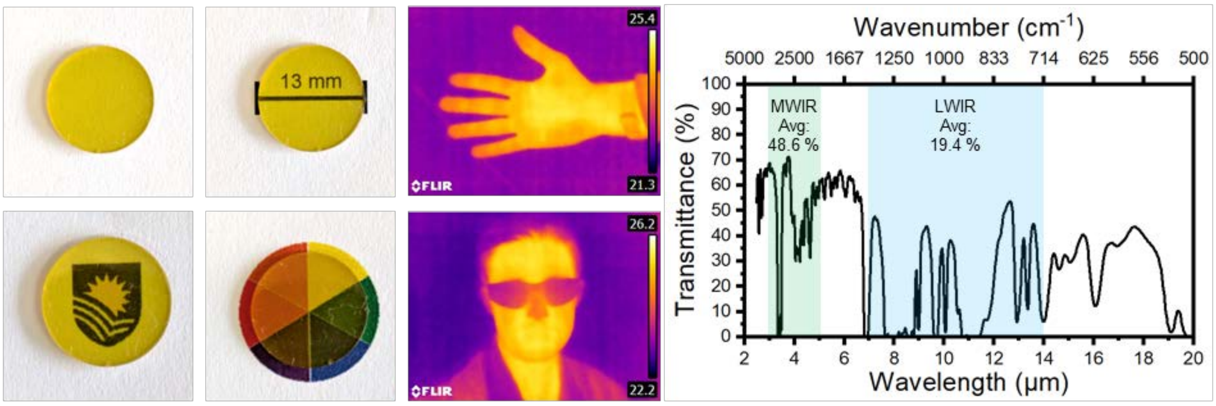

1.00 mm thick

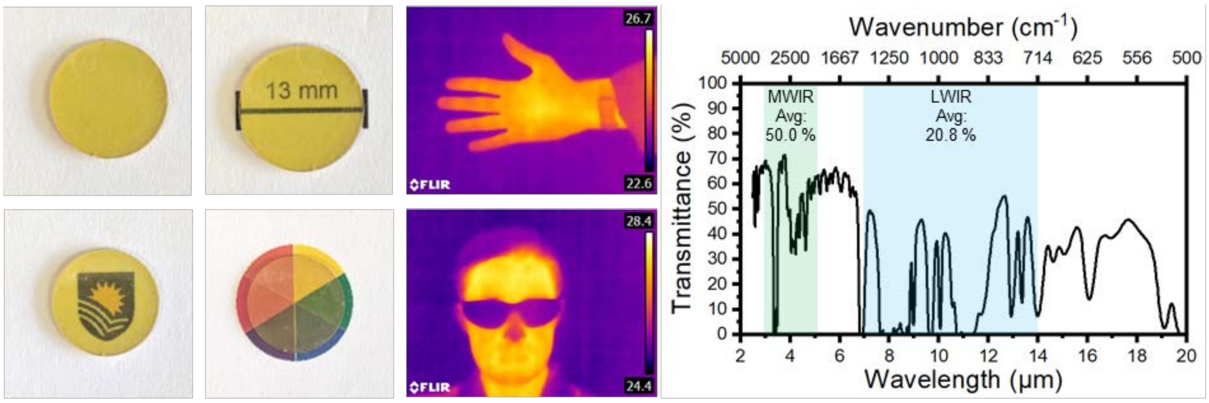

0.90 mm thick

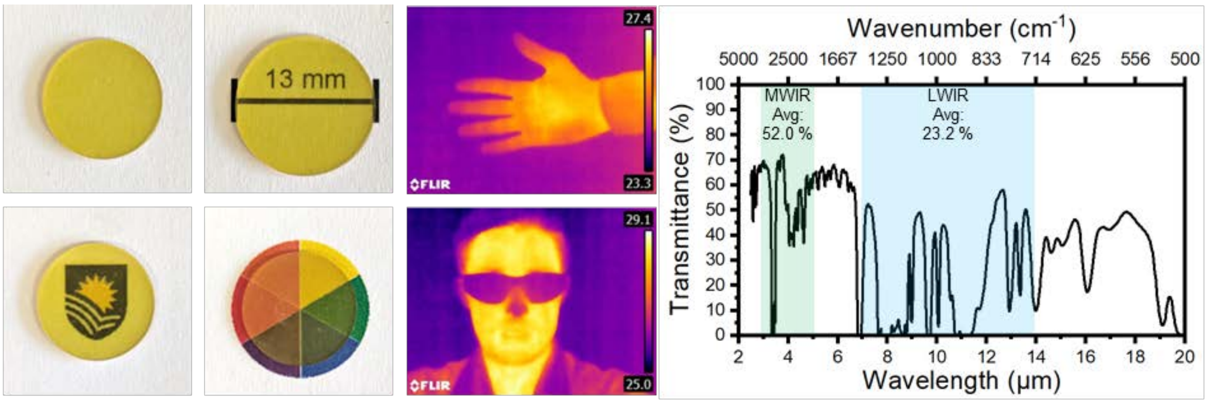

0.80 mm thick

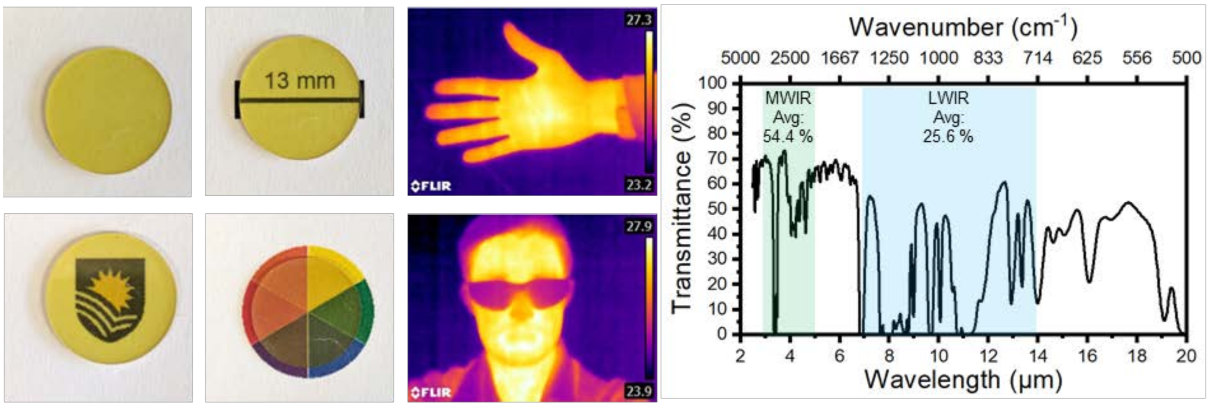

0.71 mm thick

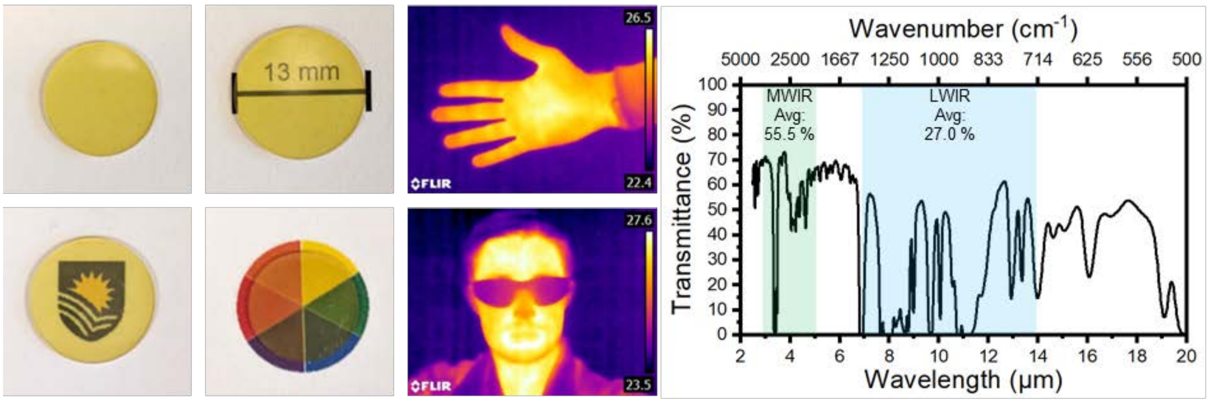

0.64 mm thick

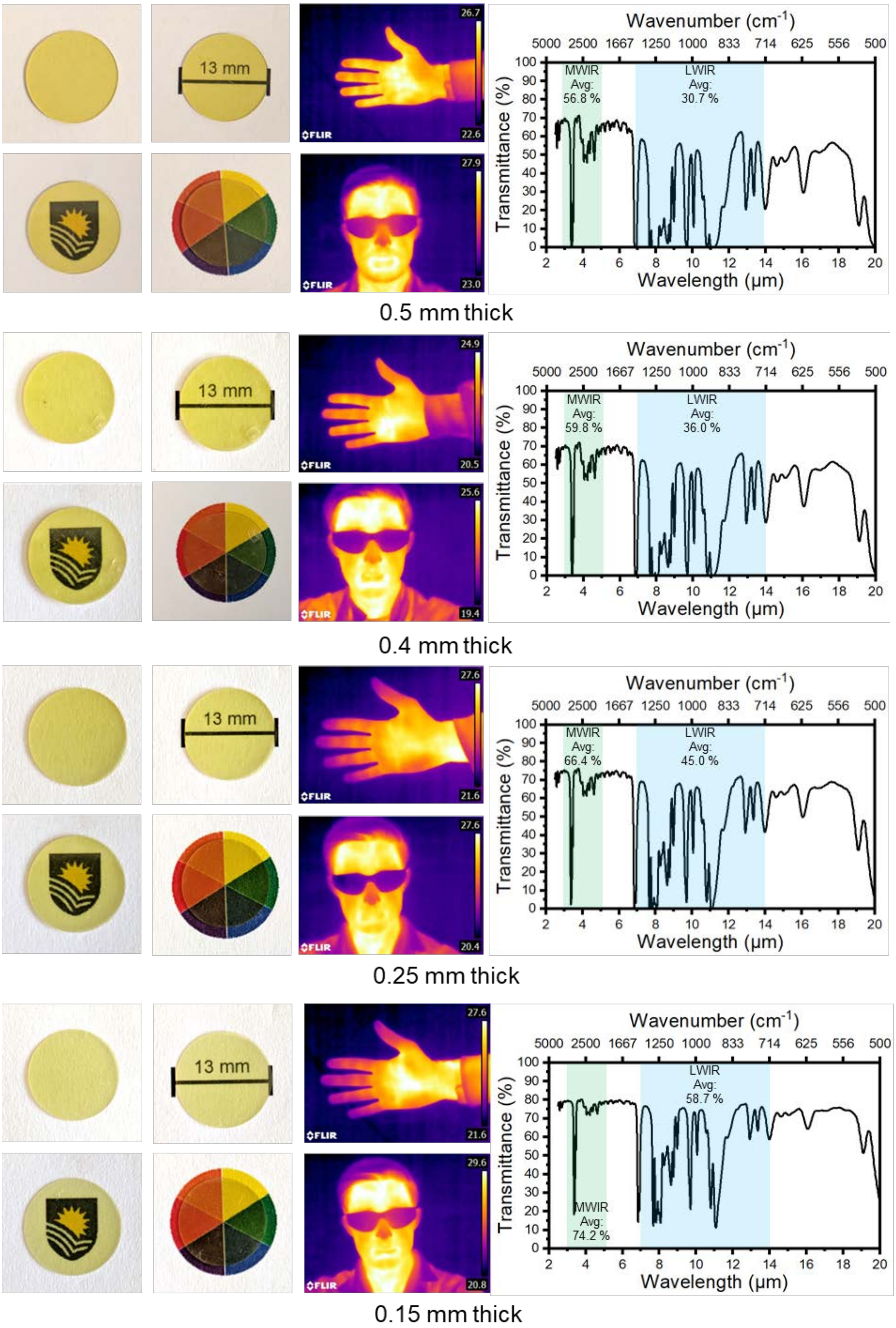

Figure S69: Photos, LWIR images and FTIR spectra through polymer **1** at different thickness.

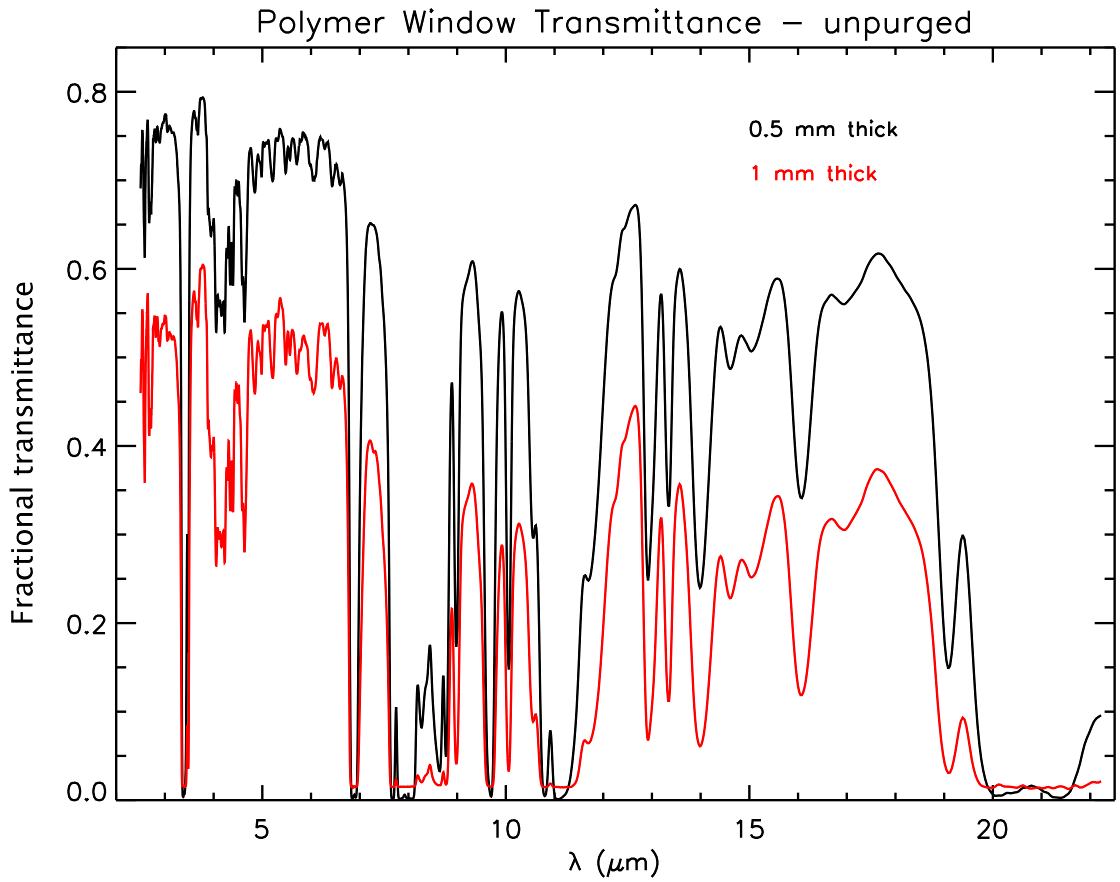

Figure S69 (continued): Expanded infrared spectrum of polymer **1** obtained through a 1 mm thick window and a 0.5 mm thick window

Refractive index testing of polymer 1

To measure the refractive index of polymer **1**, the specular reflectance of the polymer was measured and converted to refractive index using a Kramers-Kronig transform. It was found that the window thickness and surface roughness were vitally important when measuring reflectance. When thin windows were used (less than 2 mm thick), the reflectance spectrum would show a similar pattern to the transmittance spectra. This was likely due to light transmitting through the surface, reflecting off the bottom surface before being collected by the sensor. Due to these effects, the sample geometry was carefully considered. Cylinders of polymer **1** were made with a diameter of 25 mm and a thickness of 10 mm. This thickness ensured that very little light would transmit through the entire sample. Only the top face of the polymer window was polished. The polishing process was important to avoid light scattering. Therefore, the top surface of the polymer sample was polished using sandpaper with a range of grits. Starting at 200 grit, the surface was sanded flat. Next, the sample surface was wet and dry sanded with micro mesh polishing kit with sandpaper grits from 1500 to 12000. A micro gloss polish with a 1-micron abrasive crystal was then applied to the surface. Specular reflectance spectra were measured using a Bruker Vertex v80 instrument with the 1513/QA attachment for the polymers prepared from monomer **9** and **10** with sulfur. An angle from the surface normal of 15 ° was used under vacuum. The sample was referenced using the reflectance spectrum of an aluminum mirror. A Kramers-Kronig transform was applied using the OPUS V7.2 software to find the wavenumber dependent phase shift spectrum. The refractive index was then calculated using the real portion of the complex refractive index, $\eta = n + ik$.

For many applications, the dispersion or change in refractive index with wavelength is very important. The dispersion of a material is defined in the visible region by the Abbe number. The Abbe number uses the refractive index at the Fraunhofer's C (656.3 nm), d (587.6 nm) and F (486.1 nm) spectral lines to quantify the dispersion of the material. For long wave infrared transparent materials, the V-number is more commonly used. Materials that are used for thermal imaging are mostly interested in light between 7 and 14 microns so instead of using the Fraunhofer's spectral lines, the refractive index at 8 µm, 10 µm and 12 µm are used. To calculate the V-number, the following equation is used:

$$V = \frac{n(10) - 1}{n(8) - n(12)}$$

Where V is the V-number and n(x) is the refractive index at x µm. The polymer had a high average refractive index over the long wave infrared region (7 µm - 14 µm) of 1.871 and a V-number of 314.

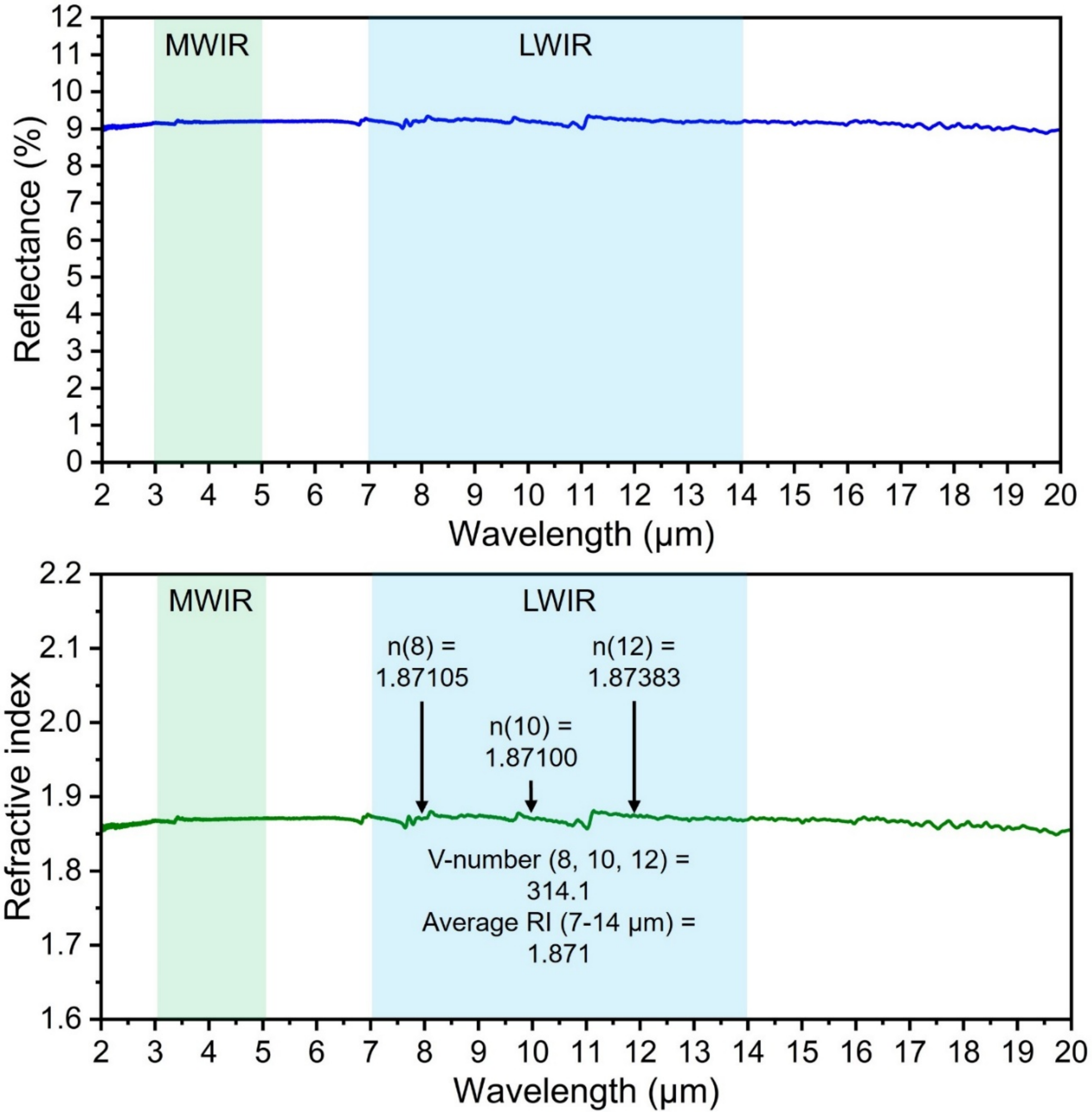

Figure S70: Reflectance and refractive index spectra of polymer **1** made from sulfur and monomers **9** and **10** with 81 % sulfur. All spectra were obtained on a Bruker Vertex v80 using the 1513/QA attachment. The V-number was calculated using the equation on the previous page and the refractive index at 8 µm, 10 µm and 12 µm.

Computational study of polymer 1

Computational methods

IR spectra of small molecules and oligomers of **1** were simulated using the density functional theory (DFT) method wB97XD[13] in combination with a double zeta basis set 6-31G(d)[14] implemented in Gaussian 16.[15] For complicated oligomers of **1** with a very large number of conformers, the low-energy conformers were screened with Conformer-Rotamer Ensemble Sampling Tool (CREST),[16] and further geometrical optimization was conducted for a set of 5 lowest conformers to identify the lowest-lying conformers before simulation of IR spectra. For oligomers with high numbers of repeating units (20 and 30), polymer matrices were generated by using the Polymatic package[17] integrated with LAMMPS.[18] These oligomer matrices were geometrically optimized, and their IR spectra were simulated at the GFN2-xTB[19] level of theory (Slater-Type AO Basis Sets designed for specific elements) with the aid of the XTB package.[20] Note that a scaling factor of 0.943[21,22] was applied to all vibrational frequencies predicted at the wB97XD level of theory. All simulated IR spectra were visualized using GaussView 6[23] with standard setting parameters.

Convergence of IR spectra at DFT

The convergence of simulated IR spectra for both linear (L) and cross-linked (CL) polymers of **1** were examined. The IR intensities were found to increase with molecular weight due to the additional vibrational modes. However, the changes are systematic and are essentially converged once the oligomers have 6 repeat units Figure S71 (panels a and c). There are also no significant differences in the IR signal pattern between the linear polymer matrix and the cross-linked polymer once the number of repeat units reaches 6 (Figures S71e and S71f). Therefore, the IR spectra of oligomers containing 6 repeated units were simulated to represent macro matrix of polymer **1** at DFT levels of theory.

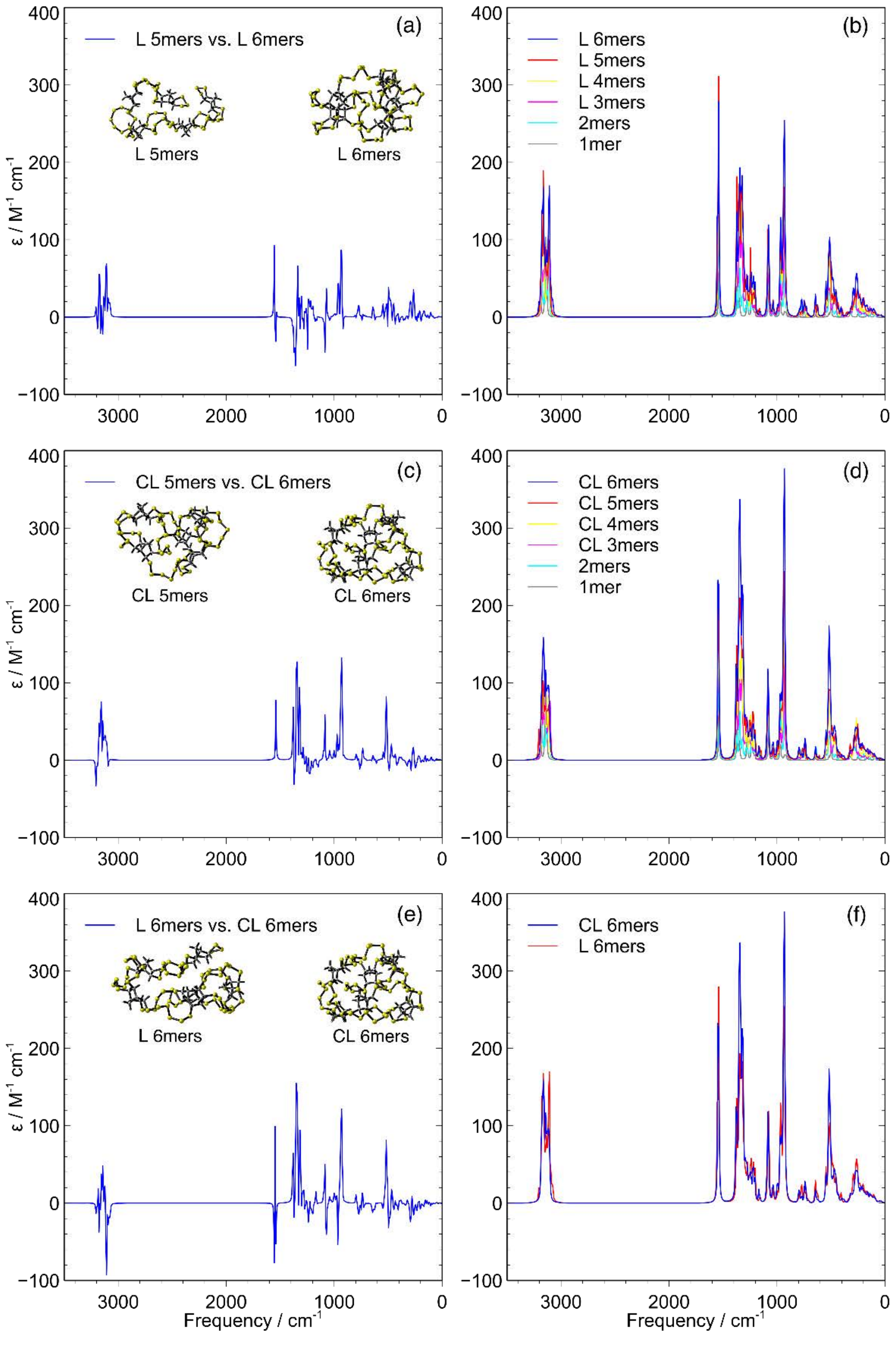

Figure S71: Comparison of IR spectra of **1** as a function of molecular weight and chain topology: linear (L) vs cross-linked (CL). Panels a, c, and e show the subtracted difference in intensities of IR spectra; panels b, d, and f show the simulated spectra of the monomer and various oligomers. No scaling factor was applied.

Integrated simulated spectra

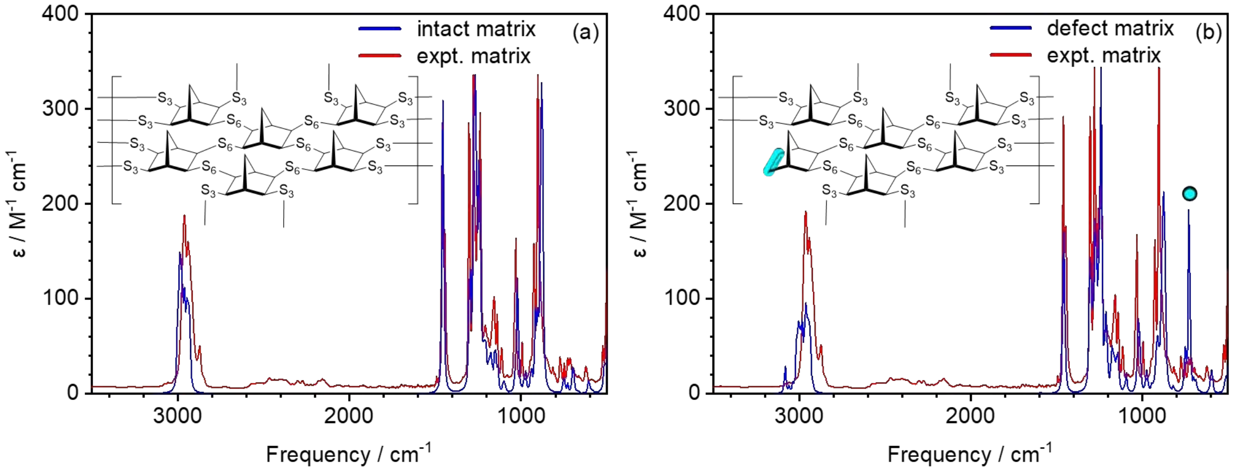

Figure S72: Simulated spectra of: (a) an intact oligomer matrix of **1** (containing 6 repeat units) (b) a defected matrix containing one C=C double bond. In the defected polymer matrix, shoulder peaks at ~3080 cm^{-1} are caused by the stretching vibrations of C-H in C=C double bonds, and a high-intensity peak around 720 cm^{-1} is caused by out-of-plane bending vibrations of C-H in C=C double bonds. Both spectra are plotted against the transmission spectrum of a freestanding window of polymer **1** (81% sulfur) with a thickness of 0.37 mm.

IR spectra of large polymer matrices

The wB97XD and XTB vibrational *frequencies* correlate very well with one another, but the *intensities* show some differences. Based on a comparison of the frequencies of the same conformers obtained from these two levels of theory (wB97XD and XTB), a scaling factor of 0.992 was used to rescale vibrational frequencies predicted at the XTB level for larger polymer matrices. While the XTB method cannot completely describe the intensities of several vibrational modes compared with wB97XD, the lower level of theory provides a reasonable description of the general features of the IR spectra (see Figure S73b)

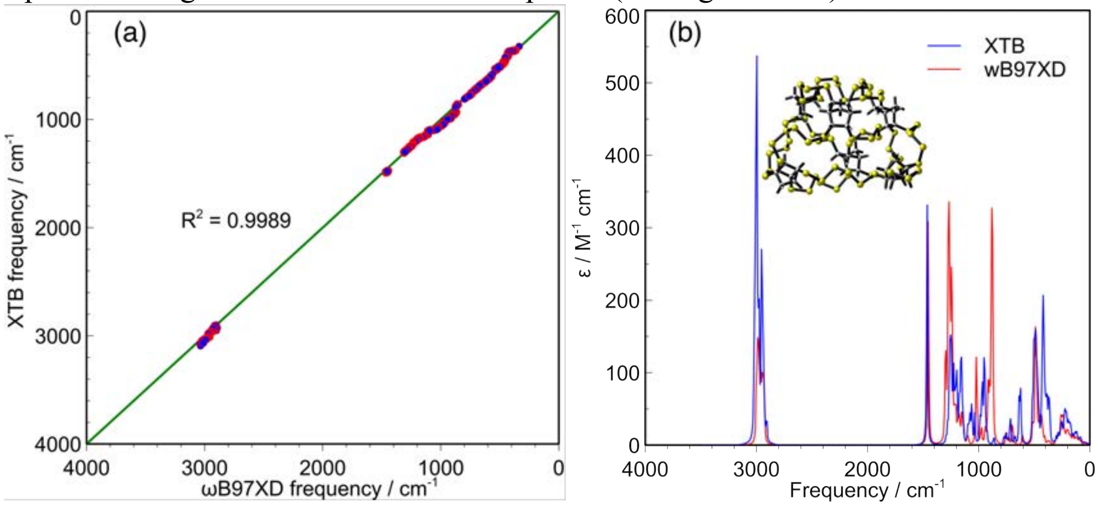

Figure S73: (a) Correlation between the wB97XD vibrational frequencies (scaled by a factor of 0.943) and the XTB ones. (b) Comparison of simulated IR spectrum of a cross-linked 6mer (CL 6mer) of **1** at wB97XD (scaled factor of 0.943) and XTB levels (scaled factor of 0.992).

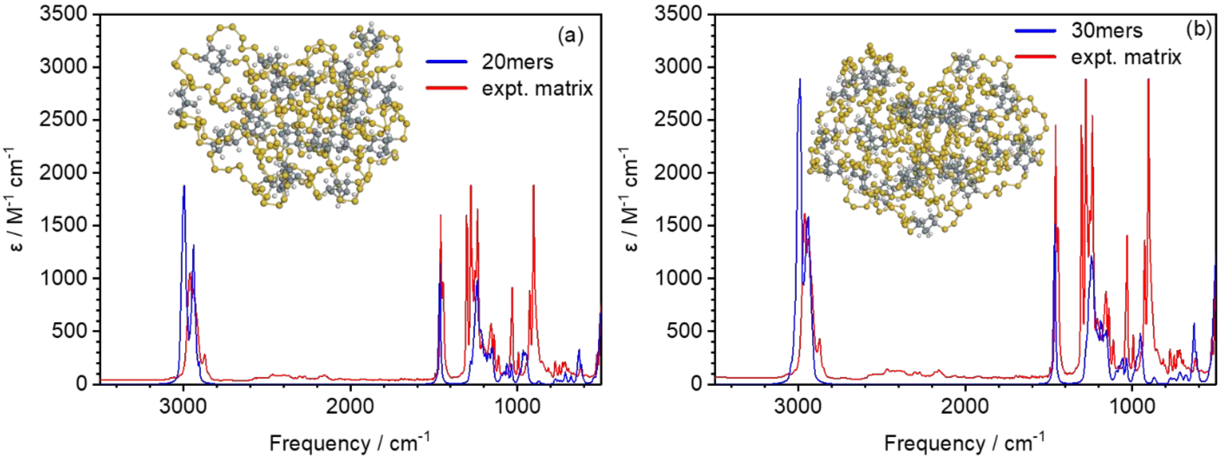

Figure S74: Simulated IR spectra of the oligomer of **1** containing 20 (a) and 30 (b) repeated units of monomers using a scaling factor of 0.992 obtained previously.

There is no significant difference between the IR spectra of 20- and 30-mers, other than their intensities, which are slightly higher in the spectra of the 30-mers due to the presence of additional vibrational modes.

IR spectra of other monomers

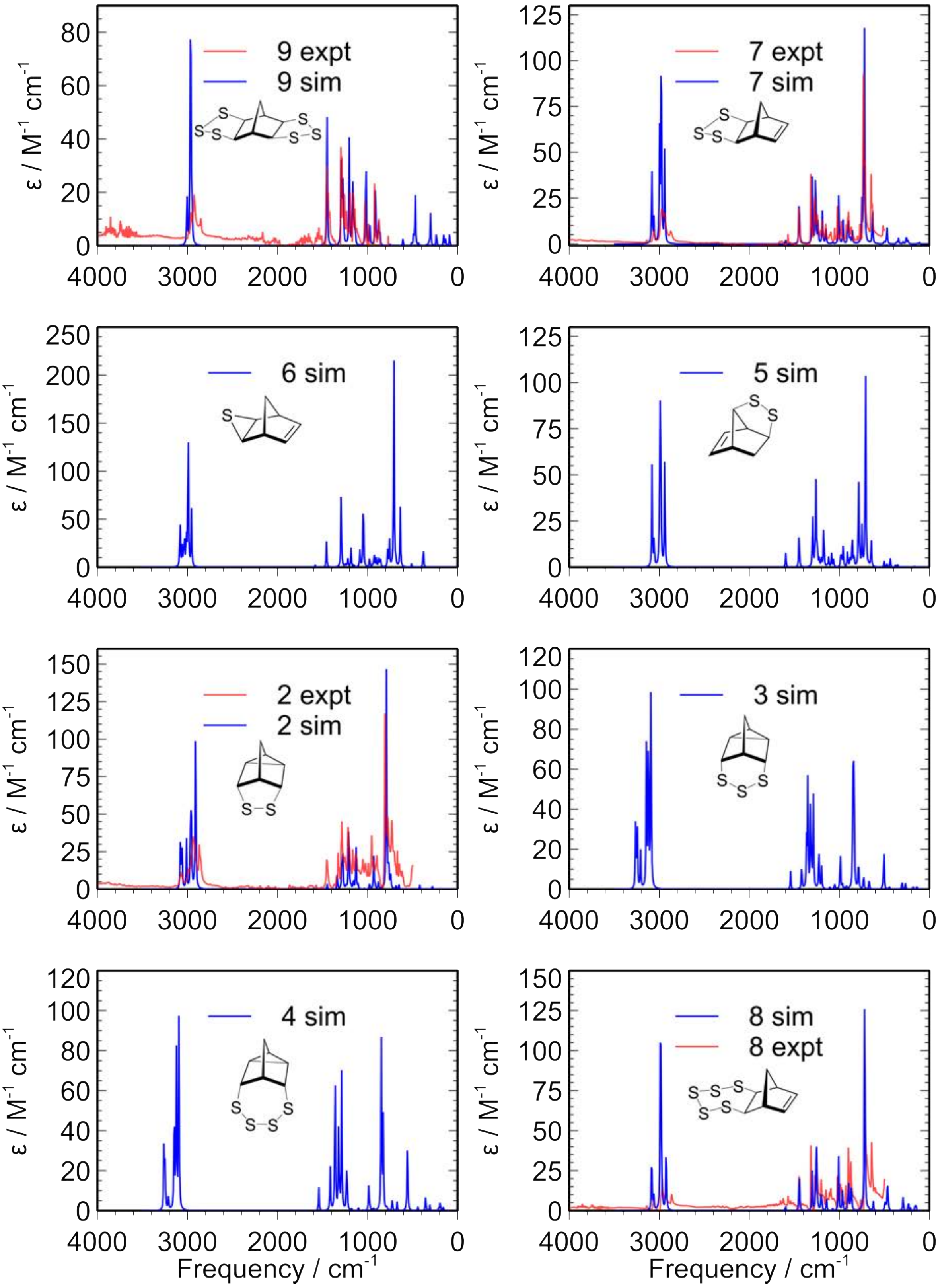

Figure S75: Experimental and simulated IR spectra of sulfurized norbornadiene derivatives in gas phase.

Preparation of lenses for FLIR Lepton 3.5

Lens designs

A total of 7 lenses were selected to investigate a range of focal lengths and f-numbers. These lenses would allow the polymer lenses to be investigated on the FLIR Lepton 3.5. These lenses were all plano convex and glass versions were purchased from Edmund Optics so they could be used to make silicone molds to cast polymer **1** in the same form. The radius of the curved section was selected to give the desired focal length when accounting for the higher refractive index of the polymer over glass. Below is a table showing the lens designs and technical parameters. The focal length in this table accounts for the refractive index of polymer **1**.

Table S3: Data of lenses used for making molds and casting polymer lenses.

**Lens number**	**Lens type**	**Radius (mm)**	**Focal length**	**Diameter (mm)**	**F-number**	**Center thickness**	**HFOV Lepton (°)**
**1**	Plano Convex	1.28	1.5	1.5	f/1	0.8	65
**2**	Plano Convex	1.28	1.5	2	1/0.75	0.8	65
**3**	Plano Convex	1.70	2	2	f/1	0.8	51
**4**	Plano Convex	1.70	2	2.5	f/0.8	0.8	51
**5**	Plano Convex	2.12	2.5	2.5	f/1	0.8	42
**6**	Plano Convex	2.55	3	2.5	f/1.2	0.8	35
**7**	Plano Convex	4.25	5	2.5	f/2	0.8	22

The lens designs were selected so the effect of focal length and f-number could be investigated. There were a range of focal lengths selected from 1.5 mm and 5 mm, to show the differences in the field of view and zoom. For two of the focal lengths, there were two lenses with different diameters. These were selected to show how the difference in f-number affects imaging when focal length was maintained. There were also several lenses with the same diameter and different focal lengths. Four lenses all had a diameter of 2.5 mm while the focal length ranged from 2 mm to 5 mm. These lenses would give a good understanding on how the imaging is changed while maintaining the diameter of the lens and altering the focal length. The center thickness of all lenses was maintained at 0.8 mm. This was important so that comparisons between the lenses could be made. The polymer **1** (81% sulfur) has approximately 23% transmission of LWIR radiation at this thickness. The expected horizontal field of view of each lens when using the FLIR Lepton was calculated using the focal length and the horizontal size of the sensor. The sensor in the FLIR Lepton 3.5 has a resolution of 160 x 120 with a 12-micron pixel size. The horizontal sensor size was calculated as 1.920 mm.

$$FOV = 2\arctan\left(\frac{x}{2f}\right)$$

Where x is the width of the sensor and f is the focal length.

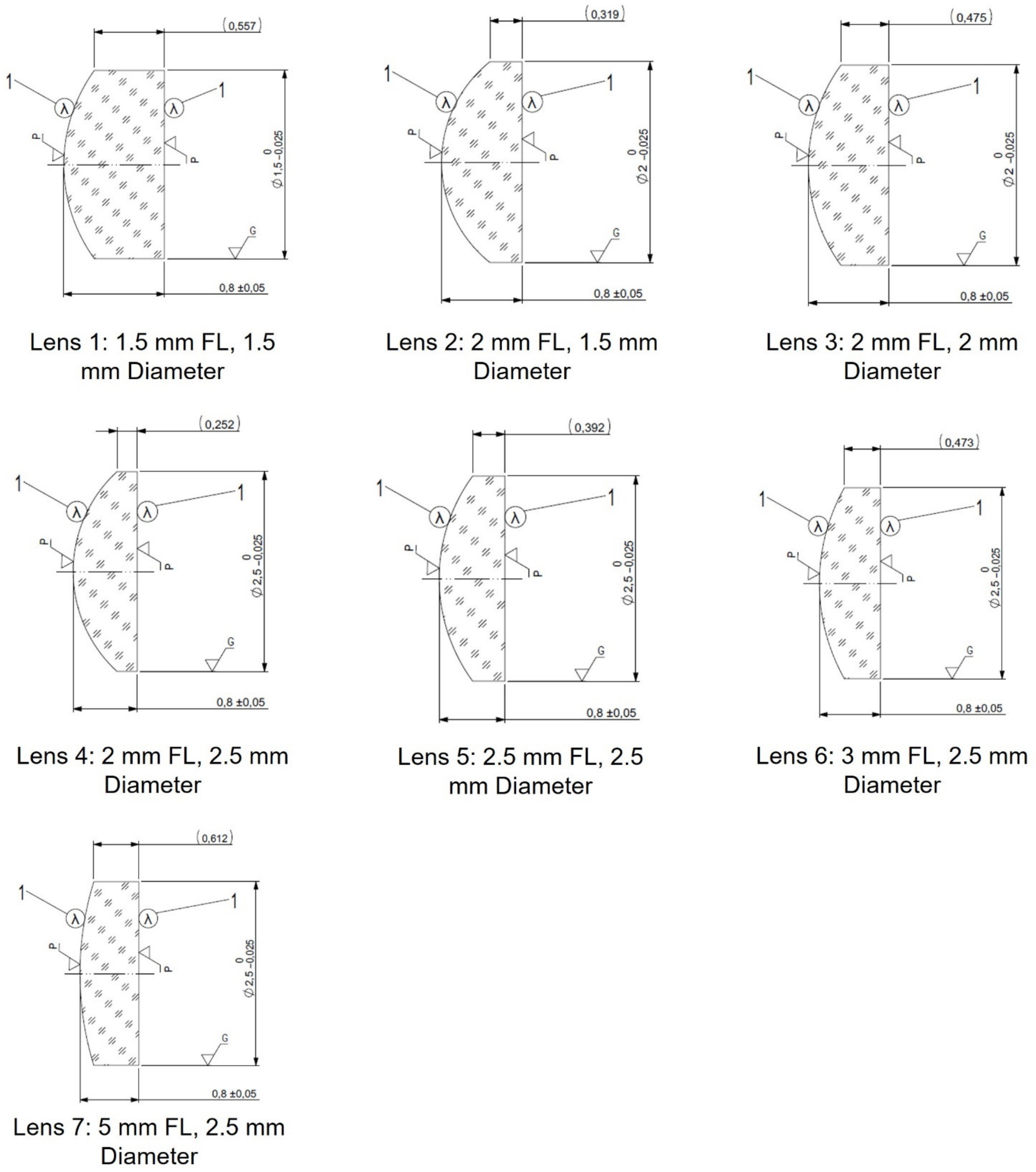

Figure S76: Diagrams showing dimensions of glass lenses purchased from Edmund Optics to use as positives for silicone molds. All dimensions are in mm.

Preparation of molds and lens casting

To cast lenses from polymer **1**, molds were first prepared using the glass positives. 3D printed parts were used to hold the positives. This allowed for the incorporation of some non-imaging carrier material to the lenses. The carrier material provided mechanical strength to the lens and also aided in molding and mounting of the lenses. The 3D printed part also allowed for the incorporation of a funnel and air outlet which helped with consistency in casting polymer **1**. To prepare a silicone mold, the glass lens was added to the 3D printed part and a M4504 silicone was mixed and poured into the opening to create the cavity side of the mold. The flat side of the mold was prepared by placing the outer piece on a glass slide and the same silicone was poured into the opening. The glass slide and lens were submerged in water with detergent, then dried, to prevent the silicone resin from sticking to the glass after curing. The final mold consisted of two silicone parts that were held together with a clamp when casting. To cast polymer **1** lenses, the silicone mold was added to a preheated oven at 100 °C and polymer **1** was prepared by the standard method described earlier. The liquid prepolymer was poured into the mold before being returned to the oven to cure at 100 °C for 1 hour. After curing, the funnel and outlet were removed by scoring a line with a scalpel and breaking the polymer **1**. Any excess polymer was removed using a hand file until only the lens and carrier material remained.

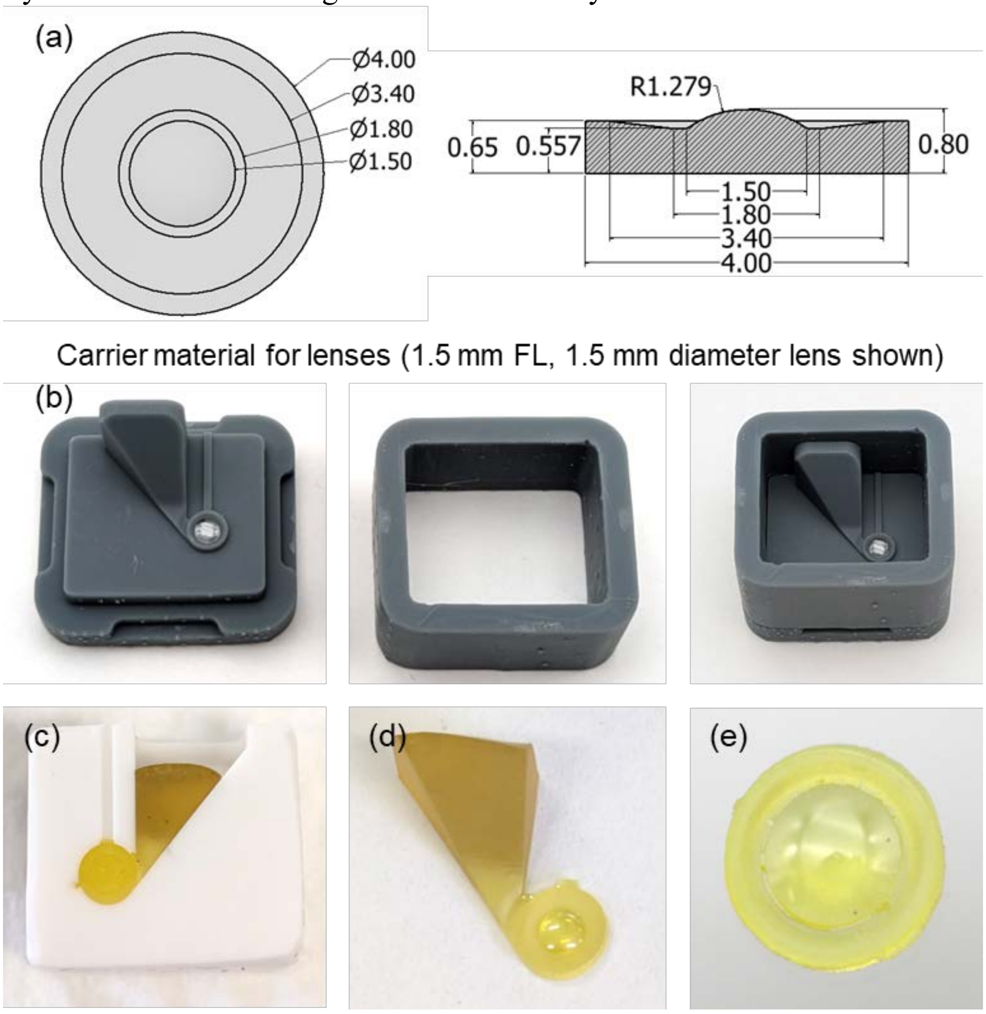

Figure S77: (a) Carrier material design. Lens 1 is shown, and all dimensions are in mm. (b) 3D printed parts used to make silicone mold. (c) Polymer **1** directly after curing in silicone mold. (d) Polymer **1** after removal from mold. (e) Photo of a lens after funnel and outlet are removed.

Overview of lens designs

This section shows all the lenses that were prepared and detailed the dimensions and important parameters of each. All lenses have the same carrier material design apart from lens 7 which had a much thicker carrier material. All lenses were plano convex with diameters varying from 1.5 mm to 2.5 mm and focal lengths ranging from 1.5 mm to 5 mm

Focal Length	1.5 mm
Back Focal Length	1.07 mm
Lens Type	Plano Convex
Diameter (lens)	1.5 mm
F-number	f/1
Centre thickness	0.8 mm
Radius	1.279 mm
Edge thickness (lens)	0.557 mm
Carrier material diameter	4 mm
Carrier material edge thickness	0.65 mm
Refractive Index	1.871
Glass transition temperature	137 °C

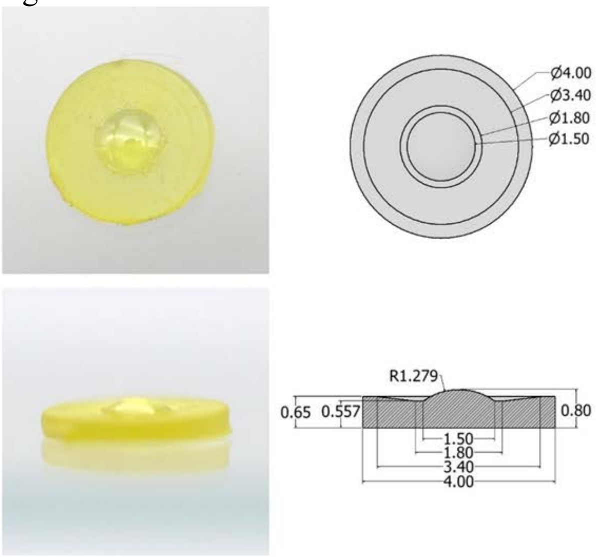

Lens 1

Focal Length	1.5 mm
Back Focal Length	1.07 mm
Lens Type	Plano Convex
Diameter (lens)	2 mm
F-number	f/0.75
Centre thickness	0.8 mm
Radius	1.279 mm
Edge thickness (lens)	0.319 mm
Carrier material diameter	4 mm
Carrier material edge thickness	0.65 mm
Refractive Index	1.871
Glass transition temperature	137 °C

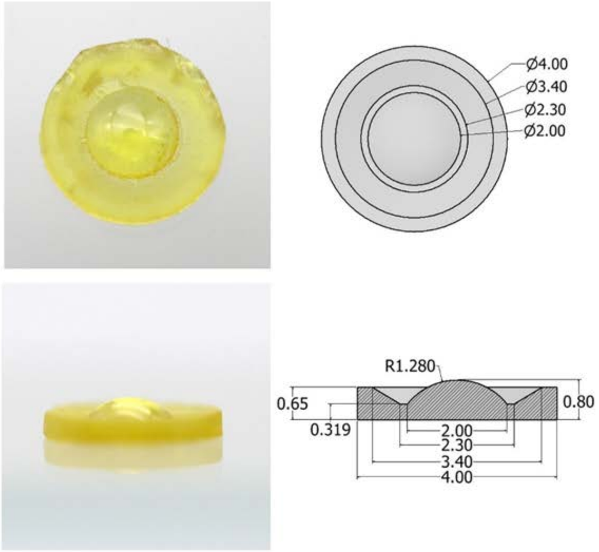

Lens 2

Focal Length	*2 mm*
Back Focal Length	*1.57 mm*
Lens Type	*Plano Convex*
Diameter (lens)	*2 mm*
F-number	*f/1*
Centre thickness	*0.8 mm*
Radius	*1.7 mm*
Edge thickness (lens)	*0.475 mm*
Carrier material diameter	*4 mm*
Carrier material edge thickness	*0.65 mm*
Refractive Index	*1.871*
Glass transition temperature	*137 °C*

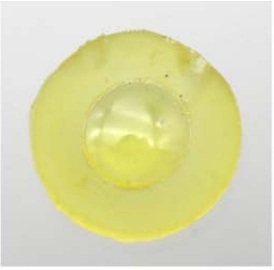
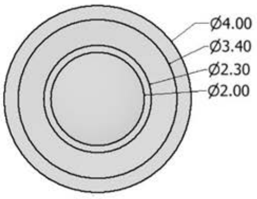

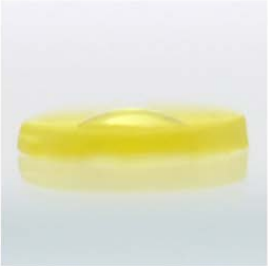
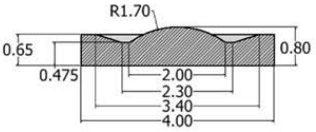

Lens 3

Focal Length	*2 mm*
Back Focal Length	*1.57 mm*
Lens Type	*Plano Convex*
Diameter (lens)	*2.5 mm*
F-number	*f/0.8*
Centre thickness	*0.8 mm*
Radius	*1.7 mm*
Edge thickness (lens)	*0.252 mm*
Carrier material diameter	*4 mm*
Carrier material edge thickness	*0.65 mm*
Refractive Index	*1.871*
Glass transition temperature	*137 °C*

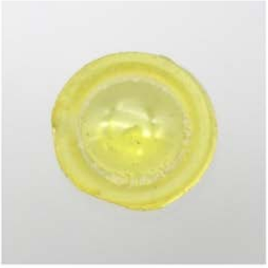
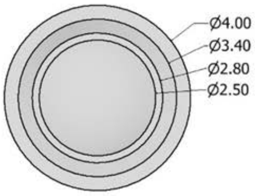

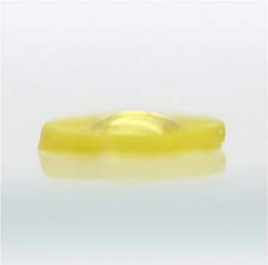
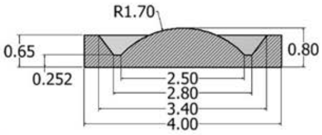

Lens 4

Focal Length	*2.5 mm*
Back Focal Length	*2.07 mm*
Lens Type	*Plano Convex*
Diameter (lens)	*2.5 mm*
F-number	*f/1*
Centre thickness	*0.8 mm*
Radius	*2.12 mm*
Edge thickness (lens)	*0.392 mm*
Carrier material diameter	*4 mm*
Carrier material edge thickness	*0.65 mm*
Refractive Index	*1.871*
Glass transition temperature	*137 °C*

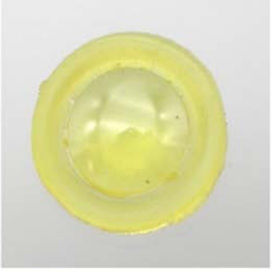
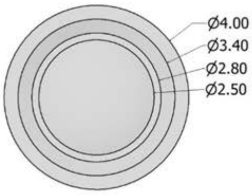

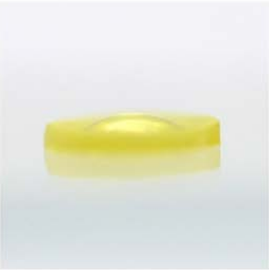
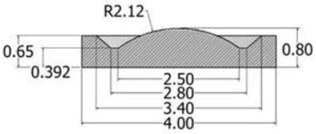

Lens 5

Focal Length	*3 mm*
Back Focal Length	*2.57 mm*
Lens Type	*Plano Convex*
Diameter (lens)	*2.5 mm*
F-number	*f/1.2*
Centre thickness	*0.8 mm*
Radius	*2.55 mm*
Edge thickness (lens)	*0.473 mm*
Carrier material diameter	*4 mm*
Carrier material edge thickness	*0.65 mm*
Refractive Index	*1.871*
Glass transition temperature	*137 °C*

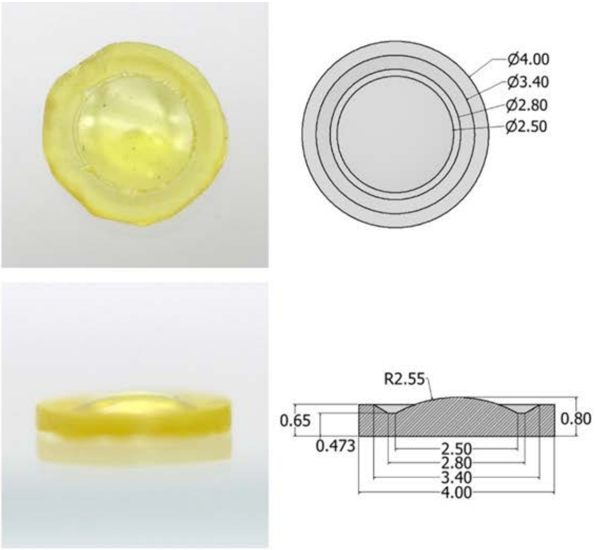

Lens 6

Focal Length	*5 mm*
Back Focal Length	*4.57 mm*
Lens Type	*Plano Convex*
Diameter (lens)	*2.5 mm*
F-number	*f/2*
Centre thickness	*0.8 mm*
Radius	*4.25 mm*
Edge thickness (lens)	*0.612 mm*
Carrier material diameter	*4 mm*
Carrier material edge thickness	*1 mm*
Refractive Index	*1.871*
Glass transition temperature	*137 °C*

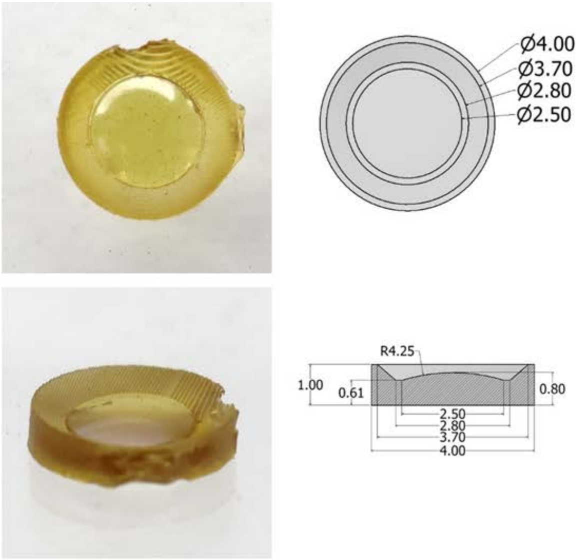

Lens 7

Figure S78: Images and dimensions for all lens designs. All dimensions are in mm.

Design of mount for FLIR Lepton 3.5

The FLIR Lepton 3.5 had an internal thread with a pitch of 350 µm. The holder was designed to thread into this component of the module. The thread not only provided a secure fitting for the holder but also allowed for fine control of the distance from the sensor to the bottom of the holder. This feature was used to find focus each lens during imaging. The holders had a hole through the middle and an opening where the lens could be placed. After inserting the lens, a press fit cap could be inserted. The cap ensured that the lens was held securely and did not move or fall out of the holder. For all but one sample, the distance between the bottom edge of the holder and the sensor was maintained at 500 µm. As there was a range of focal length lenses, several different holders were prepared which controlled the height at which the lens was held. After removing the shutter and lens from the FLIR Lepton 3.5, the holder could be directly threaded into the camera, allowing the lens to be used for imaging. As the shutter was removed, the camera would need manual full field calibration between each image. The calibration was done by covering the lens with a LWIR opaque material and calibrating in the Lepton User App.

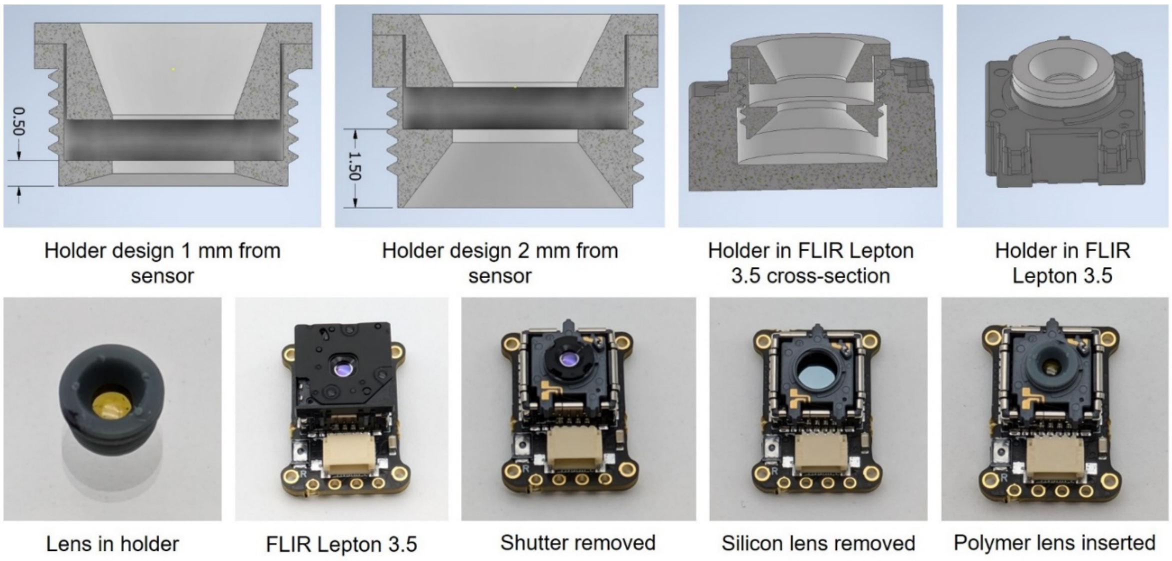

Figure S79: 3D printed holder designs and integration into a FLIR Lepton 3.5 thermal camera module.

Mask designs

To investigate the imaging when using the polymer lenses, several masks were prepared. These masks were cut from 3 mm thick acrylic using a laser cutter. A 3D printed holder with a thread allowed these masks to be attached to an optical rail. The masks were then positioned in front of a hotplate for imaging. The first mask was a USAF 1951 resolution target. This target is designed to test the resolving power of an optical system.[24] The USAF target utilizes line pairs with varying thickness and separation. The maximum resolution of the camera can be determined by the smallest set of three lines which can be resolved. The number of line pairs per millimeter and the width of each line is determined by the element and group number. The group number is written next to every set of 6 lines while the element is written next to its associated group. The elements and groups used for the target as well as their dimensions can be seen in the table below. The only modification to the standard USAF 1951 target is that three square openings with side lengths of 14, 10 and 8 mm were placed instead of an extra two groups due to the limitations on the minimum dimensions of the laser cutter. It should be noted that the target is usually used with visible light and evaluates the resolution of the entire imaging system. Here, it is evaluating the FLIR lepton 3.5 as well as the lenses.

Table S4: Number of line pairs per millimeter and the width of each line in the USAF 1951 optical target.[24,25]

	**Number of line pairs per millimeter**		**Width of line in micrometers (µm)**	
	**Group**		**Group**	
**Element**	**-2**	**-1**	**-2**	**-1**
**1**	0.25	0.5	2000.0	1000.0
**2**	0.281	0.561	1781.8	890.9
**3**	0.315	0.630	1587.4	793.7
**4**	0.354	0.707	1414.2	707.1
**5**	0.397	0.794	1259.9	630.0
**6**	0.445	0.891	1122.5	561.2

The next mask was a custom infrared mask based on a design by Pyun and co-workers.[26] It had three sets of 6 lines. For each set, three lines were horizontal and three were vertical. The widths of the lines were 1 mm, 2 mm, 3 mm for the first second and third sets respectively. The horizontal and vertical lines in each set had three different lengths with 3 mm, 7.5 mm and 12 mm and the distance between the outer edges of each group of vertical or horizontal lines was 12 mm. This target is useful to show general imaging and demonstrate the focus of the lens. The range of line widths also gives an indication on the resolution of the imaging system.

The final mask was a star mask. This mask consisted of a 60 mm diameter circle broken into 36 sectors. This gave it a 10 ° pitch with each sector being 5 °. In the center, there was a circle with a diameter of 10 mm. This mask is useful to see any astigmatism or focusing effects.[27] If there was any warping or inconsistency in the lens focus, the sectors would no longer be straight.

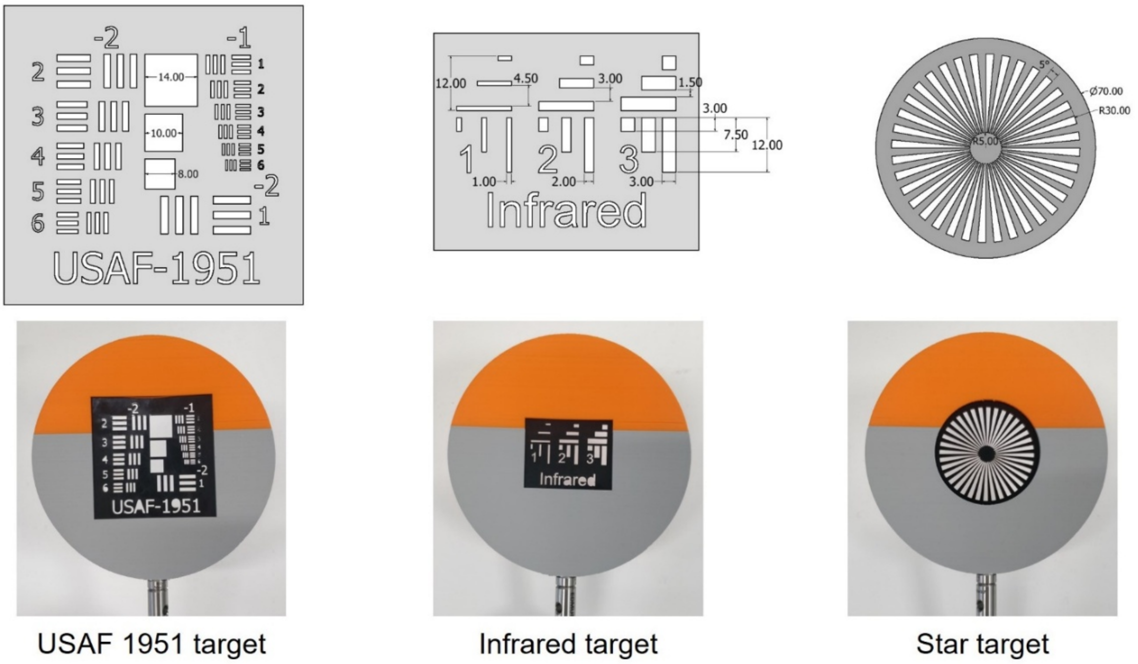

Figure S80: Laser cut masks for imaging using polymer lenses on the FLIR Lepton 3.5.

These masks were useful for investigating the imaging with the polymer lenses. However, there were several limitations that should be noted. First, the mask thickness is 3 mm. This meant that if the mask was not perfectly aligned with the camera, the gaps may become obscured due to parallax effects. For this reason, the lines or openings may appear smaller than the dimensions of the mask. It is possible that the thickness of the masks may also block much of the non-parallel light from reaching the camera, potentially affecting the focus of the lens. The second limitation of the masks is that they can heat up over time while being close to the heat source. As the masks heat up, they may emit long wave infrared light of their own. As this occurs, the contrast in the images of the masks may decrease. Overall, these limitations mean that the resolution and thermal sensitivity determined using these masks likely constitutes a lower estimate of performance.

Focal length testing

The USAF 1951 target was placed 5 cm from a 100 °C hotplate. The FLIR Lepton 3.5 was mounted onto a 3D printed holder with a thread. This mount allowed the imaging module to be attached to an optical rail and placed 20 cm from the mask. The only exception to this was lens 7 which was placed 30 cm from the mask so that the USAF-1951 target filled the vertical space of the image. The FLIR lepton was plugged in to a laptop and used to take an image of the target. By using 3D printed lens holders, the distance between the back surface of the lens and the sensor could be varied. Between each image, the holder was turned 90 °. As the thread in the FLIR Lepton 3.5 had a pitch of 350 µm, a turn of 90 ° resulted in a change in distance of 87.5 µm. A range of images were taken, and the most focused image was determined qualitatively. This distance was used for all further tests. The exact value obtained by this test is the distance from the plano surface of the lens to the highest point on the sensor. This distance obtained in this experiment should not be considered as the exact back focal length of the lens as the FLIR Lepton 3.5 has a silicon cap before the sensor. The exact thickness of the cap and separation from the sensor is not known but it is likely several hundred microns. Nonetheless, the value obtained in this experiment is useful to determine the optimum focus for future tests.
In general, it was found that the longer focal length lenses gave a sharper image and had a broader tolerance for the focus of the lens. This was likely due to their narrower field of view, making the image more magnified and increasing the number of pixels used to make up the image. The longer focal length lenses also usually had a higher f number, resulting in a decrease in spherical aberrations. As all lenses were plano convex, this would result in greater focus at the expense of intensity of light reaching the sensor.
For all lenses, the USAF 1951 target was used. The FLIR Lepton 3.5 camera has a wavelength range from 8 µm to 14 µm, making it completely within the long wave infrared region.

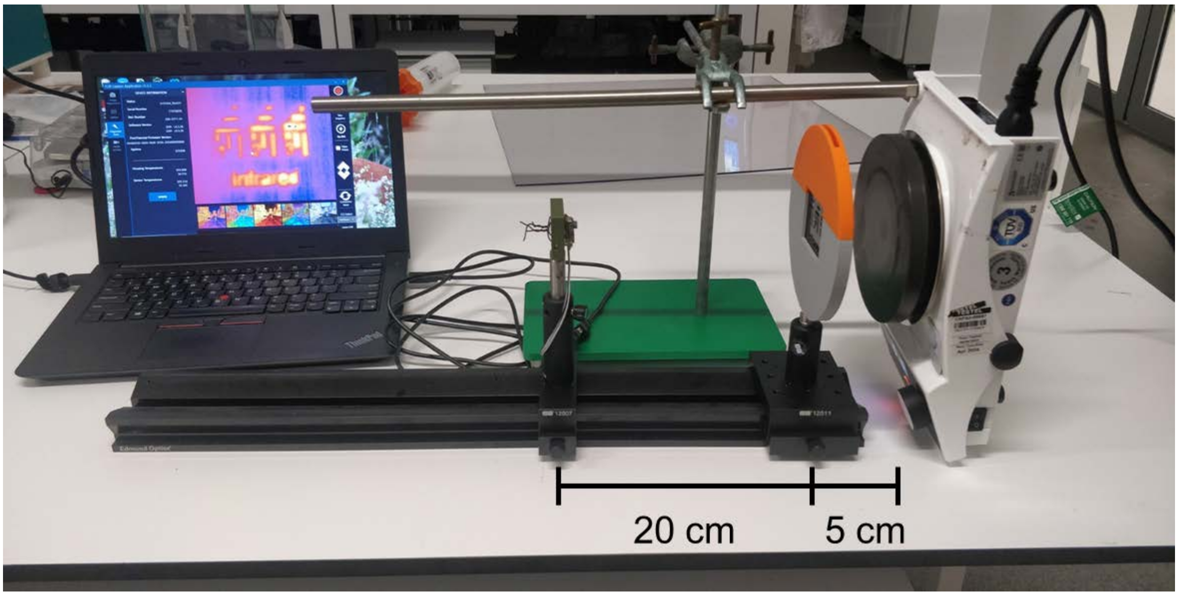

Figure S81: Setup used for focus testing with a 100 °C hotplate. The laser cut mask was placed 5 cm from the hotplate and the FLIR Lepton 3.5 was placed 20 cm from the mask.

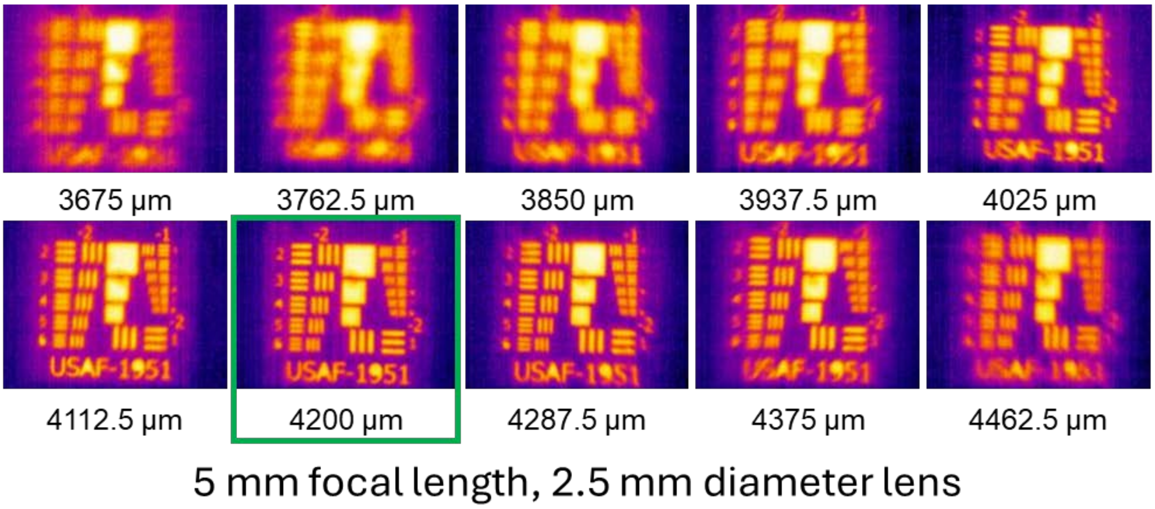

Figure S82: Focus testing using lens 7 on a FLIR Lepton 3.5. The distance from the back surface of the lens to the camera sensor was varied with the optimum focus found at 4200 µm. Testing was done with a mask placed 5 cm from a 100 °C hotplate and the thermal camera placed 20 cm from the mask.

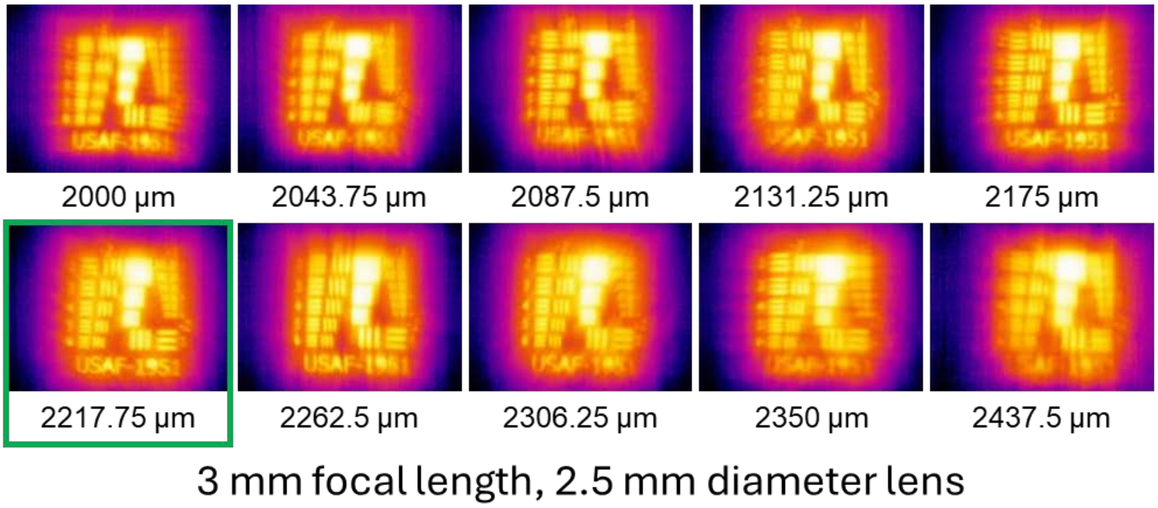

Figure S83: Focus testing using lens 6 on a FLIR Lepton 3.5. The distance from the back surface of the lens to the camera sensor was varied with the optimum focus found at 2262.5 µm. Testing was done with a mask placed 5 cm from a 100 °C hotplate and the thermal camera placed 20 cm from the mask.

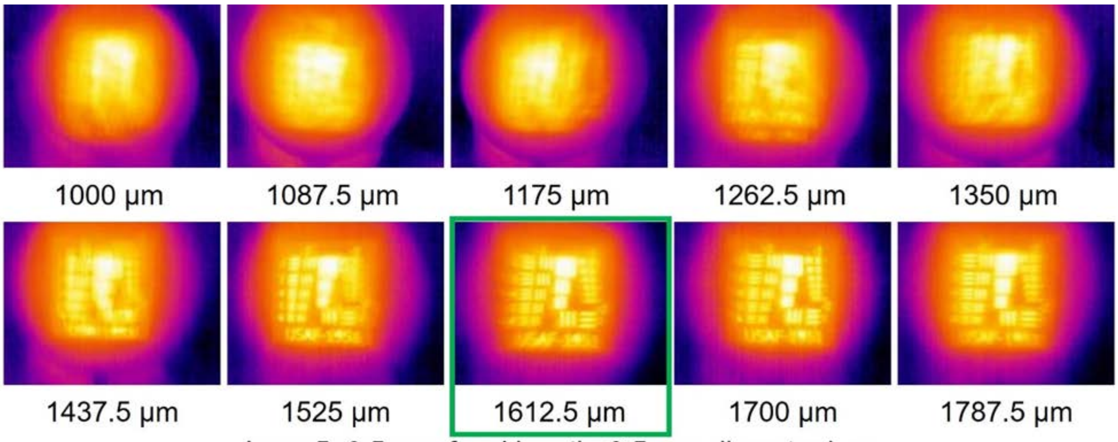

Figure S84: Focus testing using lens 5 on a FLIR Lepton 3.5. The distance from the back surface of the lens to the camera sensor was varied with the optimum focus found at 1612.5 µm. Testing was done with a mask placed 5 cm from a 100 °C hotplate and the thermal camera placed 20 cm from the mask.

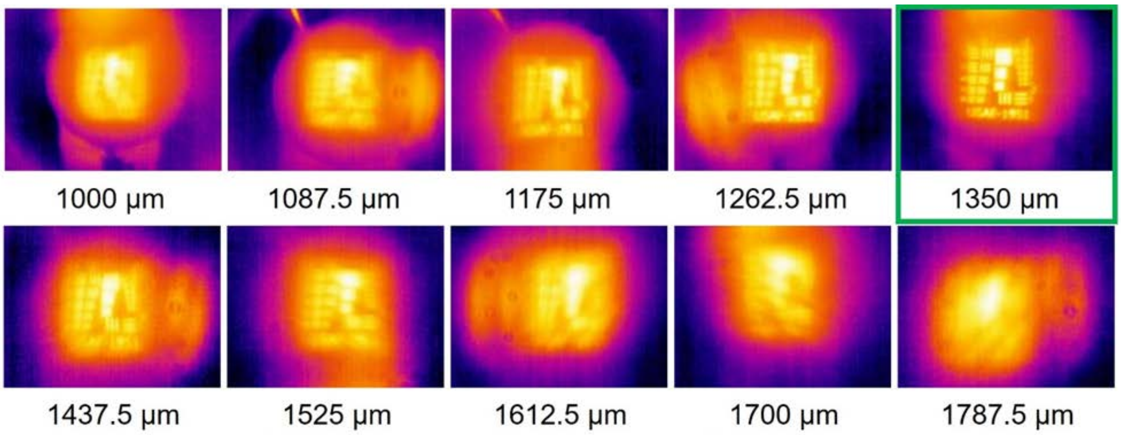

Figure S85: Focus testing using lens 4 on a FLIR Lepton 3.5. The distance from the back surface of the lens to the camera sensor was varied with the optimum focus found at 1350 µm. Testing was done with a mask placed 5 cm from a 100 °C hotplate and the thermal camera placed 20 cm from the mask.

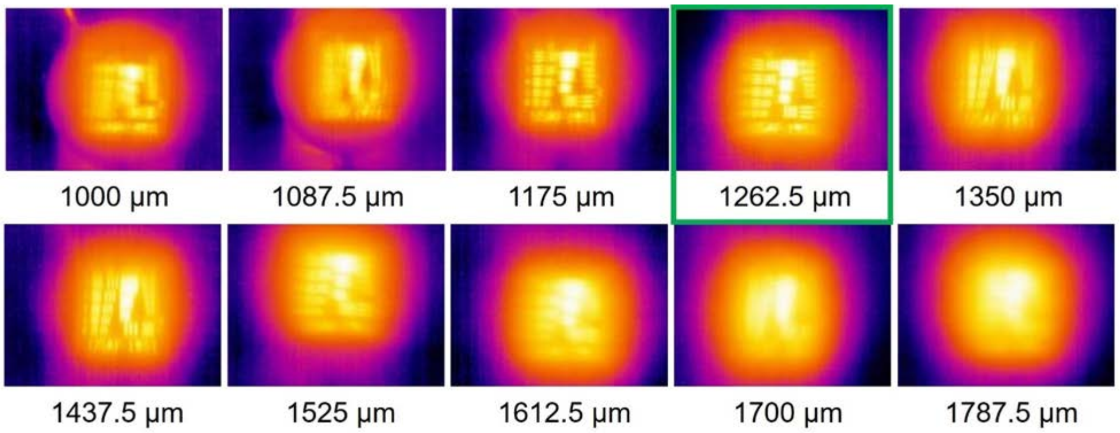

Figure S86: Focus testing using lens 3 on a FLIR Lepton 3.5. The distance from the back surface of the lens to the camera sensor was varied with the optimum focus found at 1262.5 µm. Testing was done with a mask placed 5 cm from a 100 °C hotplate and the thermal camera placed 20 cm from the mask.

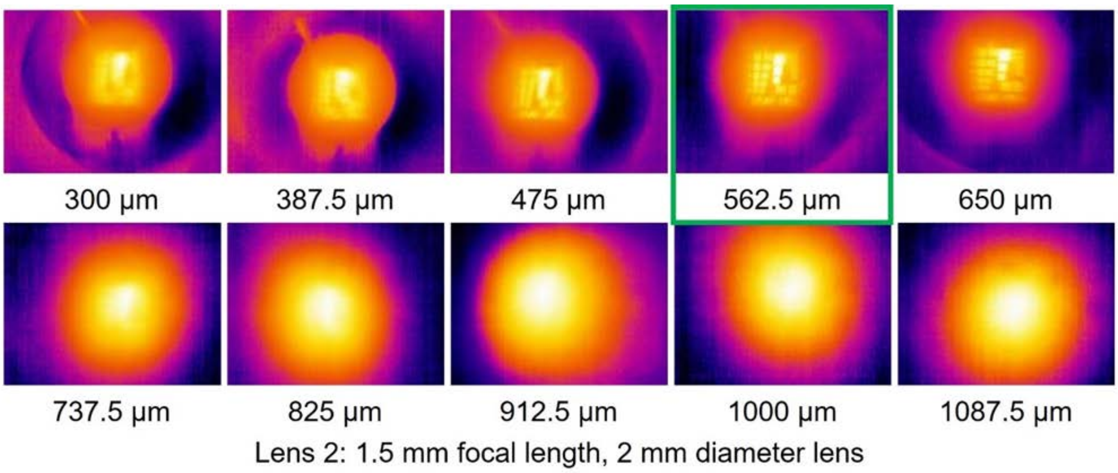

Figure S87: Focus testing using lens 2 on a FLIR Lepton 3.5. The distance from the back surface of the lens to the camera sensor was varied with the optimum focus found at 562.5 µm. Testing was done with a mask placed 5 cm from a 100 °C hotplate and the thermal camera placed 20 cm from the mask.

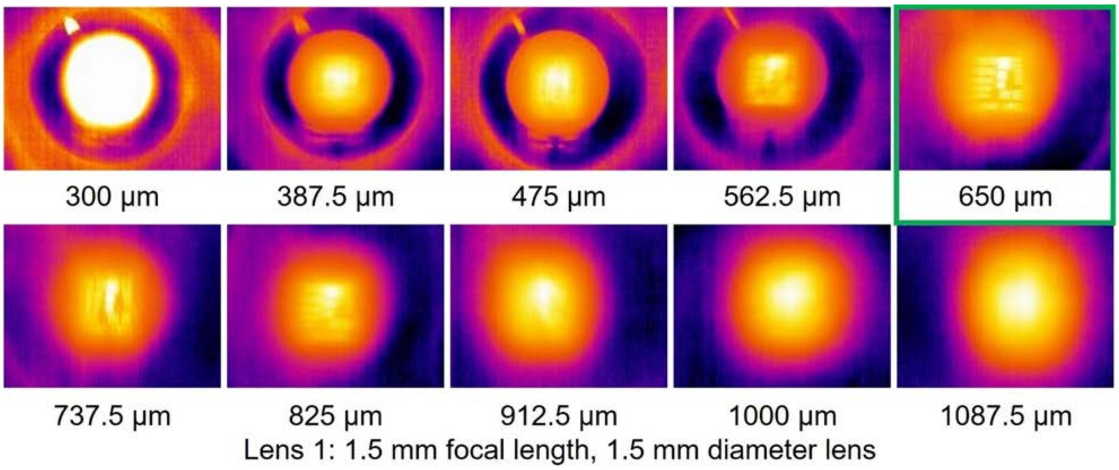

Figure S88: Focus testing using lens 1 on a FLIR Lepton 3.5. The distance from the back surface of the lens to the camera sensor was varied with the optimum focus found at 650 µm. Testing was done with a mask placed 5 cm from a 100 °C hotplate and the thermal camera placed 20 cm from the mask.

Lens resolution testing

The resolution of the lenses and FLIR Lepton 3.5 was determined using the USAF-1951 mask. The resolution was calculated by finding the smallest group of lines which were resolved when imaging the USAF-1951 target at 100 °C using the lens. The set was deemed to be resolved if the dark space between each line was visible. The horizontal and vertical resolution were calculated separately using the vertical and horizontal lines respectively. The object space resolution in line pairs per mm was determined by referring to table S4 of the smallest resolvable set of lines. This value was then converted to angular resolution by finding the angle made between the smallest resolvable line pair and the distance from the sensor to the mask. The silicon doublet control and lenses 3, 4, 5 and 6 were all taken at a distance of 20 cm from the mask. Due to the narrow field of view when using lens 7, it had to be imaged at a distance of 30 cm from the mask. Lenses 1 and 2 were imaged at a distance of 15 cm from the mask due to their wide field of view. To find the image space resolution, the number of pixels used to resolve the smallest line pair was counted using Autodesk Inventor. The FLIR Lepton 3.5 has a sensor resolution of 160 x 120 pixels and a pixel size of 12 µm (0.012 mm). The image space resolution was converted to line pairs per mm by calculating the inverse of the number of pixels multiplied by the pixel size in mm. As expected, the object space resolution was greatest in the lenses with a longer focal length with lens 7 possessing the greatest resolution. This would be expected as it had the narrowest field of view. The image space resolution of all lenses was very similar, ranging from 19.36 lp/mm to 26.56 lp/mm. It should be noted that all lenses were focused on the center of the mask. There was some field curvature observed which led to reduced focus on the edges of the images. This was most noticeable in lenses 1 and 2 and the effect seemed to be most prominent in the lenses with lower f number as expected. To improve the field curvature, more optimized lens designs could be used.

Table S5: Vertical and horizontal object space resolution and object space angular resolution for polymer **1** lenses and a silicon control.

	**Vertical**		**Horizontal**	
**Lens**	**Object space resolution (lp/mm)**	**Object space angular resolution (°)**	**Object space resolution (lp/mm)**	**Object space angular resolution (°)**
**Silicon**	0.31	0.91	0.28	1.02
**7**	0.45	0.43	0.50	0.38
**6**	0.40	0.72	0.40	0.72
**5**	0.31	0.91	0.40	0.72
**4**	0.25	1.15	0.25	1.15
**3**	0.25	1.15	0.25	1.15
**2**	0.25	1.53	0.25	1.53
**1**	0.25	1.53	0.25	1.53

Table S6: Vertical and horizontal image spatial resolution in pixels per line pair and line pairs per mm.

	Vertical		Horizontal	
**Lens**	**Image space spatial resolution (pixels/lp)**	**Image space spatial resolution (lp/mm)**	**Image space spatial resolution (pixels/lp)**	**Image space spatial resolution (lp/mm)**
**Silicon**	3.16	26.39	3.32	25.11
**7**	3.70	22.50	3.14	26.56
**6**	4.24	19.64	4.30	19.36
**5**	4.15	20.06	3.32	25.07
**4**	3.47	24.01	3.45	24.14
**3**	3.51	23.74	3.49	23.90
**2**	4.10	20.34	4.20	19.86
**1**	3.82	21.80	3.80	21.93

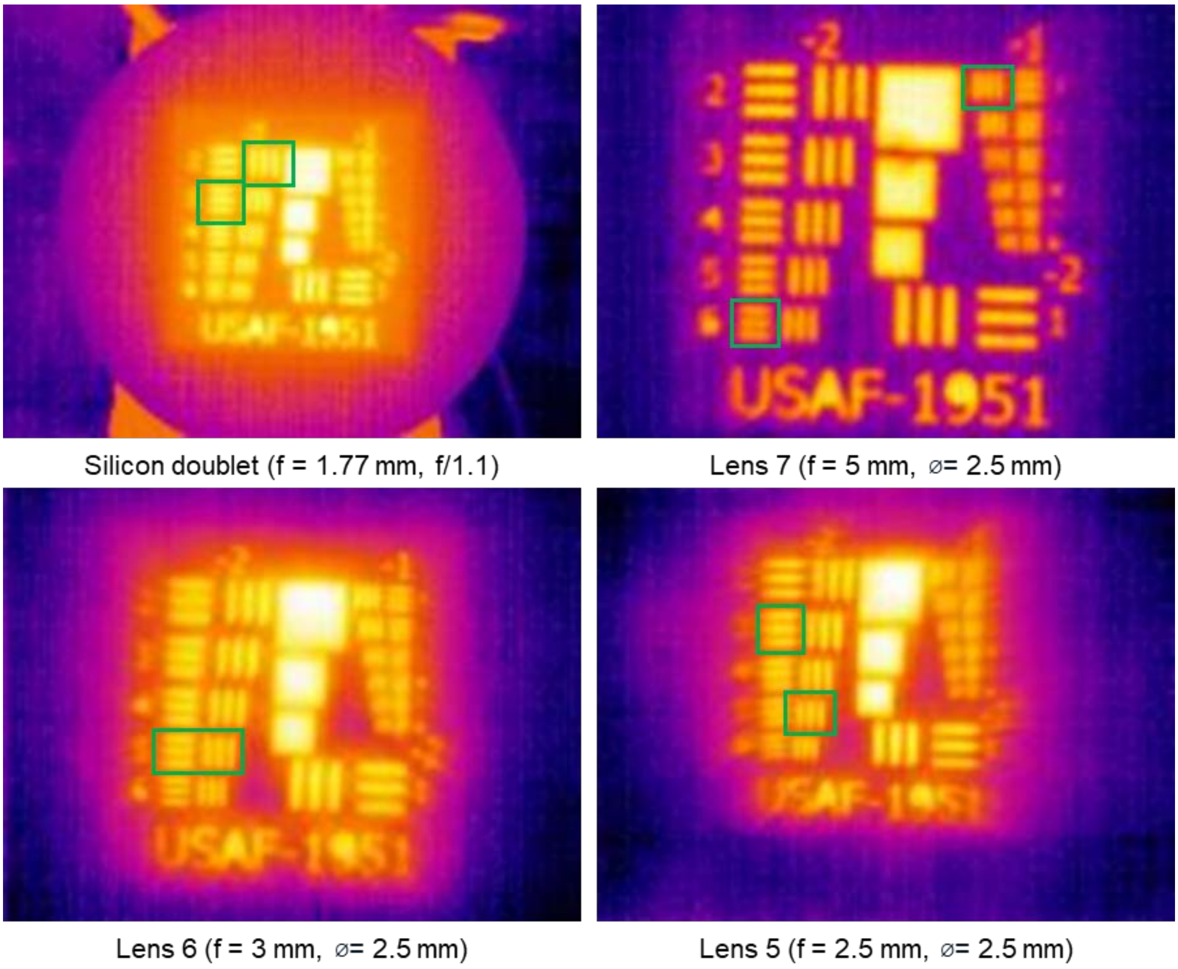

Figure S89: Images of USAF-1951 target taken by FLIR Lepton 3.5 using the silicon doublet lens or polymer **1** lenses 7, 6 and 5. All images used a hotplate temperature of 100 °C. Image taken using lens 7 was taken 30 cm from the mask, all other images were taken 20 cm from the mask.

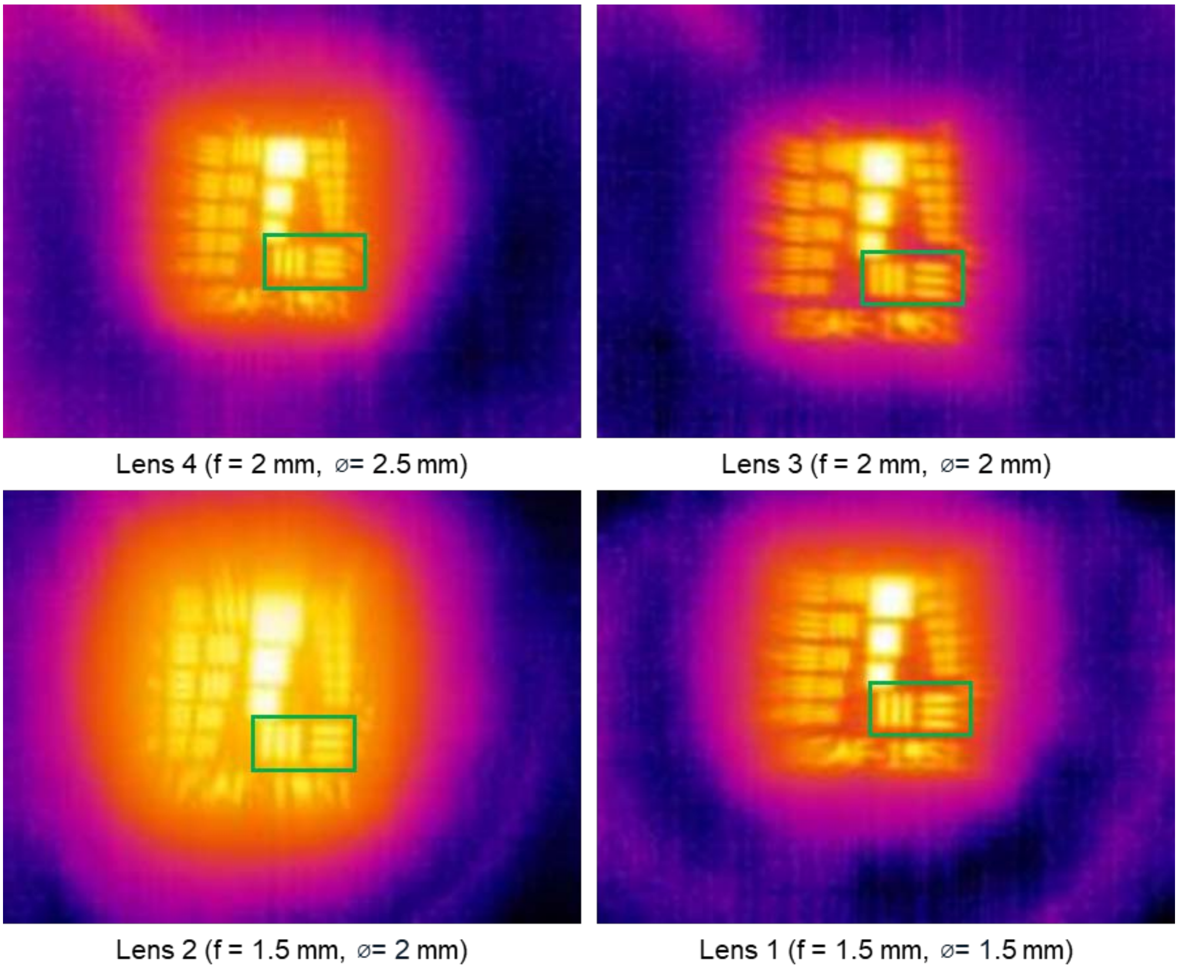

Figure S90: Images of USAF-1951 target taken by FLIR Lepton 3.5 using polymer **1** lenses 4, 3, 2 and 1. All images used a hotplate temperature of 100 °C. Images taken using lens 4 and 3 was taken 20 cm from the mask, images taken using lens 2 and 1 were taken 15 cm from the mask.

Thermal sensitivity testing

The same setup as the focus testing was used for thermal sensitivity testing. However, in this case, the lens was maintained at the optimum focus using the mount and instead, the temperature of the hotplate was varied. A LWIR image was taken every 10 °C between 30 °C and 100 °C. For all tests, the room temperature was approximately 25 °C. The thermal sensitivity testing was only quantitative as access to the intensity values for the FLIR Lepton was not possible, but it showed the lowest temperature differential at which clear images can be taken with the camera using the lenses made from polymer **1**. The lenses with a lower f-number had greater thermal sensitivity. This can be seen most clearly with lens 7 which had a f-number of 2 while all other lenses had a f-number below 1.2. Lens 7 did not have the same contrast as the other lenses at low temperatures.

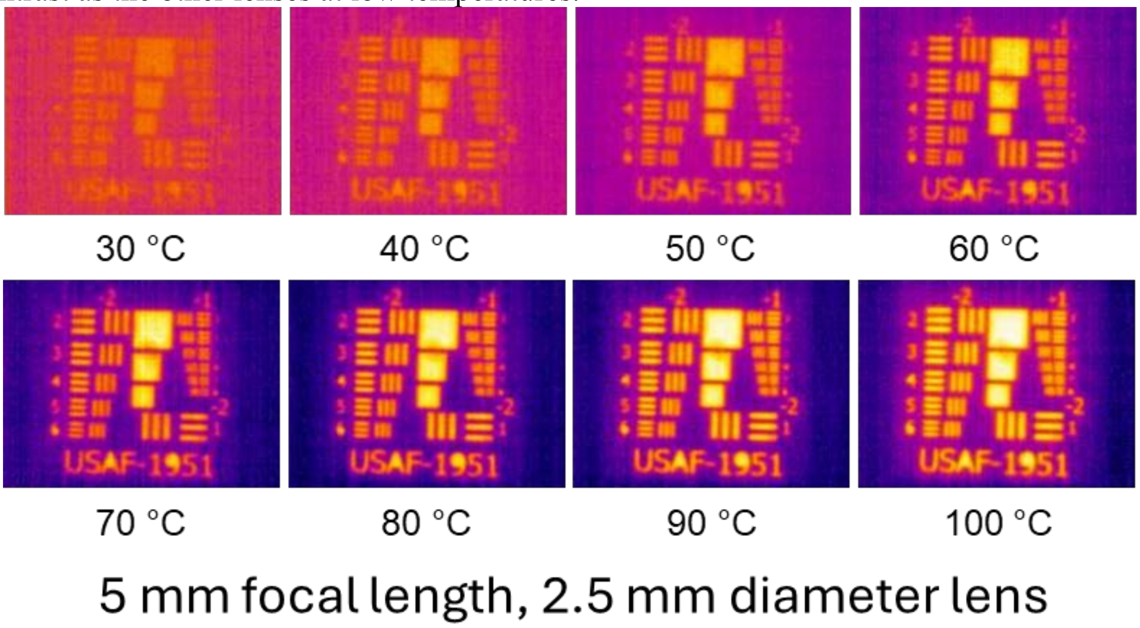

Figure S91: Thermal sensitivity testing of lens 7 on a FLIR Lepton 3.5.

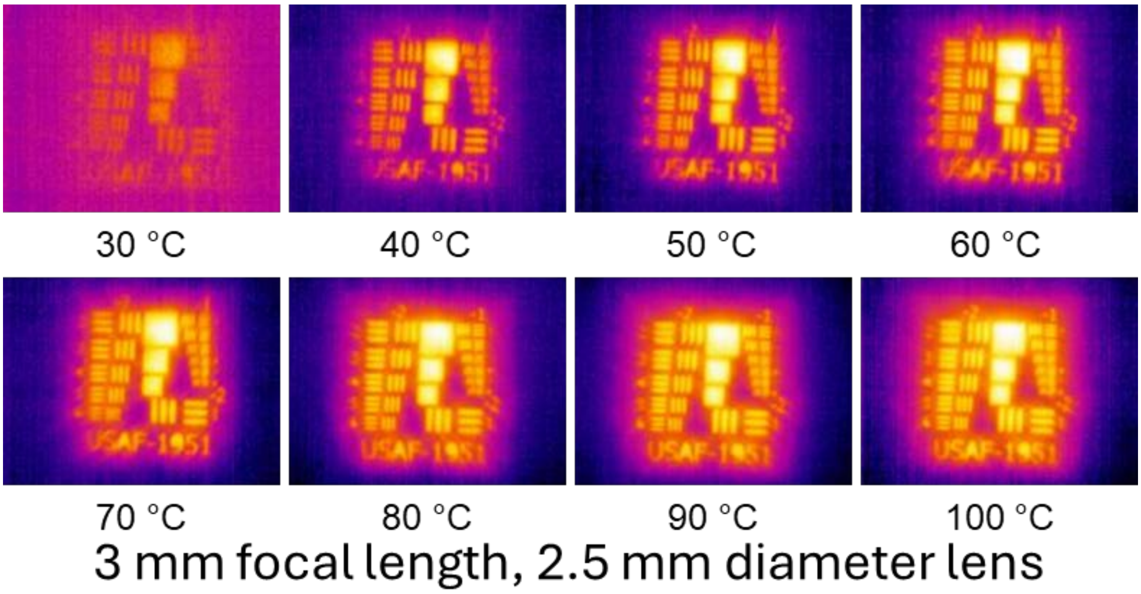

Figure S92: Thermal sensitivity testing of lens 6 on a FLIR Lepton 3.5.

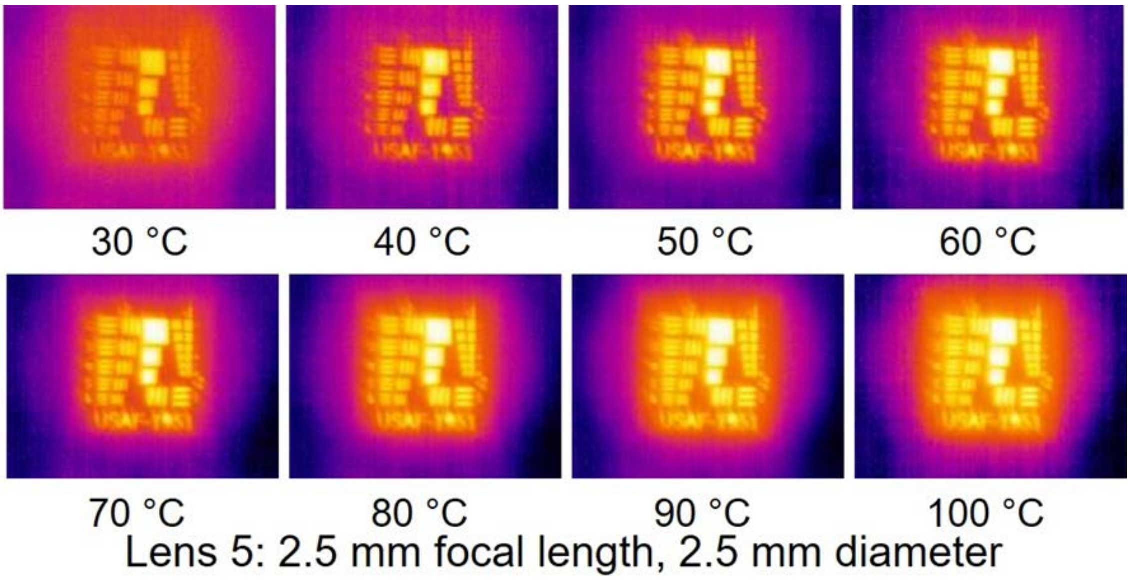

Figure S93: Thermal sensitivity testing of lens 5 on a FLIR Lepton 3.5.

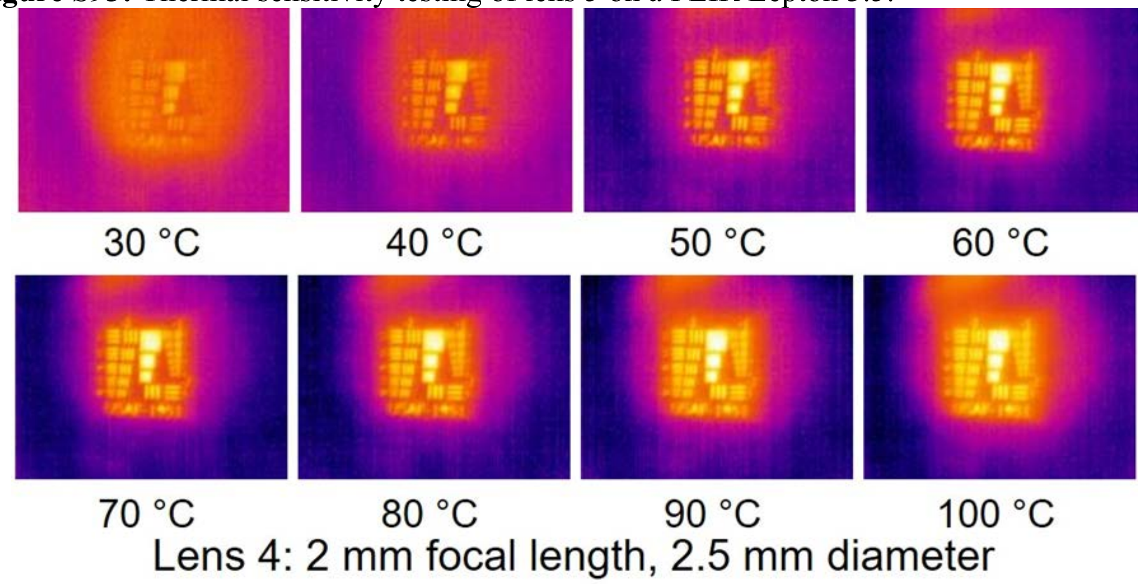

Figure S94: Thermal sensitivity testing of lens 4 on a FLIR Lepton 3.5.

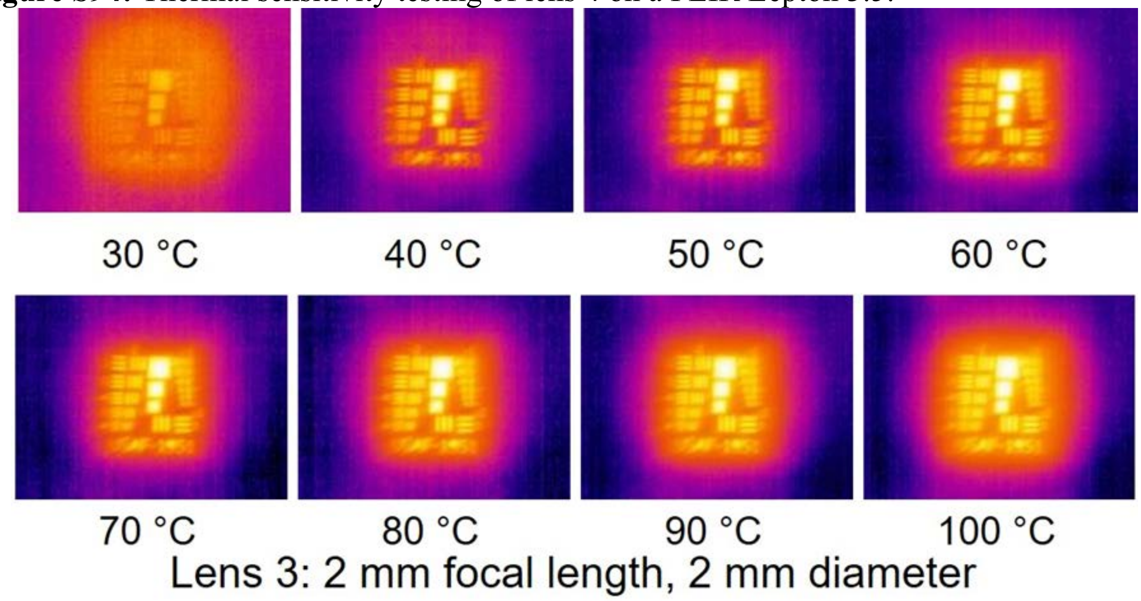

Figure S95: Thermal sensitivity testing of lens 3 on a FLIR Lepton 3.5.

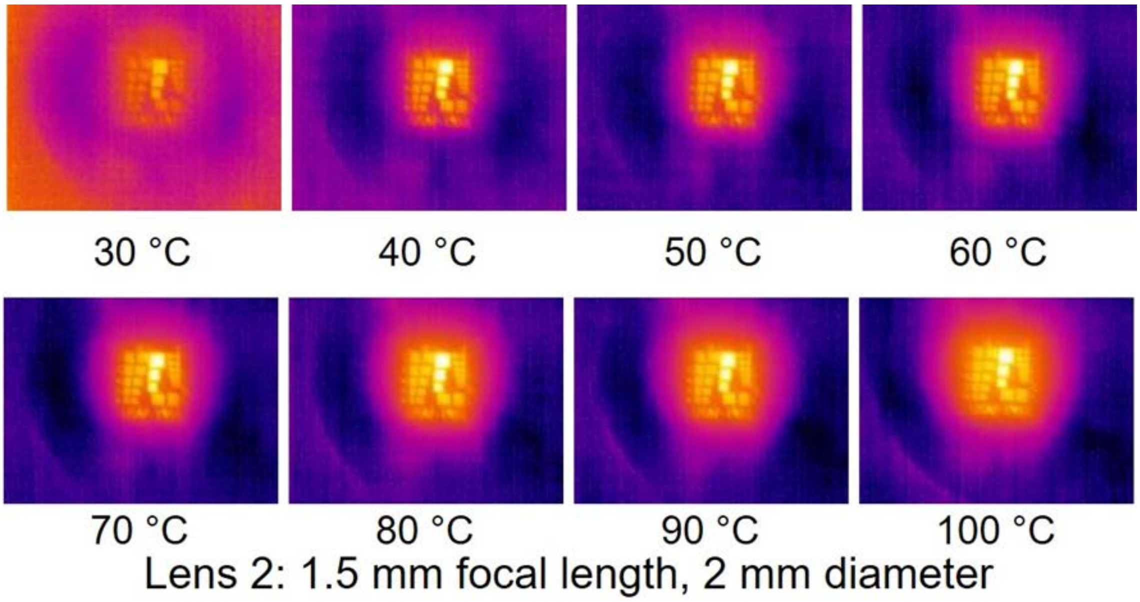

Figure S96: Thermal sensitivity testing of lens 2 on a FLIR Lepton 3.5.

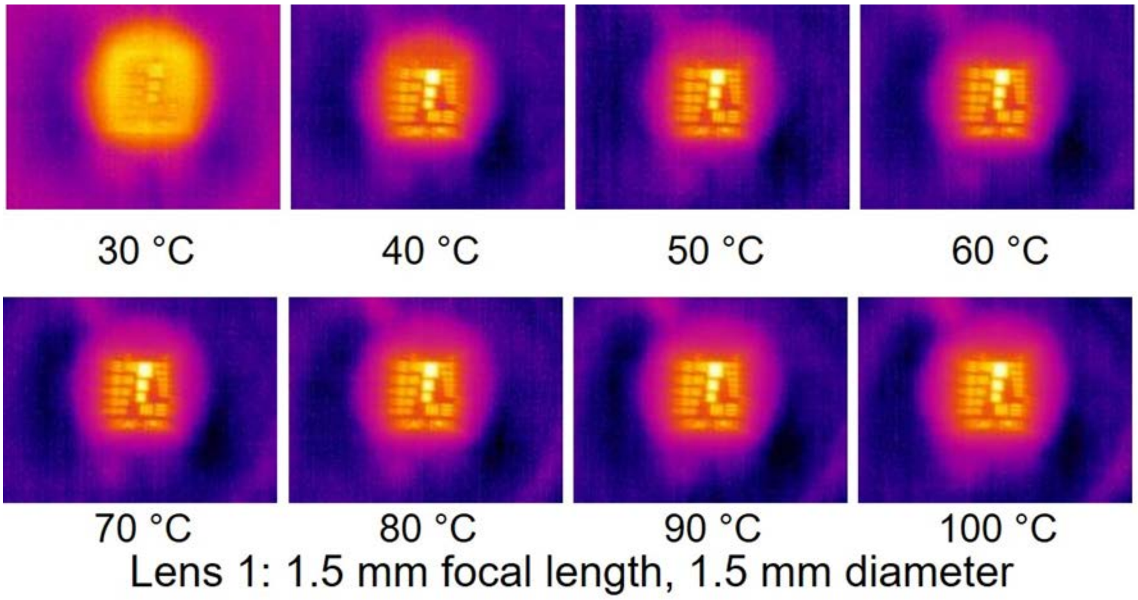

Figure S97: Thermal sensitivity testing of lens 1 on a FLIR Lepton 3.5.

Noise equivalent thermal difference (NETD) testing

To quantitatively compare the performance of the polymer **1** lenses with the stock silicon doublet on the FLIR Lepton 3.5, the noise equivalent thermal difference was measured. The NETD is a measure of a thermal imaging camera's sensitivity, with a smaller temperature difference indicating that the system could detect a smaller change in temperature.

To accurately measure the NETD, a homogeneous blackbody source with an emissivity close to one was required. To achieve this, a square aluminum block was sprayed with an even layer of high emissivity enamel paint. The enamel paint significantly reduced reflections off the surface and reduced the temperature variation across the surface. The coated aluminum block was placed on a hotplate for testing.

NETD testing requires the measurement of temporal noise and signal transfer function of the camera and lens system. The blackbody source was first heated to 30 °C and left to equilibrate. The FLIR Lepton 3.5 was also left for a minimum of 15 minutes so the sensor temperature could equilibrate to reduce thermal drift. In the Lepton user app, the USB video mode was converted to raw images and TLinear was turned off. When using these settings, the FLIR Lepton 3.5 outputs 16-bit tiff images displaying the counts recorded by each pixel without any automatic gain control. The lens was mounted and the thermal camera was placed 15 cm from the blackbody source. A flat field calibration was applied then 100 images of the blackbody source were taken under the same conditions. This process was repeated at 40 °C and 50 °C.

After acquiring the 16-bit images, a Matlab script was used to extract the required data. A 30 by 30 pixel region of interest in the center of the field of view was used for all tests. To determine the signal transfer function, counts from all pixels in the region of interest were averaged over all images. This was repeated for each temperature tested. The signal transfer function was then found from the slope of the average pixel counts against the temperature in kelvin.

To determine the temporal noise, a common-mode removal was applied to all pixels where the region of interest mean was subtracted from each pixel value. This was performed to reduce the effect of thermal drift which would significantly affect the data if left uncorrected. After common mode removal, the 100 images were stacked, and the temporal standard deviation of each pixel was determined. The temporal standard deviation was then averaged for every pixel in the region of interest. This process was repeated at 30 °C, 40 °C and 50 °C and the mean temporal standard deviation from each temperature was averaged to obtain the temporal noise used to calculate the NETD. The NETD was calculated by the temporal noise divided by the signal transfer function. The Matlab script used to perform this data processing is available as supplementary data.

Below is an example image showing the region of interest used for all tests along with the signal transfer function, temporal noise and calculated NETD for all polymer **1** lenses and the stock silicon lens. For all but lenses 6 and 7, the standard shutter was used to perform the flat field calibration before every test. Due to the longer focal length of lenses 6 and 7, the shutter could no longer be placed over the lens. Instead, a custom lens cap was 3D printed and coated with the same high emissivity enamel paint as the blackbody source. To perform the flat field calibration of these lenses, the cap was placed on the lens and left for approximately 5 minutes to equilibrate in temperature with the camera housing. The flat field calibration was then performed, and the cap was removed. Both the average pixel counts, and temporal noise increased when using this cap, likely due to the cap maintaining a lower temperature than the camera housing. When performing the flat field calibration, the FLIR Lepton assumes the cap and housing are equilibrated. However, as NETD measurements are relative, this should not affect the calculated NETD value.

The stock silicon lens is a doublet with an f-number of 1.1 and an antireflective coating on all surfaces. The NETD for this lens was calculated as 53.6 mK, which is close to the reported

value from FLIR. As can be seen in the plot below, lenses 2 and 4 are close to the NETD of the silicon lens with values of 62.1 mK and 63.0 mK respectively. Considering that these are uncoated lenses, this indicates that with an appropriate lens design, the NETD of lenses prepared from polymer **1** could be comparable to a silicon lens. In general, the lenses follow the expected trend of increasing NETD with the square of f-number.

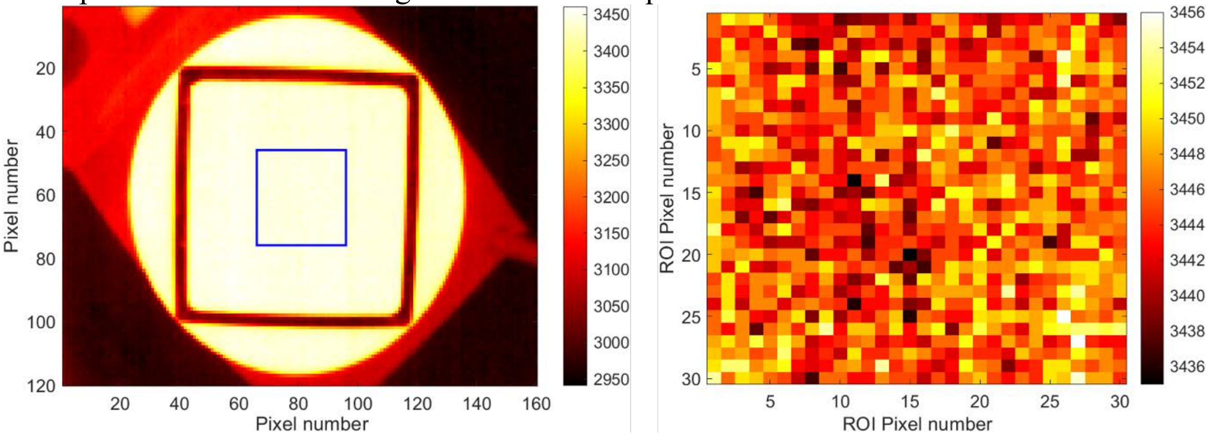

Figure S98: Plot of a single frame taken with stock silicon lens on a FLIR Lepton 3.5 thermal camera. Left image shows all pixels while the right image shows only the region of interest. The color bar shows the number of counts detected by each pixel.

Table S7: Table showing the calculated signal transfer function, temporal noise and NETD for all lenses.

Lens	Signal transfer function (K^{-1})	Temporal Noise	NETD (K)	NETD (mK)
Silicon doublet (f/1.1)	57.8	3.10	0.0536	53.6
Lens 1 (f/1)	29.8	2.95	0.0989	98.9
Lens 2 (f/0.75)	48.6	3.02	0.0621	62.1
Lens 3 (f/1)	23.5	2.98	0.1267	126.7
Lens 4 (f/0.8)	46.1	2.90	0.0630	63.0
Lens 5 (f/1)	23.8	2.98	0.1250	125.0
Lens 6 (f/1.2)	15.9	3.82	0.2397	239.7
Lens 7 (f/2)	8.61	3.51	0.4081	408.1

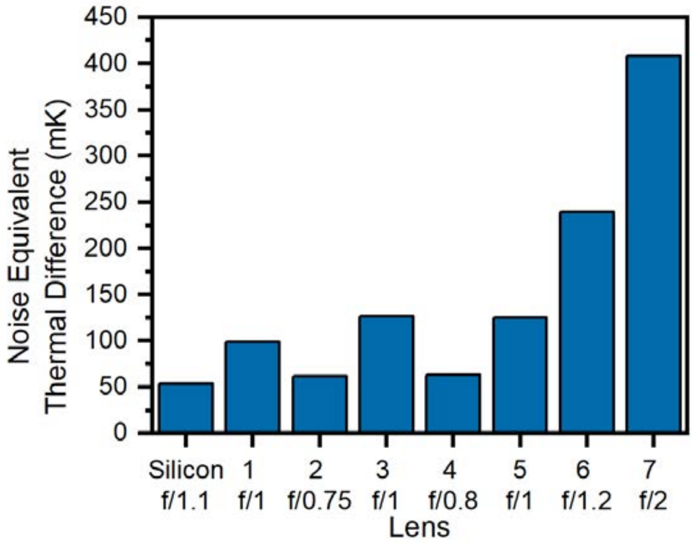

Figure S99: Noise equivalent thermal difference for all lenses tested.

Relative illumination testing

The relative illumination of each lens was tested using the following method. The blackbody source was heated to 50 °C and left for 15 minutes to reach a stable temperature. The lens being tested was mounted onto the FLIR Lepton 3.5 and a flat field calibration was performed. Following this, the thermal camera was brought to within 10 cm so that the blackbody filled the entire field of view and 10 images were taken. To control for the background counts of the detector, a room temperature cap was placed over the lens, and 10 additional images were taken. Using a Matlab script, the images were referenced against the blank and the average pixel value was plotted. To determine the relative illumination, the point with the greatest pixel counts in the image was selected and a horizonal and vertical profile was taken. The counts for every pixel along the profiles were divided by the maximum counts to convert to a relative illumination. Following this, the horizontal and vertical relative illumination was plotted. The Matlab script used to perform this data processing is available as supplementary data.

To compare the relative illumination of the lenses, the pixel value was converted to an angle based on the field of view of the lenses. In general, the relative illumination improved with focal length. This is expected for plano convex lenses due to field curvature dominating in wide field of view lenses. This can be seen in the plot below. The lenses with a shorter focal length (wider field of view) had a greater decrease in relative illumination at the edge of the lens. Improvements in lens design could give a greater relative illumination for these wide-angle lenses. Lenses 5, 6 and 7 performed much better, approaching that of silicon at a narrow angle. This indicates that polymer **1** lenses could perform similarly to silicon in relative illumination with improvements in lens design and consideration of field curvature.

The figure below compares the relative illumination for lenses with the same focal length and corresponding field of view. Lens 1 and 2 have a focal length of 1.5 mm and a horizontal field of view of 65 °. Lenses 3 and 4 have a focal length of 2 mm and a horizontal field of view of 51 °. Lenses 5, 6 and 7 have focal lengths of 2.5 mm, 3 mm and 5 mm corresponding to horizontal field of views of 42 °, 35 ° and 22 ° respectively. The silicon lens has a focal length of 1.77 mm corresponding to a horizontal field of view of 57 °.

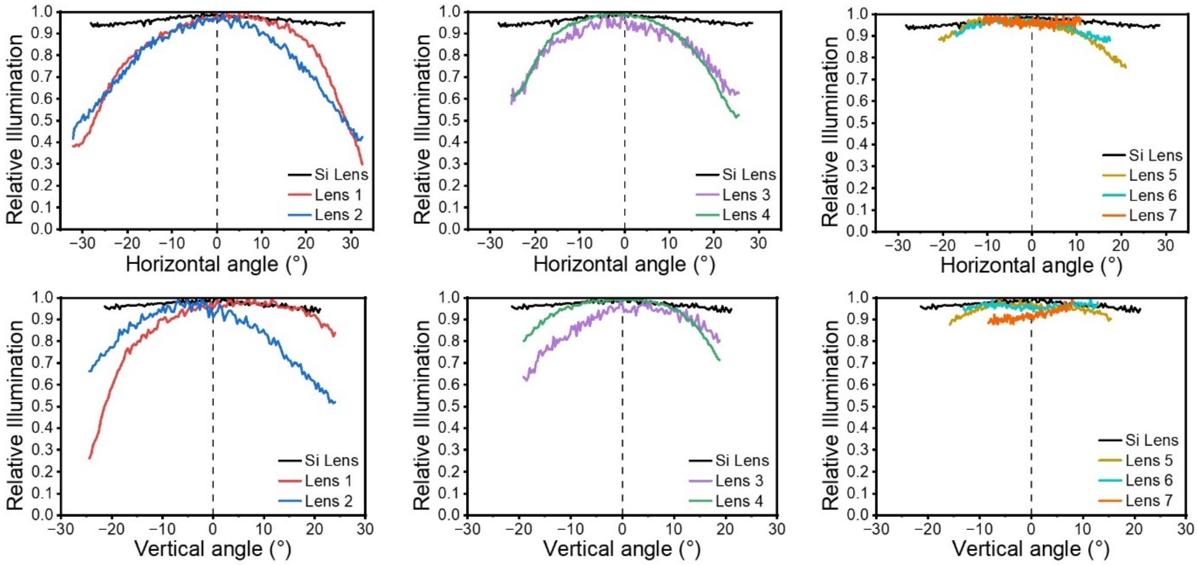

Figure S100: Comparison of horizontal (top) and vertical (bottom) relative illumination between the stock silicon lens and polymer **1** lenses.

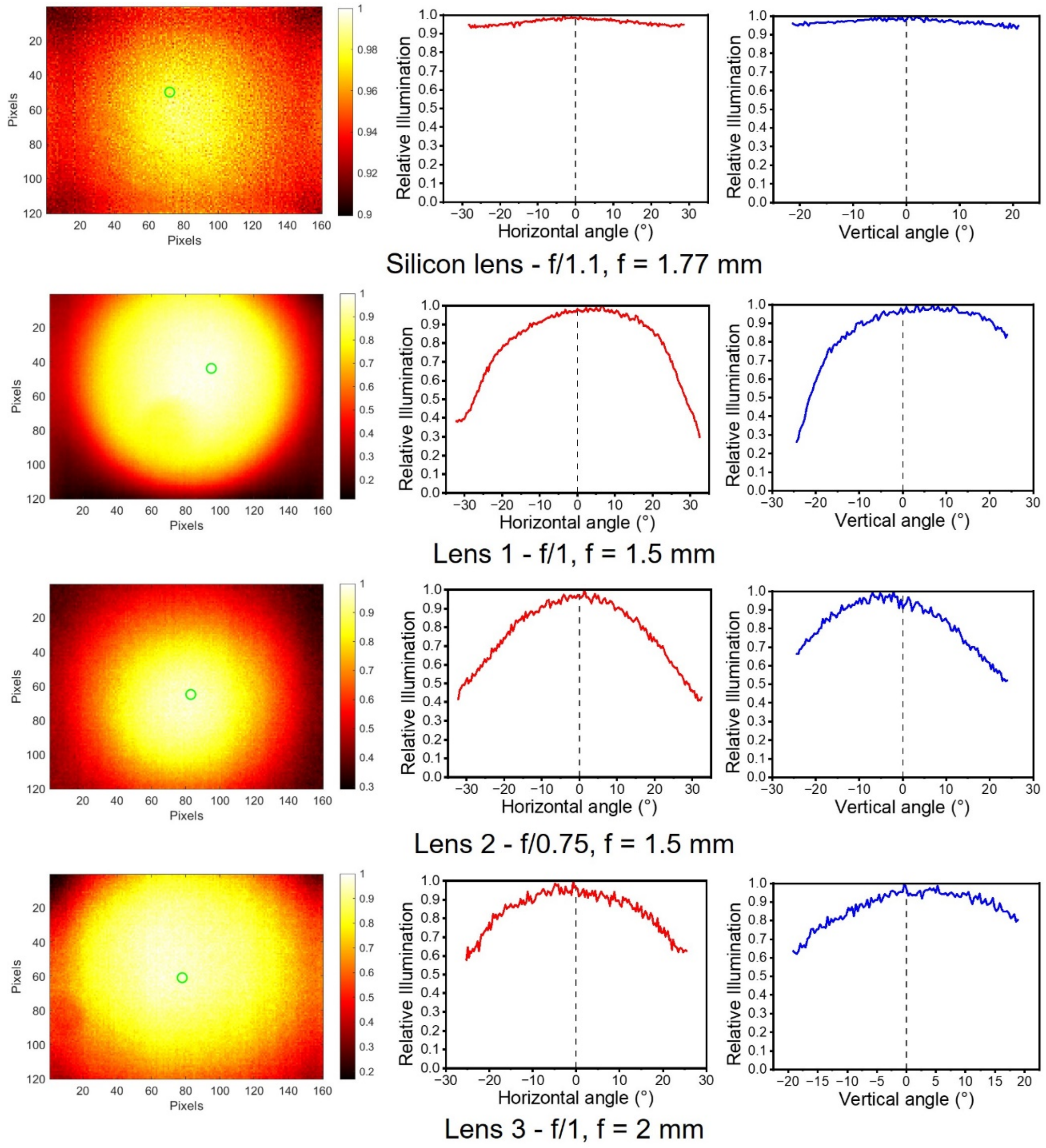

Figure S101: Relative illumination plots for a silicon lens and lens 1, 2 and 3 made from polymer **1** showing a two-dimensional map of relative illumination along with a horizontal and vertical profile from the point with the greatest intensity. All images were taken using a 50 °C blackbody source and referenced against a room temperature lens cap.

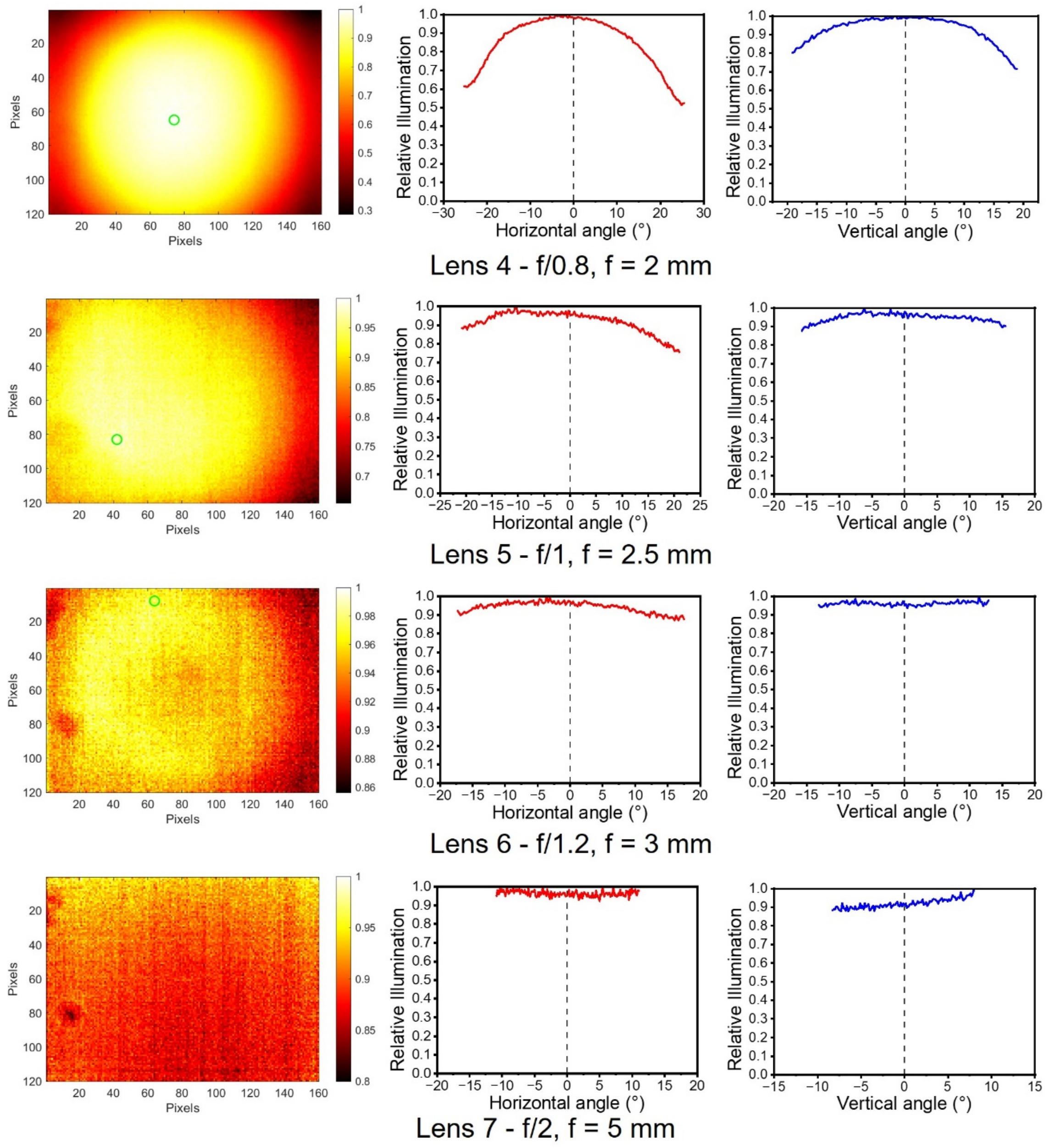

Figure S102: Relative illumination plots for lens 4, 5, 6 and 7 made from polymer **1** showing a two-dimensional map of relative illumination along with a horizontal and vertical profile from the point with the greatest intensity. All images were taken using a 50 °C blackbody source and referenced against a room temperature lens cap.

Overview of imaging using polymer lenses on FLIR Lepton 3.5 module

This section is an overview of all the images taken with each of the lenses on the FLIR Lepton 3.5. The LWIR images of the masks were all taken using the same setup described in the previous two sections. The hotplate was at 100 °C for all the mask images. To go with the images of the masks, images were taken of a person and a hand. The top set of images is a control using the stock silicon doublet. This is an optimized doublet lens made from high quality silicon with an anti-reflection coating on all surfaces. The f-number of this lens was 1.1 and it had a focal length of 1.77 mm. While there was some loss in intensity, the images using the plano convex polymer-based lenses compare very well to the silicon doublet.

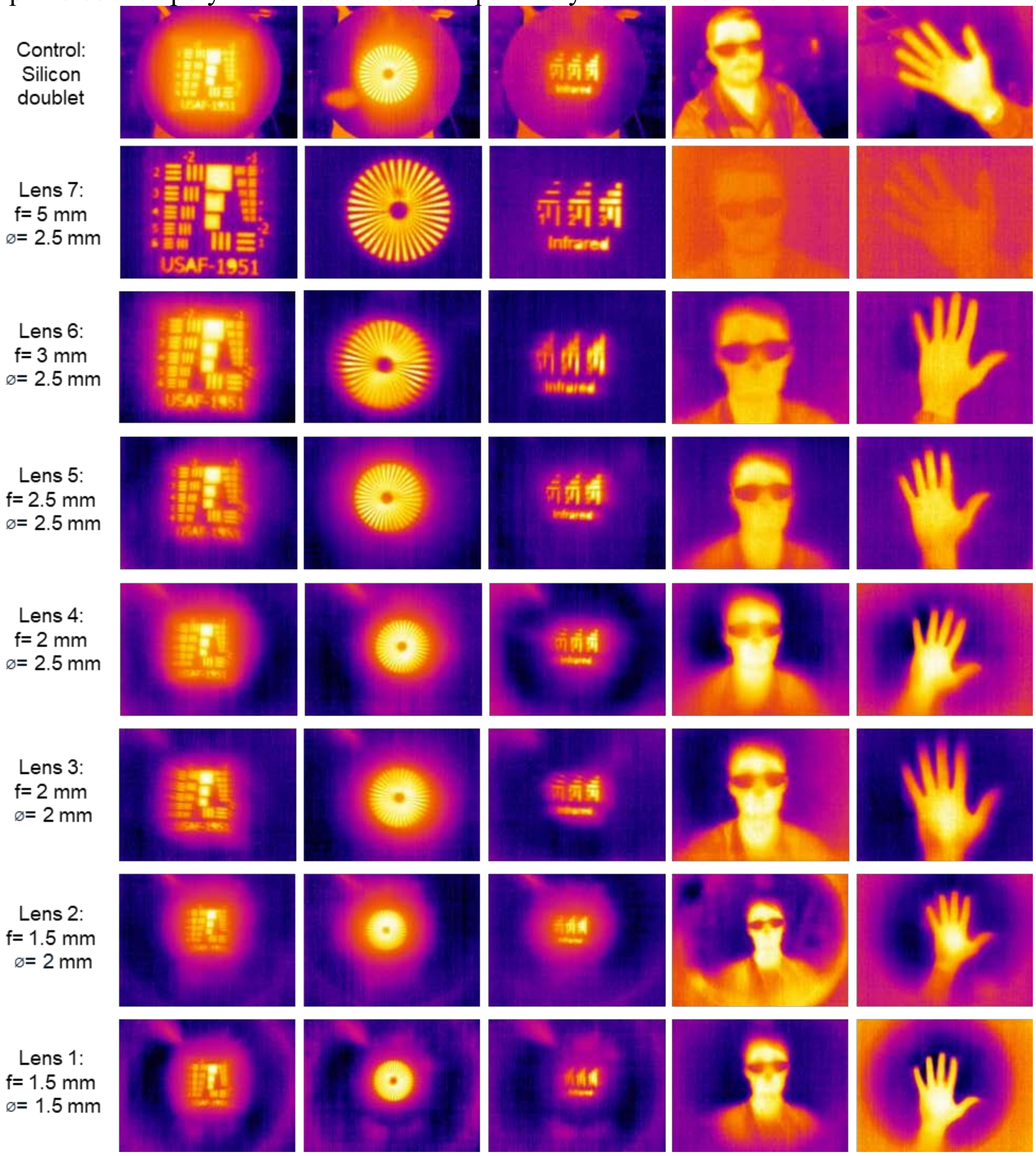

Figure S103: LWIR Images taken with polymer **1** lenses on a FLIR Lepton 3.5 thermal camera. The images of the targets were all taken using a 100 °C hotplate as a black body radiation source. The target was placed 5 cm from the hotplate and the camera was placed 20 cm from the target for all lenses except lens 7 which was placed 30 cm from the mask.

Compression molded lenses

A custom compression mold was designed to demonstrate the scalability of lens manufacture. The same lens design as lens 5 was employed with a focal length of 2.5 mm and a f-number of 1. To give the desired radius of the curved side, an array of 89 lens indents were machined into an aluminum disk. The disk had a flash ring so that excess polymer would flow into a designated area in the die. The polymer was placed on this piece before another disk was placed on top. These pieces made up the die insert. The insert was placed into a cylinder heating block and heated to the compression temperature. A push rod was then placed in the top and the entire die would be compressed using a hydraulic jack. A figure showing the compression mold design can be seen below. This compression die was designed on Autodesk Inventor and machined by the engineering staff at Flinders University from aluminum. To use the compression mold, a total of 2.5 g of polymer **1** was ground into pellets of approximately 1 mm diameter. The flash ring was pressed onto the bottom disk of the insert, the polymer was placed within the flash ring and the top disk was placed on top. Two 65 W heating cartridges and a thermocouple were inserted into holes in the heating cylinder. The push rod and bottom piece were assembled with the heating cylinder, but the insert and polymer were left separate. The die was preheated to 185 °C over approximately 10 minutes. Using heat proof gloves, the push rod was removed, and the insert was added to the preheated die. The push rod was replaced on top, and the press was left for 1 minute for the temperature in the insert to equilibrate. The die was transferred to a hydraulic press and compressed to 20 MPa for a total of 2 minutes. The die was then removed from the press and the insert was extracted. Without disassembling the insert, it was transferred to a pair of water-cooled aluminum plates. The insert was cooled for an additional seven minutes, returning to approximately room temperature. The total cycle time was 10 minutes. However, the cylinder and press rod are not cooled so multiple inserts could be used to reduce the cycle time to below 5 minutes.

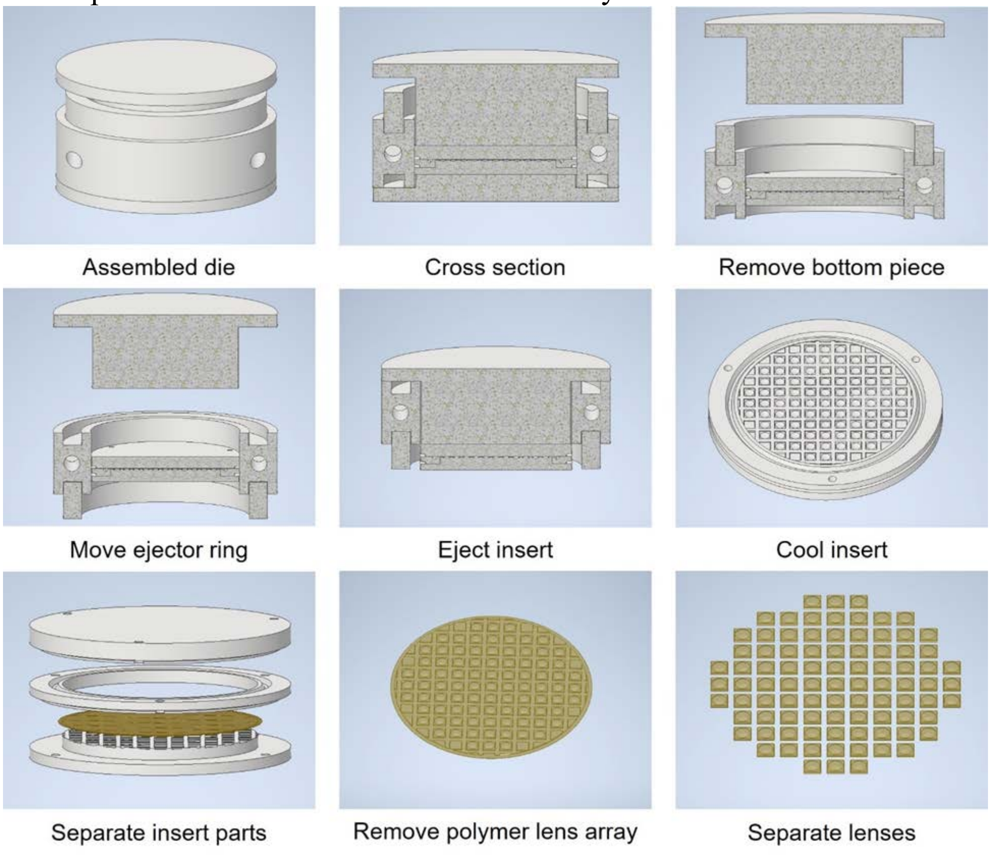

Figure S104: Design and disassembly of heated compression mold on Autodesk Inventor.

To remove the polymer disk from the insert, the top disk was removed. The flashing was separated using a scalpel and the flash ring was pried off the bottom disk. The polymer **1** disk would usually stay within the flash ring as it was removed. A 3D printed tool was used to gently remove the polymer disk from the flash ring. Following removal from the press, the flat side of the disk was polished with a range of micromesh sandpapers to achieve an optical finish. The lenses were then separated. The lenses prepared using the compression mold had a square carrier material, rather than a circular carrier material like the cast lenses. A slightly modified mount was used to accommodate the change in shape of the lens. The compression molded lens was mounted on the FLIR Lepton 3.5 and the same imaging experiments were performed as the cast lenses.

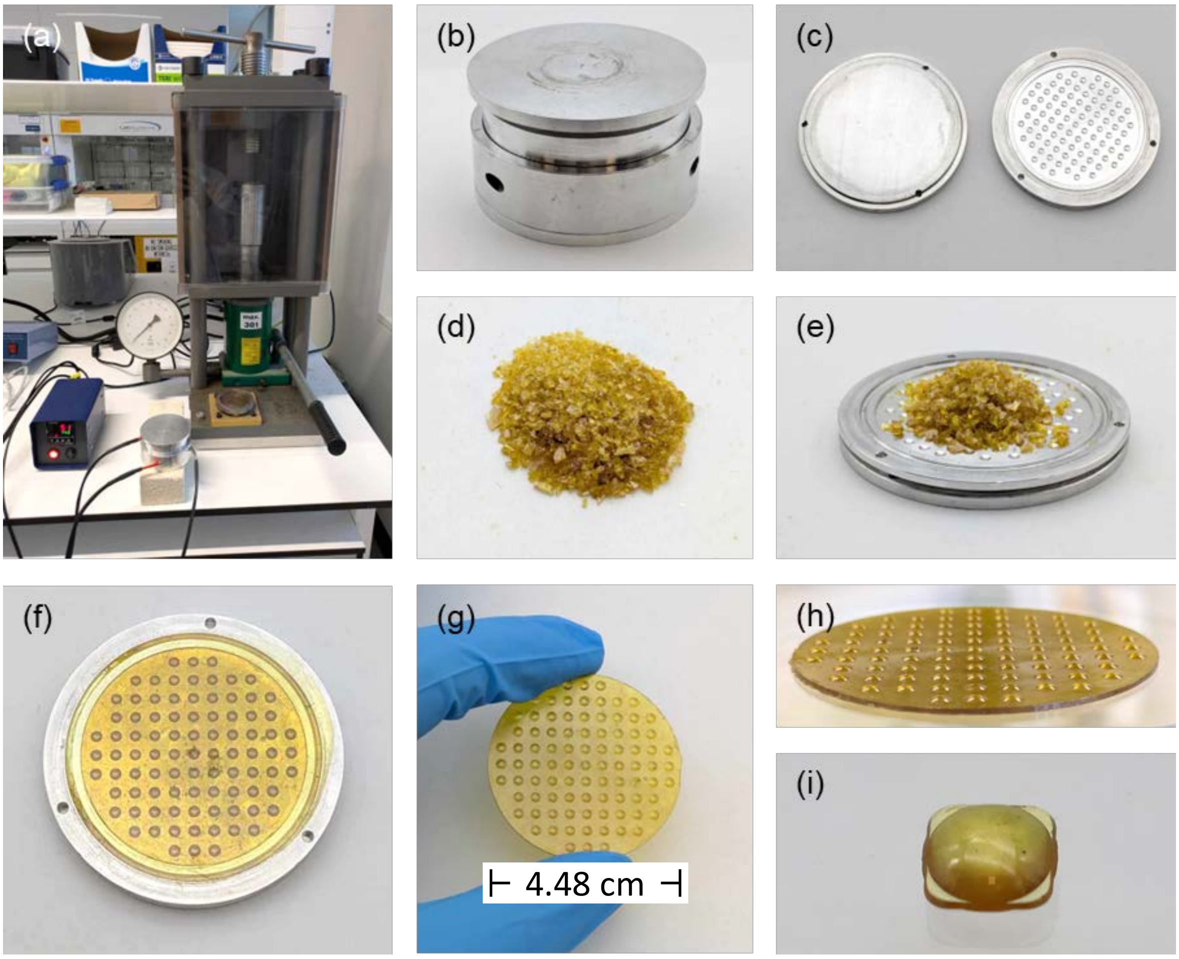

Figure S105: Compression molded lenses made from polymer **1**. (a) Hydraulic press used to compress die. (b) Aluminum die used for compression molding. (c) Insert for compression mold. (d) 2.5 g of Polymer **1** after being ground into pellets with an approximate diameter of 2 mm. (e) Polymer **1** pellets placed on insert before compression. (f) Polymer **1** disk after compression before removal from insert. (g-h) Disk of polymer **1** after removal from insert. (i) Individual lens after separation from compression molded lens array.

Imaging with compression molded lenses

The focus, thermal sensitivity and target tests were all repeated using the compression molded lens. It provided high quality lenses which were very similar to the cast lens with the same focal length and f-number.

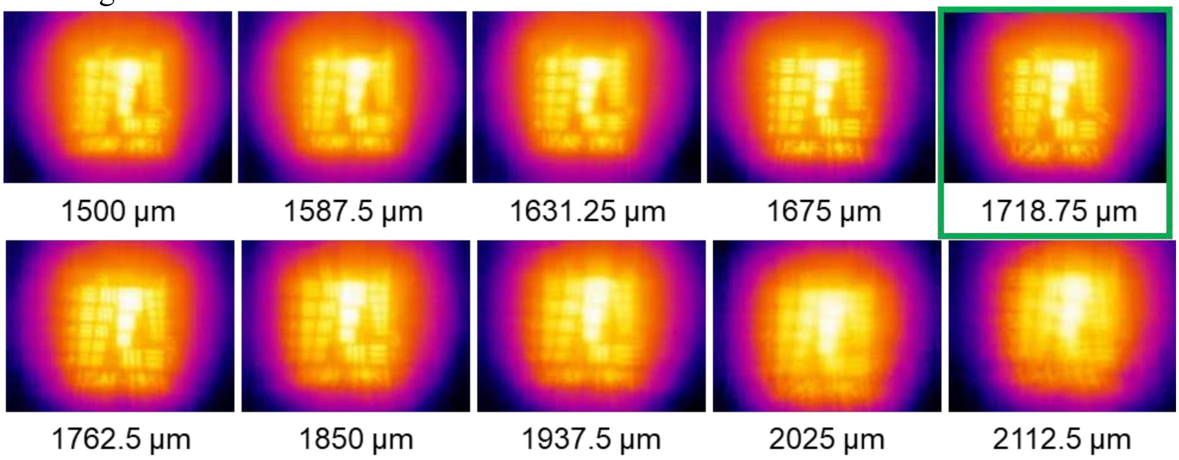

Figure S106: Focus testing using compression molded lens on a FLIR Lepton 3.5. The distance from the back surface of the lens to the camera sensor was varied with the optimum focus found at 1718.75 µm.

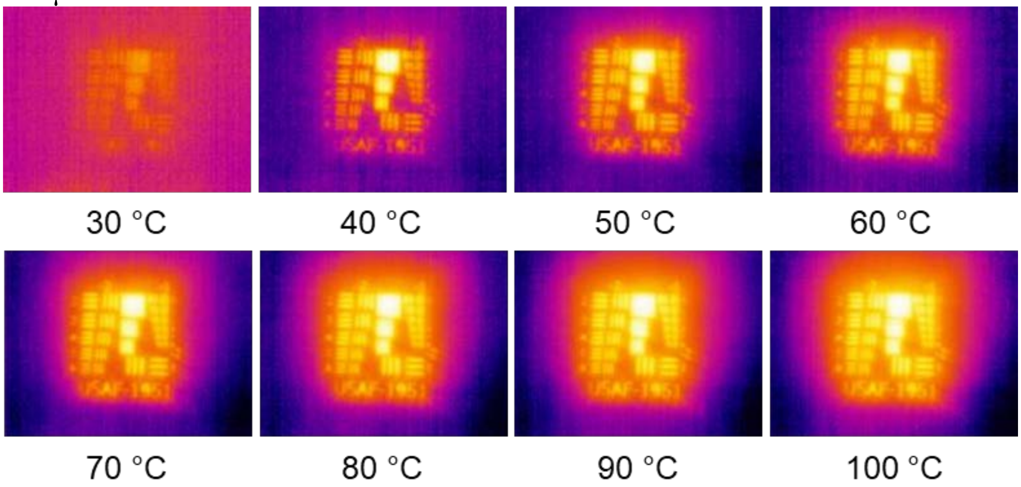

Figure S107: Thermal sensitivity testing of compression molded lens on a FLIR Lepton 3.5.

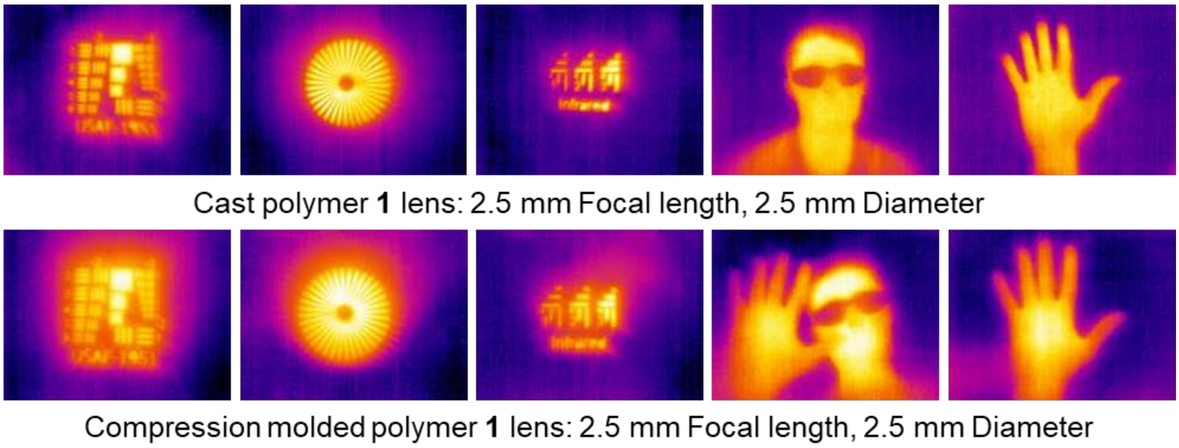

Figure S108: LWIR Images taken with polymer **1** lenses on a FLIR Lepton 3.5 thermal camera. The top lens was cast while the bottom lens was compression molded. The images of the targets were all taken using a 100 °C hotplate as a black body radiation source.

Long-term stability of polymer 1 and polymer 1 lenses

Differential scanning calorimetry and thermal gravimetric analysis were used to investigate the long-term thermal stability of polymer **1**. Both techniques were used on a freshly synthesized polymer sample and a sample that had been stored for 6 months. The polymer samples were stored in a glass vial at room temperature. For DSC analysis, the same sample from the same batch was tested. For TGA analysis, the sample that was stored for 6 months was compared to a freshly synthesized sample that was made using the same method.
For DSC, a TA Q20 DSC DSC was used with the following method. The sample was first held isothermally at 60 °C for 5 minutes to reduce any thermal history. Two full heating cycles were obtained between -60 °C and 200 °C at 10 °C/minute with a 1-minute isothermal at the end of each ramp. A third cycle was also obtained at 20 °C/minute to show the apparent change in glass transition temperature at a higher heating rate. A constant flow of nitrogen at 50 mL/min was applied to both samples throughout the entire run. Both samples showed an almost identical DSC thermogram with no significant change in glass transition. Importantly, no sulfur melting peaks were observed in either sample, indicating no sulfur blooming after storage for 6 months.

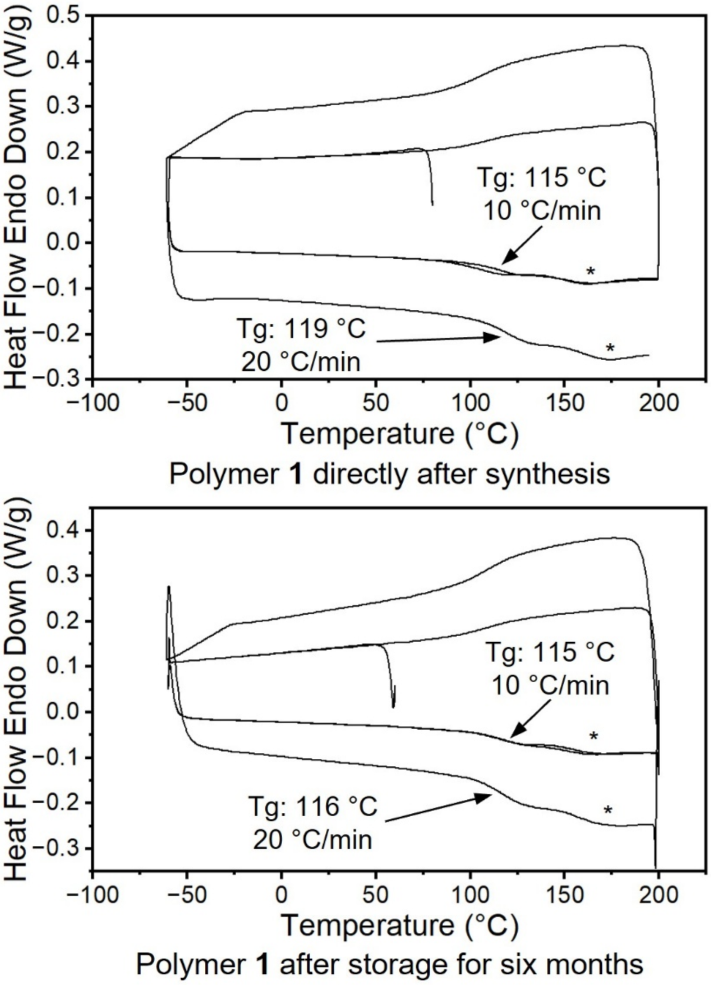

Figure S109: Differential scanning calorimetry thermogram for polymer **1** with a sulfur composition of 81 % using a TA Q20 DSC both directly after synthesis (top) and after storage at room temperature for six months (bottom). Two ramps at 10 °C/min and one at 20 °C/min are displayed. The thermal transition labelled with a star is only observed during heating and occurs at 163 °C and 174 °C with a heating rate of 10 °C/min and 20 °C/min respectively.

For thermal gravimetric analysis, a TGA Perkin Elmer 8000 was used to analyze the thermal degradation of all the polymers. Starting at 30 °C, the sample was heated to 800 °C at 20 °C/min under a 35 mL/min flow of nitrogen. At 800 °C, the gas was switched to air at 20 mL/min to burn off any remaining material.

The only difference in the TGA analysis between the two samples was a slight mass loss starting around 150 °C in the sample analyzed directly after synthesis. While the source of this mass loss is not known, it is possible that this is from water trapped within the polymer structure. Apart from this initial mass loss, both samples demonstrated very similar thermogram profiles.

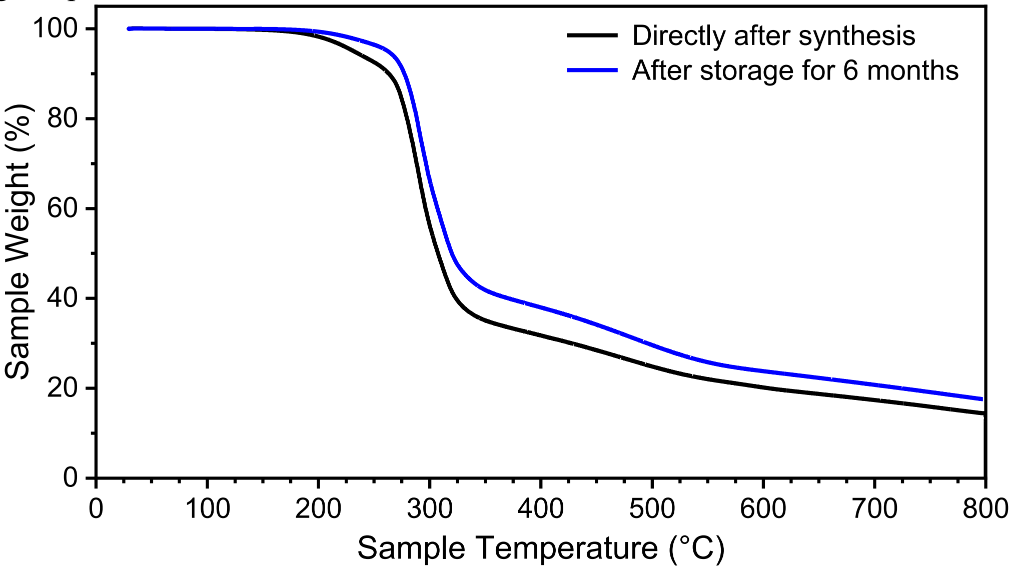

Figure S110: thermal gravimetric analysis thermogram for polymer **1** with a sulfur composition of 81 % using a TGA Perkin Elmer 8000 both directly after synthesis and a sample that was stored at room temperature for six months.

Refractive index testing of polymer 1 batches and aged polymer batches

To test the consistency of the refractive index of polymer 1 batches, six samples were prepared from different batches. The refractive index of these samples was measured using the method described below. After measurement, the samples were stored for over 8 months in a glass vial at room temperature. The samples were then repolished, and the refractive index was measured again using the same method to determine any change in refractive index over time.

To measure the refractive index of the polymer **1** samples, the specular reflectance of the polymer was measured and converted to refractive index using a Kramers-Kronig transform. It was found that the window thickness and surface roughness were vitally important when measuring reflectance. When thin windows were used (less than 2 mm thick), the reflectance spectrum would show a similar pattern to the transmittance spectra. This was likely due to light transmitting through the surface, reflecting off the bottom surface before being collected by the detector. Due to these effects, the sample geometry was carefully considered. Cylinders of polymer **1** were made with a diameter of 25 mm and a thickness of approximately 10 mm. This thickness ensured that very little light would transmit through the entire sample. Only the top face of the polymer window was polished. The polishing process was important to avoid light scattering. Starting at 200 grit, the surface was sanded flat. Next, the sample surface was wet and dry sanded with micro mesh polishing kit with sandpaper grits from 1500 to 12000. A micro gloss polish with a 1-micron abrasive crystal was then applied to the surface. Specular reflectance spectra were measured using a Bruker Vertex v80 instrument with the 1513/QA attachment. An angle from the surface normal of 15 ° was used under vacuum. The sample was referenced using the reflectance spectrum of an aluminum mirror. A Kramers-Kronig transform was applied using the OPUS V7.2 software to find the wavenumber dependent phase shift spectrum. The refractive index was then calculated using the real portion of the complex refractive index, $\eta = n + ik$.

When measured directly after synthesis, the samples had an average refractive index of 1.867 and a standard deviation of 0.015. After storage for over 8 months, the samples had an average refractive index of 1.886 and a standard deviation of 0.0088. The refractive index of the samples remained similar between batches and after storage. This technique was found to be very dependent on the surface polish which likely contributed to much of the difference in refractive index between the samples.

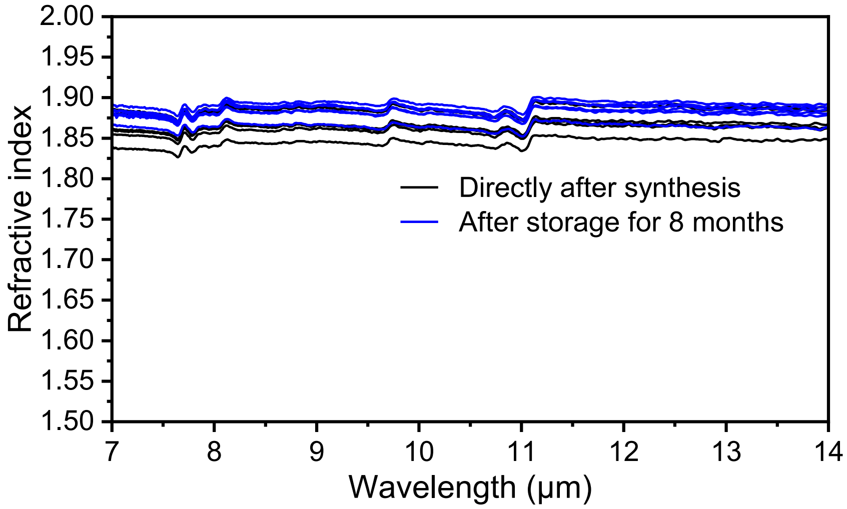

Figure S111: Refractive index spectra of polymer **1** made from sulfur and monomers **9** and **10** with 81 % sulfur. All spectra were obtained on a Bruker Vertex v80 using the 1513/QA attachment.

Imaging with a polymer 1 lens after 12 months of storage

The long-term stability of a polymer **1** lens was tested to investigate any changes in imaging performance. The lens design chosen for the long-term stability test was lens 6 with a focal length of 3 mm and a diameter of 2.5 mm. The lens was made from polymer **1** with 81 % sulfur with the casting method described earlier in this Supplementary Information. The lens was mounted to a FLIR Lepton 3.5 and several images of a hotplate with targets were taken along with images of a person. All images of the hotplate and targets were taken at a distance of 20 cm and a hotplate temperature of 100 °C.

After imaging, the lens was stored at room temperature for more than 12 months. To test any changes in imaging performance, the lens was then remounted to the FLIR Lepton 3.5, and the same images were taken again. There was no change in image quality or resolution. The focal distance from the back of the lens to the sensor was also unchanged, indicating no significant change in refractive index or lens geometry had occurred.

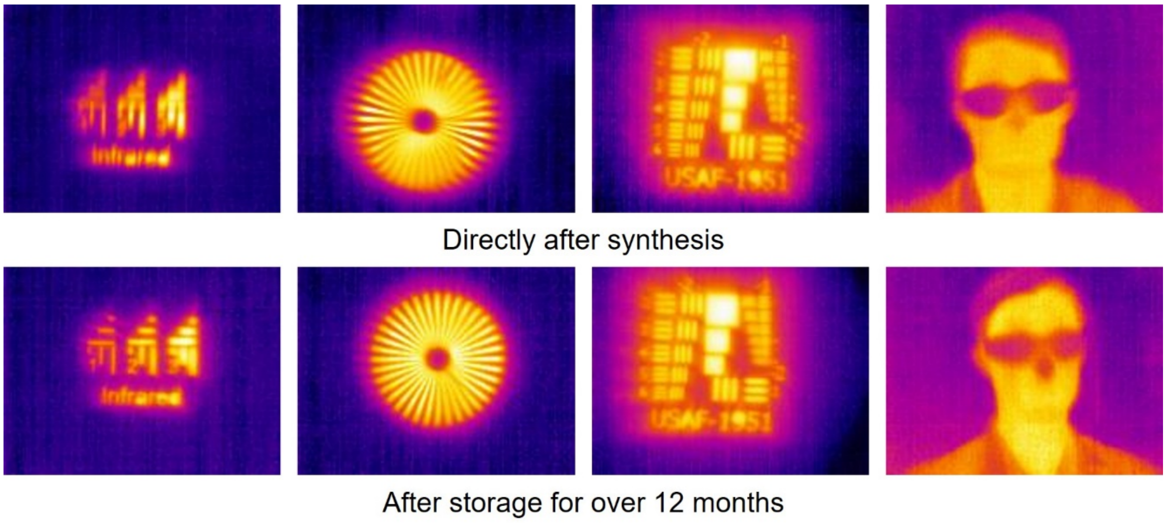

Figure S112: Images taken with lens 6 (f = 3 mm, diameter = 2.5 mm) made from polymer **1** with 81 % sulfur content. The top images were taken directly after the lens was manufactured. The bottom images were taken after storage at room temperature for more than 12 months. All hotplate images were taken from a distance of 20 cm and a hotplate temperature of 100 °C.

Comparative assessment of sulfur derived polymer optics for LWIR imaging

Table S8: Comparison of sulfur derived polymer optical and thermomechanical properties for LWIR imaging (7–14 µm) applications. Only literature reports demonstrating LWIR measurements through ~1 mm windows are included. Polymers that have demonstrated instability, such as sulfur blooming, are omitted.

**Ref.**	**Monomer unit**<br><br>**Polymer name in ref.**	**Sulfur weight based on reaction stoichiometry (%)**<br><br>***based on elemental analysis**<br><br>****based on complete reaction of thiols to form H_2S***	**Window or film thickness (mm)**	**Average LWIR (7–14 µm) transmission (%)**<br><br>***values estimated through image digitization of published spectra, using Origin 2021 software**	**DSC Tg (°C)**	**DMA Tg (°C)**	**Refractive index**<br><br>***values estimated through image digitization of published spectra, using Origin 2021 software**	**Comments**
This work	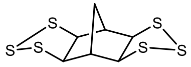<br>**9**	81	1.00	19.4	115	153	1.87 average over 7–14 µm	• LWIR imaging at ambient temperature demonstrated through plano-convex lens serving as the sole refractive optical element in a LWIR camera<br>• Lenses formed through melt casting and compression molding
28	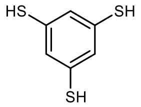<br>**23**<br>poly(S-*r*-BTT)<br>S80-BTT20	92.473*<br>(90.26**)	1.156	32.38<br>31.43*	14.85	40.83	2.00 @ 637 nm<br>1.97 @ 829 nm<br>1.94 @ 1306 nm<br>1.94 @ 1549 nm	• Toxic H_2S production during polymer synthesis<br>• Polymer window made by hot pressing<br>• LWIR imaging of hot plate demonstrated through polymer window<br>• LWIR imaging of human finger demonstrated through polymer window S70-BTT30<br>• Polymer window S70-BTT30 shows no/little decrease in transmission after 30 days of storage at room temperature
28	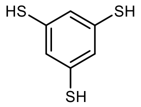<br>**23**<br>poly(S-*r*-BTT)<br>S70-BTT30	87.682*<br>(84.39**)	1.139	26.83<br>25.34*	40.39	64.28	1.99 @ 637 nm<br>1.96 @ 829 nm<br>1.93 @ 1306 nm<br>1.93 @ 1549 nm	
28	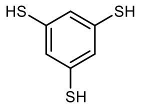<br>**23**<br>poly(S-*r*-BTT)<br>S60-BTT40	82.337*<br>(77.65**)	1.144	19.67*	66.21	96.31	1.97 @ 637 nm<br>1.94 @ 829 nm<br>1.91 @ 1306 nm<br>1.91 @ 1549 nm	

28	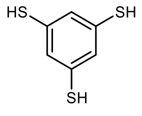 **23** poly(S-*r*-BTT) S50-BTT50	75.120* (69.84**)	1.156	13.40*	100.14	118.25	1.94 @ 637 nm<br>1.91 @ 829 nm<br>1.89 @ 1306 nm<br>1.89 @ 1549 nm	
29	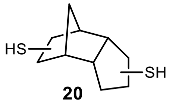 **20** poly(S-*r*-TCDSH) S70-TCDSH30	80.53* (78.82**)	1.094	7.29<br>9.20*	17	38	1.85 @ 637 nm<br>1.83 @ 828 nm<br>1.82 @ 1306 nm<br>1.81 @ 1549 nm	• Toxic H_2S production during polymer synthesis<br>• Imaging of hotplate demonstrated through polymer window<br>• Demonstrated imaging of a person/finger through polymer window
29	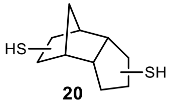 **20** poly(S-*r*-TCDSH) S60-TCDSH40	71.98* (70.74**)	1.001	5.64<br>6.64*	26	46	1.82 @ 637 nm<br>1.81 @ 828 nm<br>1.79 @ 1306 nm<br>1.79 @ 1549 nm	
29	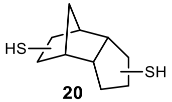 **20** poly(S-*r*-TCDSH) S50-TCDSH50	65.35* (62.06**)	1.028	4.88<br>5.58*	37	59	1.77 @ 637 nm<br>1.75 @ 828 nm<br>1.74 @ 1306 nm<br>1.74 @ 1549 nm	
30	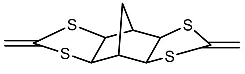 **18** poly(S-*r*-DMMD30)	84.1 (83.21*)	1.137	5.29<br>6.26*	95.1	98.3	1.93 @ 637 nm<br>1.91 @ 829 nm<br>1.89 @ 1306 nm<br>1.89 @ 1549 nm	• Polymer windows and Fresnel lens made through hot pressing<br>• LWIR imaging of hot plate demonstrated through polymer window<br>• Focusing amplification demonstrated through Fresnel lens<br>• No decrease in transmission after 15 days of storage at room temperature
30	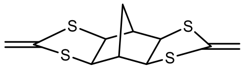 **18** poly(S-*r*-DMMD40)	78.8 (77.86*)	1.106	2.92	107.9	114.5	1.88 @ 637 nm<br>1.86 @ 829 nm<br>1.85 @ 1306 nm<br>1.84 @ 1549 nm	
30	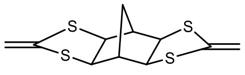 **18** poly(S-*r*-DMMD50)	73.5 (73.16*)	1.114	1.52	116.2	119.8	1.83 @ 637 nm<br>1.80 @ 829 nm<br>1.80 @ 1306 nm<br>1.80 @ 1549 nm	
22	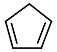 **22**, gas 50-poly(S-*r*-CPD)	50	1.00	9.0	-12.1	-	-	• Polymer sheet made using hot pressing.<br>• LWIR imaging at ambient temperature through polymer sheet

22	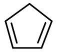 **22**, liquid<br>67-poly(S-*r*-CPD)	67	1.00	8.3	3.1	-	1.88 average over 7–14 µm	• Polymer sheet made using hot pressing.<br>• LWIR imaging at ambient temperature through polymer sheet<br>• Fresnel, plano convex, and plano concave lenes made, and LWIR imaging of hotplate and/or soldering iron demonstrated
22	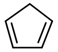 **22**, liquid<br>50-poly(S-*r*-CPD)	50	1.00	3.71	41.3	-	-	
31	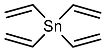 **21**<br>poly(S-*r*-TVSn)	70	1.55	6.64*	1.31 (1.95 after one week)	-	1.98 @ 636 nm<br>1.94 @ 984 nm<br>1.92 @ 1548 nm	• Initially rubbery material becomes brittle after a few days.<br>• Some unreacted S_8 observed by DSC.<br>• Able to view a hot soldering iron through window.<br>• Rapid decomposition and gas evolution has been observed during polymer synthesis – runaway reaction.<br>• Bubbles observed in polymer.
32	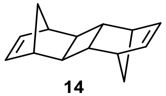 **14**<br>poly(S_{70}-*r*-$NBD2_{30}$)	70	1.05 ± 0.02	4.53*	-	100.9 ± 3.3	1.83 @ 634 nm*<br>1.82 @ 817 nm*<br>1.80 @ 1305 nm*<br>1.80 @ 1555 nm*	• Imaging of hotplate demonstrated through polymer window<br>• Diamond polishing of polymer demonstrated
32	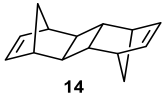 **14**<br>poly(S_{50}-*r*-$NBD2_{50}$)	50	-	-	-	104.5 ± 8.2	1.77 @ 634 nm*<br>1.75 @ 816 nm*<br>1.74 @ 1305 nm*<br>1.74 @ 1551 nm*	• LWIR imaging of hotplate demonstrated through Fresnel lens serving as the sole refractive optical element in a LWIR camera
33	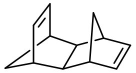 **16**<br>poly(S-*r*-Stillene)	70	-	-	134	-	1.91 @ 532 nm*<br>1.89 @ 633 nm*<br>1.86 @ 816 nm*<br>1.85 @ 1305 nm*<br>1.84 @ 1554 nm*	
33	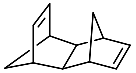 **16**<br>poly(S-*r*-Stillene)	50	1.0	1.4<br>1.32*	141	-	1.73 @ 634 nm*<br>1.72 @ 817 nm*<br>1.71 @ 1305 nm*<br>1.71 @ 1554 nm*	• LWIR imaging of hotplate demonstrated through Fresnel lens serving as the sole refractive optical element in a LWIR camera
28	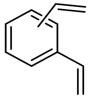 poly(S-r-DVB)<br>S90-DVB10	90<br>(93.6*)	1.083	4.32<br>4.34*	-	-	1.95 @ 637 nm<br>1.93 @ 829 nm<br>1.91 @ 1306 nm<br>1.90 @ 1549 nm	

28	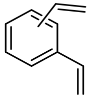 poly(S-r-DVB) S85-DVB15	85 (88.3*)	1.148	1.57*	-	-	1.94 @ 637 nm<br>1.91 @ 829 nm<br>1.89 @ 1306 nm<br>1.88 @ 1549 nm	
28	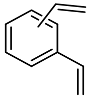 poly(S-r-DVB) S80-DVB20	80 (85.1*)	1.266	0.75*	-	-	1.92 @ 637 nm<br>1.89 @ 829 nm<br>1.87 @ 1306 nm<br>1.87 @ 1549 nm	
28	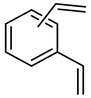 poly(S-r-DVB) S70-DVB30	70 (74.0*)	1.276	0.24<br>0.16*	20.43	-	1.87 @ 637 nm<br>1.84 @ 829 nm<br>1.82 @ 1306 nm<br>1.82 @ 1549 nm	• No decrease in transmission was observed after 30 days at room temperature.
34	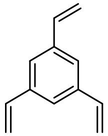 poly(S_{90}-r-TVB_{10})	90	1.1	5.41 ± 0.6	19.4, 137.8	47.1 ± 3.1	2.00 @ 532 nm<br>1.97 @ 633 nm<br>1.94 @ 829 nm	
34	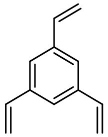 poly(S_{85}-r-TVB_{15})	85	1.1	1.62 ± 0.5	47.6, 138.6	50.7 ± 3.3	1.99 @ 532 nm<br>1.95 @ 633 nm<br>1.92 @ 829 nm	
34	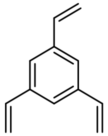 poly(S_{80}-r-TVB_{20})	80	1.1	1.31 ± 0.4	92.6, 139.8	91.6 ± 1.2	1.95 @ 532 nm<br>1.92 @ 633 nm<br>1.88 @ 829 nm	
34	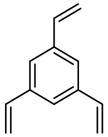 poly(S_{75}-r-TVB_{25})	75	-	-	148.6	-	-	

34	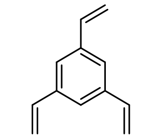 poly(S_{70}-r-TVB_{30})	70	-	-	173.1	-	-	
28	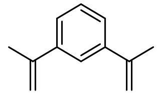 poly(S-*r*-DIB) S70-DIB30	70 (73.3*)	1.281	0.08	13.96	-	1.86 @ 637 nm	• A decrease in transmission was observed after 30 days at room temperature.
30	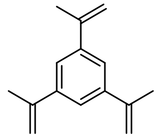 poly(S-r-TIB)	70	1.119	0.12	51.2 (Ref 9)	68 (Ref 9)	-	
28	Poly(methyl methacrylate)	-	1.153	0.05	-	-	1.49 @ 637 nm	
28	Low density polyethylene	-	1.090	14.32	-	-	1.51 @ 637 nm	
28	Germanium	-	1.087	42.37	-	-	4.00 over 2–14 µm	• Note LWIR transmission loss is due to reflection. Anti-reflective coatings applied to germanium result in LWIR transmission > 90% across 7–14 µm

Supplementary references

1 Cowieson, N. P. *et al.* MX1: a bending-magnet crystallography beamline serving both chemical and macromolecular crystallography communities at the Australian Synchrotron. *J. Synchrotron Radiat.* **22**, 187–190 (2015).

2 Aragão, D. *et al.* MX2: a high-flux undulator microfocus beamline serving both the chemical and macromolecular crystallography communities at the Australian Synchrotron. *J. Synchrotron Radiat.* **25**, 885–891 (2018).

3 Sheldrick, G. M. SHELXT - Integrated space-group and crystal-structure determination. *Acta Crystallogr. A* **71**, 3–8 (2015).

4 Sheldrick, G. M. Crystal structure refinement with SHELXL. *Acta Crystallogr. C Struct. Chem.* **71**, 3–8 (2015).

5 Hübschle, C. B., Sheldrick, G. M. & Dittrich, B. ShelXle: a Qt graphical user interface for SHELXL. *J. Appl. Crystallogr.* **44**, 1281–1284 (2011).

6 Mazumder, K., Voit, B. & Banerjee, S. Recent Progress in Sulfur-Containing High Refractive Index Polymers for Optical Applications. *ACS Omega* **9**, 6253–6279 (2024).

7 Bartlett, P. D. & Ghosh, T. Sulfuration of the norbornene double bond. *J. Org. Chem.* **52**, 4937–4943 (1987).

8 Smith, J. A., Wu, X., Berry, N. G. & Hasell, T. High sulfur content polymers: The effect of crosslinker structure on inverse vulcanization. *J. Polym. Sci., Part A: Polym. Chem.* **56**, 1777–1781 (2018).

9 Kleine, T. S. *et al.* High Refractive Index Copolymers with Improved Thermomechanical Properties via the Inverse Vulcanization of Sulfur and 1,3,5-Triisopropenylbenzene. *ACS Macro Lett.* **5**, 1152–1156 (2016).

10 Zheng, B. *et al.* Structural evolution during inverse vulcanization. *Nat. Commun.* **15**, 5507 (2024).

11 Dodd, L. J. *et al.* Raman analysis of inverse vulcanised polymers. *Polym. Chem.* **14**, 1369–1386 (2023).

12 Ward, A. T. Raman spectroscopy of sulfur, sulfur-selenium, and sulfur-arsenic mixtures. *J. Phys. Chem.* **72**, 4133–4139 (1968).

13 Chai, J.-D. & Head-Gordon, M. Long-range corrected hybrid density functionals with damped atom–atom dispersion corrections. *Phys. Chem. Chem. Phys.* **10**, 6615–6620 (2008).

14 Petersson, G. A. *et al.* A complete basis set model chemistry. I. The total energies of closed-shell atoms and hydrides of the first-row elements. *J. Chem. Phys.* **89**, 2193–2218 (1988).

15 Frisch, M. J. *et al.* Gaussian 16 Revision C.01. (2016).

16 Pracht, P., Bohle, F. & Grimme, S. Automated exploration of the low-energy chemical space with fast quantum chemical methods. *Phys. Chem. Chem. Phys.* **22**, 7169–7192 (2020).

17 Abbott, L. J., Hart, K. E. & Colina, C. M. Polymatic: a generalized simulated polymerization algorithm for amorphous polymers. *Theor. Chem. Acc.* **132**, 1334 (2013).

18 Thompson, A. P. *et al.* LAMMPS - a flexible simulation tool for particle-based materials modeling at the atomic, meso, and continuum scales. *Comput. Phys. Commun.* **271**, 108171 (2022).

19 Bannwarth, C., Ehlert, S. & Grimme, S. GFN2-xTB—An Accurate and Broadly Parametrized Self-Consistent Tight-Binding Quantum Chemical Method with

Multipole Electrostatics and Density-Dependent Dispersion Contributions. *J. Chem. Theory Comput.* **15**, 1652–1671 (2019).
20 Bannwarth, C. *et al.* Extended tight-binding quantum chemistry methods. *Wiley Interdiscip. Rev. Comput. Mol.* **11**, e1493 (2021).
21 Alecu, I. M., Zheng, J., Zhao, Y. & Truhlar, D. G. Computational Thermochemistry: Scale Factor Databases and Scale Factors for Vibrational Frequencies Obtained from Electronic Model Chemistries. *J. Chem. Theory Comput.* **6**, 2872–2887 (2010).
22 Tonkin, S. J. *et al.* Thermal Imaging and Clandestine Surveillance using Low-Cost Polymers with Long-Wave Infrared Transparency. *Adv. Optical Mater.* **11**, 2300058 (2023).
23 Dennington, R.; Keith, T. A.; Millam, J. M. GaussView Version 6. (2019).
24 Wheeler, C. C. U.S. Air Force Aerial Camera Resolution Tester. *Opt. Eng.* **14**, 142120 (1975).
25 Goncalves, D. P. & Griffith, D. J. Estimating uncertainty in resolution tests. *Opt. Eng.* **45**, 053601 (2006).
26 Molineux, J. *et al.* Fabrication of Plastic Optics from Chalcogenide Hybrid Inorganic/Organic Polymers for Infrared Thermal Imaging. *Adv. Optical Mater.* **12**, 2301971 (2024).
27 Fakhri, S. A., Motayyeb, S., Saadatseresht, M., Zakeri, H. & Mousavi, V. Comparison of UAV Image Spatial Resolution Based on the Siemens Star Target. *ISPRS Ann. Photogramm. Remote Sens. Spatial Inf. Sci.* **X-4/W1-2022**, 143–150 (2023).
28. Lee, M. *et al.* Long-wave infrared transparent sulfur polymers enabled by symmetric thiol cross-linker. *Nat. Commun.* **14**, 2866 (2023).
29. Lee, M. *et al.* Structural Effect of Cyclic Olefin Cross-Linkers on Long-Wave Infrared-Transmitting Sulfur Polymers. *Macromolecules* **57**, 2905–2914 (2024).
30. Wuliu, Y. *et al.* Sulfur-Rich Norbornadiene-Derived Infrared Transparent Polymers by Inverse Vulcanization. *Angew. Chem. Int. Ed.* **64**, e202419446 (2024).
31. Boyd, D. A. *et al.* Optical Properties of a Sulfur-Rich Organically Modified Chalcogenide Polymer Synthesized via Inverse Vulcanization and Containing an Organometallic Comonomer. *ACS Macro Lett.* **8**, 113–116 (2019).
32. Kleine, T. S. *et al.* Infrared Fingerprint Engineering: A Molecular-Design Approach to Long-Wave Infrared Transparency with Polymeric Materials. *Angew. Chem. Int. Ed.* **58**, 17656 –17660 (2019).
33. Marshall, C. M. *et al.* Synthesis of Polycyclic Olefinic Monomers from Norbornadiene for Inverse Vulcanization: Structural and Mechanistic Consequences. *J. Am. Chem. Soc.* **146**, 24061–24074 (2024).
34. Hwang, J. H. *et al.* A Microphase Separation Strategy for the Infrared Transparency-Thermomechanical Property Conundrum in Sulfur-Rich Copolymers. *Adv. Optical Mater.* **11**, 2202432 (2023).
