# Supplementary material for: Thermal imaging using sulfur polymer optics
Source: Nat Commun. 2026 Feb 18;17:1561. doi: 10.1038/s41467-026-68889-0 (PMC12916755; doi:10.1038/s41467-026-68889-0)
Supplement: Supplementary file 2 — Description of Additional Supplementary File [file 41467_2026_68889_MOESM2_ESM.pdf]

The Description of Additional Supplementary Files

Supplementary Movie 1: Illustrates the use of lens 6 and the FLIR Lepton 3.5 in capturing video of the operation of a kitchen appliance.
