# Supplementary material for: Thermal imaging using sulfur polymer optics
Source: Nat Commun. 2026 Feb 18;17:1561. doi: 10.1038/s41467-026-68889-0 (PMC12916755; doi:10.1038/s41467-026-68889-0)

checkCIF/PLATON report

You have not supplied any structure factors. As a result the full set of tests cannot be run.

THIS REPORT IS FOR GUIDANCE ONLY. IF USED AS PART OF A REVIEW PROCEDURE FOR PUBLICATION, IT SHOULD NOT REPLACE THE EXPERTISE OF AN EXPERIENCED CRYSTALLOGRAPHIC REFEREE.

No syntax errors found. CIF dictionary Interpreting this report

Datablock: BCHDTS

Bond precision: C-C = 0.0020 A Wavelength=0.71073

Cell:	a=6.7640(14)	b=13.008(3)	c=12.204(2)
	alpha=90	beta=100.82(3)	gamma=90
Temperature:	100 K		

	Calculated	Reported
Volume	1054.7(4)	1054.7(4)
Space group	P 21/c	P 21/c
Hall group	-P 2ybc	-P 2ybc
Moiety formula	C7 H8 S6	?
Sum formula	C7 H8 S6	C7 H8 S6
Mr	284.49	284.49
Dx,g cm-3	1.792	1.792
Z	4	4
Mu (mm-1)	1.243	1.243
F000	584.0	584.0
F000'	587.06	
h,k,lmax	8,17,16	8,17,16
Nref	2511	2510
Tmin,Tmax	0.740,0.800	
Tmin'	0.726	

Correction method= Not given

Data completeness= 1.000 Theta(max)= 27.882

R(reflections)= 0.0301(2476) wR2(reflections)= 0.0768(2510)

S = 1.080 Npar= 119

The following ALERTS were generated. Each ALERT has the format
test-name_ALERT_alert-type_alert-level.
Click on the hyperlinks for more details of the test.

Alert level G

```
ABSMU01_ALERT_1_G  Calculation of _exptl_absorpt_correction_mu
                not performed for this radiation type.
PLAT012_ALERT_1_G N.O.K.   _shelx_res_checksum Found in CIF ......     Please Check
PLAT720_ALERT_4_G Number of Unusual/Non-Standard Labels .........          1 Note
              H4AB
PLAT793_ALERT_4_G Model has Chirality at C1          (Centro SpGr)          S Verify
PLAT793_ALERT_4_G Model has Chirality at C2          (Centro SpGr)          R Verify
PLAT793_ALERT_4_G Model has Chirality at C6          (Centro SpGr)          R Verify
PLAT793_ALERT_4_G Model has Chirality at C7          (Centro SpGr)          S Verify
PLAT883_ALERT_1_G Absent Datum for _atom_sites_solution_primary ..     Please Do !
PLAT899_ALERT_4_G SHELXL2018 is Outdated   and Succeeded by SHELXL     2019/3 Note
```

0 **ALERT level A** = Most likely a serious problem - resolve or explain
0 **ALERT level B** = A potentially serious problem, consider carefully
0 **ALERT level C** = Check. Ensure it is not caused by an omission or oversight
9 **ALERT level G** = General information/check it is not something unexpected

3 ALERT type 1 CIF construction/syntax error, inconsistent or missing data
0 ALERT type 2 Indicator that the structure model may be wrong or deficient
0 ALERT type 3 Indicator that the structure quality may be low
6 ALERT type 4 Improvement, methodology, query or suggestion
0 ALERT type 5 Informative message, check

It is advisable to attempt to resolve as many as possible of the alerts in all categories. Often the minor alerts point to easily fixed oversights, errors and omissions in your CIF or refinement strategy, so attention to these fine details can be worthwhile. In order to resolve some of the more serious problems it may be necessary to carry out additional measurements or structure refinements. However, the purpose of your study may justify the reported deviations and the more serious of these should normally be commented upon in the discussion or experimental section of a paper or in the "special_details" fields of the CIF. checkCIF was carefully designed to identify outliers and unusual parameters, but every test has its limitations and alerts that are not important in a particular case may appear. Conversely, the absence of alerts does not guarantee there are no aspects of the results needing attention. It is up to the individual to critically assess their own results and, if necessary, seek expert advice.

Publication of your CIF in IUCr journals

A basic structural check has been run on your CIF. These basic checks will be run on all CIFs submitted for publication in IUCr journals (*Acta Crystallographica*, *Journal of Applied Crystallography*, *Journal of Synchrotron Radiation*); however, if you intend to submit to *Acta Crystallographica Section C* or *E* or *IUCrData*, you should make sure that full publication checks are run on the final version of your CIF prior to submission.

Publication of your CIF in other journals

Please refer to the *Notes for Authors* of the relevant journal for any special instructions relating to CIF submission.

PLATON version of 02/02/2025; check.def file version of 02/02/2025

Datablock BCHDTS - ellipsoid plot

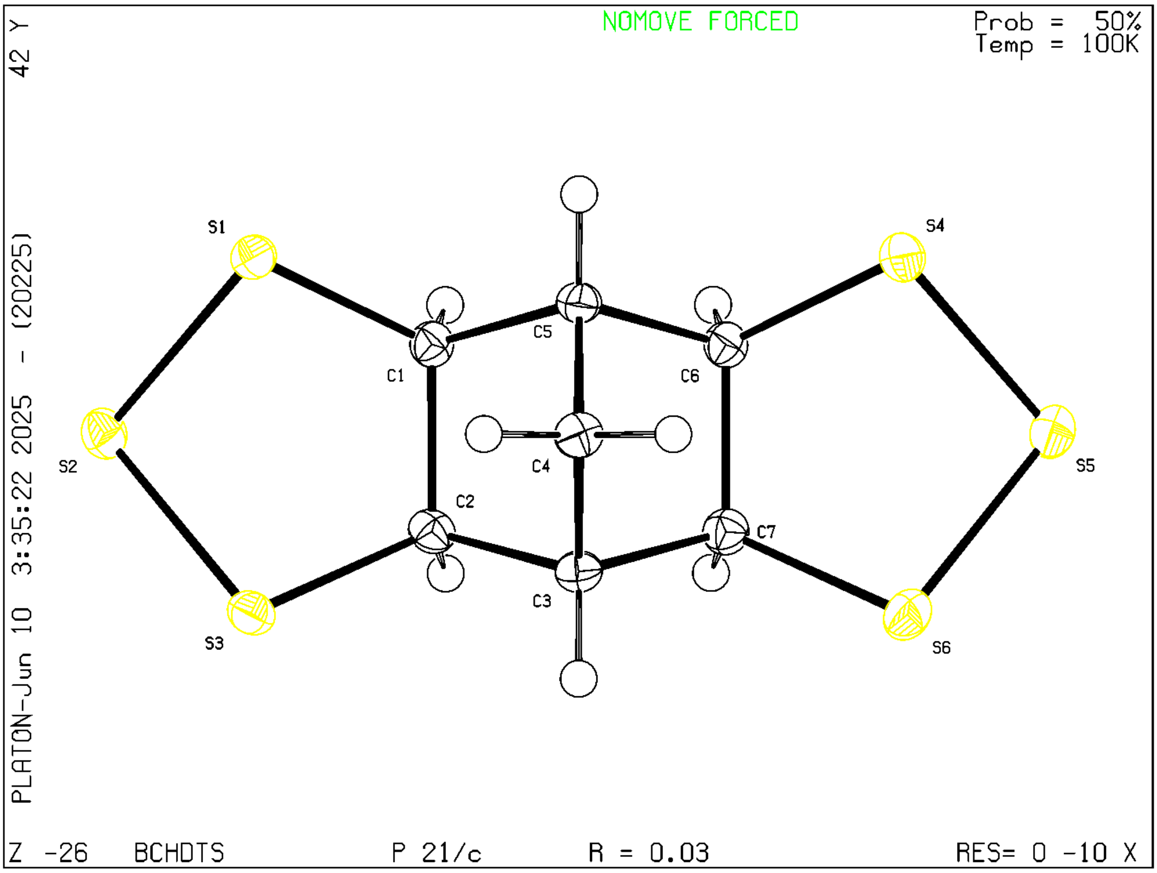

checkCIF/PLATON report

You have not supplied any structure factors. As a result the full set of tests cannot be run.

THIS REPORT IS FOR GUIDANCE ONLY. IF USED AS PART OF A REVIEW PROCEDURE FOR PUBLICATION, IT SHOULD NOT REPLACE THE EXPERTISE OF AN EXPERIENCED CRYSTALLOGRAPHIC REFEREE.

No syntax errors found. CIF dictionary Interpreting this report

Datablock: NBDTS

```
Bond precision:   C-C = 0.0019 A          Wavelength=0.71073

Cell:              a=42.045(8)      b=7.7730(16)      c=9.6930(19)
                   alpha=90         beta=90           gamma=90
Temperature:       100 K
```

	Calculated	Reported
Volume	3167.8(11)	3167.8(11)
Space group	P b c n	P b c n
Hall group	-P 2n 2ab	-P 2n 2ab
Moiety formula	C7 H8 S3	?
Sum formula	C7 H8 S3	C7 H8 S3
Mr	188.31	188.31
Dx,g cm-3	1.579	1.579
Z	16	16
Mu (mm-1)	0.849	0.849
F000	1568.0	1568.0
F000’	1574.26	
h,k,lmax	55,10,12	55,10,12
Nref	3783	3673
Tmin,Tmax	0.866,0.903	
Tmin’	0.866	

```
Correction method= Not given

Data completeness= 0.971            Theta(max)= 27.882

R(reflections)= 0.0300( 3542)                      wR2(reflections)=
                                                   0.0806( 3673)
S = 1.105                  Npar= 181
```

The following ALERTS were generated. Each ALERT has the format
test-name_ALERT_alert-type_alert-level.
Click on the hyperlinks for more details of the test.

Alert level G

```
ABSMU01_ALERT_1_G  Calculation of _exptl_absorpt_correction_mu
                not performed for this radiation type.
PLAT012_ALERT_1_G N.O.K.   _shelx_res_checksum Found in CIF ......     Please Check
PLAT720_ALERT_4_G Number of Unusual/Non-Standard Labels .........          1 Note
              H4AB
PLAT793_ALERT_4_G Model has Chirality at C1          (Centro SpGr)          R Verify
PLAT793_ALERT_4_G Model has Chirality at C2          (Centro SpGr)          S Verify
PLAT793_ALERT_4_G Model has Chirality at C3          (Centro SpGr)          S Verify
PLAT793_ALERT_4_G Model has Chirality at C5          (Centro SpGr)          R Verify
PLAT793_ALERT_4_G Model has Chirality at C21         (Centro SpGr)          R Verify
PLAT793_ALERT_4_G Model has Chirality at C22         (Centro SpGr)          S Verify
PLAT793_ALERT_4_G Model has Chirality at C23         (Centro SpGr)          S Verify
PLAT793_ALERT_4_G Model has Chirality at C25         (Centro SpGr)          R Verify
PLAT883_ALERT_1_G Absent Datum for _atom_sites_solution_primary ..     Please Do !
PLAT899_ALERT_4_G SHELXL2018 is Outdated   and Succeeded by SHELXL     2019/3 Note
PLAT965_ALERT_2_G The SHELXL WEIGHT Optimisation has not Converged     Please Check
```

   0 **ALERT level A** = Most likely a serious problem - resolve or explain
   0 **ALERT level B** = A potentially serious problem, consider carefully
   0 **ALERT level C** = Check. Ensure it is not caused by an omission or oversight
  14 **ALERT level G** = General information/check it is not something unexpected

```
   3 ALERT type 1 CIF construction/syntax error, inconsistent or missing data
   1 ALERT type 2 Indicator that the structure model may be wrong or deficient
   0 ALERT type 3 Indicator that the structure quality may be low
  10 ALERT type 4 Improvement, methodology, query or suggestion
   0 ALERT type 5 Informative message, check
```

It is advisable to attempt to resolve as many as possible of the alerts in all categories. Often the minor alerts point to easily fixed oversights, errors and omissions in your CIF or refinement strategy, so attention to these fine details can be worthwhile. In order to resolve some of the more serious problems it may be necessary to carry out additional measurements or structure refinements. However, the purpose of your study may justify the reported deviations and the more serious of these should normally be commented upon in the discussion or experimental section of a paper or in the "special_details" fields of the CIF. checkCIF was carefully designed to identify outliers and unusual parameters, but every test has its limitations and alerts that are not important in a particular case may appear. Conversely, the absence of alerts does not guarantee there are no aspects of the results needing attention. It is up to the individual to critically assess their own results and, if necessary, seek expert advice.

Publication of your CIF in IUCr journals

A basic structural check has been run on your CIF. These basic checks will be run on all CIFs submitted for publication in IUCr journals (*Acta Crystallographica*, *Journal of Applied Crystallography*, *Journal of Synchrotron Radiation*); however, if you intend to submit to *Acta Crystallographica Section C* or *E* or *IUCrData*, you should make sure that full publication checks are run on the final version of your CIF prior to submission.

Publication of your CIF in other journals

Please refer to the *Notes for Authors* of the relevant journal for any special instructions relating to CIF submission.

PLATON version of 02/02/2025; check.def file version of 02/02/2025

Datablock NBDTS - ellipsoid plot

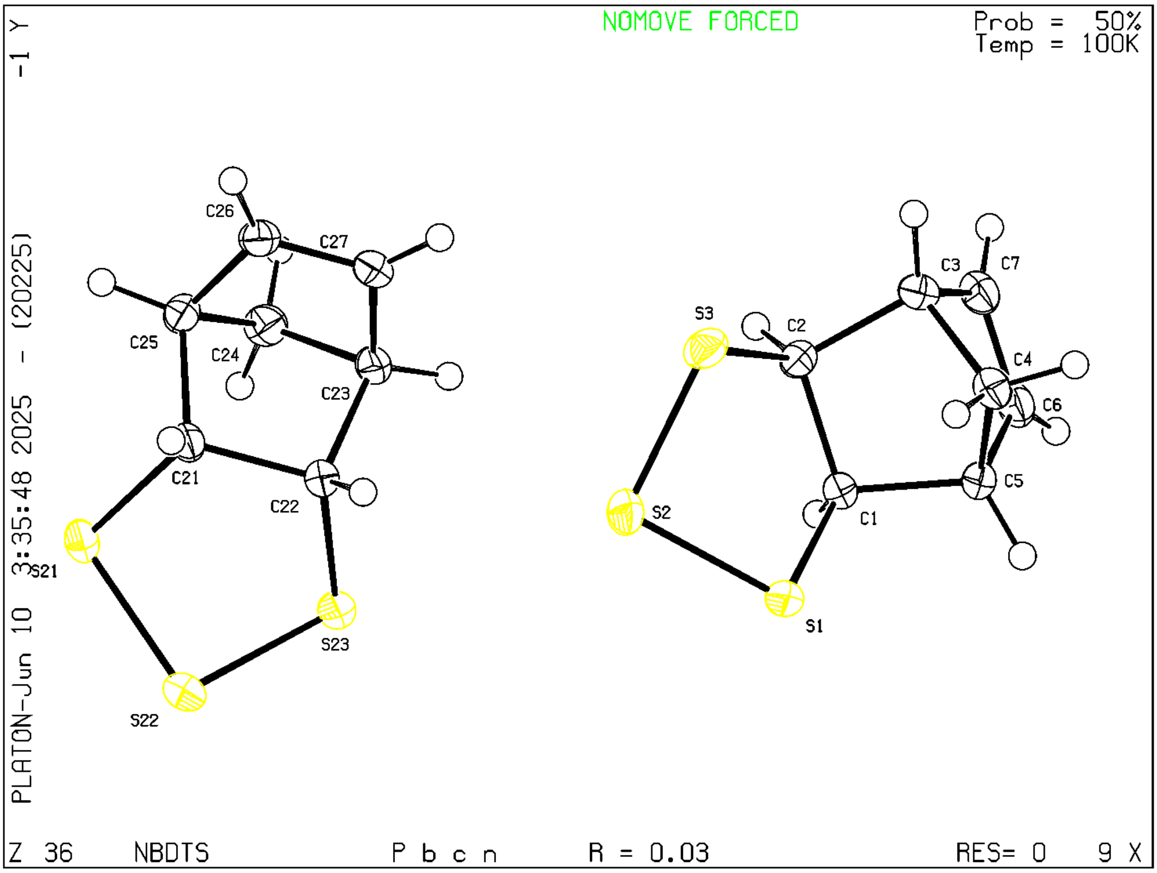

checkCIF/PLATON report

You have not supplied any structure factors. As a result the full set of tests cannot be run.

THIS REPORT IS FOR GUIDANCE ONLY. IF USED AS PART OF A REVIEW PROCEDURE FOR PUBLICATION, IT SHOULD NOT REPLACE THE EXPERTISE OF AN EXPERIENCED CRYSTALLOGRAPHIC REFEREE.

No syntax errors found. CIF dictionary Interpreting this report

Datablock: ReNBDS

```
Bond precision:   C-C = 0.0023 A          Wavelength=0.71073

Cell:              a=5.8750(12)      b=9.2290(18)        c=6.527(2)
                   alpha=90          beta=108.75(3)      gamma=90
Temperature:       100 K

                 Calculated                   Reported
Volume           335.12(15)                   335.12(15)
Space group      P 21/m                       P 21/m
Hall group       -P 2yb                       -P 2yb
Moiety formula   C7 H8 S2                     ?
Sum formula      C7 H8 S2                     C7 H8 S2
Mr               156.25                       156.25
Dx,g cm-3        1.548                        1.549
Z                2                            2
Mu (mm-1)        0.686                        0.686
F000             164.0                        164.0
F000'            164.53
h,k,lmax         7,12,8                       7,11,7
Nref             903                          720
Tmin,Tmax        0.936,0.953
Tmin'            0.860

Correction method= Not given

Data completeness= 0.797          Theta(max)= 28.506

R(reflections)= 0.0305( 710)                       wR2(reflections)=
                                                   0.0902( 720)
S = 1.083                 Npar= 46
```

The following ALERTS were generated. Each ALERT has the format
test-name_ALERT_alert-type_alert-level.
Click on the hyperlinks for more details of the test.

Alert level C

```
PLAT029_ALERT_3_C _diffrn_measured_fraction_theta_full value Low .      0.966 Why?
```

Author Response: Due to geometry constraints of the endstation only a single omega swee

Alert level G

```
ABSMU01_ALERT_1_G  Calculation of _exptl_absorpt_correction_mu
                not performed for this radiation type.
PLAT012_ALERT_1_G N.O.K.   _shelx_res_checksum Found in CIF ......     Please Check
PLAT299_ALERT_4_G Atom Site Occupancy Constrained at ............        0.5 Check
              H4AB    H4A
PLAT367_ALERT_2_G Long?  C(sp?)-C(sp?) Bond  C2       - C4       .       1.53 Ang.
PLAT367_ALERT_2_G Long?  C(sp?)-C(sp?) Bond  C4       - C5       .       1.52 Ang.
PLAT720_ALERT_4_G Number of Unusual/Non-Standard Labels .........          1 Note
              H4AB
PLAT793_ALERT_4_G Model has Chirality at C1          (Centro SpGr)         R Verify
PLAT793_ALERT_4_G Model has Chirality at C6          (Centro SpGr)         S Verify
PLAT802_ALERT_4_G CIF Input Record(s) with more than 80 Characters         1 Info
PLAT883_ALERT_1_G Absent Datum for _atom_sites_solution_primary ..     Please Do !
PLAT899_ALERT_4_G SHELXL2018 is Outdated   and Succeeded by SHELXL     2019/3 Note
```

---

0 **ALERT level A** = Most likely a serious problem - resolve or explain
0 **ALERT level B** = A potentially serious problem, consider carefully
1 **ALERT level C** = Check. Ensure it is not caused by an omission or oversight
11 **ALERT level G** = General information/check it is not something unexpected

3 ALERT type 1 CIF construction/syntax error, inconsistent or missing data
2 ALERT type 2 Indicator that the structure model may be wrong or deficient
1 ALERT type 3 Indicator that the structure quality may be low
6 ALERT type 4 Improvement, methodology, query or suggestion
0 ALERT type 5 Informative message, check

It is advisable to attempt to resolve as many as possible of the alerts in all categories. Often the minor alerts point to easily fixed oversights, errors and omissions in your CIF or refinement strategy, so attention to these fine details can be worthwhile. In order to resolve some of the more serious problems it may be necessary to carry out additional measurements or structure refinements. However, the purpose of your study may justify the reported deviations and the more serious of these should normally be commented upon in the discussion or experimental section of a paper or in the "special_details" fields of the CIF. checkCIF was carefully designed to identify outliers and unusual parameters, but every test has its limitations and alerts that are not important in a particular case may appear. Conversely, the absence of alerts does not guarantee there are no aspects of the results needing attention. It is up to the individual to critically assess their own results and, if necessary, seek expert advice.

Publication of your CIF in IUCr journals

A basic structural check has been run on your CIF. These basic checks will be run on all CIFs submitted for publication in IUCr journals (*Acta Crystallographica*, *Journal of Applied Crystallography*, *Journal of Synchrotron Radiation*); however, if you intend to submit to *Acta Crystallographica Section C* or *E* or *IUCrData*, you should make sure that full publication checks are run on the final version of your CIF prior to submission.

Publication of your CIF in other journals

Please refer to the *Notes for Authors* of the relevant journal for any special instructions relating to CIF submission.

PLATON version of 02/02/2025; check.def file version of 02/02/2025

Datablock ReNBDS - ellipsoid plot

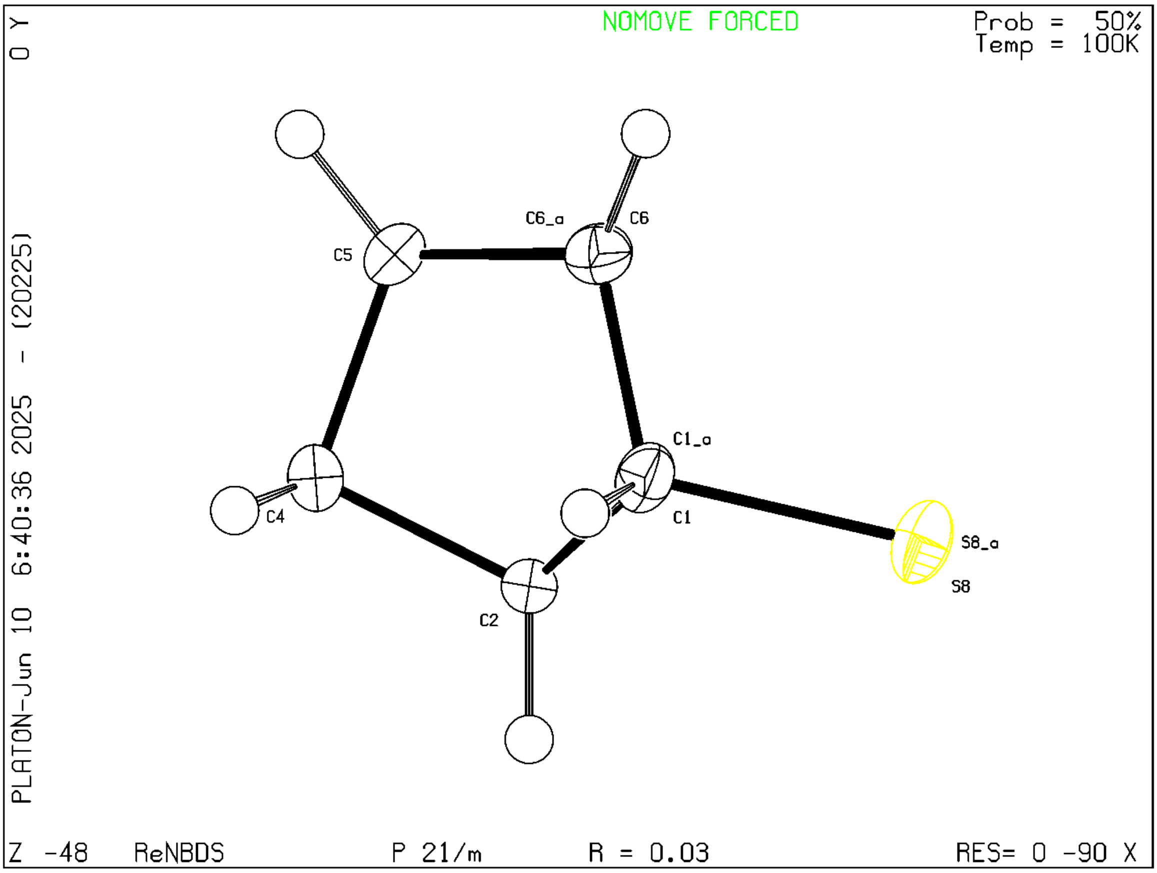

checkCIF/PLATON report

You have not supplied any structure factors. As a result the full set of tests cannot be run.

THIS REPORT IS FOR GUIDANCE ONLY. IF USED AS PART OF A REVIEW PROCEDURE FOR PUBLICATION, IT SHOULD NOT REPLACE THE EXPERTISE OF AN EXPERIENCED CRYSTALLOGRAPHIC REFEREE.

No syntax errors found. CIF dictionary Interpreting this report

Datablock: sol_a

```
Bond precision:   C-C = 0.0038 A          Wavelength=0.71073

Cell:            a=9.0240(18)      b=9.921(2)       c=11.724(2)
                 alpha=87.16(3)    beta=71.21(3)    gamma=86.85(3)
Temperature:     100 K

                 Calculated                  Reported
Volume           991.6(4)                    991.6(4)
Space group      P -1                        P -1
Hall group       -P 1                        -P 1
Moiety formula   C7 H8 S5                    ?
Sum formula      C7 H8 S5                    C7 H8 S5
Mr               252.43                      252.43
Dx,g cm-3        1.691                       1.691
Z                4                           4
Mu (mm-1)        1.107                       1.108
F000             520.0                       520.0
F000'            522.56
h,k,lmax         12,13,15                    10,10,13
Nref             5078                        3433
Tmin,Tmax        0.853,0.885
Tmin'            0.775

Correction method= Not given

Data completeness= 0.676          Theta(max)= 28.597

                                                  wR2(reflections)=
R(reflections)= 0.0361( 3247)                     0.0990( 3433)

S = 1.081                Npar= 217
```

The following ALERTS were generated. Each ALERT has the format
test-name_ALERT_alert-type_alert-level.
Click on the hyperlinks for more details of the test.

Alert level A

PLAT029_ALERT_3_A _diffrn_measured_fraction_theta_full value Low . 0.874 Why?

Author Response: the residual positive density is not related to disorder, and we expec

Alert level C

DIFMX02_ALERT_1_C The maximum difference density is > 0.1*ZMAX*0.75
The relevant atom site should be identified.

Author Response: The residual density is nearest to the sulfur atoms, however, it does

PLAT094_ALERT_2_C Ratio of Maximum / Minimum Residual Density .... 2.61 Report

Author Response: the residual positive density is not related to disorder, and we expec

PLAT097_ALERT_2_C Large Reported Max. (Positive) Residual Density 1.21 eA-3

Author Response: the residual positive density is not related to disorder, and we expec

Alert level G

```
ABSMU01_ALERT_1_G  Calculation of _exptl_absorpt_correction_mu
                not performed for this radiation type.
PLAT012_ALERT_1_G N.O.K.   _shelx_res_checksum Found in CIF ......     Please Check
PLAT154_ALERT_1_G The s.u.'s on the Cell Angles are Equal ..(Note)       0.03 Degree
PLAT720_ALERT_4_G Number of Unusual/Non-Standard Labels .........          1 Note
              H6AB
PLAT793_ALERT_4_G Model has Chirality at C1          (Centro SpGr)         S Verify
PLAT793_ALERT_4_G Model has Chirality at C2          (Centro SpGr)         S Verify
PLAT793_ALERT_4_G Model has Chirality at C5          (Centro SpGr)         R Verify
PLAT793_ALERT_4_G Model has Chirality at C7          (Centro SpGr)         R Verify
PLAT793_ALERT_4_G Model has Chirality at C21         (Centro SpGr)         R Verify
PLAT793_ALERT_4_G Model has Chirality at C22         (Centro SpGr)         R Verify
PLAT793_ALERT_4_G Model has Chirality at C25         (Centro SpGr)         S Verify
PLAT793_ALERT_4_G Model has Chirality at C27         (Centro SpGr)         S Verify
PLAT802_ALERT_4_G CIF Input Record(s) with more than 80 Characters         4 Info
PLAT883_ALERT_1_G Absent Datum for _atom_sites_solution_primary ..     Please Do !
PLAT899_ALERT_4_G SHELXL2018 is Outdated   and Succeeded by SHELXL     2019/3 Note
PLAT941_ALERT_3_G Average HKL Measurement Multiplicity ..........        3.7 Low
PLAT950_ALERT_5_G Calculated (ThMax) and CIF-Reported Hmax Differ          2 Units
PLAT951_ALERT_5_G Calculated (ThMax) and CIF-Reported Kmax Differ          3 Units
PLAT952_ALERT_5_G Calculated (ThMax) and CIF-Reported Lmax Differ.         2 Units
```

```
PLAT965_ALERT_2_G The SHELXL WEIGHT Optimisation has not Converged     Please Check
```

```
  1 ALERT level A = Most likely a serious problem - resolve or explain
  0 ALERT level B = A potentially serious problem, consider carefully
  3 ALERT level C = Check. Ensure it is not caused by an omission or oversight
 20 ALERT level G = General information/check it is not something unexpected

  5 ALERT type 1 CIF construction/syntax error, inconsistent or missing data
  3 ALERT type 2 Indicator that the structure model may be wrong or deficient
  2 ALERT type 3 Indicator that the structure quality may be low
 11 ALERT type 4 Improvement, methodology, query or suggestion
  3 ALERT type 5 Informative message, check
```

It is advisable to attempt to resolve as many as possible of the alerts in all categories. Often the minor alerts point to easily fixed oversights, errors and omissions in your CIF or refinement strategy, so attention to these fine details can be worthwhile. In order to resolve some of the more serious problems it may be necessary to carry out additional measurements or structure refinements. However, the purpose of your study may justify the reported deviations and the more serious of these should normally be commented upon in the discussion or experimental section of a paper or in the "special_details" fields of the CIF. checkCIF was carefully designed to identify outliers and unusual parameters, but every test has its limitations and alerts that are not important in a particular case may appear. Conversely, the absence of alerts does not guarantee there are no aspects of the results needing attention. It is up to the individual to critically assess their own results and, if necessary, seek expert advice.

Publication of your CIF in IUCr journals

A basic structural check has been run on your CIF. These basic checks will be run on all CIFs submitted for publication in IUCr journals (*Acta Crystallographica*, *Journal of Applied Crystallography*, *Journal of Synchrotron Radiation*); however, if you intend to submit to *Acta Crystallographica Section C* or *E* or *IUCrData*, you should make sure that full publication checks are run on the final version of your CIF prior to submission.

Publication of your CIF in other journals

Please refer to the *Notes for Authors* of the relevant journal for any special instructions relating to CIF submission.

PLATON version of 02/02/2025; check.def file version of 02/02/2025

Datablock sol_a - ellipsoid plot

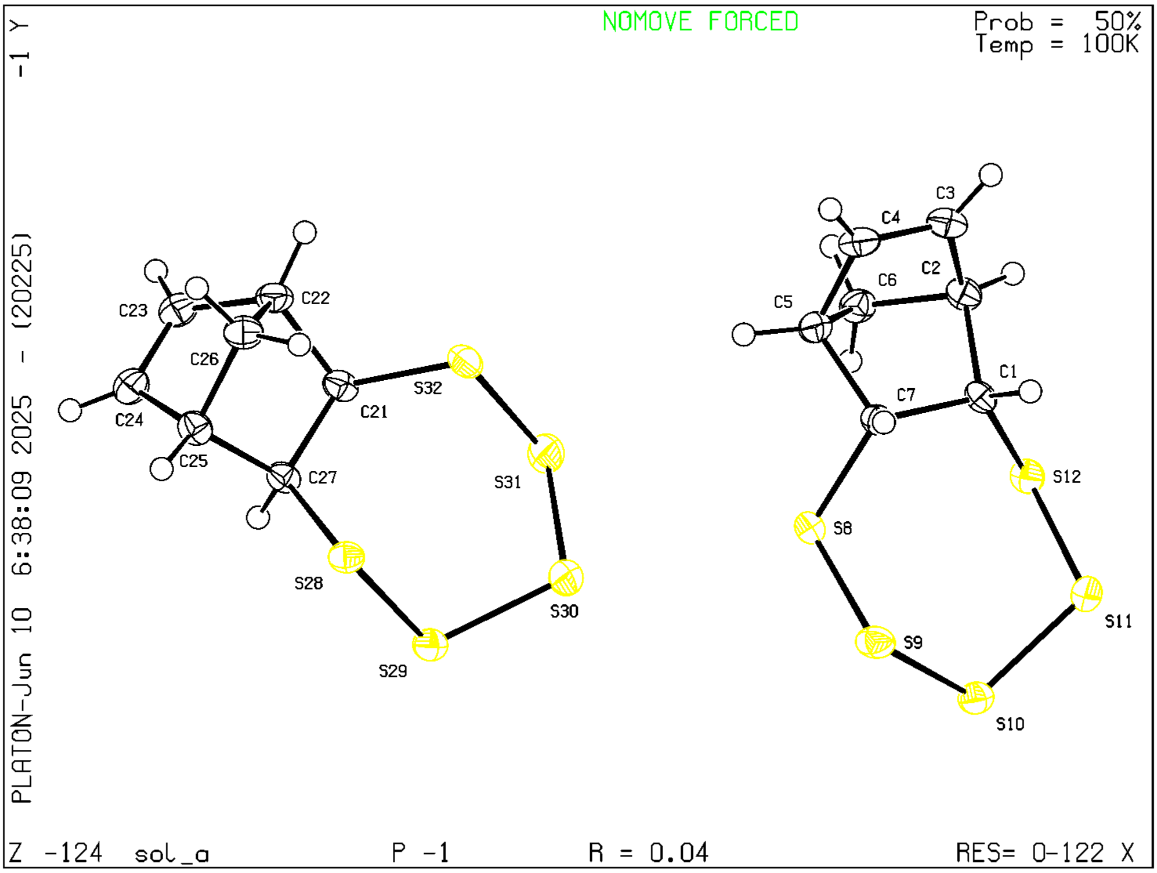

Supplement: Supplementary file 5 — Source Data [file 41467_2026_68889_MOESM5_ESM.zip › Figure 1 Source Data/checkCIF_combined_IR_paper.pdf]
